# Diversity Forests: Using Split Sampling to Enable Innovative Complex Split Procedures in Random Forests

**DOI:** 10.1007/s42979-021-00920-1

**Published:** 2021-10-21

**Authors:** Roman Hornung

**Affiliations:** Institute for Medical Information Processing, Biometry and Epidemiology, University of Munich, Marchioninistr. 15, 81377 Munich, Germany

**Keywords:** Random forests, Ensemble learning, Classification, Decision trees

## Abstract

The diversity forest algorithm is an alternative candidate node split sampling scheme that makes innovative complex split procedures in random forests possible. While conventional univariable, binary splitting suffices for obtaining strong predictive performance, new complex split procedures can help tackling practically important issues. For example, interactions between features can be exploited effectively by bivariable splitting. With diversity forests, each split is selected from a candidate split set that is sampled in the following way: for $l = 1, \dots , {nsplits}$: (1) sample one split problem; (2) sample a single or few splits from the split problem sampled in (1) and add this or these splits to the candidate split set. The split problems are specifically structured collections of splits that depend on the respective split procedure considered. This sampling scheme makes innovative complex split procedures computationally tangible while avoiding overfitting. Important general properties of the diversity forest algorithm are evaluated empirically using univariable, binary splitting. Based on 220 data sets with binary outcomes, diversity forests are compared with conventional random forests and random forests using extremely randomized trees. It is seen that the split sampling scheme of diversity forests does not impair the predictive performance of random forests and that the performance is quite robust with regard to the specified *nsplits* value. The recently developed interaction forests are the first diversity forest method that uses a complex split procedure. Interaction forests allow modeling and detecting interactions between features effectively. Further potential complex split procedures are discussed as an outlook.

**Supplementary Information:**

The online version contains supplementary material available at 10.1007/s42979-021-00920-1.

## Introduction

Random forests [4] are one of the strongest and most well-known prediction methods for categorical and continuous outcomes. There are also closely related variants for various other types of outcomes, for example, survival [16] and ordinal [14] outcomes. Demonstrating their strong practical relevance, random forests have been used frequently for predicting various outcomes in the context of the current COVID-19 pandemic [32]. Particular examples in this area include [8], who used random forests to predict the effect of social distancing and [17], who used a combination of random forests with the AdaBoost algorithm [11] to predict patient outcome. In the eponymous paper on random forests by Breiman [4], they are defined as ensembles of tree prediction rules, where each of these trees depends on a random vector $\Theta$. These random vectors are sampled anew for each tree, but from the same distribution. This definition is quite general and not confined to a single, specific procedure, as the nature and generation of the random vector $\Theta$ is not further specified. Today, the term “random forests” is commonly used for a specific procedure; see below for details. Breiman [4] showed that the more accurate the predictions of the individual trees and the smaller the correlations between these predictions, the greater the predictive performance of random forests becomes. Given that the predictive information contained in the training data set is limited, it is not possible that the individual trees perform very well and, at the same time, deliver predictions that are very disparate. Instead, any randomization procedure will be associated with some kind of tradeoff between the quality and the disparity of the tree predictions.

Breiman [4] proposed a randomization procedure that improved on the previously existing bagged ensembles of trees by strongly increasing the disparity of the tree predictions while accepting a small drop in the quality of these predictions. This increased disparity of the tree predictions is attained by randomizing the choices of the features considered for each split in the trees as opposed to considering all features in each split. Unlike in the original paper on random forests [4], today the term “random forests” is (almost) exclusively used to describe the forest algorithm associated with this specific randomization procedure. In this procedure, the trees are constructed in the following way: (1) draw a bootstrap sample or subsample from the training data set; (2) construct a tree using the sample drawn in step (1), where each split in the tree is determined as follows: (a) draw a random subset of size *mtry* from all features; (b) determine that split of the current node in the ordered values of the *mtry* features drawn in step (a) that is best with respect to a pre-specified split criterion. The elements of this procedure that are associated with the random vector $\Theta$ are the indices of the training data observations sampled in step (1) and the indices of the features sampled for each split. The term “conventional random forests” will denote this procedure in the following, where “random forest” will be abbreviated as “RF”.

As seen from the description above, in the split selection of conventional RFs each possible split in the *mtry* randomly sampled features is considered. In this paper the procedure of extending the randomization procedure in the split selection by not only randomly sampling from the features, but also, in addition, randomly sampling from the splits in the features will be investigated. The latter idea was, in its most basic form, first considered in extremely randomized trees [12]. With extremely randomized trees, the split selection is performed in the following way: first, sample *mtry* features as in the case of conventional RFs. Second, randomly draw only one split in each of the *mtry* features sampled in the first step. Third, use the split from the *mtry* splits drawn in the second step that is associated with the best value of the split criterion. The term candidate split set will in the following be used for the set of splits considered as candidates for a specific split and the members of the candidate split set will be referred to as candidate splits. For example, in the case of conventional RFs, the candidate split set associated with a split is the collection of all possible splits in the *mtry* sampled features, where each of these splits is thus a candidate split.

A major advantage of randomizing the split selection in the considered features that has, to my knowledge, not been considered so far is that it makes innovative complex split procedures tangible. Examples of more complex split procedures than the univariable, binary splitting used in conventional RFs are multivariable splitting and multi-way splitting. A multivariable split procedure uses (binary) splits that involve a number of $K > 1$ or fewer features and in a multi-way split procedure the nodes are split into (potentially) more than two child nodes. Splits in complex split procedures involve one or more features. In the following, the collection of all possible splits of a node that involve a specific set of one or several features will be denoted a split problem. The structures of split problems depend on the specific split procedures considered. In the case of classical, univariable, binary splitting a split problem would be all binary splits in feature $x_5$. A split problem in the case of a multivariable split procedure with binary splitting could be all (multivariable) binary splits that involve one, two or all three of the features $x_1$, $x_7$, and $x_{123}$. When randomizing the split selection, not all splits contained in a split problem have to be tried out, but instead a single split or a smaller number of splits can be randomly sampled from the split problem. The following can be performed repeatedly: first, sample one of the split problems randomly and, second, sample one or a few splits randomly from the split problem drawn in the first step. In the following, RFs that randomize the split selection in randomly selected features in this form and may feature complex split procedures (e.g., multivariable splitting described above) will be denoted diversity forests, hereafter referred to as DFs. As will be discussed in this paper, the DF algorithm both reduces the computational burden associated with complex split procedures and avoids overfitting resulting from trying out all splits in large split problems. There exist works on decision tree approaches for the complex split procedures multivariable splitting (see, e.g., [1, 5, 6, 22, 29] and references therein) and multi-way splitting [2, 10, 33] mentioned above. None of these approaches seem to have become established for constructing trees in RFs. These approaches use specialized (estimation) procedures to find suitable splits. In contrast, the DF split sampling scheme does not require specialized split finding algorithms for different complex split procedures. Instead, this simple split finding procedure can be applied to split forms of any complexity. Moreover, the procedure is independent of the considered outcome type and can thus be applied, for example, to categorical, continuous or survival outcomes. New complex split procedures realizable with the DF algorithm should focus less on improving the predictive performance of RFs, and more on tackling practically important issues, such as interaction detection or measuring feature importance for multi-class outcomes. The persistent popularity of conventional RFs suggests that it is difficult to improve on the predictive performance of this algorithm. Nevertheless, oblique random forests [21] and in particular, the recently introduced heterogeneous oblique random forests [19], both of which use multivariable splitting, showed promising results. As can be seen from the above descriptions, the DF algorithm is not to be used to improve on existing random forest variants that use complex split procedures, but to make new practically useful split procedures computationally possible while avoiding overfitting.

When using univariable, binary splitting as is also done in conventional RFs, DFs are similar to RFs with extremely randomized trees. The single difference is that with DFs, it is possible that non-fixed numbers of splits in the same feature are contained in the candidate split sets, whereas with RFs with extremely randomized trees, one split (or a fixed number of splits, see “Empirical Comparison Study Using Univariable, Binary Splitting”) is considered per randomly sampled feature. The reason why non-fixed numbers of splits in the same feature can be contained in the candidate split sets for DFs, is that members of the same split problem can be featured in the candidate split sets, because the split problems are sampled with replacement. Calhoun et al. [7] presented RFs with acceptance–rejection trees (RFARs), where trees are constructed in a similar manner to the trees in DFs. As with DFs using univariable, binary splitting, Calhoun et al. [7] repeatedly sample single splits from randomly sampled features, where the latter are drawn with replacement. The difference between the two split selection procedures is that, in contrast to the trees in DFs, the number of candidate splits is not fixed in acceptance–rejection trees. Instead, the split selection procedure is ended as soon as a split is found that delivers a statistically significant association between the values of the outcome and those of the binary variable that indicates, which of the observations in the current node belong to the two resulting child nodes (see “Discussion” for a discussion of the properties of this proceeding). Note that, even though, in the case of univariable, binary splitting, DFs, conventional RFs, RFs with extremely randomized trees, and RFARs all consider the same collections of possible splits, the trees obtained using these different approaches will differ, because the split selection is performed differently.

The main purposes of this paper are to introduce DFs, to compare extensively the predictive performance associated with random split selection as performed by DFs to that associated with conventional split selection, and to study how sensitive the results are to the number of candidate splits used. In these analyses the basic form of DFs that uses univariable, binary splitting will be used. However, a recently developed DF method that uses an innovative complex split procedure, interaction forests [15], will be presented as well. Interaction forests allow to model and detect interaction effects between features effectively. More DF methods with other types of complex split procedures will be considered in future work.

The rest of the paper is structured as follows. In “Description of Diversity Forests” the DF algorithm is described in its general form. “Heuristic Discussion of Advantages of the Split Selection Procedure of DFs over that of Conventional RFs for Complex Split Procedures” provides heuristic discussions of the advantages of the DF split selection procedure in comparison to that of conventional RFs. The extensive empirical comparison study of DFs with other RF-based approaches using univariable, binary splitting is presented in “Empirical Comparison Study Using Univariable, Binary Splitting”. In this section, first, the large collection of data sets with binary outcomes used in the analysis is introduced. Second, a preliminary study using a subset of these data sets is described; this study focused on investigating the sensitivity of DFs to tuning parameter value selection and determining suitable parameter value grids for optimizing these parameters. Third, the design and the results of the large-scale comparison study are detailed. In “Examples of Complex Split Procedures” the DF method interaction forests and further potential complex split procedures are treated as an outlook. The discussion (“Discussion”) briefly recalls important findings of the analyses performed in the paper and discusses various additional issues. Finally, “Conclusions” summarizes the main conclusions from the paper.

## Description of Diversity Forests

As conventional RFs, DFs are large collections of decision trees, where each of them is constructed using a random subsample or bootstrap sample from the training data. IFs differ from conventional RFs in the way the splits are selected during the construction of the trees.

As described above, with conventional RFs, the candidate split sets consist of all splits in the sampled split problems, where in the case of univariable, binary splitting, a split problem contains all splits in a specific feature. Thus, the candidate split sets considered with this procedure are specifically structured subsets of the set $allsplits_{node}$ of all splits in all features associated with the current node. With DFs differently structured subsets of $allsplits_{node}$ are considered. A candidate split set considered with DFs consists of (small) subsets of the splits in the sampled split problems.

Algorithm 1 shows a sketch of the split selection procedure performed in the construction of the trees in DFs. Details follow below.
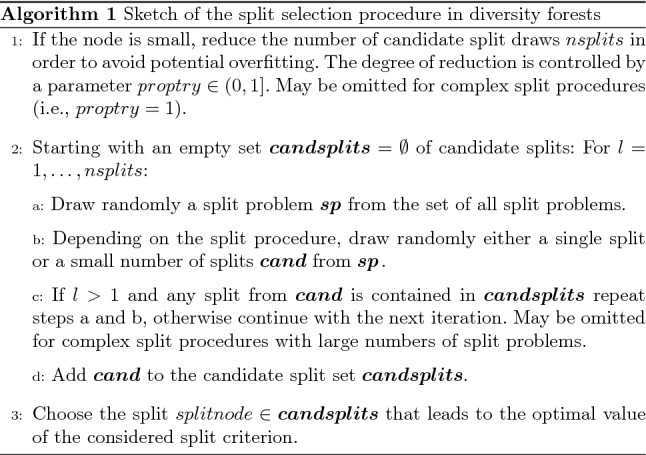


While the number of candidate split draws is generally set to a fixed number *nsplits*, where the latter is a tuning parameter, choosing smaller numbers of candidate splits for small nodes may prevent overfitting. For small nodes the pre-specified number of candidate split draws *nsplits* might be too large, because, depending on the split procedure, for small nodes the set $allsplits_{node}$ of all possible splits can also be small. If the latter is the case, *nsplits* candidate split draws may constitute an overly large proportion of $allsplits_{node}$. For this reason, when using *nsplits* candidate split draws in the case of small nodes, the best splits out of these candidate splits are likely to divide the node very well or even perfectly. Such splits may, however, not be that distinct when applied to new test observations for the purpose of prediction. Instead, the lower layers of the trees may be overly well adjusted to the training data, performing suboptimally when used in prediction. To inhibit such an overfitting resulting from sampling too many candidate splits, a parameter *proptry* is introduced that allows limitation of the maximum number of candidate splits for small nodes. If the pre-specified *nsplits* value is larger than $proptry \times \#\{allsplits_{node}\}$, where $\#$ indicates the cardinality, *nsplits* is reduced to $\lfloor proptry \times \#\{allsplits_{node}\}\rfloor$. The parameter *proptry* is larger than zero with a maximum value of one, where the latter value corresponds to always selecting the pre-specified number of candidate splits, irrespective of node size.

As discussed above the structures of the split problems depend on the type of the split procedure considered. Moreover, the split problems may also have unequal selection probabilities in step 2 a. For complex split procedures it may be difficult or costly to determine the number of all possible splits $\#\{allsplits_{node}\}$, which is necessary for the reduction of *nsplits* using *proptry* in step 1. In such situations, a *proptry* value of one may be used, in which case there would be no restriction of the numbers of candidate splits for small nodes (that is, step 1 above would be omitted). This is justifiable by the real data analysis shown in “Pre-study: Determination of Suitable Grids for the Tuning Parameter Values”, which revealed that the results are not sensitive to the choice of *proptry* and that a value of one is often suitable. The selection probabilities of the splits in the sets $\varvec{cand}$ may not be independent (for an example, see “Interaction Detection Through Bivariable Splitting in Interaction Forests)”.

The basic form of DFs that uses univariable, binary splitting and the recently developed method interaction forests are implemented in the R package diversityForest, which is a fork of the popular R package ranger [30] that uses a fast C++ implementation for most of the calculations. In future work, further diversity forest methods will be implemented in diversityForest as well. The package is available on CRAN (https://cran.r-project.org/web/packages/diversityForest/index.html) and github (https://github.com/RomanHornung/diversityForest).

## Heuristic Discussion of Advantages of the Split Selection Procedure of DFs Over that of Conventional RFs for Complex Split Procedures

The split selection procedure of DFs has two advantages over that of conventional RFs when considering complex split procedures.

*Smaller candidate split sets* Because only one or a few candidate splits are sampled from each split problem the candidate split sets are generally much smaller than in the case of conventional RFs. For complex split procedures the split problems contain many splits and with the split selection procedure of conventional RFs these would all be added to the candidate split sets. Evaluating the resulting candidate split sets would be computationally challenging or even impossible. For example, suppose that in a *K*-variable split procedure all combinations of split points between up to *K* features would be considered. This would make the corresponding split problems exceedingly large, in particular for larger sample sizes.

*More informative splits in the candidate split sets* The second advantage of the split selection procedure of DFs is that the candidate split sets tend to feature more informative splits than in the case of conventional RFs. The first reason for this is that, in the case of DFs, the sampled candidate splits have more diverse qualities than in the case of conventional RFs. This is because with DFs the different candidate splits in the candidate split sets mostly stem from different split problems and the qualities of the splits tend to differ more strongly across different split problems than within a certain split problem. This in turn is due to the fact that the features differ (strongly) in terms of the predictive information contained, making it more important which features are considered for splitting than which specific splits in a certain split problem involving one or several features are used. The second reason, why the candidate splits sets likely contain more informative splits for DFs is that they can be expected to feature more split problems than in the case of conventional RFs. This is because trying out all splits in a larger number of split problems, as would be done for conventional RFs, would not be beneficial, because it would lead to overfitting: An extensive search for good splits using the training data set makes it likely that the selected split delivers child nodes that are very homogenous with respect to the outcome. However, unfortunately, it is also likely that due to this extensive search in the training data, the child nodes resulting from this split will exhibit a higher level of homogeneity than would sets of new, independent observations associated with these child nodes. This is due to the fact that observed measurements are subject to random fluctuation not related to the predictive information contained in the features. This overfitting issue would likely be relevant for particularly complex split procedures, because for these the split problems are very large and more heterogeneous. Thus, it would likely only be possible to consider (very) few split problems with conventional RFs without causing a deteriorated predictive performance resulting from overfitting.

## Empirical Comparison Study Using Univariable, Binary Splitting

In this section, after introducing the collection of data sets considered in this paper, the designs and the results of the preliminary study and the large-scale comparison study are detailed.

### Data

As data material 220 publicly available data sets with binary outcomes were used. These data sets were also used in the study by Couronné et al. [9], who compared RFs with default tuning parameter values with logistic regression. In their study, the mean cross-validated value of the area under the ROC curve (AUC) obtained with RFs with default tuning parameter values was 0.041 points higher than that obtained with logistic regression. From the 243 data sets used by Couronné et al. [9], all data sets with more than 10,000 observations were excluded ($n=19$) to limit the computational burden and from the remaining 224 data sets all data sets with more than 500 features were excluded ($n=4$). All data sets are publicly available from the OpenML database [27]; for details on the acquisition of this collection of data sets, see [9]. Tables S1–S5 in Online Resource 1 provide information on each of the data sets. In the following, all tables and figures labeled using the prefix “S” are found in Online Resource 1.

The analysis was performed using the statistical software environment R (versions 3.5.0 and 3.6.0). All R code written to perform and evaluate the analyses as well as the pre-processed versions of the data sets used in the analyses are made available in Online Resource 2.

### Pre-study: Determination of Suitable Grids for the Tuning Parameter Values

As seen in “Description of Diversity Forests”, DFs feature two tuning parameters: the number of candidate split draws *nsplits* considered for each split and the proportion *proptry* of candidate splits to sample from all possible splits in the cases of small nodes.

A common way of choosing tuning parameter values in RFs is to try out several different values for these parameters from a pre-specified grid and choose the values that lead to the smallest out-of-bag (OOB) prediction error [3, 18]. The grid of tuning parameter values should not be specified overly dense, because the more parameter values have to be tried out, the more computationally costly the optimization will be. However, the grid should still be dense enough to ensure that the differences in prediction error associated with neighboring grid values can be expected to be small for the great majority of data sets.

In this subsection an analysis of the sensitivity of the performance of DFs to the choices of the values of the two parameters *nsplits* and *proptry* will be presented. Using this analysis a two-dimensional grid for *nsplits* and *proptry* was determined, which was used for tuning in the comparison study in “Large Scale Comparison Study of DFs Against Conventional RFs and RFs with Extremely Randomized Trees”. To determine this grid, first, for a collection of 50 data sets the prediction error of DFs using univariable, binary splitting was measured for each pair of tuning parameter values *nsplits* and *proptry* from a dense two-dimensional grid. Subsequently, the estimated prediction errors obtained for the different tuning parameter values and data sets were inspected to coarsen the two-dimensional grid to obtain a grid sufficiently dense for practical use. Note, however, that this grid is likely unsuitable for more complex split procedures than univariable, binary splitting. The main purpose of this analysis was to study the sensitivity of the performance of DFs to the choice of *nsplits* and *proptry*.

The 50 data sets used in this pre-study were randomly sampled from the 220 data sets described in “Data”. The two-dimensional grid considered for *nsplits* and *proptry* involved each pairwise combination from the grids $\{2, 5, 10, 30, 50, 100, 200\}$ and $\{0.01, 0.05, 0.1, 0.3, 0.5, 0.8, 1\}$, where the former was used for *nsplits* and the latter for *proptry*. To estimate the prediction error in each case fivefold stratified cross-validation repeated two times was used. For each DF 2000 trees were grown.

Figures S1, S2, and S3 show the cross-validated AUC values for each data set and for each parameter value pair from the two-dimensional grid. It can be seen easily from these plots that very small values of *proptry* are detrimental for many data sets, but beneficial for some data sets. However, the plots look quite different for different data sets and, given the multitude of data sets and parameter values, it is difficult to draw reliable conclusions from these plots. For this reason, we will first study the influences of *nsplits* and *proptry* separately before commenting on potential interactions between the values of these parameters. Figures S4, S5, and S6 show the influences of *nsplits* on the cross-validated AUC values for each data set. More precisely, for each *nsplits* value considered, the plots show the maximum cross-validated AUC value obtained over the seven different values of *proptry*. For some data sets (e.g., for ‘448’ and ‘788’), the prediction results are notably worse for very small values of *nsplits*. Apart from this observation, *nsplits* does not seem to have any notable influence on the predictive performance. Moreover, for none of the 50 data sets does the predictive performance change notably for *nsplits* values larger than 30. Figures S7, S8, and S9 show the influences of *proptry* on the cross-validated AUC values in an analogous way as in the cases of Figures S4, S5, and S6. For most of the data sets the prediction results are worse for small values of *proptry*, where this performance drop for small *proptry* values is often negligible; for a considerably large number of data sets, however, it is strong or very strong (e.g., for ‘334’, ‘448’, and ‘1455’ the cross-validated AUC is about 0.5 for $proptry = 0.01$, but close to 1 for large *proptry* values). The performance is frequently particularly bad for $proptry = 0.01$, that is, the smallest of the *proptry* values. Nevertheless, there are some data sets, for which the prediction results were slightly better for small *proptry* values (e.g., for ‘43’ and ‘1464’). In almost no cases does the predictive performance change notably for *proptry* values between 0.3 and 1 and, in those cases in which there is a slight difference, larger values of *proptry* delivered better results (e.g., for ‘334’ and ‘788’).

Above the influences of *nsplits* and *proptry* were studied separately. The first important finding in this analysis was that, while very small values of *nsplits* can be detrimental, the predictive performance does not seem to change notably for *nsplits* values larger than or equal to 30. This finding would suggest recommending use of the fixed value 30 for *nsplits*. The second important finding was that large values of *proptry* lead to (much) better results for the majority of data sets, but for some data sets small *proptry* are preferable. Moreover, often $proptry = 0.01$ lead to much worse results than $proptry = 0.05$. Given that the results hardly differed when varying the *proptry* value between 0.3 and 1 with a slight tendency for better performance for larger values of *proptry*, these results would suggest recommending the grid $\{0.05, 1\}$ for *proptry*. Using $nsplits = 30$ and choosing *proptry* from $\{0.05, 1\}$ would, however, only be reasonable, if there are no interactions between these parameters with respect to their effect on the performance that would lead to different combinations of *nsplits* and *proptry* being optimal for a relevant proportion of data sets. To study whether or not the latter is the case, we reexamine Figures S1, S2, and S3: For most data sets, the plots either do not suggest any relevant interaction effects or the differences in performance are very small across all parameter values considered. However, for some data sets (e.g., for ‘53’ and ‘479’) we observe the following pattern: The larger the value of *nsplits*, the more the predictive performance depends negatively on *proptry*; as a consequence, bad predictive performances occurring in the case of large *nsplits* values are prevented when the *proptry* values are small at the same time. This negative dependency of the predictive performance on the *proptry* value for large *nsplits* values can be interpreted as follows: for these data sets, larger values of *nsplits* lead to overfitting in the cases of smaller nodes. This overfitting is prevented, if the *proptry* values are small at the same time, because small *proptry* values will have the effect that fewer candidate split values are considered for smaller nodes. For such data sets, for which there is a danger of a relevantly strong overfitting of small nodes by considering too many candidate splits, this overfitting should be prevented by fixing $nsplits = 30$ and choosing *proptry* from the grid $\{0.05, 1\}$. In this situation the optimization algorithm will choose the *proptry* value 0.05, provided that this *proptry* value will be associated with a smaller OOB prediction error than the *proptry* value one.

The results described above show that, regardless of whether the values of *nsplits* and *proptry* interact with respect to their effect on the predictive performance, fixing $nsplits = 30$ and choosing the *proptry* value from the grid $\{0.05, 1\}$ is appropriate. Therefore, the latter proceeding is recommended and will be followed in the analyses presented later in this paper. The main results of the analysis presented in this subsection are presented in Table 1.

**Table 1:** Overview of results of pre-study on the sensitivity of DFs to the choices of *nsplits* and *proptry*

Parameter	Meaning	Range	Conclusions from pre-study
*nsplits*	Number of sampled split problems / splits	$[1, \infty )$	Low impact on results; fixed value ($nsplits = 30$) sufficient
*proptry*	Controls the degree of reduction of *nsplits* for small nodes	(0, 1]	Low impact on results; select from $\{0.05, 1\}$ (using OOB error)

In general, the above analysis revealed that, provided that the values of *nsplits* and *proptry* are not specified very small, the influences of these parameters are only weak. The split selection procedure of DFs does not depend on the split procedure considered. For this reason, the general conclusion that the sensitivity of DFs to the choices of the values of *nsplits* and *proptry* is low, provided that these values are not chosen too small, can be assumed to also hold for more complex split procedures. However, since the above analysis considered exclusively univariable, binary splitting, for other, more complex, split procedures no detailed statements regarding the choice of suitable values for *nsplits* and *proptry* can be made.

### Large Scale Comparison Study of DFs Against Conventional RFs and RFs with Extremely Randomized Trees

The main purpose of the analysis presented in this subsection was to compare the predictive performance of DFs with that of conventional RFs and RFs with extremely randomized trees using the large collection of data sets described in “Data”. Additional goals were to analyze the influence of data set characteristics on the predictive performances of the methods and the optimized tuning parameter values, as well as the sensitivity of the performances of the methods to changes in the values of the tuning parameters. As described above, both RFs with extremely randomized trees and DFs have in common that the split selection is randomized. In their original definition [12], extremely randomized trees use only one randomly sampled split per considered feature. However, the implementation of RFs with extremely randomized forest provided with the R package ranger [30] allows consideration of larger, fixed numbers of splits per considered feature.

As seen in the analysis presented in the paper introducing RFARs [7], they perform similarly well to RFs with extremely randomized trees: Using ten data sets with binary outcomes, Calhoun et al. [7] compared the classification performance of RFARs with that of conventional RFs, RFs with extremely randomized trees and RFs with so-called smooth sigmoid surrogate trees [26]; RFARs, RFs with extremely randomized trees and RFs with smooth sigmoid surrogate trees each performed best for three of the data sets and, for one data set, RFARs and RFs with extremely randomized trees both performed best. Calhoun et al. [7] used the OOB AUC to compare the predictive performances of the methods. For all four methods, I calculated the medians of these values across the ten data sets and found that these median values were very similar between the methods. In the current paper, RFARs were not included in the comparison study, because its goal was not to demonstrate superiority of DFs using conventional univariable, binary splitting over specific other approaches. Instead, its main goal was to compare the predictive performance resulting from random split selection with that resulting from conventional split selection. If random split selection would be (strongly) inferior to conventional split selection, it would not be meaningful to apply the concept of random split selection for realizing innovative complex split procedures using DFs.

#### Study Design

For DFs, *nsplits* was set to 30 and the *proptry* value associated with the smaller OOB prediction error was selected from the grid $\{0.05, 1\}$. Two variants of RFs with extremely randomized trees were considered, where, for the first variant, only one split was randomly drawn from each of the *mtry* considered features and, for the second variant, five splits were drawn. These two variants will be referred to as “RFsextr1” and “RFsextr5”, respectively. The values of *mtry* for RFs, RFsextr1, and RFsextr5 were selected from grids, where again in each case the value was selected that featured the smallest OOB prediction error. If the number of features was at most 20, the grid considered featured each possible value for *mtry*. For data sets with more than 20 features, a grid for *mtry* featuring 20 values was formed in the following way: $\{[i^{\log (p)/\log (20)}] \mid i \in \{1, \dots , 20\}\}$, where *p* denotes the number of features. The forests constructed for each parameter value from the respective grids consisted of 1000 trees and the forests constructed after optimization that featured the optimized parameter values consisted of 2000 trees. Again, as in the pre-study, fivefold stratified cross-validation repeated twice was used for predictive performance estimation.

As Couronné et al. [9] who used the same data sets in their study, the prediction accuracy (ACC), the AUC, and the Brier score were considered as performance measures, where, in consistency with Couronné et al. [9], most of the interpretations are based on the ACC without loss of generality.

#### Results

*Global performance* Table 2 shows summary estimates of the performances of the different methods across data sets according to the three considered performances measures. More precisely, the quartiles of the different performance measures calculated per data set are shown. For all three performance measures, the quartiles differ only slightly between the different methods. Nevertheless, for all three measures, RFs are outperformed by the other methods in terms of the median. The biggest differences are seen in terms of the AUC, where both RFsextr1 and RFsextr5 outperform RFs by more than 0.01 in terms of the median. RFsextr1 and RFsextr5 also outperform DFs with respect to the median AUC; however, DFs outperform both RFsextr1 and RFsextr5 in terms of the first quartile of the AUC values and perform almost identical in terms of the third quartile of these values.

**Table 2:** Performances of the different methods summarized across the 220 data sets

Measure	Method	$Q_1$ (25% quantile)	Median	$Q_3$ (75% quantile)
ACC	RF	0.7681	0.8802	0.9568
	DF	0.7673	0.8823	0.9602
	RFsextr1	0.7761	0.8810	0.9598
	RFsextr5	0.7704	0.8821	0.9600
AUC	RF	0.7637	0.9070	0.9849
	DF	0.7794	0.9106	0.9878
	RFsextr1	0.7704	0.9207	0.9869
	RFsextr5	0.7737	0.9202	0.9881
Brier	RF	0.0384	0.0910	0.1657
	DF	0.0355	0.0896	0.1680
	RFsextr1	0.0360	0.0891	0.1648
	RFsextr5	0.0353	0.0881	0.1654

Method-specific quartiles of the different performance measures calculated per data set

Wilcoxon tests were used to test, whether the slight improvements of DFs, RFsextr1, and RFsextr5 over RFs with respect to the three measures are statistically significant. In the case of DFs the 50 data sets that had been used in the pre-study for determining suitable grids for the tuning parameter values were excluded. This choice was made to avoid potential overoptimism resulting from having used part of the collection of data sets in setting up the algorithm. DFs performed significantly better ($\alpha$ = 0.05) than RFs with respect to the AUC and the Brier score, but not with respect to the ACC (*p* values: ACC: 0.057, AUC: 0.002, Brier: < 0.001). Note that the same conclusions are obtained when including the 50 data sets used in in the pre-study. RFsextr1 were significantly better than RFs for all three measures (*p* values: ACC: 0.046, AUC: < 0.001, Brier: < 0.001) and for RFsextr5 the same conclusions were obtained as in the case of DFs (*p* values: ACC: 0.057, AUC: < 0.001, Brier: < 0.001). It must be stated that these *p* values might be slightly overoptimistic, because the data sets are not fully independent. As the data set labels in Tables S1–S5 reveal, several of the data sets form groups in the sense that they constitute versions of the same data set. The effect sizes of the tests were all in the small to moderate range (DF vs. RF: ACC: *r* = 0.15, AUC: *r* = 0.24, Brier: *r* = 0.35; RFsextr1 vs. RF: ACC: *r* = 0.14, AUC: *r* = 0.34, Brier: *r* = 0.43; RFsextr5 vs. RF: ACC: *r* = 0.14, AUC: *r* = 0.27, Brier: *r* = 0.40).

**Fig. 1:**
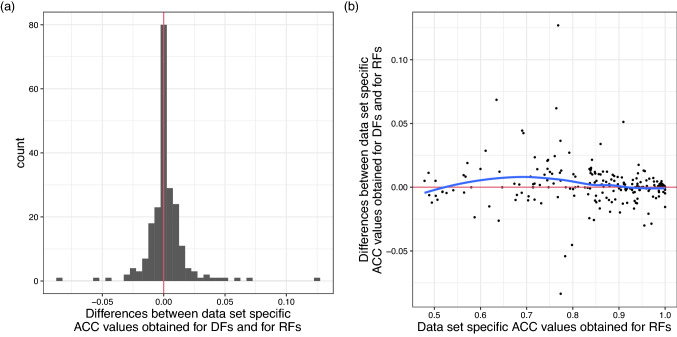
Data set specific performances of DFs compared to that of RFs. **a** Histogram of the differences between the data set specific ACC values obtained for DFs and for RFs. The red line indicates the zero line. **b** Scatter plot of the differences between the data set specific ACC values obtained for DFs and for RFs against the data set specific ACC values obtained for RFs. The blue curved line represents a LOESS fit. The red horizontal line again indicates the zero line

*Performance differences obtained for the individual data sets* The above analysis concerned the summarized performances of the methods across data sets. However, naturally the performances of the methods differ (strongly) across data sets, because each data set features a different data distribution. The remaining evaluations presented below will focus on the data set specific performances of the methods. Following Couronné et al. [9], these evaluations will be restricted to the ACC as performance measure. However, when using the other two measures, very similar conclusions are obtained (results not shown). Differences in results obtained for the different measures will be outlined in the following descriptions. Panel (a) of Fig. 1 shows that for the majority of data sets studied the performances of DFs and RFs are similar, for some data sets DFs perform notably better than RFs and for few data sets notably worse. In panel (b) of Fig. 1 it can be seen that DFs tend to outperform RFs primarily in situations in which RFs perform medium well, whereas, in cases in which RFs perform well to very well, on average there does not seem to be a benefit from using DFs. In the cases of RFsextr1 and RFsextr5 (Figure S10) the differences in performance, compared to that of RFs, are again small for the majority of data sets. However, particularly for RFsextr1, but also for RFsextr5, there are more data sets for which there are notably strong differences in performance compared to RFs than in the case of DFs. The latter higher variability in results might be explained by the fact that, for RFsextr1 and RFsextr5, larger numbers of different candidate tuning parameter values are considered in the optimization than in the case of DFs. The reason why the variability in performance compared to RFs is slightly larger for RFsextr1 than for RFsextr5 could be that the optimization of *mtry* can be expected to be more stable for RFsextr5 than for RFsextr1, because for the former, more candidate splits are drawn per considered feature. Like DFs, RFsextr1 and RFsextr5 also tend to outperform RFs in cases in which RFs perform medium well.

**Fig. 2:**
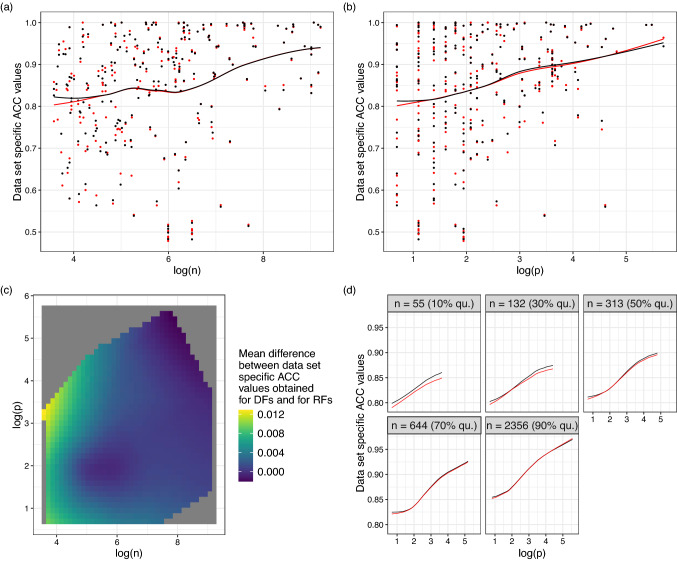
Influence of sample size *n* and number of features *p* on the performance of DFs and RFs. **a** and **b** Data set specific ACC values obtained for DFs and RFs plotted against the logarithmized values of *n* and *p*. The lines show LOESS fits obtained for DFs and RFs, respectively. **c** Two-dimensional LOESS fit of the influences of the logarithmized values of *n* and *p* on the differences between the data set specific ACC values obtained for DFs and for RFs. **d** Cross sections of two-dimensional LOESS fits of the influences of the logarithmized values of *n* and *p* on the data set specific ACC values obtained for DFs and RFs, respectively. The cross sections were taken at different quantiles of the sample sizes of all data sets. Where applicable, in each plot the black (dark) lines show the results obtained for DFs and the red (light) lines those obtained for RFs

*Influence of sample size and numbers of features on the performance differences* As seen at the beginning of this subsection, overall, the differences in performance between the methods are small. However, it was also seen that there are data sets for which there is a notably strong difference in performance between DFs, RFsextr1, and RFsextr5 on the one hand and RFs on the other. This suggests that there are specific circumstances, where larger differences in performance between the methods can be expected. For example, the available sample size *n* may affect the methods differently so that, depending on the sample size, some methods may be preferable over others. Another factor that might be of relevance is the number of features *p* available for a data set. In Fig. 2, the influences of sample size and number of features on the performance of DFs and RFs are illustrated. As expected, both *n* and *p* have a positive influence on the data set specific ACC values (panels (a) and (b) of Fig. 2). Given that we did not observe notably strong differences between RFs and DFs above, it is not surprising that, for most sample sizes and numbers of features, the differences in performance between the two methods are not notable. However, panels (a) and (b) of Fig. 2 do reveal that for very small *n* and *p* DFs perform slightly better than RFs. Panels (c) and (d) of the figure do not reveal any noteworthy interaction between the effect of the sample size and that of the number of features on performance differences between RFs and DFs.

The influence of *n* and *p* on the performance of RFsextr1 and RFsextr5 in comparison to that of RFs is investigated in Section D of Online Resource 1.

*Influence of data set characteristics on the selected tuning parameter values* While *nsplits* was set to a fixed value in the analyses, the value of *proptry* was selected from 0.05 and 1 in each cross-validation iteration. Moreover, for RFs, RFsextr1, and RFsextr5 the parameter *mtry* was selected from a larger grid of values.

It is interesting to study, how different data set characteristics influence the selected tuning parameter values. A study of the influence of sample size, number of features and strength of signal in the features on the selected tuning parameter values of the different methods was performed. For reasons of brevity, the in-depth analysis and discussion of this study is described in Section E of Online Resource 1. In the following, the main findings from this study will be discussed. It was seen that *proptry* values of 0.05 tend to be selected more frequently for data sets with comparably weak signal. This is likely due to the fact that data sets with weaker signal are more susceptible to overfitting. By choosing a *proptry* value of 0.05, less candidate splits are sampled for smaller nodes and the trees are not grown to full size, because tree growing stops as soon as 0.05 times the number of all possible splits is smaller than one. Both of these effects help avoiding overfitting small nodes.

As expected, the larger the number of features *p*, the larger the value of the optimal *mtry* value tended to be for RFs, RFsextr1, and RFsextr5. However, this relationship was not strong. The optimal *mtry* values were the largest for RFsextr1, smaller for RFsextr5 and the smallest for RFs. It was also seen that the common rule of thumb of setting $mtry = \sqrt{p}$ for RFs delivers too small *mtry* values for larger values of *p*. This is congruent with the findings of Probst et al. [24] who used a collection of 38 data sets to determine better default values for *mtry* in RFs. Probst et al. [24] found that using the rule $mtry = 0.432 p$ delivers better results. While it was seen in Section E.2 of Online Resource 1 that this rule may tend to deliver too small *mtry* values for small *p* and too large *mtry* values for large *p*, it was clearly superior to the standard rule $mtry = \sqrt{p}$. The fact that the optimal *mtry* values are larger for larger *p* does not mean that it is necessary to choose large *mtry* values—or large *nsplits* values in the case of DFs—for data sets with large *p* to achieve a close to optimal performance: Although the optimal *mtry* values were larger for larger *p*, the OOB errors were close to the minimal OOB errors even when specifying the *mtry* values much smaller (Online Resource 3). Typically, the OOB errors were considerably larger only for very small *mtry* values, took similar values beyond that and, for some data sets and only in the case of RFs, increased again considerably for *mtry* values larger than the optimal *mtry* values. For RFsextr1 and RFsextr5 interestingly the OOB errors got quickly small and similar beyond very small *mtry* values, but, as opposed to in the case of RFs, did not get notably larger for larger *mtry* values. This is congruent with the finding from “Pre-study: Determination of Suitable Grids for the Tuning Parameter Values” that the predictive performance of DFs is weak for very small *nsplits* values, but consistently strong for larger values. The sample size only had a weak, non-linear effect on the optimal *mtry* values. The strength of signal in the features had a very weak effect on the optimal *mtry* values, which differed slightly between RFs, RFsextr1, and RFsextr5. For more details and further heuristic discussions the interested reader is referred to Section E2 of Online Resource 1. Overall the influence of the data set characteristics on the optimal *mtry* values was weak, where a clear influence was only seen for the number of features.

## Examples of Complex Split Procedures

Conventionally, RFs are constructed using univariable, binary splitting. More complex split procedures do not seem to have received much attention in the literature. A natural reason for the latter is likely that, as discussed in “Heuristic Discussion of Advantages of the Split Selection Procedure of DFs over that of Conventional RFs for Complex Split Procedures”, with the split selection procedure of conventional RFs many complex split procedures would be computationally very demanding or simply intractable. Moreover, as also explained in “Heuristic Discussion of Advantages of the Split Selection Procedure of DFs over that of Conventional RFs for Complex Split Procedures”, even if a complex split procedure with split problems that involve many splits would be computationally tractable, the split selection procedure of RFs would still not be suitable, because trying out all members of complex split problems can be expected to lead to overfitting.

A major advantage of randomly selecting splits as performed by the DF algorithm is that this, in contrast to the split selection procedure of conventional RFs, does enable use of such complex split procedures. In this section, I will first present the recently developed interaction forests. Subsequently, as an outlook I will discuss multi-way splitting and further complex split procedures.

### Interaction Detection Through Bivariable Splitting in Interaction Forests

The interaction forest algorithm is the first and currently the only published DF method that uses complex splitting. In the literature it is often stated that conventional RFs would be particularly effective for taking interaction effects between features into account (see, e.g., the literature references given in [31]). However, as discussed in [15, 31], since the splitting—and consequently also the split selection—is performed univariably in conventional RFs, they focus on strong univariable effects without modeling interaction effects effectively. There exists quite a variety of approaches that aim at identifying interaction effects from tree ensembles; for a literature overview see [15]. However, most of these use classical, univariable, binary splitting. This likely explains why they typically perform poorly at differentiating truly interacting feature pairs from feature pairs for which both pair members only feature strong marginal effects, but no interacting effects.

**Fig. 3:**
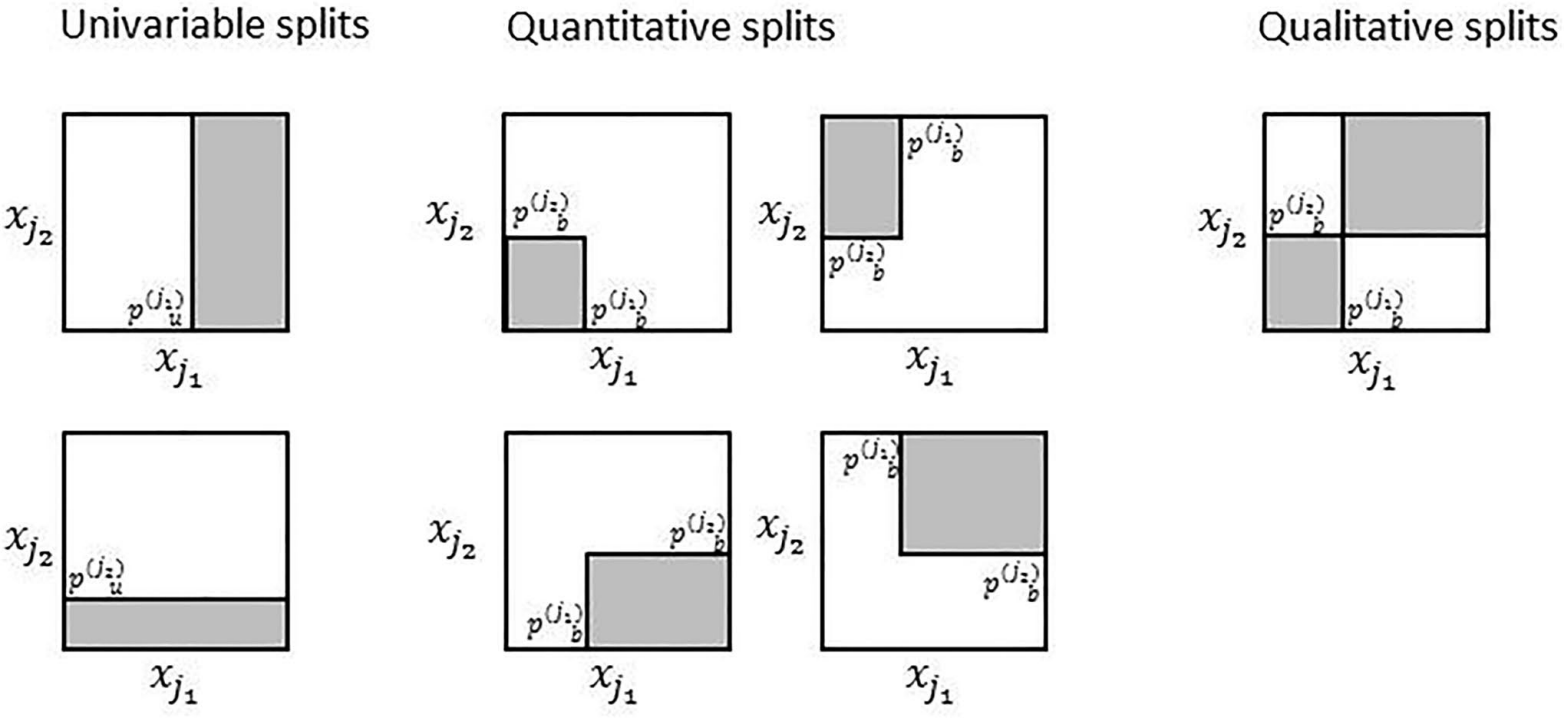
Split types considered in the interaction forest algorithm. Each square visualizes the feature space spanned by two features $x_{j_1}$ and $x_{j_2}$. The points $p_u^{(j_1)}$, $p_u^{(j_2)}$, and $(p_b^{(j_1)}, p_b^{(j_2)})$ denote the split points for univariable and bivariable splits, respectively. The white and gray areas depict the regions associated with the two child nodes of the splits. Figure adapted from [15]

In interactions forests we model interaction effects directly using bivariable splitting. Here we differentiate between quantitative and qualitative interaction effects [23]. A quantitative interaction means that the strength of the effect of feature *A* on the outcome depends on feature *B*, but the direction of that effect does not change in dependency of the value of *B*. In contrast, with a qualitative interaction not only the strength but also the direction of the effect of feature *A* depends on feature *B*, such that *A* both has a positive and negative effect. A split problem in interaction effect contains all possible univariable and bivariable splits in one feature pair that are of the types visualized in Fig. 3. Trying out all possible splits of these types in a pair of features would be computationally very demanding, especially because many trees (default: 20,000) have to be constructed to identify interaction effects reliably. Using the diversity forest algorithm, however, only one split of each type is sampled, that is, seven splits per split problem. Note that the selection probabilities of the quantitative and qualitative splits (Fig. 3) among these seven splits are not independent, because they all share the same split points $(p^{(j_1)}_b, p^{(j_2)}_b)$.

Interaction detection with interaction forests is performed using its effect importance measure (EIM). The latter uses a procedure by Hapfelmeier et al. [13] to measure the importance of each of the split types shown in Fig. 3 for each feature and feature pair. This allows to rank the feature pairs according to the importance of their quantitative and qualitative interaction effects for prediction in addition to ranking the features according to the importance of their univariable effects. The split types are defined in such a way in interaction forests that they target well interpretable interaction effects that are easy to communicate. For higher dimensional data the number of possible feature pairs becomes too large, making in impossible to consider all feature pairs. Therefore, for data sets with more than 100 features we pre-select 5000 feature pairs that show the strongest indications of interaction effects according to a screening procedure. As stated before, interaction forests are implemented in the diversityForest R package. Here, we also provide plot functions for visualizing the estimated bivariable influence of feature pairs with large EIM values. The latter is crucial for drawing conclusions on the exact forms of the interaction effects, because these cannot be deterred from the EIM values. Simulation results [15] suggest that the EIM performs superior to other tree-based ensemble methods for identifying interaction effects. Moreover, in an extensive real data analysis using the 220 data sets that were also used in the current paper interaction forests tended to deliver better prediction results than conventional RFs and competing RF-based methods that use multivariable splitting. For more details, see [15].

### Outlook: Multi-way Splitting

In the case of classification problems with more than two classes of the outcome, multi-way split procedures are likely more appropriate. If the outcome has *K* classes, a *K*-way split procedure could be applied, that is, the nodes be split into *K* child nodes as opposed to only two. This would allow to obtain *K* child nodes for which each of them primarily contains observations from one of the *K* classes, which is not possible using conventional binary splitting. Using a multi-way split procedure can also improve on the (permutation) feature importance measure [4] of RFs for multi-class outcomes. This is because a multi-way split procedure puts more emphasis on selecting split features that differentiate well between all *K* classes instead of only a subset of the classes.

Multi-way split procedures are, however, likely also beneficial for two-class classification problems. Multi-way splitting increases the flexibility by which the univariable effects of the features are taken into account. When performing conventional binary splitting, the algorithm searches for single splits in the values of the features that divide the observations in the node into two child nodes that are as homogenous as possible with respect to the outcome. However, the splits in the features found in this way may often be suboptimal, because there might be features that have a stronger, but more complex influence. Such influences are more likely to be detected using multi-way splits. An example of a more complex influence of a feature on a binary outcome would be a tub-shaped influence, where the probability for class 1 is high for small values of the feature, low for moderate values and large again for large values. Another example would be very large probabilities for class 1 for small feature values, probabilities around 0.5 for moderate values, and very small probabilities for large values. When using such a feature in binary splitting, at least one of the resulting two child nodes will be impure. However, when using a three-way split, we would be able to obtain two (almost) pure child nodes and one impure node.

Considering multi-way splits instead of binary splits can not only be expected to deliver better predictive predictive performance of the trees, but their predictions will likely also be more diverse. This is because the variety of features considered in the trees is greater, as complex feature influences like the ones described above are exploited in addition to simpler influences that can be taken into account by binary splits.

Given the recursive nature of the trees, the advantage of multi-way splitting to allow for a more sophisticated rendering of the influences of the features is particularly important with respect to the first few splits in the trees. The first splits have the strongest influence on the structures of the trees. For this reason, using a highly informative multi-way split at the root of the tree can be expected to deliver a better performing tree than using a simple, binary first split; such a simple split will be more likely to prematurely divide observations that are relatively similar with respect to the outcome.

Multi-way splitting may be particularly effective for large data sets because of the complexity of this procedure, which benefits from the high precision associated with training using large sample sizes. A split problem in multi-way splitting would be the following: all possible splits in a specific feature that divide the current node into (up to) *K* child nodes. The larger the specified value of *K*, the more complex the splits will be.

### Outlook: Further Potential Complex Split Procedures

Above the advantages of multi-/bivariable and multi-way splits were discussed. Apart from these specific split procedures, the split selection procedure of DFs allows consideration of split procedures of any complexity, avoiding problems resulting from overfitting and high computational cost or even infeasibility from a computational point of view. For example, combining multivariable and multi-way splitting may also be considered. It would also be possible to consider split procedures with diffuse partitions of the feature space, which may help to detect interesting predictive patterns in the data. One example of such a split procedure would be bivariable splitting with random partitions of the spaces spanned by pairs of features, where these partitions are used to assign the observations to the child nodes. A specific application area for new complex split procedures could be multi-label data. While published random forest-based approaches to dealing with multi-label data exist [20, 28], it could be of interest to develop a DF approach with a more simple tree structure for the specific purpose of measuring feature importance for multi-label data.

## Discussion

In this paper the diversity forest algorithm was introduced, an alternative split sampling scheme that allows to use innovative complex split procedures in random forests. The analyses presented in this paper revealed that random split selection as performed by diversity forests does not impair predictive performance compared to conventional split selection and that the results are quite robust with respect to the number of candidate split draws *nsplits* considered in split selection. These two properties open the door to using the diversity forest algorithm for realizing new complex split procedures in RFs. Interaction forests are the first published DF method with a complex split procedure.

In the case of complex split procedures the total numbers of possible splits can become very large. If, for such split procedures, the required number of candidate splits to consider per split would be required to be large in relation to the total number of possible splits, this would render the procedure computationally intractable. However, luckily, the required number of candidate splits is not expected to grow with the complexity of the split procedure. This is because the number of features is the same, independent of the complexity of the split procedure. For complex split procedures the probability that a sampled split involves strong features is as large as or even larger than (the latter being the case, e.g., for multivariable splitting) for univariable, binary splitting. Given that the quality of a split depends primarily on the feature or features used in it, it becomes clear that the density of strong splits among the sampled candidate splits is fairly independent of the complexity of the split procedure. Therefore, even though the numbers of all possible splits are much larger for complex split criteria, the optimal numbers of candidate splits should be in the same order of magnitude as that associated with univariable, binary splitting. Moreover, we saw in “Pre-study: Determination of Suitable Grids for the Tuning Parameter Values” and “Results” that, independent of the number of available features, for random split selection, beyond very small numbers of candidate splits, the predictive performance is only weakly influenced by the number of candidate splits. For these reasons, independently of the number of features or the degree of complexity of the split procedure, it should not be necessary to consider large numbers of candidate splits when randomizing the split selection, making forest construction well feasible from a computational point of view. For example, in interaction forests we sample a maximum number of ten split problems per split, which, given that we sample seven splits per split problem, leads to a maximum of 70 considered candidate splits.

The analyses presented in this paper were restricted to the case of binary outcomes. It has been observed previously that *mtry* in RFs should be chosen larger in the regression case than in the classification case [4]. Similarly, in DFs using a larger *nsplits* value in the case of metric outcomes may be preferable. However, we also saw that the performance of DFs is quite robust with regard to the choice of *nsplits*, which is why the performance is likely not affected notably when choosing the same *nsplits* value for different outcome types. Correspondingly, the popular ranger R package [30] uses the same default value $mtry = \sqrt{p}$ for all considered outcome types.

The intention of using the parameter *proptry* that limits the number of candidate splits for small nodes was to inhibit potential overfitting of small nodes resulting from considering too many candidate splits. For complex split criteria the total numbers of candidate splits can be expected to be large even for small nodes, which is why for specific complex split criteria it will not be necessary to limit the numbers of candidate splits for small nodes, but instead it will be enough to simply use a *proptry* value of one.

When using univariable, binary splitting, randomizing the split selection leads to only little improvement in predictive performance. Nevertheless, for big data applications it can be expected to also be advantageous for univariable, binary splitting. This is because, for very large data sets it is computationally demanding to try out all possible splits in the features. Moreover, as seen in [7], randomizing the split selection avoids problems caused by the tendency of the trees in conventional RFs to select features with many possible splits [25].

As described before, RFARs [7] do not use fixed numbers of candidate splits, but instead they continue split sampling until a split is found that delivers a statistically significant association between child node membership and the values of the outcome. Using a stop criterion in candidate split sampling would also be possible in DFs. An advantage of this would be that, by letting the number of required candidate splits be determined automatically, we would avoid situations in which the default number of candidate split draws *nsplits* is not suitable. However, such situations can be expected to be rare given the robustness of the results with respect to the choice of *nsplits*. Moreover, it is not clear whether using a stopping procedure in split selection is beneficial in general. Such a procedure has the effect that all splits in the trees are of similar quality. However, it is not clear, why this property would be beneficial; in contrast, trees that deliver splits of varying qualities are likely more diverse, which is associated with better predictive performance of RFs [4].

If a stopping rule is used, it should, however, not be based on significance testing of the form used by RFARs. This is because the stopping rule used in RFARs tends to consider more candidate splits for smaller nodes and, as explained in “Description of Diversity Forests”, selecting too many candidate splits for small nodes can lead to overfitting. RFARs tend to select larger numbers of candidate splits for smaller nodes, because the *p* values of the tests used to decide whether the split selection procedure of RFAR is stopped are naturally larger for smaller nodes. Therefore, the probability of a *p* value falling below the significance threshold is smaller for smaller nodes. The fact that the number of candidate splits depends on the node size in RFARs is suboptimal, not only with respect to small nodes. By considering fewer candidate splits for large nodes the strong predictive information contained in these nodes is less well exploited. For these reasons it might be effective to replace the significance testing in RFARs by a criterion that does not depend on node size. A suitable criterion for binary outcomes might be Cramér’s V, a measure for the degree of association between two binary features. In this case, instead of using a significance threshold, we would need a threshold in the values of Cramér’s V. Unlike the number of candidate split draws *nsplits* in DFs, the values of Cramér’s V can be directly interpreted in terms of the degree of the decisiveness of the splits.

Comparing the results obtained by [7] with those obtained in this paper, it can be seen that the performance differences between RFsextr1 and RFs were larger in this paper than they were in [7]. One important reason for this discrepancy is likely that the signals in the data sets tended to be larger in [7] than in the collection of data sets considered in this paper; for example, the median AUC value obtained with conventional RFs was 0.9621 in [7], while it was 0.9070 in this paper (see Table 2). Another reason for the smaller performance differences seen in [7] is likely that they did not optimize the values of *mtry* in each cross-validation iteration, but used default values for *mtry*. Finally, the results obtained in this paper are based on a much larger collection of data sets than that of [7]. The large number of data sets considered in this paper made it possible to obtain reliable conclusions and perform more sophisticated analyses, such as studying the influence of number of features and sample size on the performances of the methods.

## Conclusions

Randomizing the split selection in RFs via the DF algorithm enables integrating practically useful split procedures of any complexity and does not impair predictive performance in comparison to conventional split selection. Moreover, the performance of DFs is quite robust with respect to the number of candidate split draws *nsplits* used. The DF split finding algorithm is easy to understand and works in the same way for different outcome types, such as categorical, continuous, and survival outcomes. DFs may not feature a stronger predictive power compared to conventional RFs. However, the main focus of complex split procedures realized using the DF algorithm should not be predictive performance, but solving practically important problems, such as interaction detection or measuring feature importance for multi-class outcomes. RF-based methods lend themselves to solving such problems because of their capability to capture complex dependency patterns between the outcome and the features.

The DF algorithm is intended for methodologically oriented researchers with a focus on applications. I hope that this work will inspire other researchers to develop DF variants that solve practically important problems. The recently introduced DF method, interaction forests, shows promising results and illustrates the practicability of DFs. One future project will focus on a DF with a multi-way split procedure, because as described in “Outlook: Multi-way Splitting”, this allows for the use of a feature importance measure for multi-class outcomes that identifies features which can differentiate well between all the outcome classes instead of only a subset. Another planned project will focus on developing a statistical testing procedure for the effect importance measure values of interaction forests that will enable testing which feature pairs display statistically significant interaction effects.

## Supplementary Information

Below is the link to the electronic supplementary material.

Supplementary file1 (PDF 676 KB)

Supplementary file1 (ZIP 108032 KB)

Supplementary file1 (PDF 466 KB)

## Data Availability

The pre-processed versions of all data sets are available in Online Resource 2. Note that all data sets are also publicly available in the OpenML database.

## Code Availability

All R code written for the analyses presented in the paper and supplement are available in Online Resource 2.
