# Supplementary material for: Diversity Forests: Using Split Sampling to Enable Innovative Complex Split Procedures in Random Forests
Source: SN Comput Sci. 2021 Oct 21;3(1):1. doi: 10.1007/s42979-021-00920-1 (PMC8533673; doi:10.1007/s42979-021-00920-1)
Supplement: Supplementary file 1 — Supplementary file1 (PDF 676 KB) [file 42979_2021_920_MOESM1_ESM.pdf]

Online Resource 1 to the article "Diversity Forests: Using Split Sampling to Enable Innovative Complex Split Procedures in Random Forests" by Roman Hornung published in the journal SN Computer Science

Various additional contents referred to in the article

Roman Hornung*,[1]

[1] Institute for Medical Information Processing, Biometry and Epidemiology, University of Munich, Marchioninistr. 15, 81377 Munich, Germany

*Contact: hornung@ibe.med.uni-muenchen.de

A Overview of the Data Sets used in the Analyses

Table S1: Overview of data sets – I. The following information is provided: 'data.id': OpenML ID of the data set, 'label': data set label, 'n': sample size, 'p': number of features, 'prop. categ.': proportion of categorial features, 'prop. min. class': proportion of observations in the smaller class of the target variable.

data.id	label	n	p	prop. categ.	prop. min. class
31	credit-g	1000	20	0.650	0.300
37	diabetes	768	8	0.000	0.349
40	sonar	208	60	0.000	0.466
43	haberman	306	3	0.333	0.265
44	spambase	4601	57	0.000	0.394
50	tic-tac-toe	958	9	1.000	0.347
53	heart-statlog	270	13	0.000	0.444
59	ionosphere	351	34	0.000	0.359
164	molecular-biology_promoters	106	57	1.000	0.500
292	Australian	690	14	0.000	0.445
311	oil_spill	937	49	0.000	0.044
312	scene	2407	299	0.017	0.179
316	yeast_ml8	2417	116	0.112	0.014
333	monks-problems-1	556	6	1.000	0.500
334	monks-problems-2	601	6	1.000	0.343
335	monks-problems-3	554	6	1.000	0.480
336	SPECT	267	22	1.000	0.206
337	SPECTF	349	44	0.000	0.272
346	aids	50	4	0.500	0.500
444	analcatdata_boxing2	132	3	1.000	0.462
446	prnn_crabs	200	7	0.143	0.500
448	analcatdata_boxing1	120	3	1.000	0.350
450	analcatdata_lawsuit	264	4	0.250	0.072
459	analcatdata_asbestos	83	3	0.667	0.446
467	analcatdata_japansolvent	52	8	0.000	0.481
472	lupus	87	3	0.000	0.402
476	analcatdata_bankruptcy	50	5	0.000	0.500
479	analcatdata_cyyoung9302	92	9	0.333	0.207
682	sleuth_ex2016	87	10	0.100	0.414
683	sleuth_ex2015	60	7	0.000	0.500
713	vineyard	52	3	0.000	0.462
714	fruitfly	125	4	0.500	0.392
717	rmftsa_ladata	508	10	0.000	0.437
719	veteran	137	7	0.571	0.314
720	abalone	4177	8	0.125	0.498
721	pwLinear	200	10	0.000	0.485
724	analcatdata_vineyard	468	3	0.333	0.444
725	bank8FM	8192	8	0.000	0.404
728	analcatdata_supreme	4052	7	0.000	0.240
729	visualizing_slope	44	3	0.000	0.386
731	baskball	96	4	0.000	0.490
733	machine_cpu	209	6	0.000	0.268
735	cpu_small	8192	12	0.000	0.302

Table S2: Overview of data sets – II. The following information is provided: 'data.id': OpenML ID of the data set, 'label': data set label, 'n': sample size, 'p': number of features, 'prop. categ.': proportion of categorial features, 'prop. min. class': proportion of observations in the smaller class of the target variable.

data.id	label	n	p	prop. categ.	prop. min. class
736	visualizing_environmental	111	3	0.000	0.477
737	space_ga	3107	6	0.000	0.496
741	rmftsa_sleepdata	1024	2	0.500	0.497
745	auto_price	159	15	0.067	0.340
747	servo	167	4	1.000	0.228
748	analcatdata_wildcat	163	5	0.400	0.288
750	pm10	500	7	0.000	0.492
753	wisconsin	194	32	0.000	0.464
755	sleuth_ex1605	62	5	0.000	0.500
758	analcatdata_election2000	67	14	0.000	0.269
759	analcatdata_olympic2000	66	11	0.000	0.500
761	cpu_act	8192	21	0.000	0.302
764	analcatdata_apnea3	450	3	0.667	0.122
765	analcatdata_apnea2	475	3	0.667	0.135
767	analcatdata_apnea1	475	3	0.667	0.128
770	strikes	625	6	0.000	0.496
771	analcatdata_michiganacc	108	4	0.500	0.444
772	quake	2178	3	0.000	0.445
774	disclosure_x_bias	662	3	0.000	0.479
777	sleuth_ex1714	47	7	0.000	0.426
778	bodyfat	252	14	0.000	0.492
780	rabe_265	51	6	0.000	0.412
782	rabe_266	120	2	0.000	0.475
784	newton_hema	140	3	0.333	0.500
787	witmer_census_1980	50	4	0.000	0.480
788	triazines	186	60	0.000	0.414
790	elusage	55	2	0.500	0.436
791	diabetes_numeric	43	2	0.000	0.395
795	disclosure_x_tampered	662	3	0.000	0.494
800	pyrim	74	27	0.000	0.419
801	chscase_funds	185	2	0.000	0.470
803	delta_ailerons	7129	5	0.000	0.469
804	hutsof99_logis	70	7	0.571	0.486
807	kin8nm	8192	8	0.000	0.491
811	rmftsa_ctoarrivals	264	2	0.500	0.383
814	chscase_vine2	468	2	0.000	0.453
815	chscase_vine1	52	9	0.000	0.462
816	puma8NH	8192	8	0.000	0.498
817	diggle_table_a1	48	4	0.000	0.479
818	diggle_table_a2	310	8	0.125	0.468
819	delta_elevators	9517	6	0.000	0.497
820	chatfield_4	235	12	0.000	0.396
826	sensory	576	11	1.000	0.415
827	disclosure_x_noise	662	3	0.000	0.497
835	analcatdata_vehicle	48	4	1.000	0.438

Table S3: Overview of data sets – III. The following information is provided: 'data.id': OpenML ID of the data set, 'label': data set label, 'n': sample size, 'p': number of features, 'prop. categ.': proportion of categorial features, 'prop. min. class': proportion of observations in the smaller class of the target variable.

data.id	label	n	p	prop. categ.	prop. min. class
841	stock	950	9	0.000	0.486
847	wind	6574	14	0.000	0.467
848	schlvote	38	5	0.200	0.263
851	tecator	240	124	0.000	0.425
853	boston	506	13	0.077	0.413
857	bolts	40	7	0.000	0.350
859	analcatdata_gviolence	74	8	0.000	0.419
860	vinnie	380	2	0.000	0.487
872	boston	506	13	0.154	0.413
874	rabe_131	50	5	0.000	0.420
875	analcatdata_chlamydia	100	3	1.000	0.190
880	mu284	284	10	0.000	0.500
882	pollution	60	15	0.000	0.483
885	transplant	131	3	0.000	0.366
886	no2	500	7	0.000	0.498
887	mbagrade	61	2	0.500	0.475
890	cloud	108	7	0.143	0.296
892	sleuth_case1201	50	6	0.000	0.480
893	visualizing_hamster	73	5	0.000	0.452
894	rabe_148	66	5	0.000	0.500
895	chscase_geyser1	222	2	0.000	0.396
900	chscase_census6	400	6	0.000	0.412
902	sleuth_case2002	147	6	0.667	0.469
905	chscase_adopt	39	2	0.000	0.308
906	chscase_census5	400	7	0.000	0.482
907	chscase_census4	400	7	0.000	0.485
908	chscase_census3	400	7	0.000	0.480
909	chscase_census2	400	7	0.000	0.492
914	balloon	2001	2	0.000	0.241
915	plasma_retinol	315	13	0.231	0.422
919	rabe_166	40	2	0.000	0.475
921	analcatdata_seropositive	132	3	0.333	0.348
923	visualizing_soil	8641	4	0.250	0.450
924	humandevel	130	2	0.000	0.500
925	visualizing_galaxy	323	4	0.000	0.458
927	hutsof99_child_witness	42	16	0.000	0.405
928	rabe_97	46	4	0.250	0.457
929	rabe_176	70	4	0.000	0.500
931	disclosure_z	662	3	0.000	0.474
934	socmob	1156	5	0.800	0.221
945	kidney	76	6	0.500	0.474
946	visualizing_ethanol	88	2	0.000	0.489
947	arsenic-male-bladder	559	3	0.000	0.043
949	arsenic-female-bladder	559	3	0.000	0.143
950	arsenic-female-lung	559	3	0.000	0.034

Table S4: Overview of data sets – IV. The following information is provided: 'data.id': OpenML ID of the data set, 'label': data set label, 'n': sample size, 'p': number of features, 'prop. categ.': proportion of categorial features, 'prop. min. class': proportion of observations in the smaller class of the target variable.

data.id	label	n	p	prop. categ.	prop. min. class
951	arsenic-male-lung	559	3	0.000	0.023
954	spectrometer	531	101	0.010	0.104
955	tae	151	5	0.400	0.344
958	segment	2310	19	0.000	0.143
962	mfeat-morphological	2000	6	0.000	0.100
964	pasture	36	22	0.045	0.333
965	zoo	101	16	0.938	0.406
969	iris	150	4	0.000	0.333
970	analcatdata_authorship	841	70	0.000	0.377
971	mfeat-fourier	2000	76	0.000	0.100
973	wine	178	13	0.000	0.399
974	hayes-roth	132	4	0.000	0.386
976	JapaneseVowels	9961	14	0.000	0.162
978	mfeat-factors	2000	216	0.000	0.100
980	optdigits	5620	64	0.000	0.102
983	cmc	1473	9	0.778	0.427
987	collins	500	22	0.091	0.160
988	fl2000	67	15	0.067	0.388
991	car	1728	6	1.000	0.300
994	vehicle	846	18	0.000	0.258
995	mfeat-zernike	2000	47	0.000	0.100
997	balance-scale	625	4	0.000	0.461
1005	glass	214	9	0.000	0.355
1009	white-clover	63	31	0.129	0.397
1011	ecoli	336	7	0.000	0.426
1013	analcatdata_challenger	138	2	0.500	0.065
1014	analcatdata_dmft	797	4	1.000	0.194
1015	confidence	72	3	0.000	0.167
1016	vowel	990	13	0.231	0.091
1020	mfeat-karhunen	2000	64	0.000	0.100
1021	page-blocks	5473	10	0.000	0.102
1022	mfeat-pixel	2000	240	1.000	0.100
1025	analcatdata_germangss	400	5	1.000	0.225
1043	ada_agnostic	4562	48	0.000	0.248
1045	kc1-top5	145	94	0.000	0.055
1048	jEdit_4.2_4.3	369	8	0.000	0.447
1049	pc4	1458	37	0.000	0.122
1050	pc3	1563	37	0.000	0.102
1054	mc2	161	39	0.000	0.323
1055	cm1_req	89	8	0.125	0.225
1056	mc1	9466	38	0.000	0.007
1059	ar1	121	29	0.000	0.074
1060	ar3	63	29	0.000	0.127
1061	ar4	107	29	0.000	0.187
1062	ar5	36	29	0.000	0.222

Table S5: Overview of data sets – V. The following information is provided: ‘data.id’: OpenML ID of the data set, ‘label’: data set label, ‘n’: sample size, ‘p’: number of features, ‘prop. categ.’: proportion of categorial features, ‘prop. min. class’: proportion of observations in the smaller class of the target variable.

data.id	label	n	p	prop. categ.	prop. min. class
1063	kc2	522	21	0.000	0.205
1064	ar6	101	29	0.000	0.149
1065	kc3	458	39	0.000	0.094
1066	kc1-binary	145	94	0.000	0.414
1067	kc1	2109	21	0.000	0.155
1068	pc1	1109	21	0.000	0.069
1069	pc2	5589	36	0.000	0.004
1071	mw1	403	37	0.000	0.077
1073	jEdit_4.0_4.2	274	8	0.000	0.489
1075	datatrieve	130	8	0.000	0.085
1121	badges2	294	11	0.273	0.286
1441	KungChi3	123	39	0.000	0.130
1442	MegaWatt1	253	37	0.000	0.107
1443	PizzaCutter1	661	37	0.000	0.079
1444	PizzaCutter3	1043	37	0.000	0.122
1446	CostaMadre1	296	37	0.000	0.128
1447	CastMetal1	327	37	0.000	0.128
1451	PieChart1	705	37	0.000	0.087
1452	PieChart2	745	36	0.000	0.021
1453	PieChart3	1077	37	0.000	0.124
1454	PieChart4	1458	37	0.000	0.122
1455	acute-inflammations	120	6	0.833	0.417
1462	banknote-authentication	1372	4	0.000	0.445
1463	blogger	100	5	1.000	0.320
1464	blood-transfusion-service-center	748	4	0.000	0.238
1467	climate-model-simulation-crashes	540	20	0.000	0.085
1473	fertility	100	9	0.000	0.120
1479	hill-valley	1212	100	0.000	0.500
1480	ilpd	583	10	0.100	0.286
1487	ozone-level-8hr	2534	72	0.000	0.063
1488	parkinsons	195	22	0.000	0.246
1489	phoneme	5404	5	0.000	0.293
1490	planning-relax	182	12	0.000	0.286
1494	qsar-biodeg	1055	41	0.000	0.337
1495	qualitative-bankruptcy	250	6	1.000	0.428
1498	sa-heart	462	9	0.111	0.346
1504	steel-plates-fault	1941	33	0.000	0.347
1510	wdbc	569	30	0.000	0.373
1511	wholesale-customers	440	8	0.125	0.323
1524	vertebra-column	310	6	0.000	0.323
1547	autoUniv-au1-1000	1000	20	0.000	0.259
1570	wilt	4839	5	0.000	0.054

B Pre-study: Cross-validated AUC Values Obtained for the Different Tuning Parameter Values and Data Sets

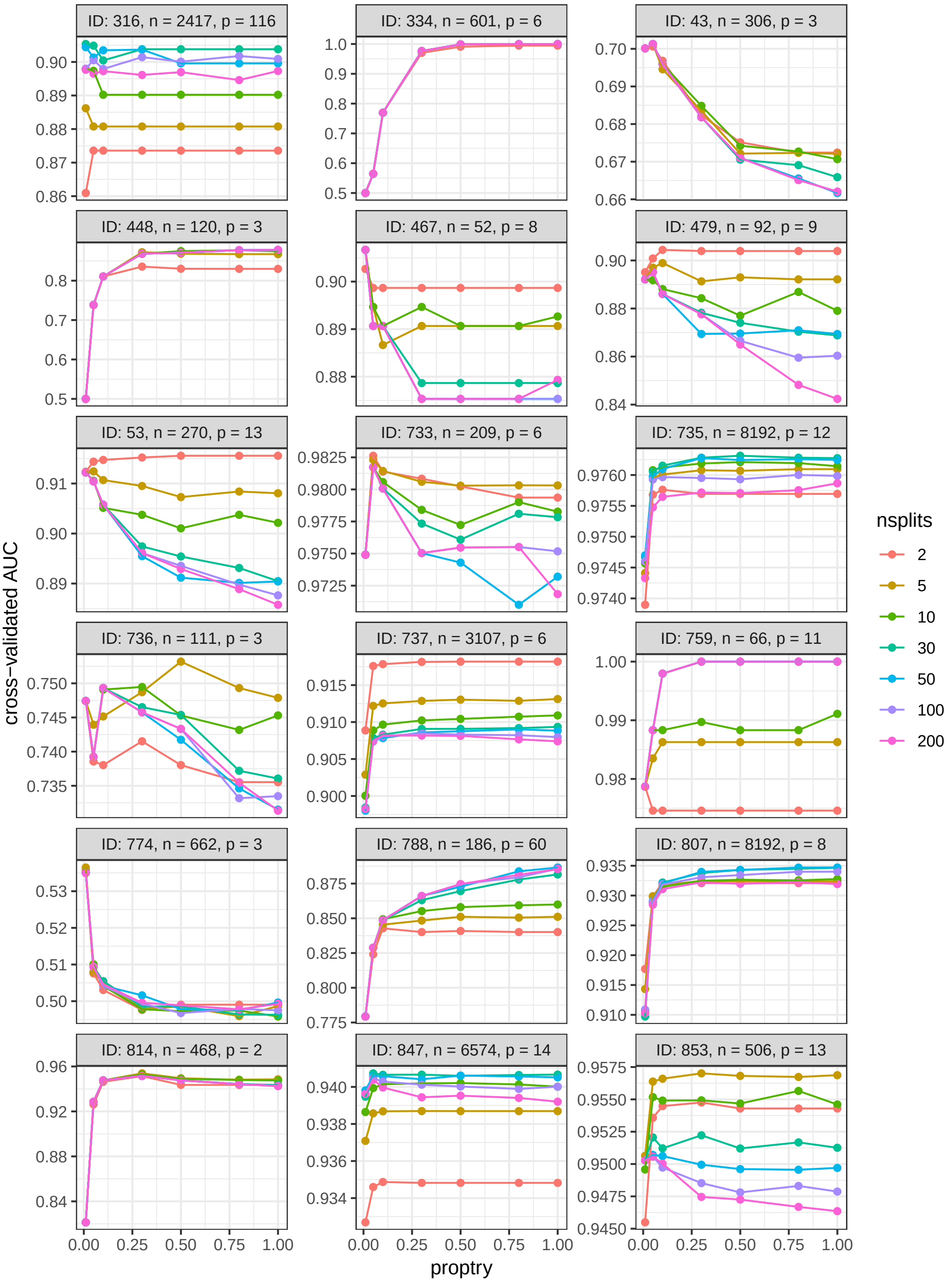

Fig. S1: Pre-study: Cross-validated AUC values obtained for the different parameter values. Each panel shows the results obtained for a particular data set. – I

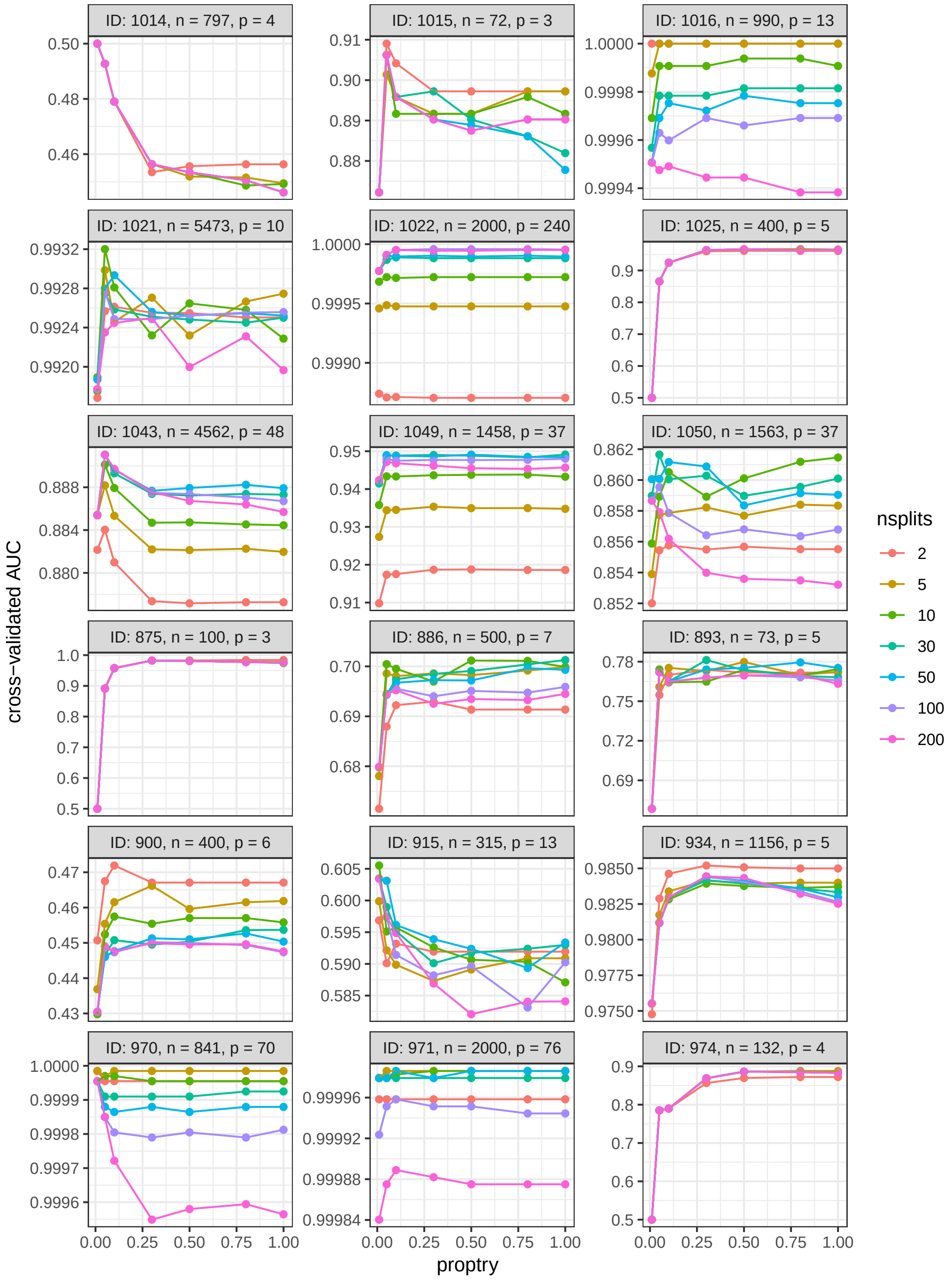

Fig. S2: Pre-study: Cross-validated AUC values obtained for the different parameter values. Each panel shows the results obtained for a particular data set. – II

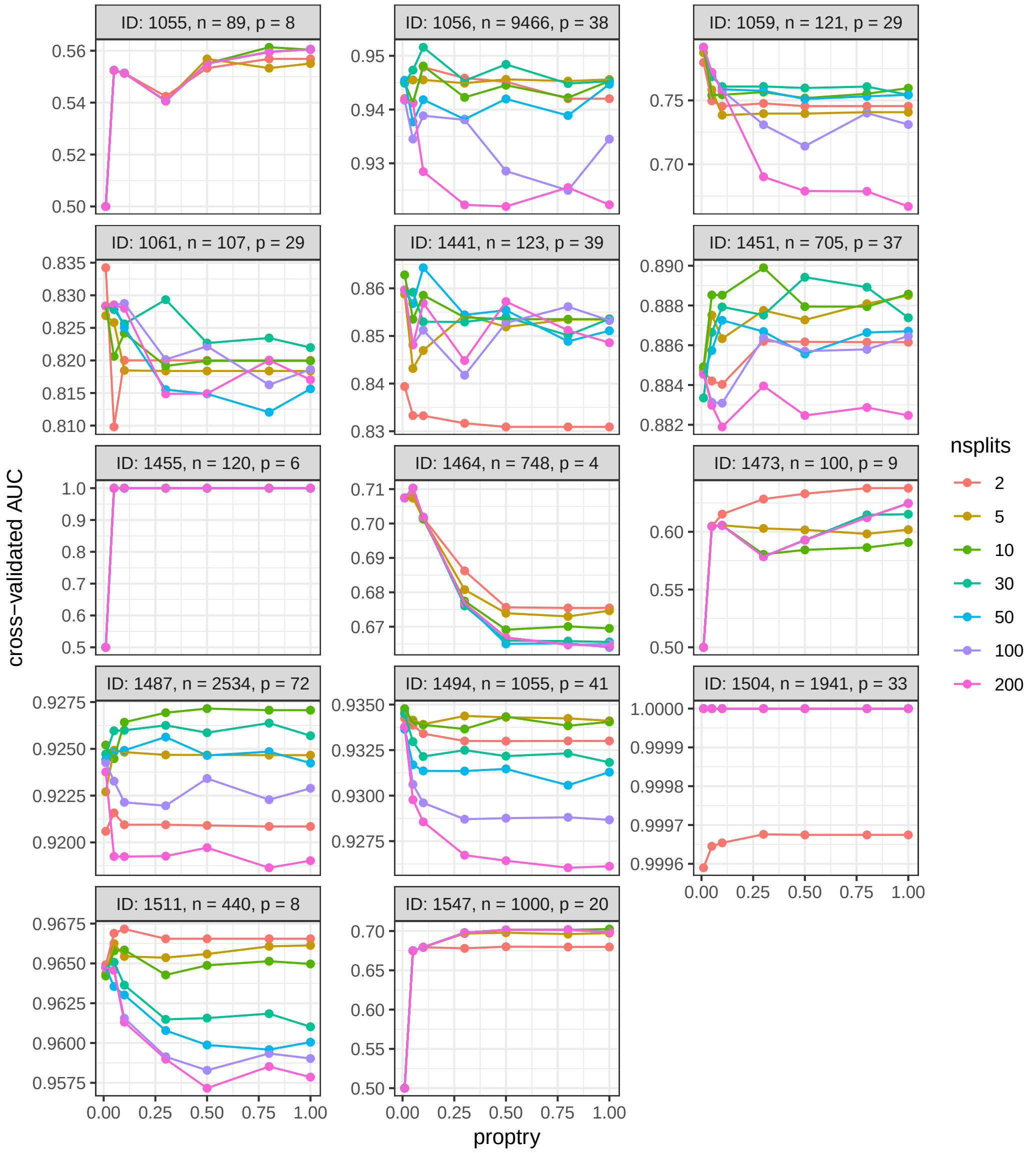

Fig. S3: Pre-study: Cross-validated AUC values obtained for different parameter values. Each panel shows the results obtained for a particular data set. – III

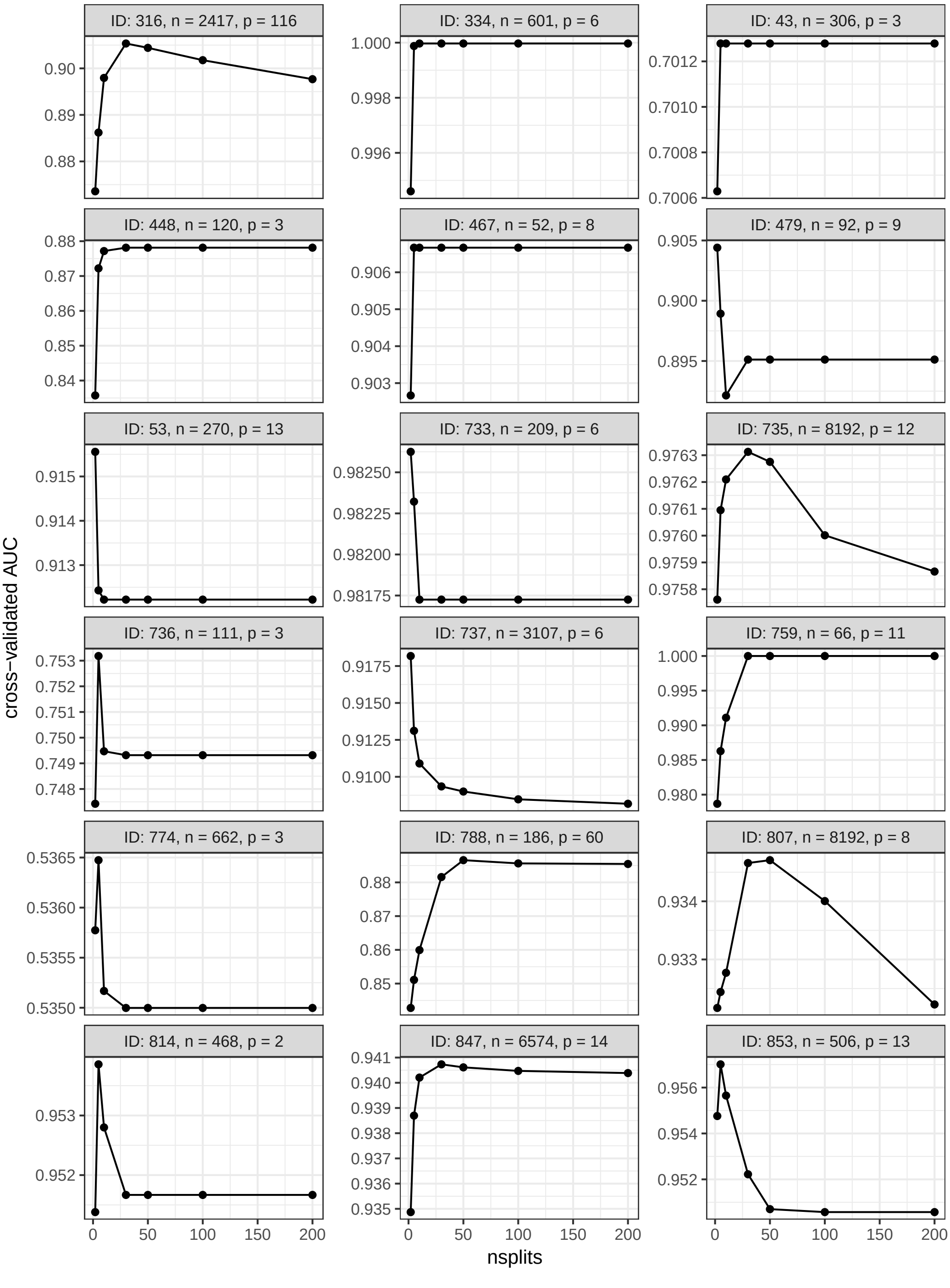

Fig. S4: Pre-study: Cross-validated AUC values obtained for different *nsplits* values. For each *nsplits* value considered, the plots show the maximum cross-validated AUC value obtained over the seven different values of *proptry*. Each panel shows the results obtained for a particular data set. – I

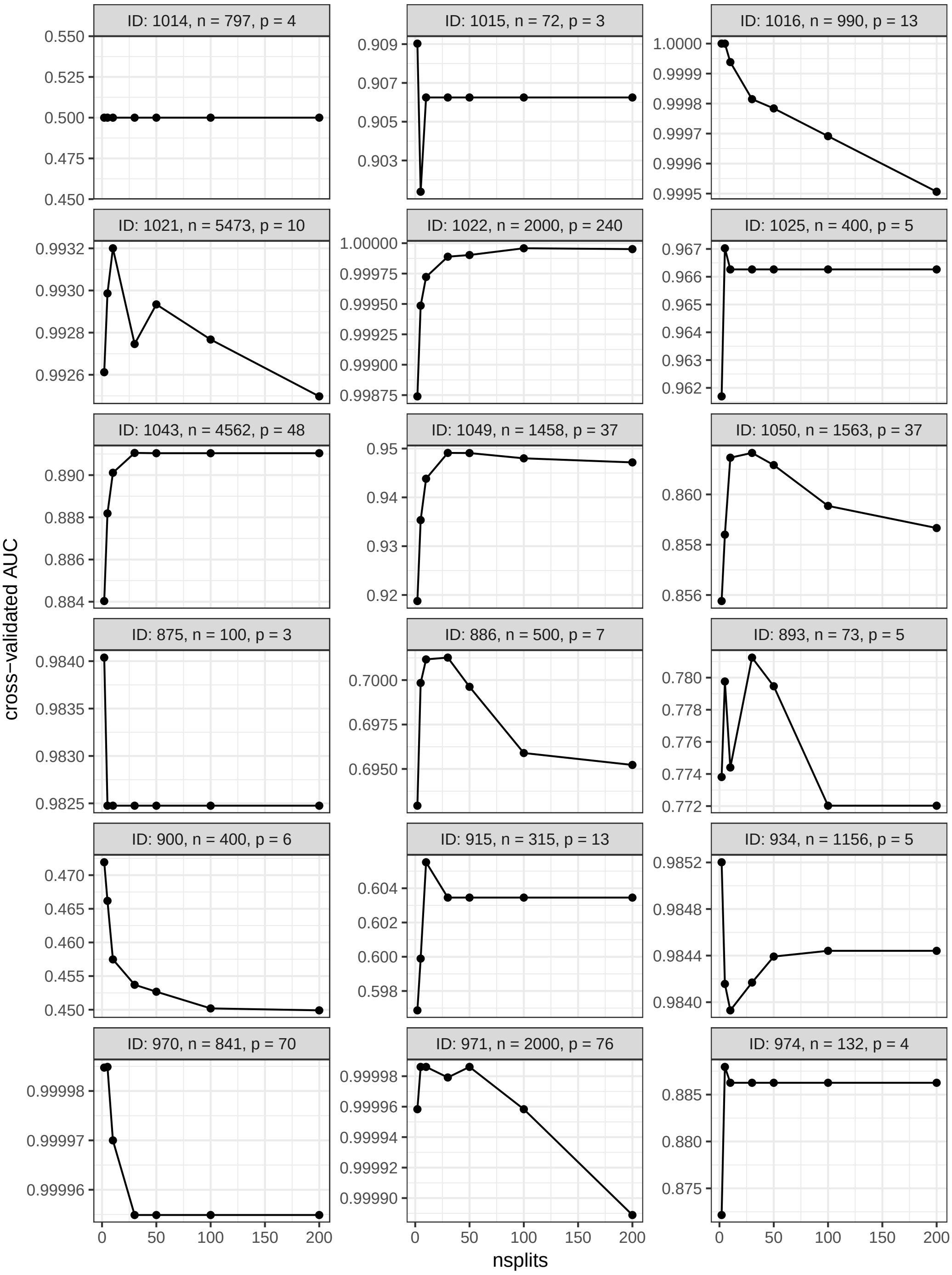

Fig. S5: Pre-study: Cross-validated AUC values obtained for different *nsplits* values. For each *nsplits* value considered, the plots show the maximum cross-validated AUC value obtained over the seven different values of *proptry*. Each panel shows the results obtained for a particular data set. – II

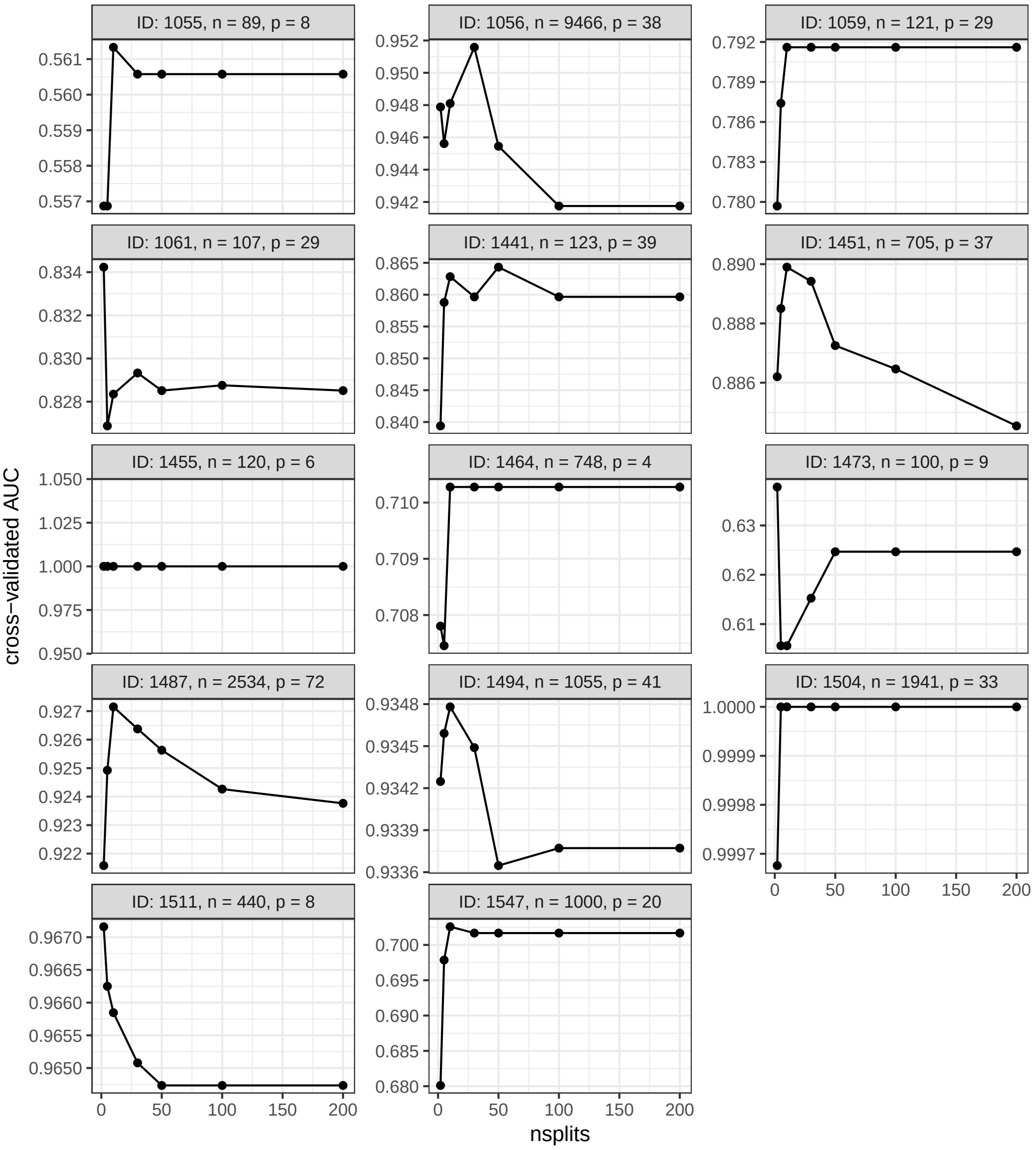

Fig. S6: Pre-study: Cross-validated AUC values obtained for different *nsplits* values. For each *nsplits* value considered, the plots show the maximum cross-validated AUC value obtained over the seven different values of *proptry*. Each panel shows the results obtained for a particular data set. – III

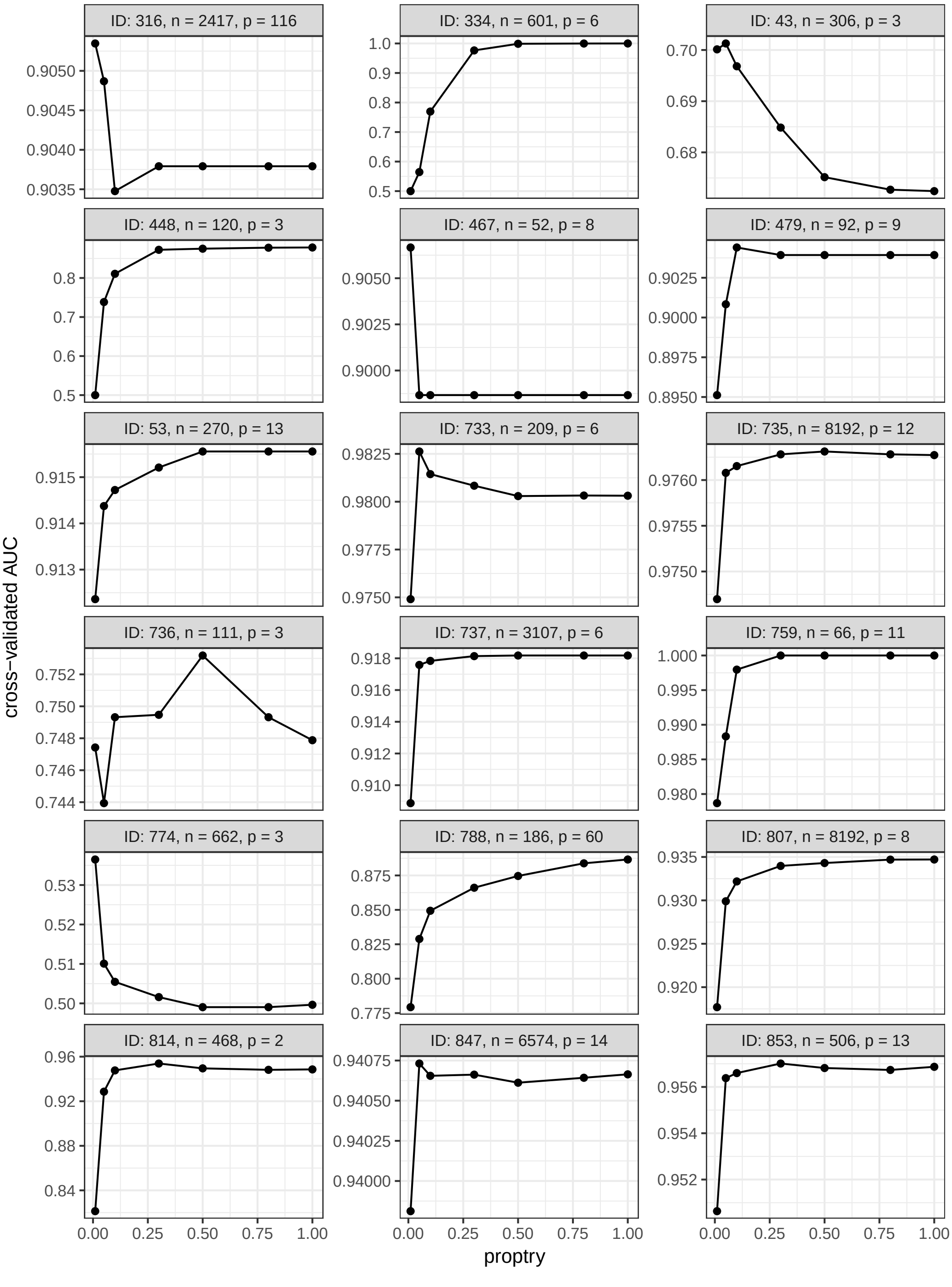

Fig. S7: Pre-study: Cross-validated AUC values obtained for different *proptry* values. For each *proptry* value considered, the plots show the maximum cross-validated AUC value obtained over the seven different values of *nsplits*. Each panel shows the results obtained for a particular data set. – I

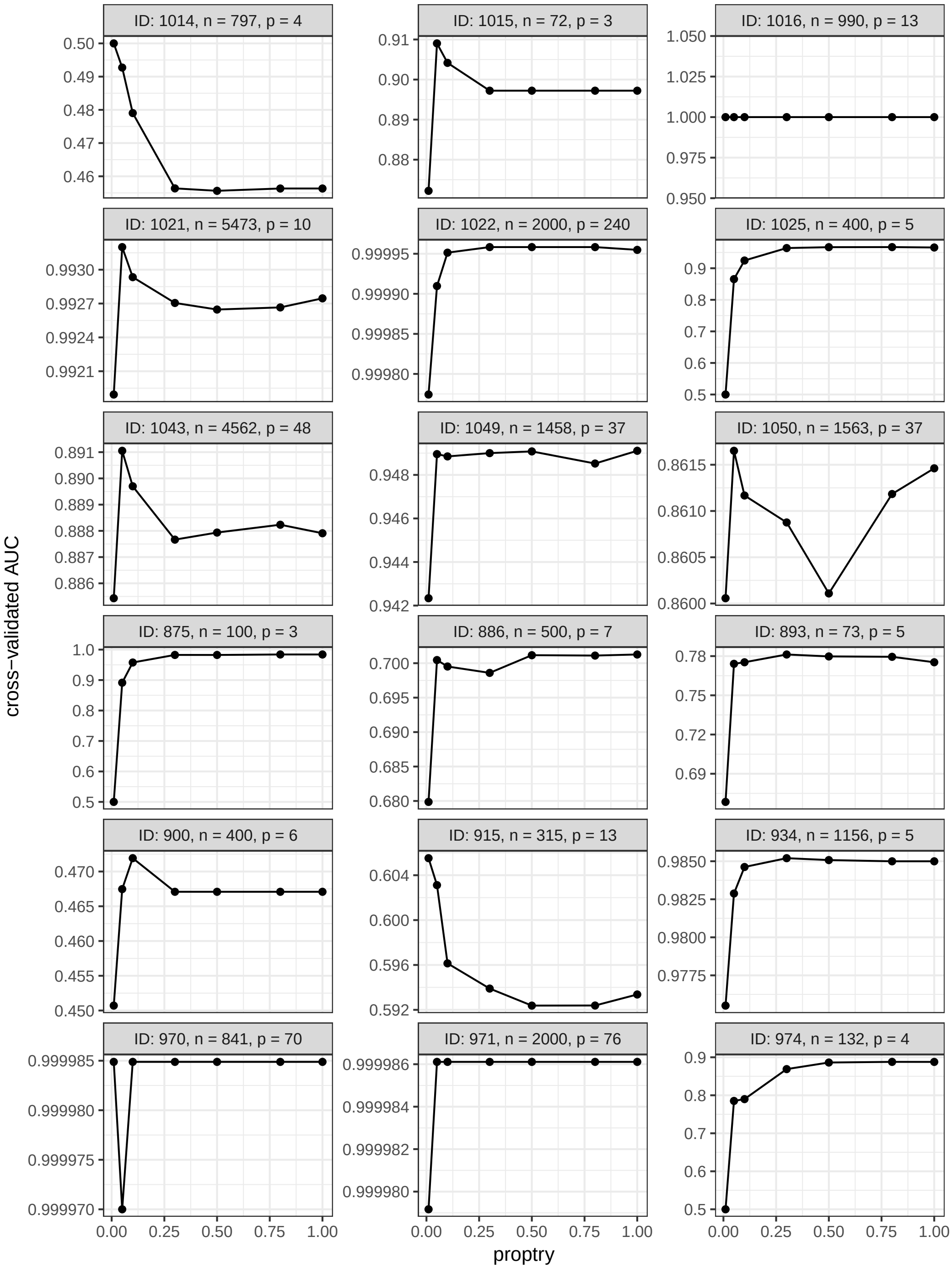

Fig. S8: Pre-study: Cross-validated AUC values obtained for different *proptry* values. For each *proptry* value considered, the plots show the maximum cross-validated AUC value obtained over the seven different values of *nsplits*. Each panel shows the results obtained for a particular data set. – II

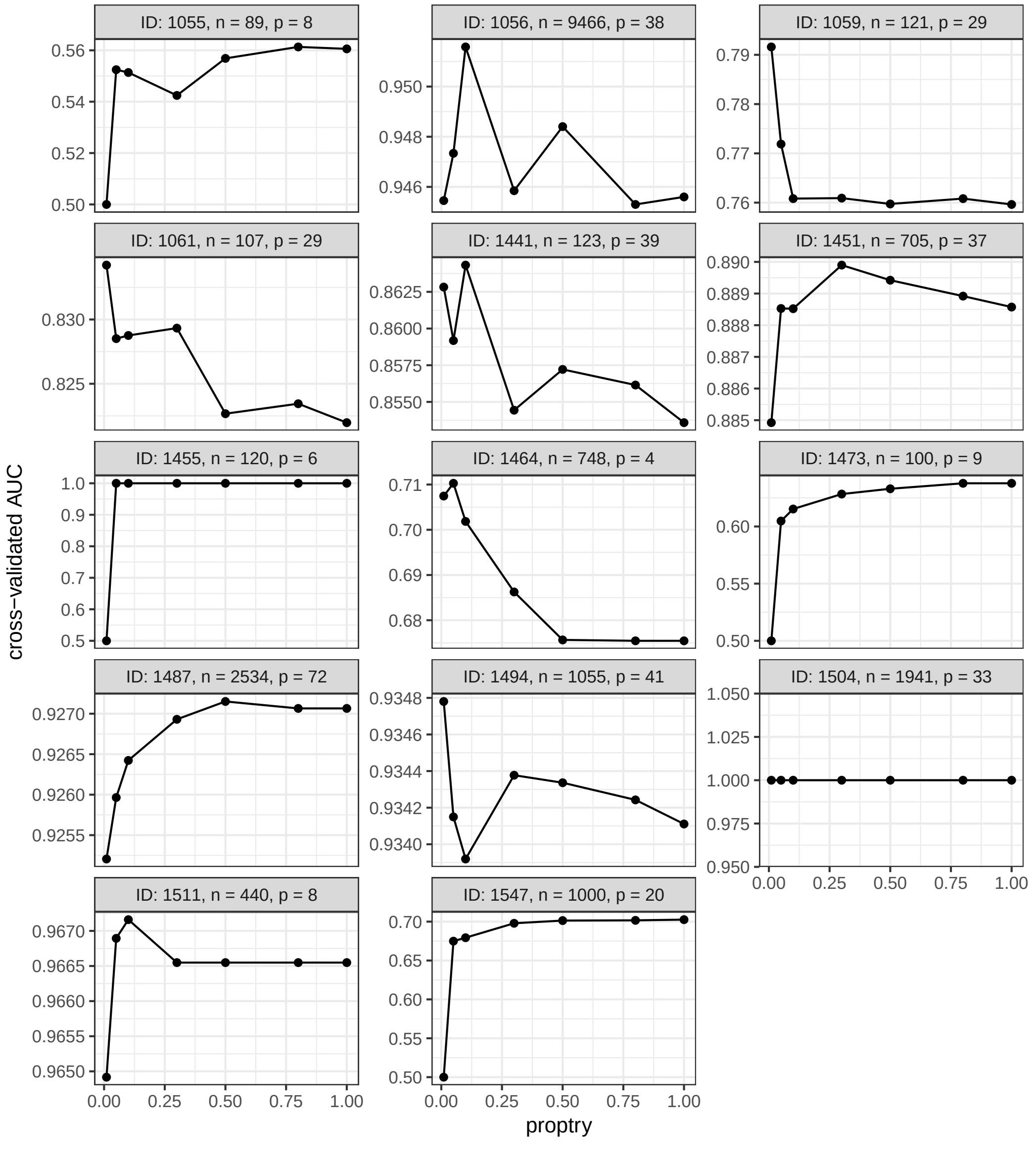

Fig. S9: Pre-study: Cross-validated AUC values obtained for different *proptry* values. For each *proptry* value considered, the plots show the maximum cross-validated AUC value obtained over the seven different values of *nsplits*. Each panel shows the results obtained for a particular data set. – III

C Data Set Specific Performances of RFsextr1 and RFsextr5 Compared to that of RFs

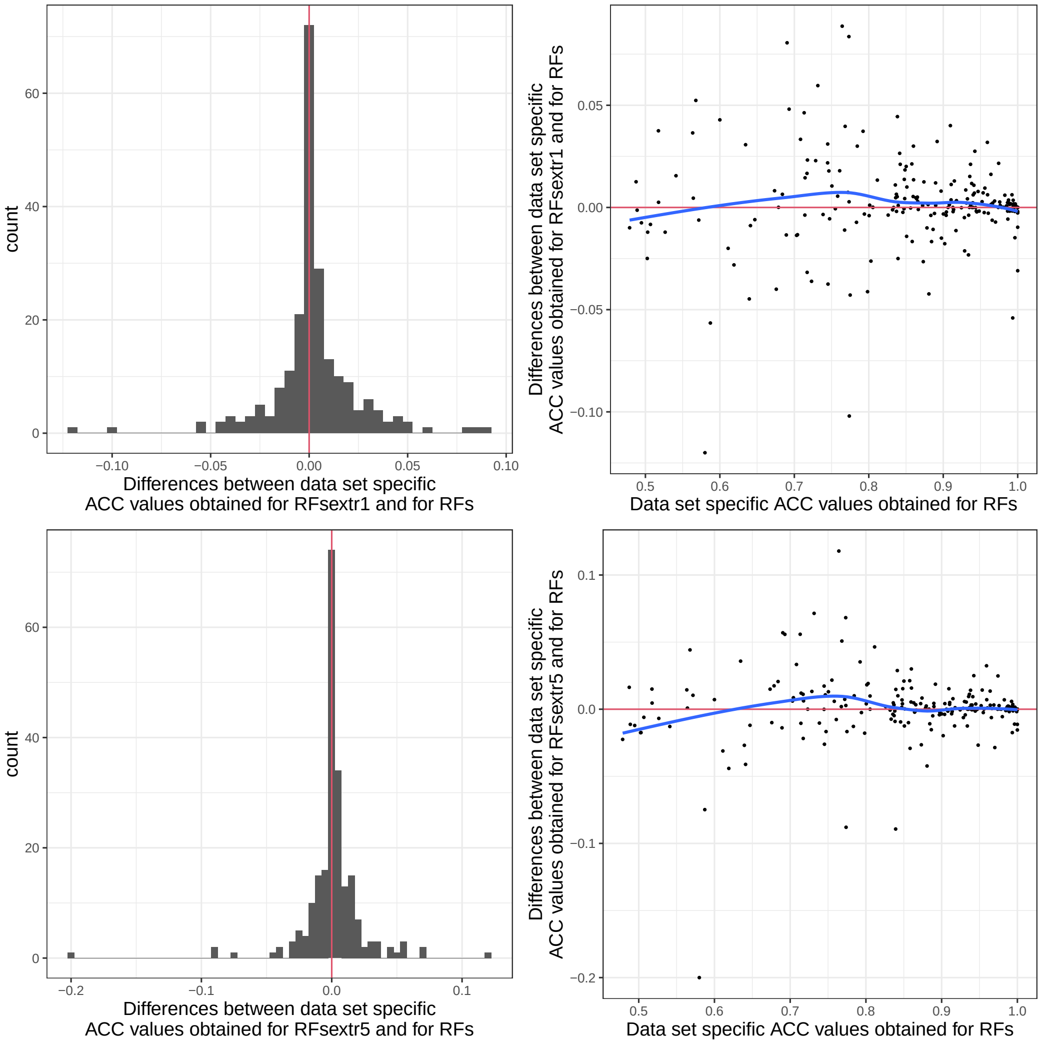

Fig. S10: Data set specific performances of RFsextr1 and RFsextr5 compared to that of RFs. Left panels: Histograms of the differences between the data set specific ACC values obtained for RFsextr1 / RFsextr5 and for RFs. The red lines indicate the zero line. Right panels: Scatter plot of the differences between the data set specific ACC values obtained for RFsextr1 / RFsextr5 and for RFs against the data set specific ACC values obtained for RFs. The blue lines represents LOESS fits. The red lines again indicate the zero line. The upper panels show the results obtained for RFsextr1 and the lower panels those obtained for RFsextr5

D Influence of Sample Size and Number of Features on the Performance of RFsextr1, RFsextr5 and RFs

In Figure S11 the influences of n and p on the performance of RFsextr1 and RFs are shown in a form analogous to Figure 2 in the main paper. The picture is different to Figure 2. For very small values of n RFsextr1 performs slightly better than RFs, but the difference is smaller compared to that obtained between DFs and RFs for small n (for the AUC, RFs are even slightly better than RFsextr1 for very small values of n; results not shown). Moreover, on average, for small p, RFsextr1 and RFs perform equally well, but for numbers of features larger than about ten RFsextr1 performs consistently slightly better than RFs. The results obtained for the three measures differ with respect to a potential interaction between the influences of n and p: For the ACC and, to a lesser degree, for the Brier score (results not shown), the observation that the improvement of RFsextr1 over RFs becomes stronger for larger p is more pronounced in the case of smaller n, but for the AUC it is more pronounced for larger n (results not shown).

The general picture obtained for RFsextr5 (Figure S12) is similar to that obtained for RFsextr1. Nevertheless, contrary to in the case of RFsextr1, for RFsextr5 we do not observe a general improvement over RFs for very small data sets and the trend of greater improvements for larger numbers of features is less clear. The former is probably because, as the analysis of the bivariable influence of n and p on the predictive performance in the lower panels of Figure S12 reveals, for RFsextr5 there is a small performance loss for data sets with very small n and at the same time small p.

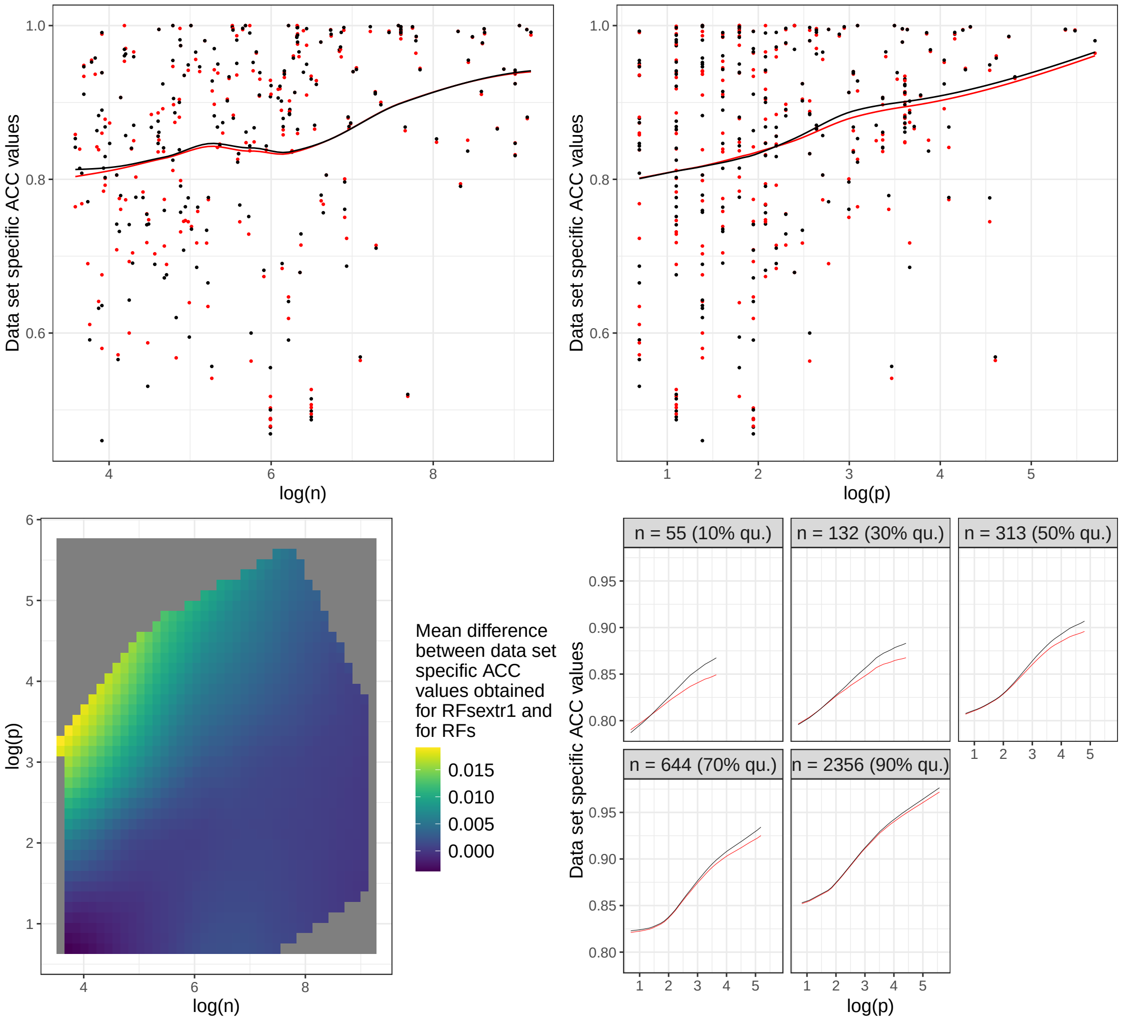

Fig. S11: Influence of sample size n and number of features p on the performance of RFsextr1 and RFs. Upper left / right panel: Data set specific ACC values obtained for RFsextr1 and RFs plotted against the logarithmized values of n and p. The lines show LOESS fits obtained for RFsextr1 and RFs, respectively. Lower left panel: Two-dimensional LOESS fit of the influences of the logarithmized values of n and p on the differences between the data set specific ACC values obtained for RFsextr1 and for RFs. Lower right panel: Cross sections of two-dimensional LOESS fits of the influences of the logarithmized values of n and p on the data set specific ACC values obtained for RFsextr1 and RFs, respectively. The cross sections were taken at different quantiles of the sample sizes of all data sets. Where applicable, in each plot the black lines show the results obtained for RFsextr1 and the red lines those obtained for RFs

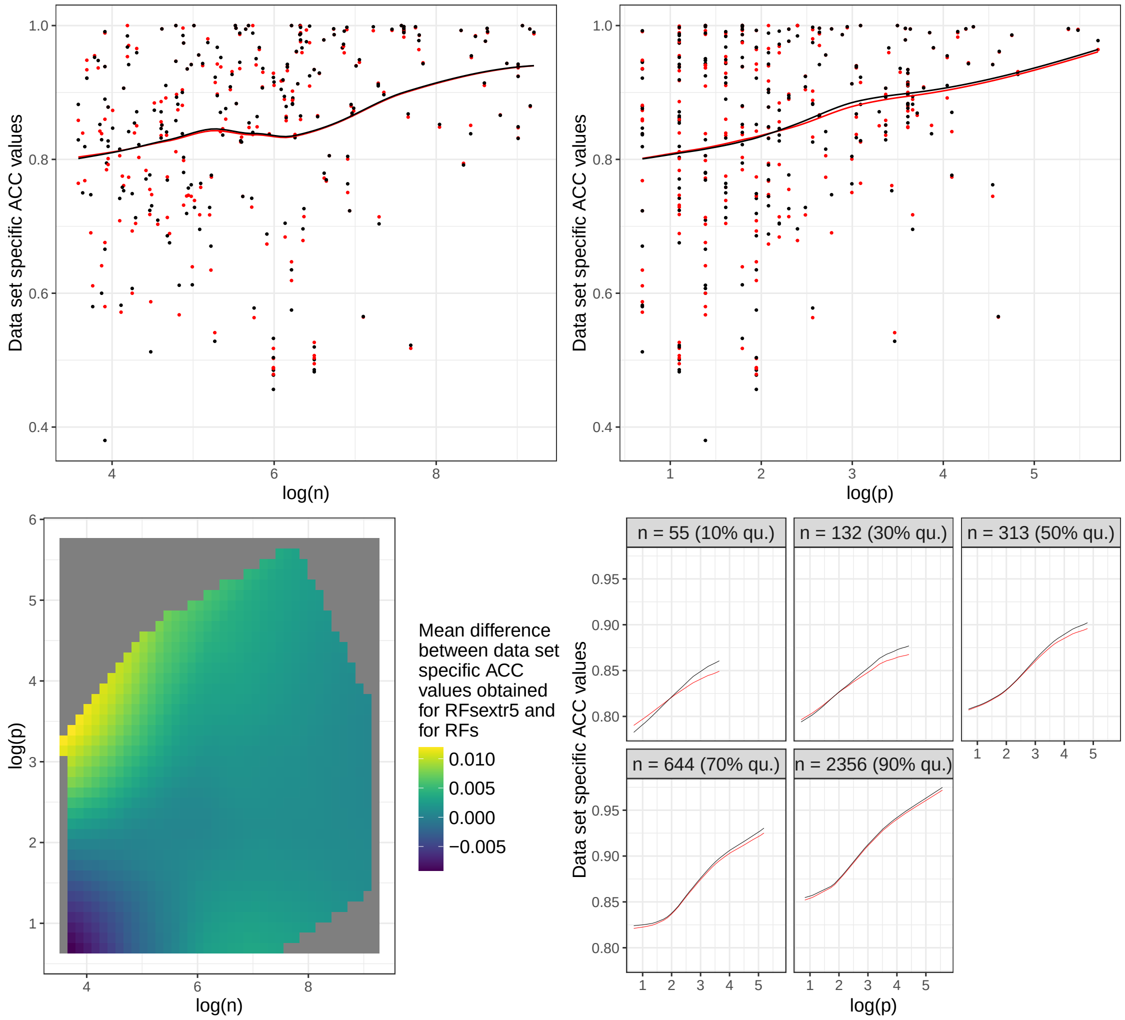

Fig. S12: Influence of sample size n and number of features p on the performance of RFsextr5 and RFs. Upper left / right panel: Data set specific ACC values obtained for RFsextr5 and RFs plotted against the logarithmized values of n and p. The lines show LOESS fits obtained for RFsextr5 and RFs, respectively. Lower left panel: Two-dimensional LOESS fit of the influences of the logarithmized values of n and p on the differences between the data set specific ACC values obtained for RFsextr5 and for RFs. Lower right panel: Cross sections of two-dimensional LOESS fits of the influences of the logarithmized values of n and p on the data set specific ACC values obtained for RFsextr5 and RFs, respectively. The cross sections were taken at different quantiles of the sample sizes of all data sets. Where applicable, in each plot the black lines show the results obtained for RFsextr5 and the red lines those obtained for RFs

E Influence of data set characteristics on the selected tuning parameters

E.1 DFs: Selected *proptry* Values

While the parameter *nsplits* of DFs was fixed to the value 30, for *proptry*, in each cross-validation iteration, the value out of 0.05 and 1 was selected that featured the smaller OOB prediction error.

In Figure S13 the relations between the selected *proptry* values and various quantities are illustrated. The sample size does not seem to have any relevant influence on which *proptry* value is selected (upper left panel of Figure S13). There seems to be a trend that $proptry = 1$ is selected slightly more often for larger numbers of features (upper right panel of Figure S13).

There is a clear relationship between the predictive information contained in the data and the selected *proptry* value (lower left panel of Figure S13): In cases in which the *proptry* value 1 was selected, the predictive performance of RFs was considerably stronger compared to in cases in which the selected *proptry* value was 0.05. Note that the selected *proptry* values were related to the predictive performance of RFs instead of that of DFs, because the actual interest lay in the relationship between the strength of the signal in the features and the selected *proptry* value. Using the predictive performance of DFs would not have been appropriate here for the following reason: Differences in the performance of DFs between the two different *proptry* values can also be due to the method simply performing differently well for these two *proptry* values, independent of the strengths of the signals in the data sets that are of actual interest. An explanation why DFs tend to choose $proptry = 1$ more frequently than $proptry = 0.05$ in the presence of stronger signals in the data, could be that, in situations in which the signal is strong, it is beneficial to sample many splits even for small nodes. This is because, if the signal is strong, there tend to be more features with effect, in which situation using many candidate splits for small nodes will not lead to overfitting, because a large proportion of these candidate splits will be informative. In contrast, if the signal is weak there are likely few features with effect, in which situation sampling many candidate splits from small nodes can result in overfitting because many candidate splits will not be informative in this situation, increasing the likelihood of selecting uninformative candidate splits that divide the training observations well simply by chance. Another reason why $proptry = 0.05$ might be preferrable for weak signals could be that choosing $proptry = 0.05$ has the effect that the trees are not grown to full size, but growing is stopped as soon as 0.05 times the number of possible splits is smaller than 1 (see Section 2 of the main paper). If the signal is weak, growing the trees to full size could lead to overfitting the training data.

Lastly, as seen in the lower right panel of Figure S13, RFs tended to sample larger proportions of features for data sets for which the *proptry* value 1 was selected more frequently, which is not surprising given that these data sets tend to be associated with stronger signals, as seen above.

E.2 RFs, RFsextr1, and RFsextr5: Selected *mtry* Values

The tuning parameter value optimized in the case of RFs, RFsextr1, and RFsextr5 was the number of features *mtry* sampled randomly for each split. In the following descriptions, for ease of presentation, the *mtry* values optimized for the different methods will frequently simply be denoted as the "*mtry* values" without referring to the fact that these values were optimized (using

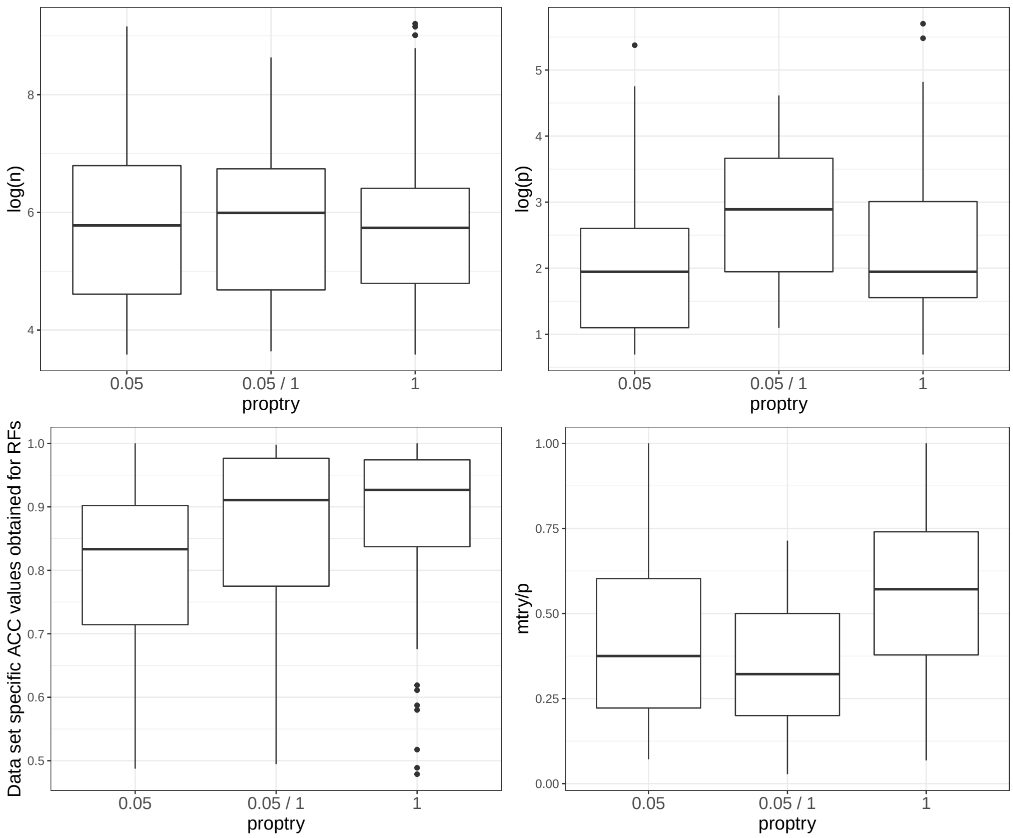

Fig. S13: Relationships between the selected *proptry* values and various quantities. The three categories "0.05", "0.05 / 1", and "1" for the selected *proptry* values shown in the plots were obtained as follows: First, for each data set the numbers of times that the values 0.05 and 1 were selected for *proptry* were counted in the 10 training iterations of the two times repeated 5-fold stratified cross-validation. Subsequently, data sets for which the *proptry* value 0.05 had been selected more often were assigned to the category "0.05", data sets for which the *proptry* value 1 had been selected more often to the category "1", and data sets for which both 0.05 and 1 had been selected equally often (i.e., five times each) to the category "0.05 / 1". Upper left panel: Boxplots showing the relationship between the logarithmized sample sizes and the selected *proptry* values. Upper right: Boxplots showing the relationship between the logarithmized numbers of features and the selected *proptry* values. Lower left: Boxplots showing the relationship between the data set specific ACC values obtained with RFs and the selected *proptry* values. Lower right: Boxplots showing the relationship between the *mtry* values selected by RFs divided by the numbers of features and the selected *proptry* values

the procedure described in Section 4.3.1 of the main paper). The upper left panel of Figure S14 shows, for each of the three methods, the distributions of the proportions $mtry/p$ of the features that were sampled per split. Obviously, RFsextr1 selected the largest numbers of candidate features, followed by RFsextr5 and RFs, where the difference between RFsextr5 and RFs is larger than that between RFsextr1 and RFsextr5.

For all three RF variants, we observe that the larger the number of features becomes, the smaller the proportions $mtry/p$ tend to become (upper right panel of Figure S14). This tendency is congruent with the very commonly used default choice for $mtry$, which is setting it equal to the square root of the number of features p. With this choice, $mtry/p$ decreases with larger values of p, because $mtry/p = \sqrt{p}/p = 1/\sqrt{p}$ for $mtry = \sqrt{p}$. However, as the black line in the upper right panel of Figure S14 shows, when using the default choice $mtry = \sqrt{p}$, the $mtry$ values tend to be specified too small, in particular for data sets with larger numbers of features. It is important to note that the (strong) increases of the three LOESS curves for very large values of p can be ascribed to a single data set. This data set with ID '312' has a strong influence on the curves because, not only does it feature the maximum number of features out of all data sets ($p = 299$), but also all three RF variants selected the maximum possible $mtry$ value (i.e., 299) for this data set. Because of these two facts, the three data points associated with this data set are at the same location found in the upper-right corner of the plot. Figure S15 shows a version of Figure S14 that excludes data set '312'. Here, only the LOESS curve associated with RFsextr1 shows a small increase for large values of p. For RFs and RFsextr5, the curves are monotonous decreasing, where in the case of RFs the decrease is strongly diminishing for larger values of p. Even though in Figure S15 we do not see (strong) increases of the curves for large values of p, the selected $mtry$ values still tend to be larger than those that would have resulted from the default choice $mtry = \sqrt{p}$, where the differences are particularly strong for large numbers of features. Probst et al. [2] investigated the gains in predictive performance attainable through tuning parameter optimization for various machine learning algorithms and determined new default choices for the respective tuning parameter values. Using a collection of 38 binary classification data sets, Probst et al. [2] determined the default value $mtry = p \cdot 0.432$ for obtaining optimal prediction accuracies with RFs, where in the optimization of this default value they assumed that the optimal values for $mtry$ are proportional to the number of features. Note that in Figures S14 and S15, given that the y axes show the values of $mtry/p$, the curves corresponding to the choice $mtry = p \cdot 0.432$ would be horizontal lines at the value 0.432. The fact that the LOESS lines corresponding to the results of the optimized $mtry$ values are decreasing shows that it is not viable to assume that the optimal $mtry$ values are proportional to the numbers of features. Using the rule $mtry = p \cdot 0.432$ will instead tend to deliver too small $mtry$ values for small numbers of features and too large $mtry$ values for large numbers of features. However, on average this rule seems to be appropriate because, interestingly, for RFs, the mean of the optimized $mtry$ values divided by the values of p is 0.470, which is close to the value 0.432 associated with using $mtry = p \cdot 0.432$. While the rule $mtry = p \cdot 0.432$ does thus not model the dependency between p and the optimal $mtry$ values exactly, it does deliver much better $mtry$ values than the conventional rule $\sqrt{p}$. Nevertheless, capturing the precise mean dependency of the optimal $mtry$ values on the numbers of features is of limited importance, because, as the large variability of the points in the scatter plot (upper right panel of Figure S14) reveals, the influence of p on the optimal $mtry$ value is not strong.

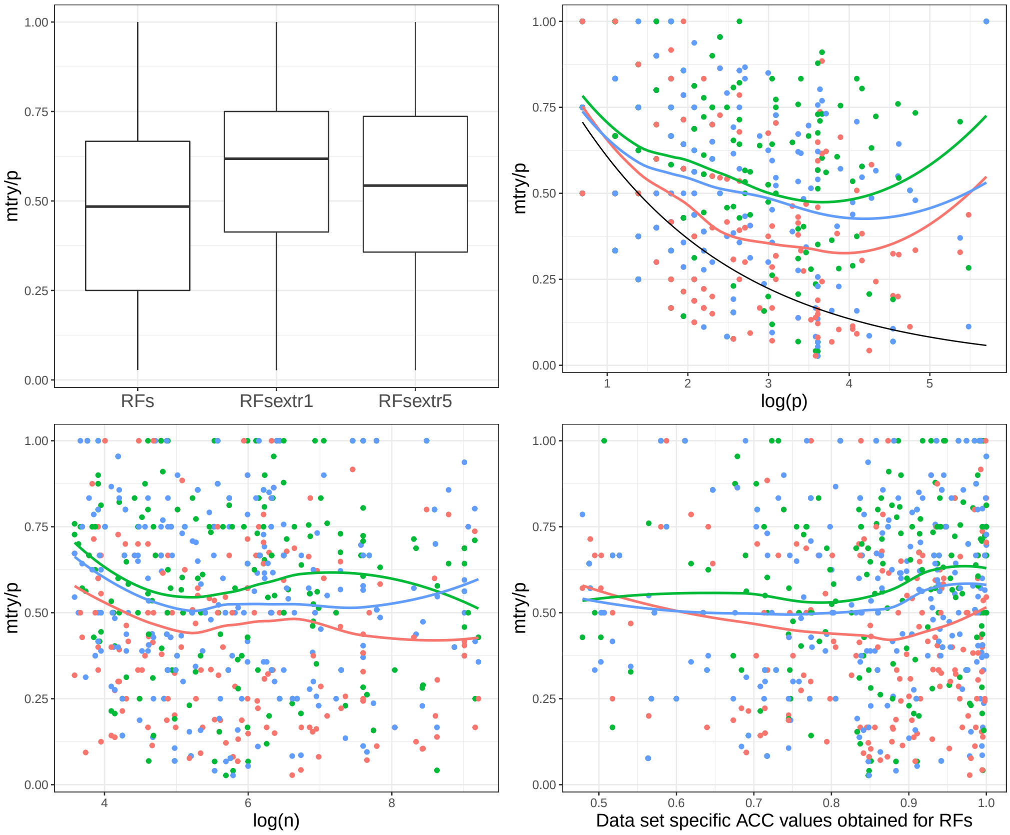

Fig. S14: Relationships between the *mtry* values selected by RFs, RFsextr1, and RFsextr5 and various quantities. Analogous to the analysis of the *proptry* values selected by DFs, for each data set a single *mtry* value was considered in the plots. These data set specific *mtry* values were obtained by taking the median of the *mtry* values selected in the 10 training iterations of the two times repeated 5-fold stratified cross-validation. Upper left panel: Boxplots showing the *mtry* values divided by the numbers of features selected by RFs, RFsextr1, and RFsextr5. Upper right / lower left / lower right panel: *mtry* values divided by the numbers of features plotted against the logarithmized values of the numbers of features (upper right panel), the logarithmized values of the sample size (lower left panel), and the data set specific ACC values obtained for RFs (lower right panel). The black line in the upper right panel shows the $mtry/p$ values associated with the default choice $mtry = \sqrt{p}$. The different colors distinguish the different methods, where the results obtained for RFs are shown in red, those obtained for RFsextr1 in green and those obtained for RFsextr5 in blue. The colored lines show LOESS fits.

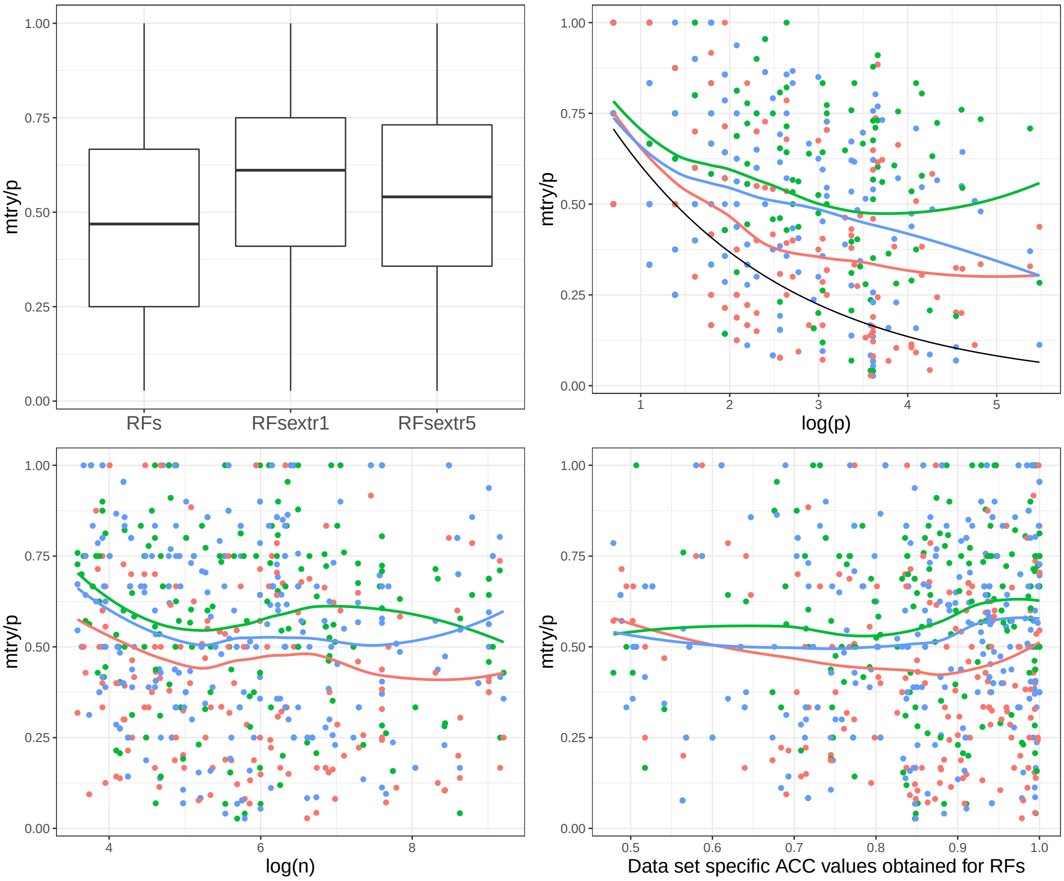

Fig. S15: Relationships between the *mtry* values selected by RFs, RFsextr1, and RFsextr5 and various quantities—excluding data set '312'. Analogous to the analysis of the *proptry* values selected by DFs, for each data set a single *mtry* value was considered in the plots. These data set specific *mtry* values were obtained by taking the median of the *mtry* values selected in the 10 training iterations of the two times repeated 5-fold stratified cross-validation. Upper left panel: Boxplots showing the *mtry* values divided by the numbers of features selected by RFs, RFsextr1, and RFsextr5. Upper right / lower left / lower right panel: *mtry* values divided by the numbers of features plotted against the logarithmized values of the numbers of features (upper right panel), the logarithmized values of the sample size (lower left panel), and the data set specific ACC values obtained for RFs (lower right panel). The black line in the upper right panel shows the $mtry/p$ values associated with the default choice $mtry = \sqrt{p}$. The different colors distinguish the different methods, where the results obtained for RFs are shown in red, those obtained for RFsextr1 in green and those obtained for RFsextr5 in blue. The colored lines show LOESS fits

The influence of the sample size (lower left panel of Figure S14) on the values of $mtry/p$ is weak and neither monotonous decreasing nor increasing. Before further interpreting the nature of the influence of the sample size, it is important to note that the sample sizes and the numbers of features of the data sets are weakly and positively correlated, with the Pearson correlation between the logarithmized versions of these quantities taking the value 0.30. For this reason, the influence of the sample size cannot be fully distinguished from that of the number of features. In order to exclude that the observed influence of the sample size in Figure S14 primarily reflects the

influence of the number of features, the influence of the sample size was additionally investigated in a stratified fashion, where the different strata were defined according to the numbers of features: Figure S16 shows the influence of the sample size stratified for data sets with small ($p \leq 5$, 75 data sets), medium ($5 < p \leq 15$, 79 data sets), and large ($p > 15$, 66 data sets) numbers of features. The general forms of the observed influences are similar to those observed in the lower left panel of Figure S14 suggesting that they are sufficiently robust towards a potential confounding by the influence of the numbers of features. For all three RF variants, the optimal *mtry* values tend to become larger in the direction of very small sample sizes. An explanation for this phenomenon could be that for very small sample sizes it is primarily important to obtain sufficiently strong tree predictors by exploiting the predictive information contained in the strong features. This is indeed achieved through using a large *mtry*, because the strong features will appear more often among the sampled features for large *mtry* values. If smaller *mtry* values were used, the tree predictors and their predictions would be more diverse, but many of the splits in these tree predictors would perform badly. This is due to the fact that, for smaller *mtry* values, the sampled features would often miss many of the strong features and, due to the high variance associated with the split selection for very small sample sizes, good splits are difficult to identify in sets of weaker features. To summarize, for very small sample sizes, it seems to be important to put a strong focus on the first factor "tree predictor performance" of the two factors influencing the predictive performance of RFs that were identified by Breiman [1]. In contrast for very large sample sizes, the *mtry* values tend to become smaller. This can be interpreted as that, for very large sample sizes the tree predictors perform well, even when sampling small numbers of features. This is because the selected splits in the sampled features, that is, those splits that feature the optimal value of the split criterion, will perform comparable to the true best splits in the considered sets of splits. The fact that the split selection is so precise for very large sample sizes and that the tree predictors thus also perform well for smaller *mtry* values makes it profitable to reduce the correlations between the tree predictions by choosing smaller *mtry* values. Beyond the observations made above for very small and very large sample sizes, the selected *mtry* values tend to become smaller when shifting from very small to small sample sizes, before becoming larger for large sample sizes and smaller again for very large sample sizes. The above explanations of the results obtained for very small and very large sample sizes were based on the two factors influencing the predictive performance of RFs identified by Breiman [1]. For moderately small and moderately large sample sizes, the obtained results might be explainable by a larger degree of overfitting of RF variants for larger *mtry* values. This larger degree of overfitting can be explained by the fact that, the more features are considered per split, the more extensively the training data is used for constructing the RF variant. An extensive use of the training data is directly associated with a larger probability of overfitting, that is, capturing of artifacts in the observed data that are not generalizable to independent observations for the purpose of prediction. For smaller sample sizes the danger of overfitting is higher than for large sample sizes, which, taking into account the relationship between *mtry* value and susceptibility to overfitting, might explain why for smaller sample sizes the selected *mtry* values are smaller than for large sample sizes.

Lastly, the lower right panel of Figure S14 shows the relationship between the prediction accuracy obtained with RFs and the proportions $mtry/p$ of features from all features sampled per split for each of the three RF variants. Overall, there does not seem to be a strong influence of the

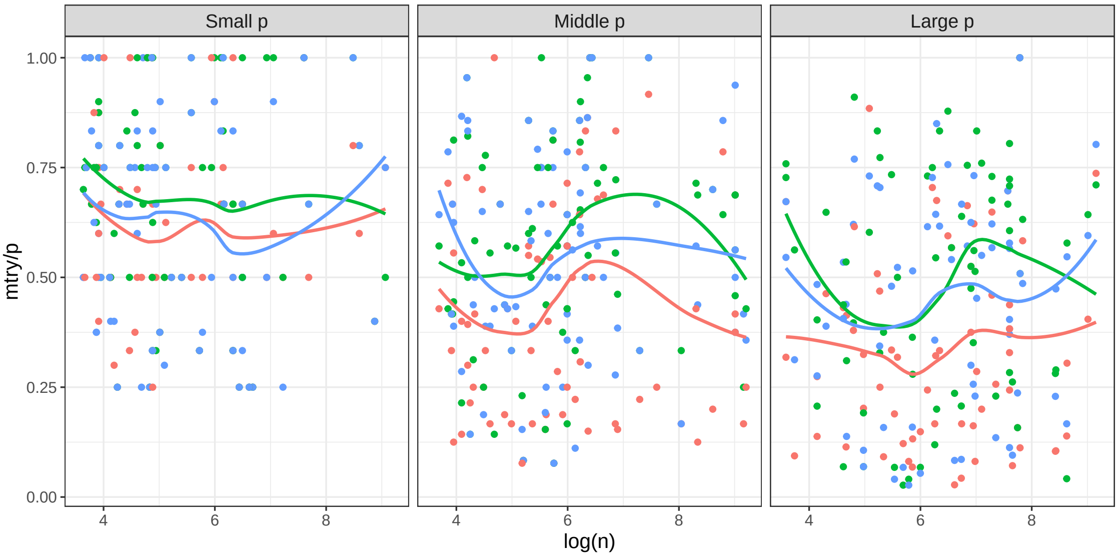

Fig. S16: Relationships between the *mtry* values selected by RFs, RFsextr1, and RFsextr5 and the logarithmized values of the sample size stratified according to the numbers of features in the data sets. The data set specific *mtry* values shown in the plots were obtained in the same way as in the case of Figure S15. Left / middle / right panel: results obtained for the data sets with small ($p \leq 5$, 75 data sets), medium ($5 < p \leq 15$, 79 data sets), and large ($p > 15$, 66 data sets) numbers of features. The different colors distinguish the different methods, where the results obtained for RFs are shown in red, those obtained for RFsextr1 in green and those obtained for RFsextr5 in blue. The lines show LOESS fits

prediction accuracy obtained with RFs as a measure of the predictive information in the features on the numbers of sampled features for any of the three RF variants. However, the plot suggests that the greater the predictive information in the features, the greater the degree by which RFsextr1 and RFsextr5 tend to sample more features than RFs. These results can again be explained by the tradeoffs that the methods make between the two factors influencing the predictive performance of RFs and its variants. Data sets that feature greater predictive information tend to contain more strong features. In this situation, for RFs the optimal *mtry* value will not be as large as in the case of RFsextr1 and RFsextr5, because, for RFs, the tree predictors will also perform well when smaller numbers of features are considered per split. Here, a very large *mtry* value would make the tree predictions of RFs too similar without substantially increasing the predictive performance of the tree predictors. In contrast, for RFsextr1 and RFsextr5, a large *mtry* value will not make the tree predictions too similar in this setting, because the fact that the splits are randomly drawn from the sampled features inhibits that similar (or the same) splits performed with the same features are selected too often. For these methods a large *mtry* value will increase the performance of the individual tree predictors without making their predictions too similar.

Above, the influences of different quantities on the selected *mtry* values were investigated. As seen in the corresponding scatter plots in Figure S14, these influences are not strong. Probst et al. [2] showed that, in general, the influence of *mtry* on the predictive performance of RFs is relatively small. For this reason it does not seem fruitful to put much emphasis on studying

factors that influence the optimal choice for *mtry*. The above investigations are, however, helpful for understanding the behavior of RFs and their variants.

We saw above that, when randomizing the split selection using RFsextr1 and RFsextr5, the optimal *mtry* values divided by the numbers of features tended to be larger than in the case of RFs. At the same time, as seen in Figure S15, for all three methods, the larger the numbers of features become, the smaller the optimal *mtry* values divided by the numbers of features tend to be. Therefore, it can be expected that the optimal number of candidate splits to draw will not be excessively large for high-dimensional feature data.

Studying the mean OOB prediction errors obtained for the different *mtry* values per data set in Online Resource 3 reveals the following: Even though the optimal *mtry* values tended to be relatively large for RFsextr1 and RFsextr5, in the great majority of cases smaller *mtry* values would have been associated with an only slightly worse predictive performance for all three RF variants. When focusing on the 44 data sets with numbers of features larger than 30, for RFsextr1, only in four data sets did the difference between the mean OOB prediction error obtained for the *mtry* value of $\sqrt{p}$ and the minimum mean OOB prediction error obtained for the *mtry* values larger than $\sqrt{p}$ exceed the value 0.02. These data sets were '312', '1441', '851', and '164'. Inspecting the corresponding plots in Online Resource 3 reveals that the mean OOB prediction errors tend to be very similar for larger *mtry* values even for these four data sets. Typically, the mean OOB prediction errors are considerably larger for very small *mtry* values, but take similar, small values for larger *mtry* values. Similar observations were made for DFs with regard to the values of *proptry* (see Figures S1, S2, and S3).

Two further interesting observations that can be made when studying Online Resource 3 are the following: For RFs the curves of the mean OOB prediction errors tend to reach clear minima for relatively small *mtry* values, with the mean OOB prediction errors gradually rising again for larger *mtry* values after reaching the minimum. For RFsextr1 and RFsextr5, in contrast, the curves frequently do not reach a sharp minimum, but tend to take very similar values for large and very large *mtry* values after reaching the minimum. This illustrates that, as explained above, the performance of RFsextr1 and RFsextr5 suffers less from overly large *mtry* values than that of RFs.

References

[1] Breiman L. Random forests. Mach Learn, 2001;45(1):5–32. https://doi.org/10.1023/A:1010933404324.

[2] Probst P, Boulesteix AL, Bischl B. Tunability: Importance of hyperparameters of machine learning algorithms. J Mach Learn Res, 2019;20(53):1–32.
