# Supplementary material for: Diversity Forests: Using Split Sampling to Enable Innovative Complex Split Procedures in Random Forests
Source: SN Comput Sci. 2021 Oct 21;3(1):1. doi: 10.1007/s42979-021-00920-1 (PMC8533673; doi:10.1007/s42979-021-00920-1)
Supplement: Supplementary file 2 — Supplementary file1 (ZIP 108032 KB) [file 42979_2021_920_MOESM2_ESM.zip › Online_Resource_2/Results/Figures/OnlineResource3.pdf]

Online Resource 3 to the article 'Diversity Forests: Using Split Sampling to Enable Innovative Complex Split Procedures in Random Forests' by Roman Hornung* published in the journal SN Computer Science

Mean OOB prediction errors obtained for the different considered 'mtry' values per data set

* Institute for Medical Information Processing, Biometry and Epidemiology, University of Munich, Marchioninistr. 15, 81377 Munich, Germany; e−mail: hornung@ibe.med.uni−muenchen.de

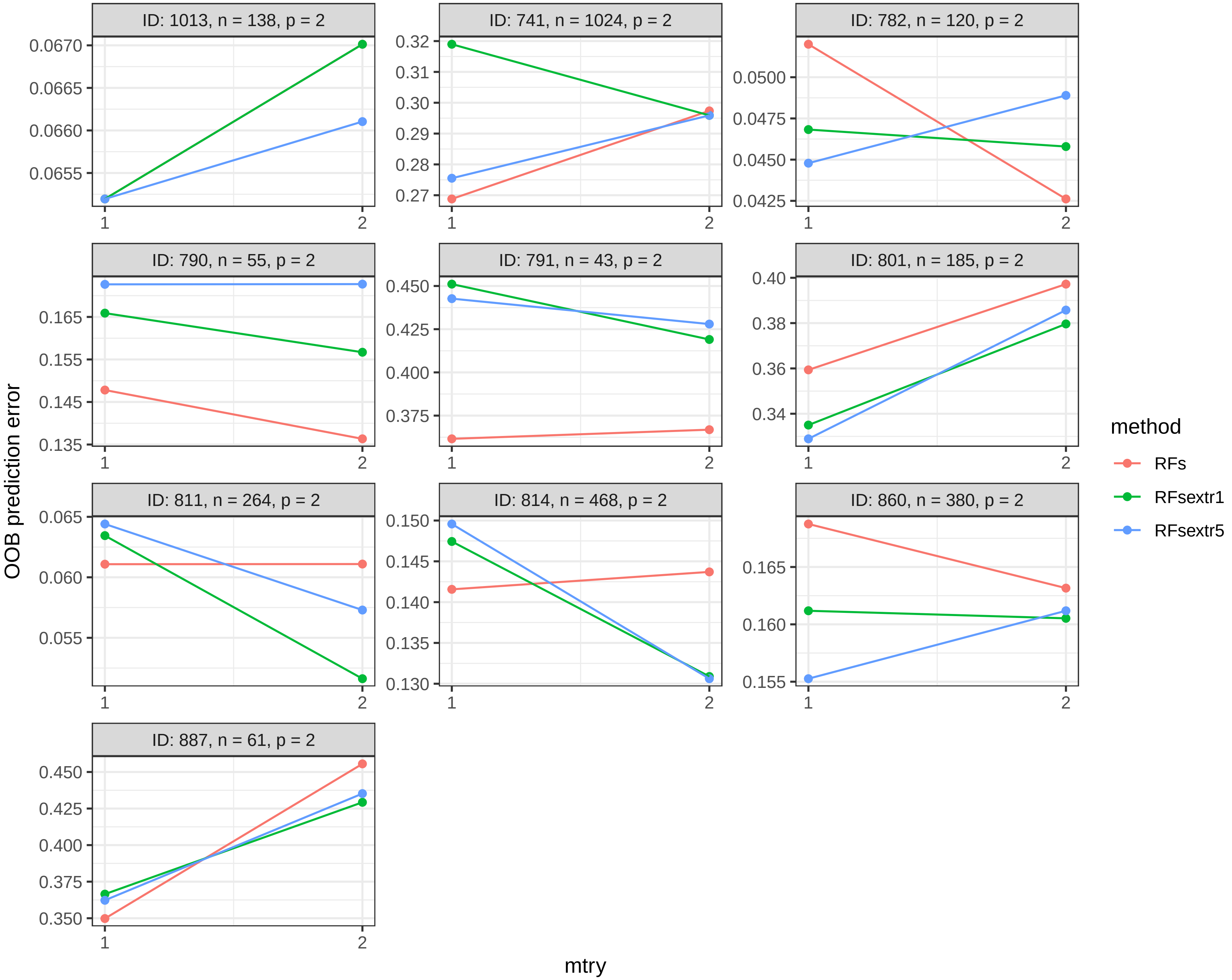

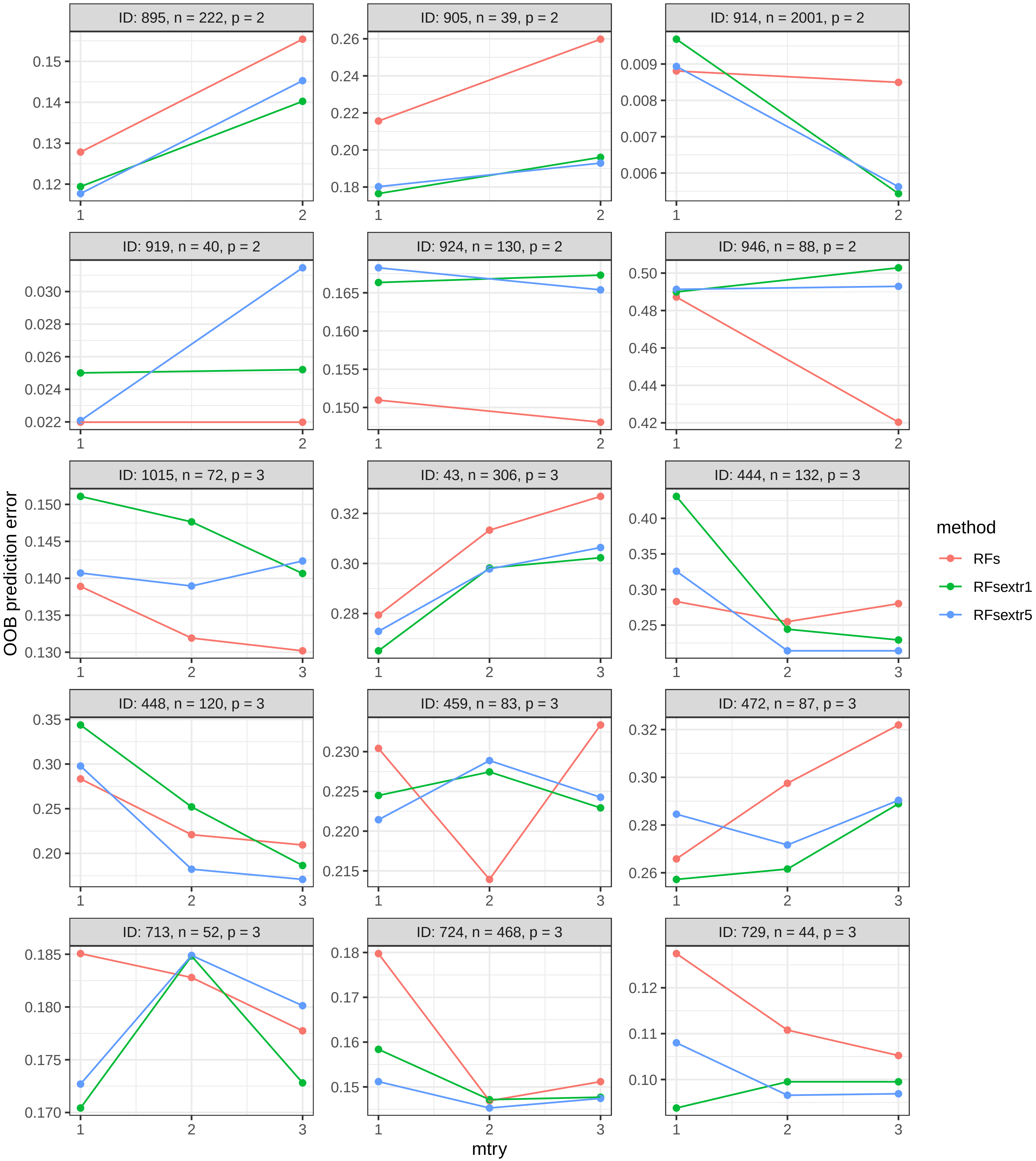

ID: 895, n = 222, p = 2
ID: 905, n = 39, p = 2
ID: 914, n = 2001, p = 2
ID: 919, n = 40, p = 2
ID: 924, n = 130, p = 2
ID: 946, n = 88, p = 2
ID: 1015, n = 72, p = 3
ID: 43, n = 306, p = 3
ID: 444, n = 132, p = 3
ID: 448, n = 120, p = 3
ID: 459, n = 83, p = 3
ID: 472, n = 87, p = 3
ID: 713, n = 52, p = 3
ID: 724, n = 468, p = 3
ID: 729, n = 44, p = 3
OOB prediction error
mtry
method
RFs
RFsextr1
RFsextr5

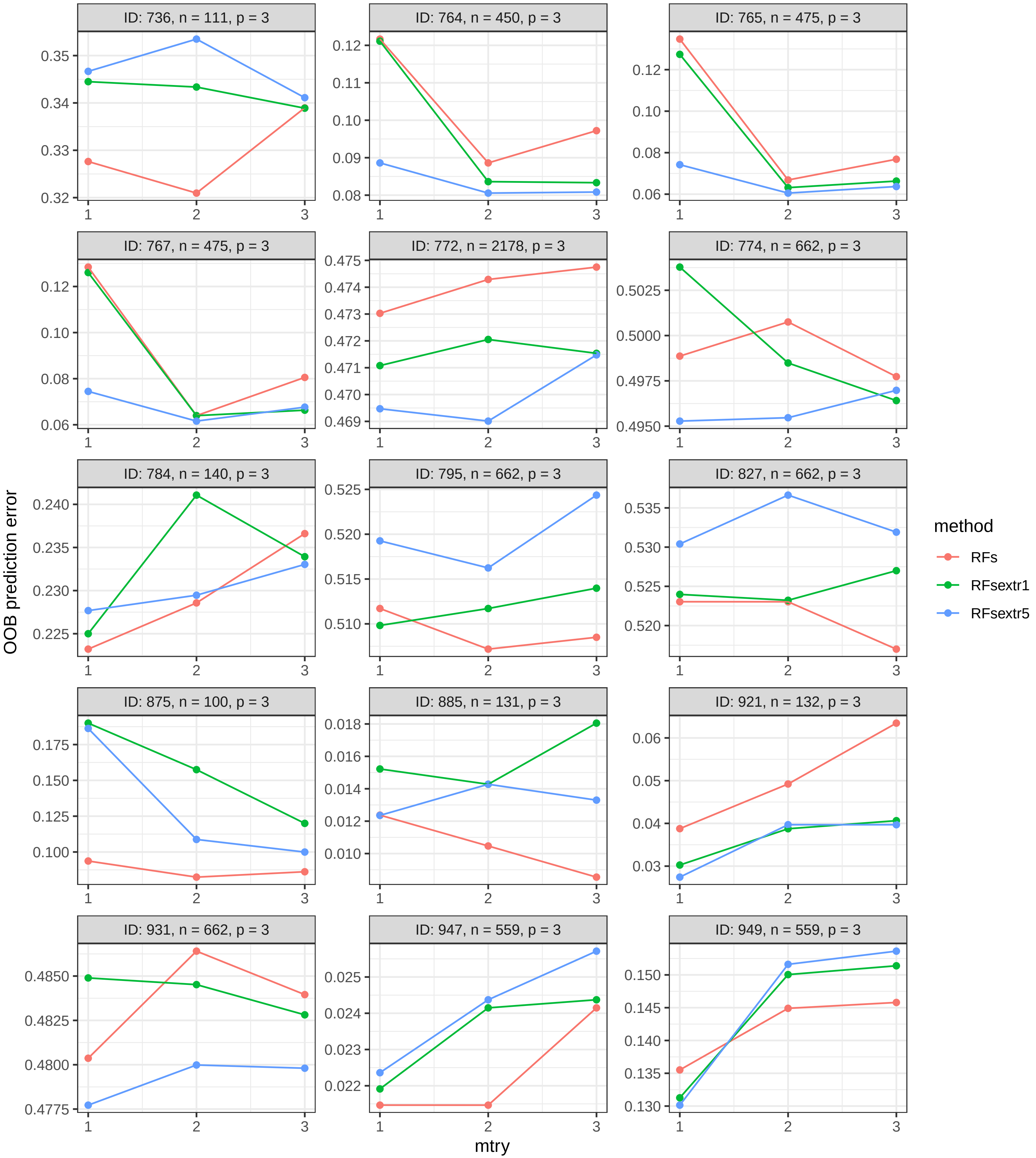
ID: 736, n = 111, p = 3
ID: 764, n = 450, p = 3
ID: 765, n = 475, p = 3
ID: 767, n = 475, p = 3
ID: 772, n = 2178, p = 3
ID: 774, n = 662, p = 3
ID: 784, n = 140, p = 3
ID: 795, n = 662, p = 3
ID: 827, n = 662, p = 3
ID: 875, n = 100, p = 3
ID: 885, n = 131, p = 3
ID: 921, n = 132, p = 3
ID: 931, n = 662, p = 3
ID: 947, n = 559, p = 3
ID: 949, n = 559, p = 3
OOB prediction error
mtry
method
RFs
RFsextr1
RFsextr5

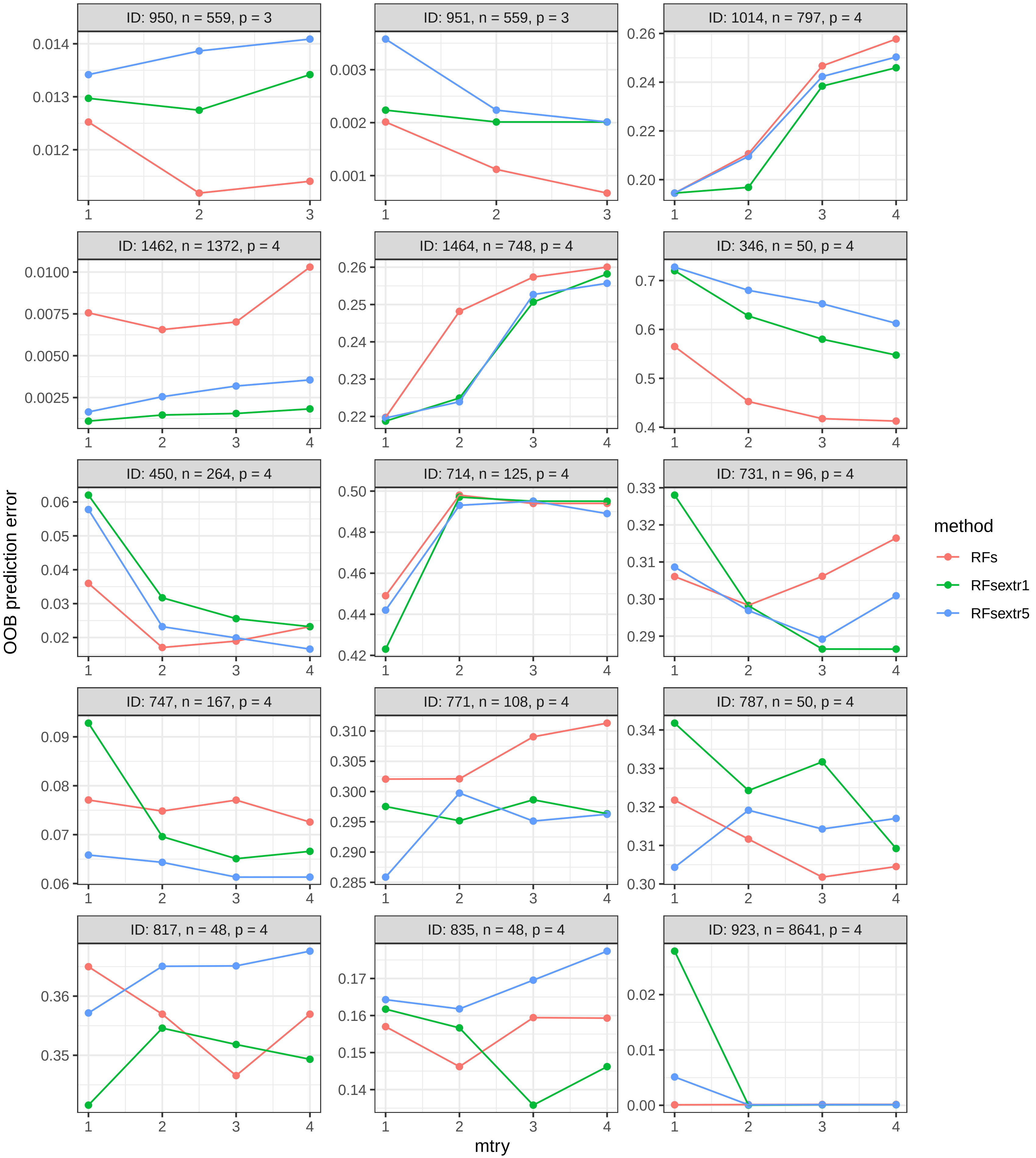
ID: 950, n = 559, p = 3
ID: 951, n = 559, p = 3
ID: 1014, n = 797, p = 4
ID: 1462, n = 1372, p = 4
ID: 1464, n = 748, p = 4
ID: 346, n = 50, p = 4
ID: 450, n = 264, p = 4
ID: 714, n = 125, p = 4
ID: 731, n = 96, p = 4
ID: 747, n = 167, p = 4
ID: 771, n = 108, p = 4
ID: 787, n = 50, p = 4
ID: 817, n = 48, p = 4
ID: 835, n = 48, p = 4
ID: 923, n = 8641, p = 4
OOB prediction error
mtry
method
RFs
RFsextr1
RFsextr5

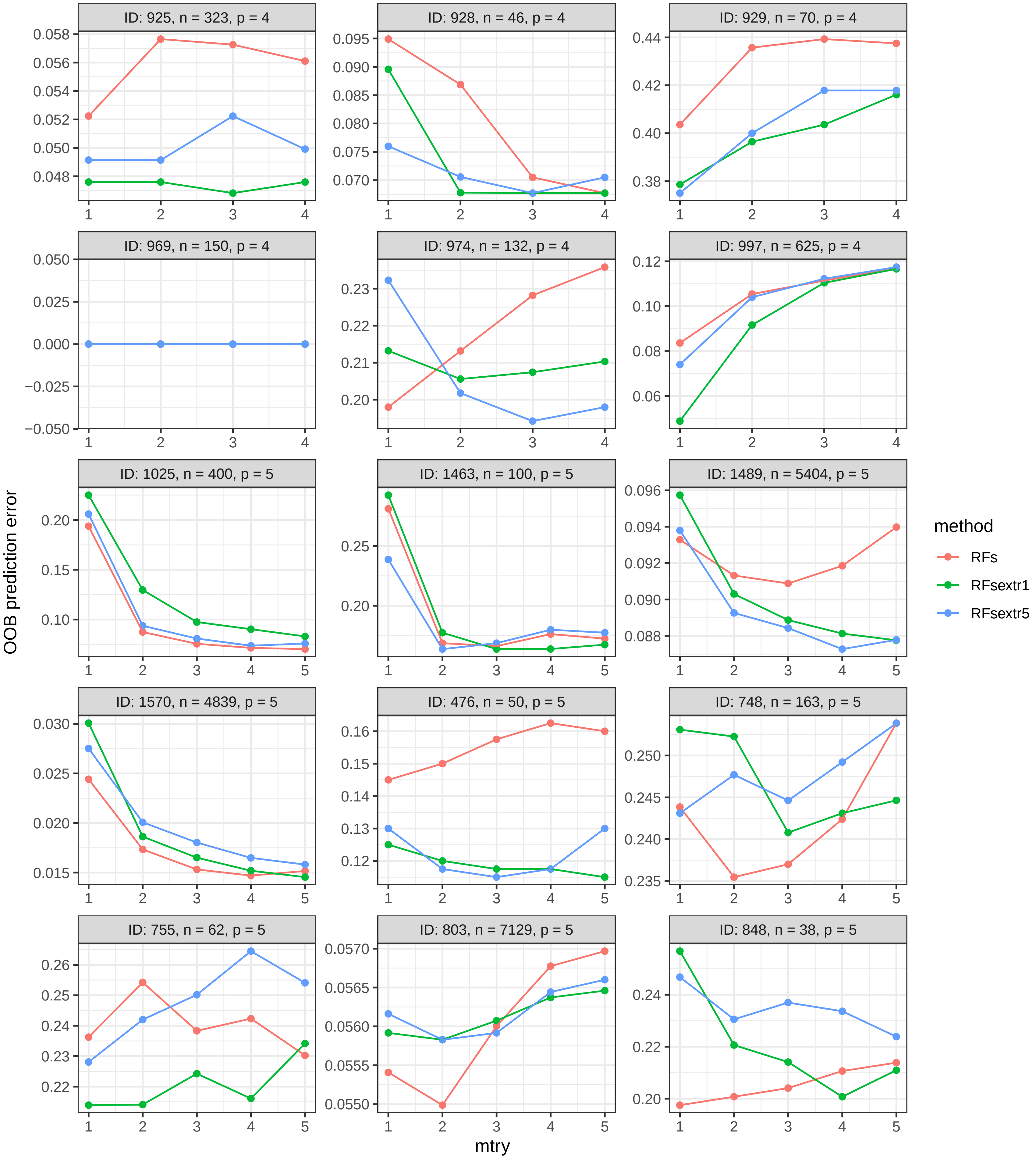
ID: 925, n = 323, p = 4
ID: 928, n = 46, p = 4
ID: 929, n = 70, p = 4
ID: 969, n = 150, p = 4
ID: 974, n = 132, p = 4
ID: 997, n = 625, p = 4
ID: 1025, n = 400, p = 5
ID: 1463, n = 100, p = 5
ID: 1489, n = 5404, p = 5
ID: 1570, n = 4839, p = 5
ID: 476, n = 50, p = 5
ID: 748, n = 163, p = 5
ID: 755, n = 62, p = 5
ID: 803, n = 7129, p = 5
ID: 848, n = 38, p = 5
OOB prediction error
mtry
method
RFs
RFsextr1
RFsextr5

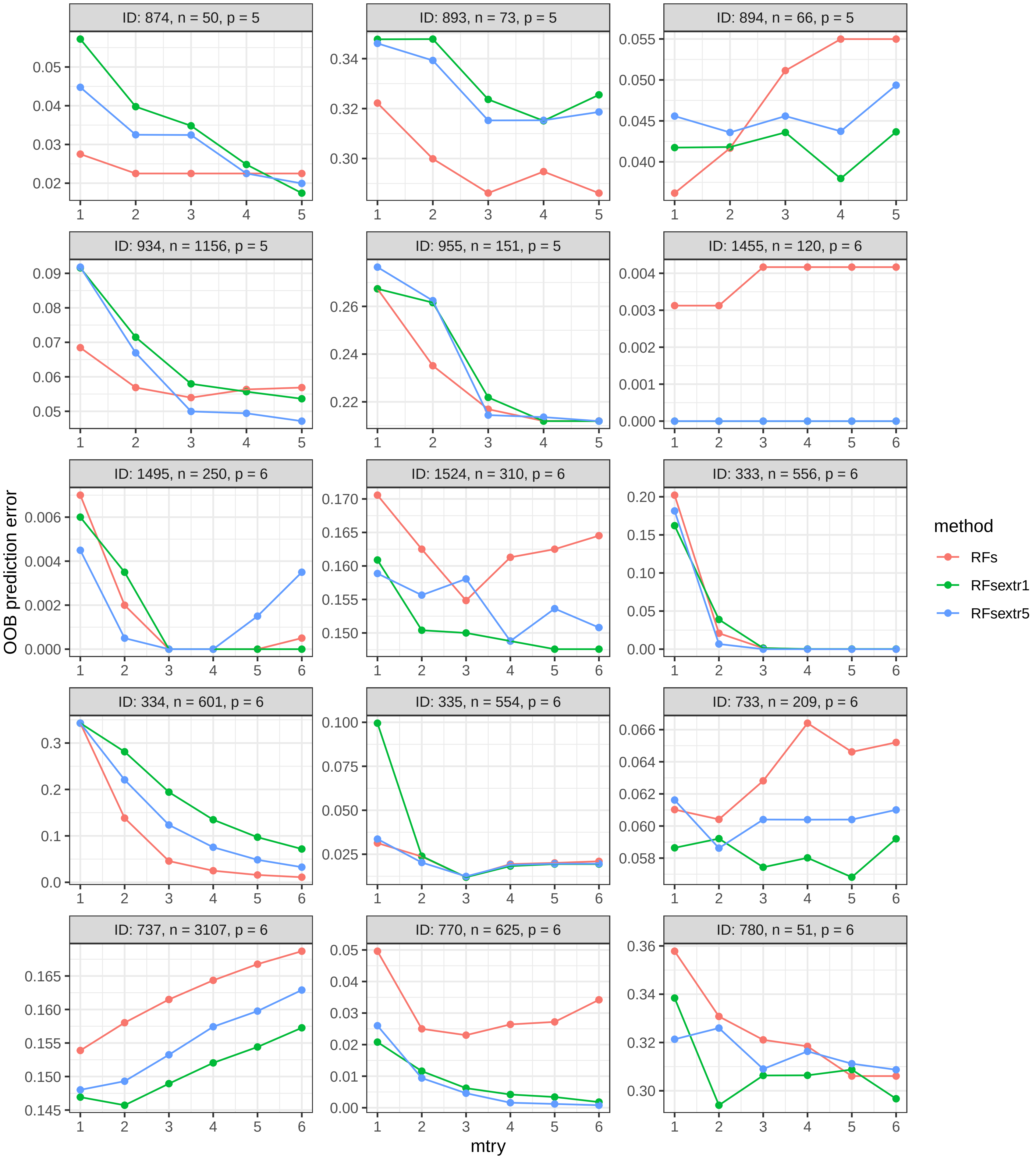

ID: 874, n = 50, p = 5
ID: 893, n = 73, p = 5
ID: 894, n = 66, p = 5
ID: 934, n = 1156, p = 5
ID: 955, n = 151, p = 5
ID: 1455, n = 120, p = 6
ID: 1495, n = 250, p = 6
ID: 1524, n = 310, p = 6
ID: 333, n = 556, p = 6
ID: 334, n = 601, p = 6
ID: 335, n = 554, p = 6
ID: 733, n = 209, p = 6
ID: 737, n = 3107, p = 6
ID: 770, n = 625, p = 6
ID: 780, n = 51, p = 6
OOB prediction error
mtry
method
RFs
RFsextr1
RFsextr5

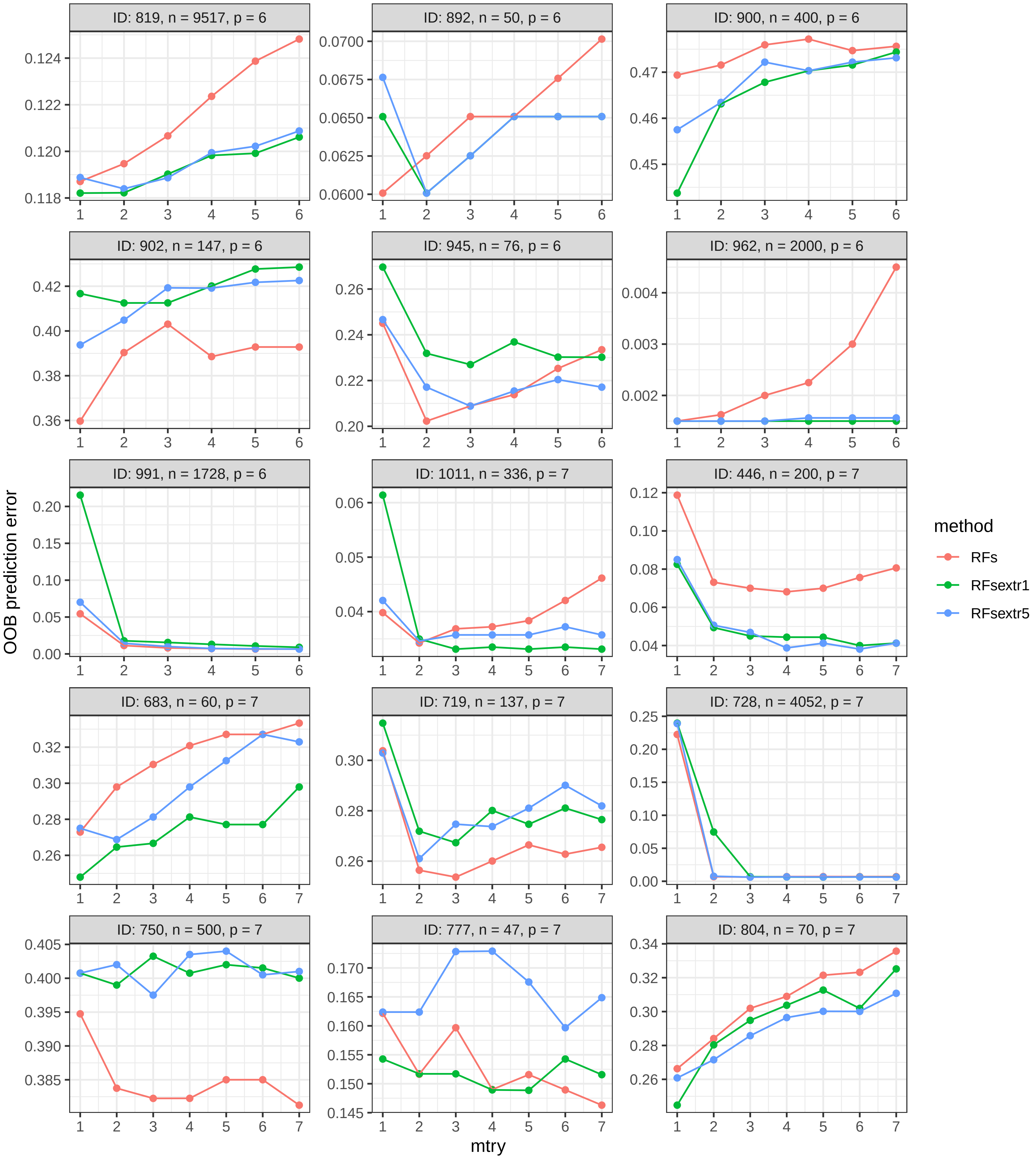
ID: 819, n = 9517, p = 6
ID: 892, n = 50, p = 6
ID: 900, n = 400, p = 6
ID: 902, n = 147, p = 6
ID: 945, n = 76, p = 6
ID: 962, n = 2000, p = 6
ID: 991, n = 1728, p = 6
ID: 1011, n = 336, p = 7
ID: 446, n = 200, p = 7
ID: 683, n = 60, p = 7
ID: 719, n = 137, p = 7
ID: 728, n = 4052, p = 7
ID: 750, n = 500, p = 7
ID: 777, n = 47, p = 7
ID: 804, n = 70, p = 7
OOB prediction error
mtry
method
RFs
RFsextr1
RFsextr5

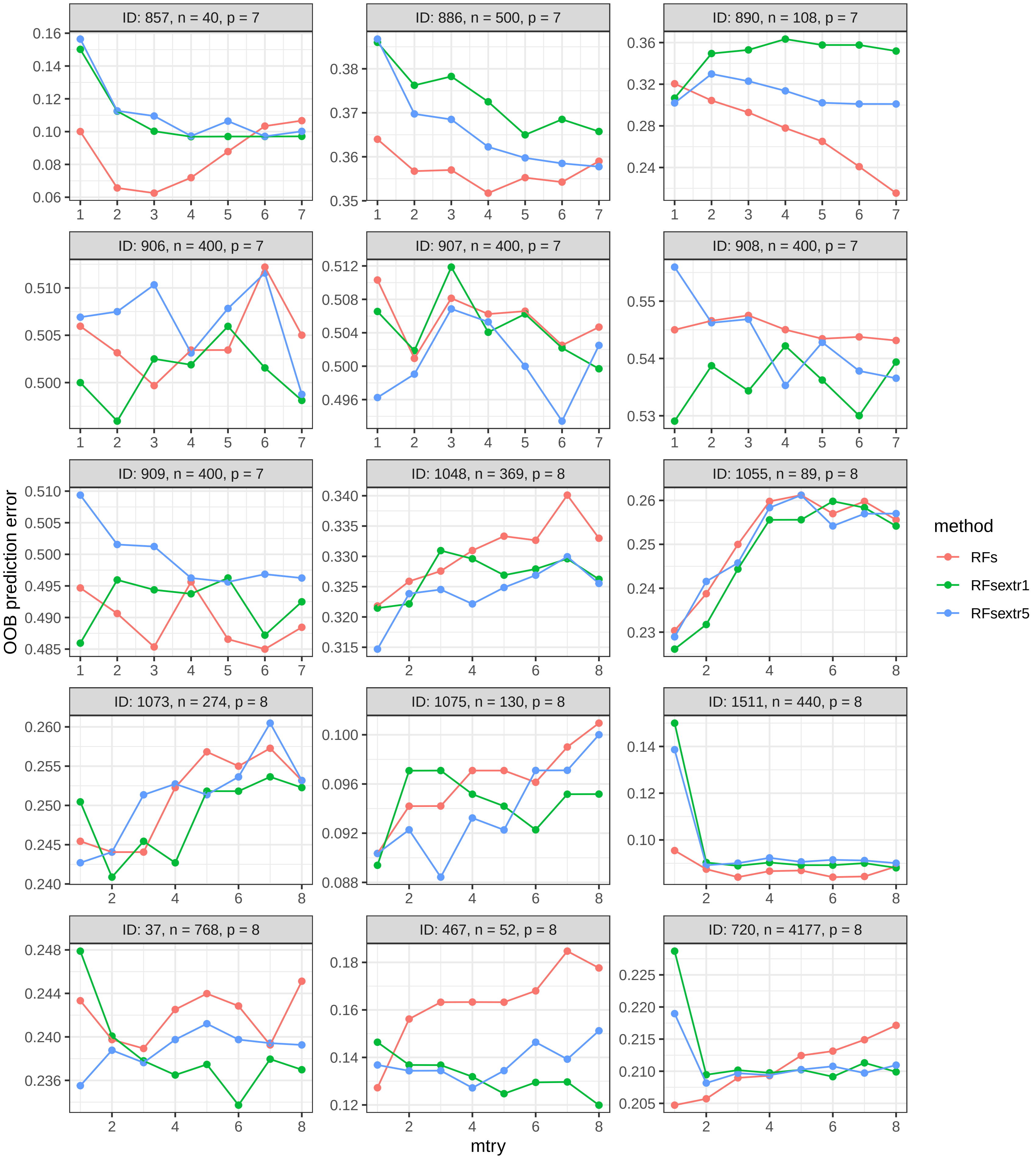
ID: 857, n = 40, p = 7
ID: 886, n = 500, p = 7
ID: 890, n = 108, p = 7
ID: 906, n = 400, p = 7
ID: 907, n = 400, p = 7
ID: 908, n = 400, p = 7
ID: 909, n = 400, p = 7
ID: 1048, n = 369, p = 8
ID: 1055, n = 89, p = 8
ID: 1073, n = 274, p = 8
ID: 1075, n = 130, p = 8
ID: 1511, n = 440, p = 8
ID: 37, n = 768, p = 8
ID: 467, n = 52, p = 8
ID: 720, n = 4177, p = 8
OOB prediction error
mtry
method
RFs
RFsextr1
RFsextr5

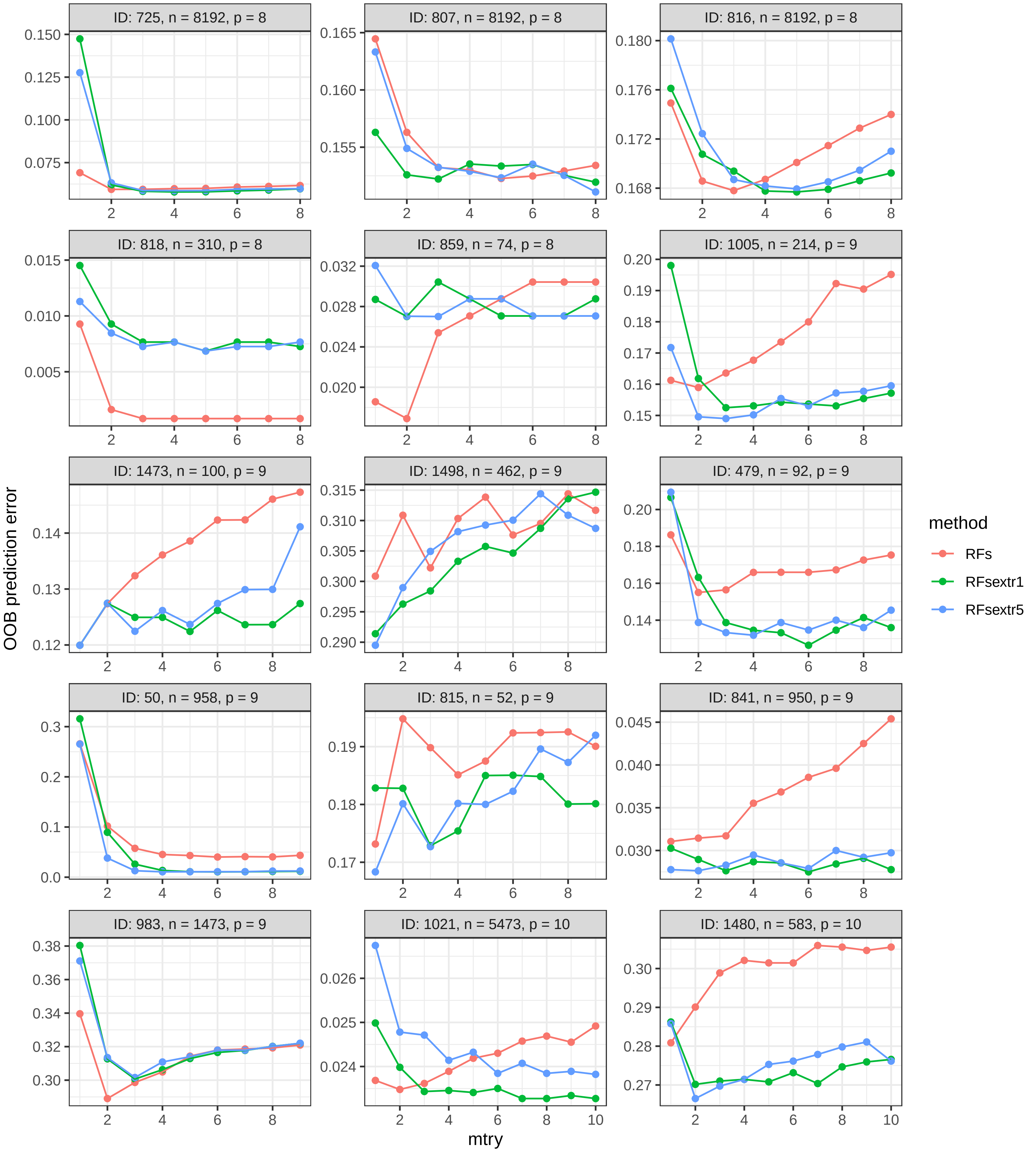
ID: 725, n = 8192, p = 8
ID: 807, n = 8192, p = 8
ID: 816, n = 8192, p = 8
ID: 818, n = 310, p = 8
ID: 859, n = 74, p = 8
ID: 1005, n = 214, p = 9
ID: 1473, n = 100, p = 9
ID: 1498, n = 462, p = 9
ID: 479, n = 92, p = 9
ID: 50, n = 958, p = 9
ID: 815, n = 52, p = 9
ID: 841, n = 950, p = 9
ID: 983, n = 1473, p = 9
ID: 1021, n = 5473, p = 10
ID: 1480, n = 583, p = 10
OOB prediction error
mtry
method
RFs
RFsextr1
RFsextr5

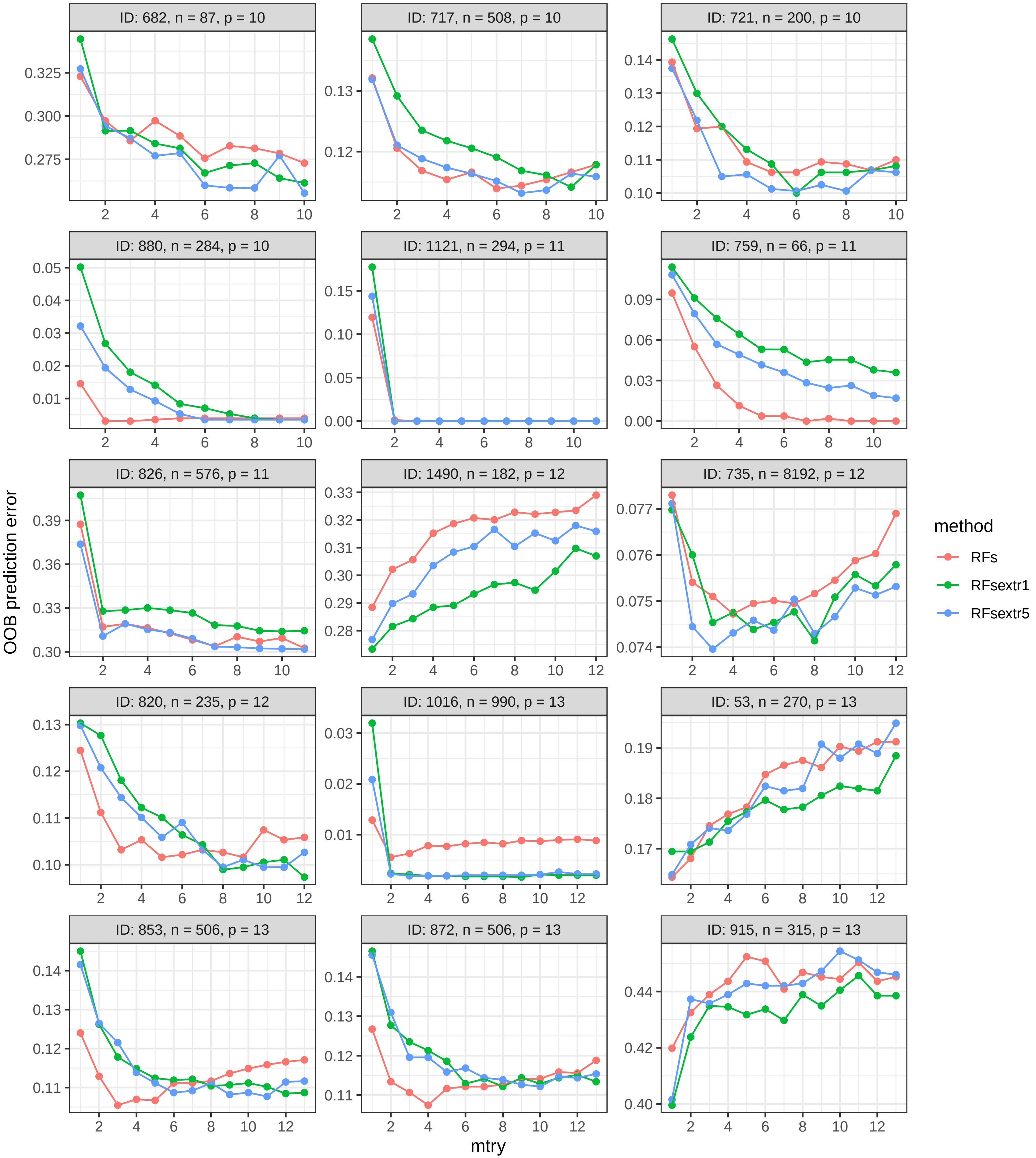
ID: 682, n = 87, p = 10
ID: 717, n = 508, p = 10
ID: 721, n = 200, p = 10
ID: 880, n = 284, p = 10
ID: 1121, n = 294, p = 11
ID: 759, n = 66, p = 11
ID: 826, n = 576, p = 11
ID: 1490, n = 182, p = 12
ID: 735, n = 8192, p = 12
ID: 820, n = 235, p = 12
ID: 1016, n = 990, p = 13
ID: 53, n = 270, p = 13
ID: 853, n = 506, p = 13
ID: 872, n = 506, p = 13
ID: 915, n = 315, p = 13
OOB prediction error
mtry
method
RFs
RFsextr1
RFsextr5

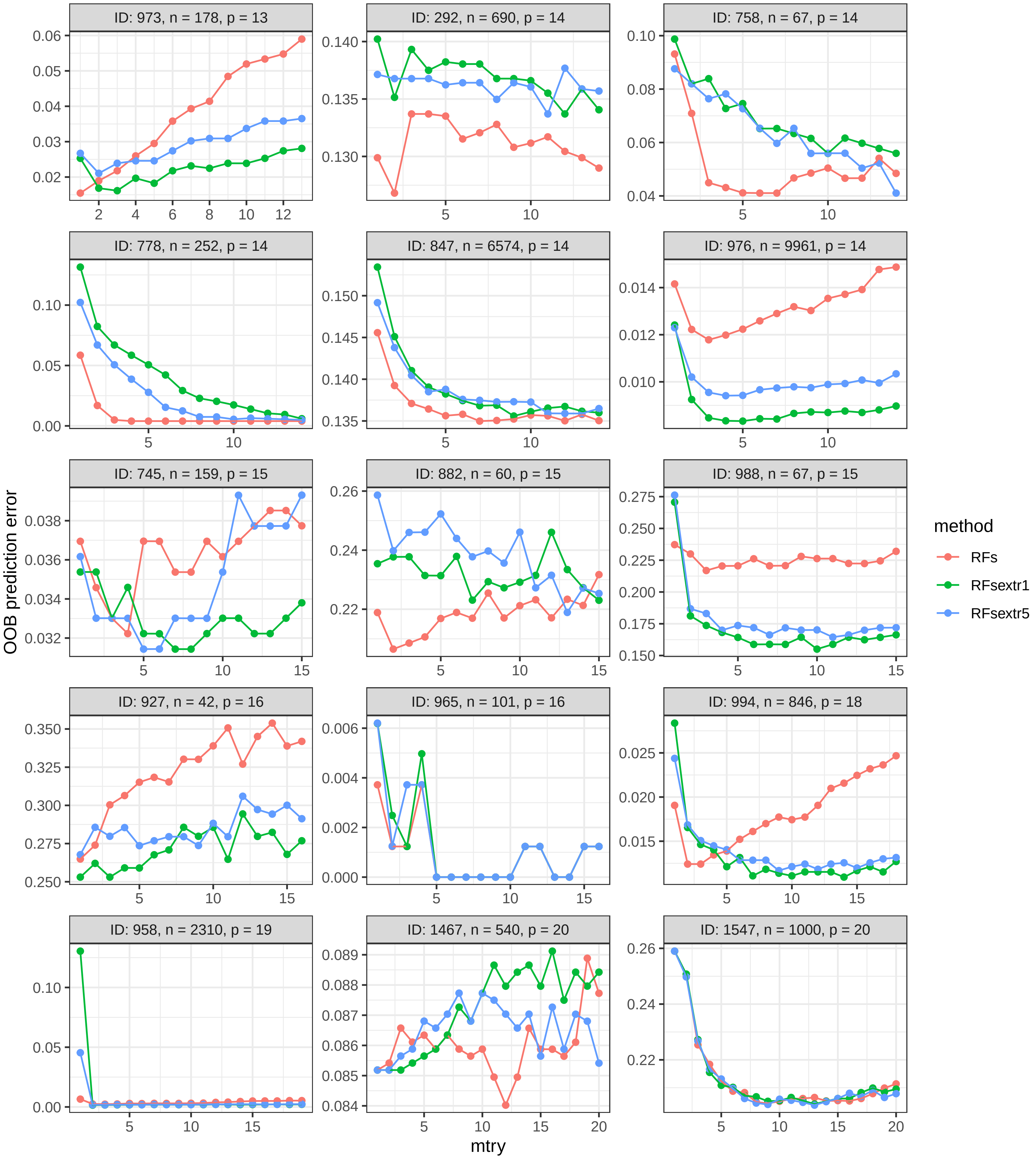

ID: 973, n = 178, p = 13
ID: 292, n = 690, p = 14
ID: 758, n = 67, p = 14
ID: 778, n = 252, p = 14
ID: 847, n = 6574, p = 14
ID: 976, n = 9961, p = 14
ID: 745, n = 159, p = 15
ID: 882, n = 60, p = 15
ID: 988, n = 67, p = 15
ID: 927, n = 42, p = 16
ID: 965, n = 101, p = 16
ID: 994, n = 846, p = 18
ID: 958, n = 2310, p = 19
ID: 1467, n = 540, p = 20
ID: 1547, n = 1000, p = 20
OOB prediction error
mtry
method
RFs
RFsextr1
RFsextr5

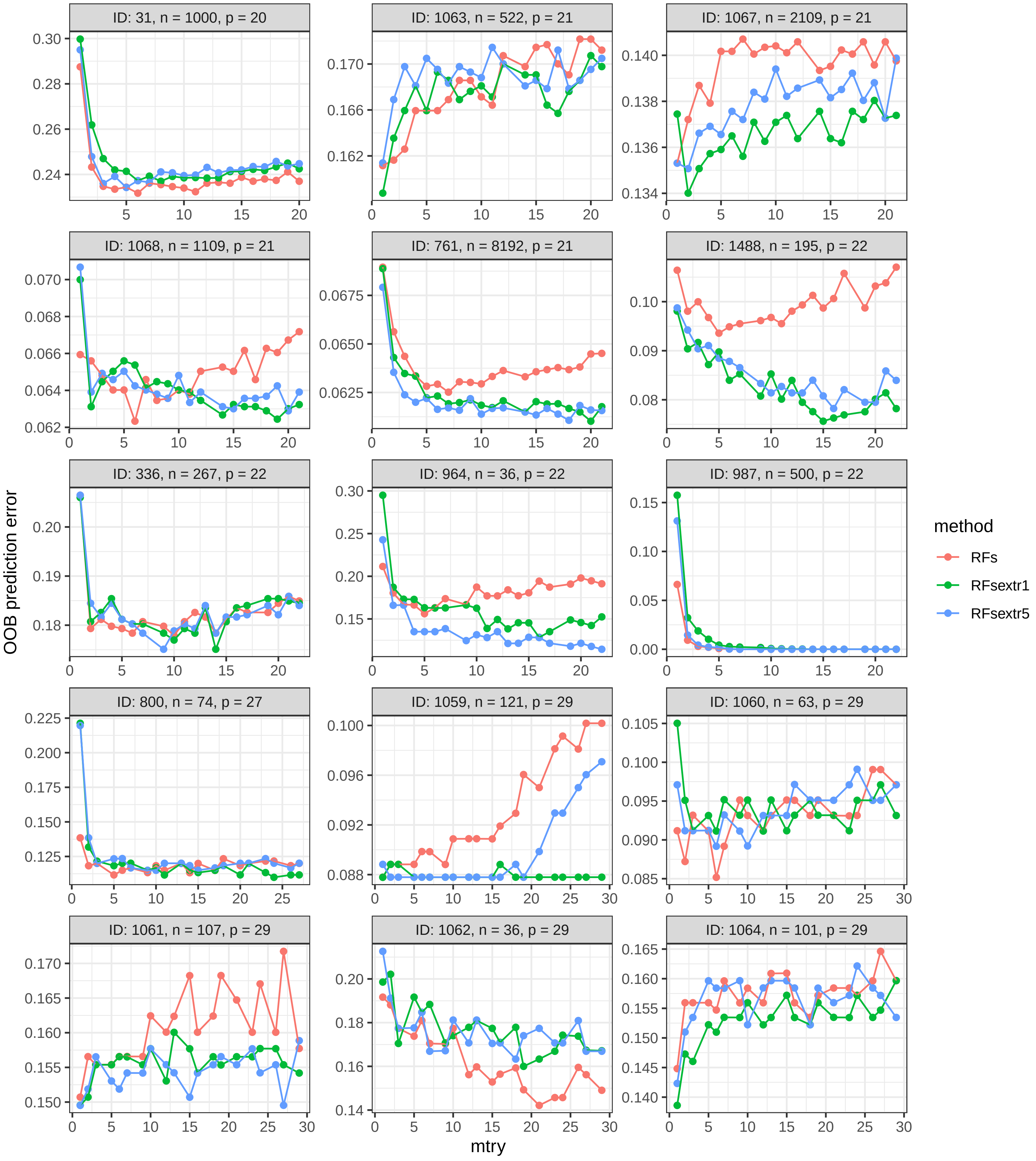
ID: 31, n = 1000, p = 20
ID: 1063, n = 522, p = 21
ID: 1067, n = 2109, p = 21
ID: 1068, n = 1109, p = 21
ID: 761, n = 8192, p = 21
ID: 1488, n = 195, p = 22
ID: 336, n = 267, p = 22
ID: 964, n = 36, p = 22
ID: 987, n = 500, p = 22
ID: 800, n = 74, p = 27
ID: 1059, n = 121, p = 29
ID: 1060, n = 63, p = 29
ID: 1061, n = 107, p = 29
ID: 1062, n = 36, p = 29
ID: 1064, n = 101, p = 29
OOB prediction error
mtry
method
RFs
RFsextr1
RFsextr5

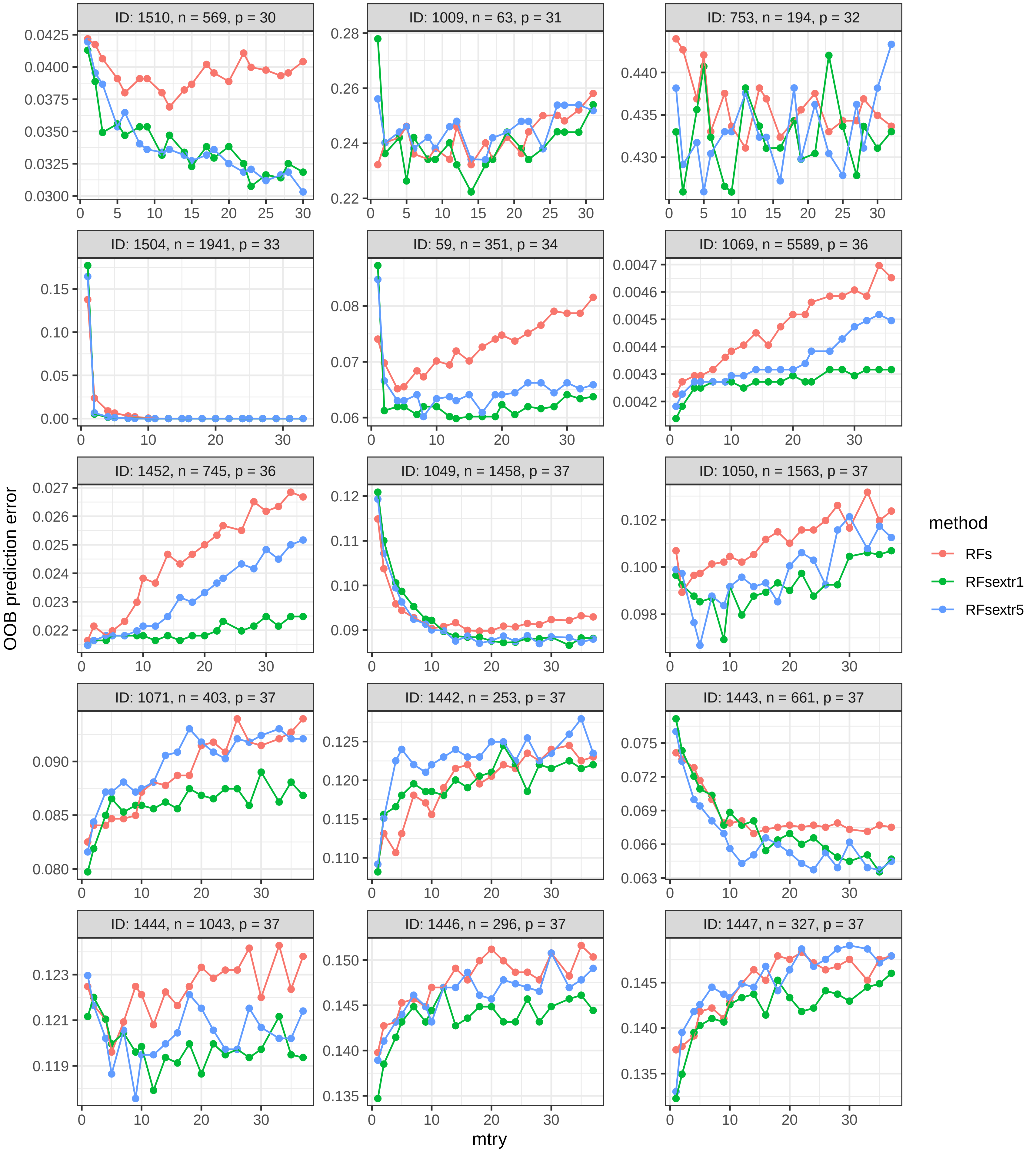
ID: 1510, n = 569, p = 30
ID: 1009, n = 63, p = 31
ID: 753, n = 194, p = 32
ID: 1504, n = 1941, p = 33
ID: 59, n = 351, p = 34
ID: 1069, n = 5589, p = 36
ID: 1452, n = 745, p = 36
ID: 1049, n = 1458, p = 37
ID: 1050, n = 1563, p = 37
ID: 1071, n = 403, p = 37
ID: 1442, n = 253, p = 37
ID: 1443, n = 661, p = 37
ID: 1444, n = 1043, p = 37
ID: 1446, n = 296, p = 37
ID: 1447, n = 327, p = 37
OOB prediction error
mtry
method
RFs
RFsextr1
RFsextr5

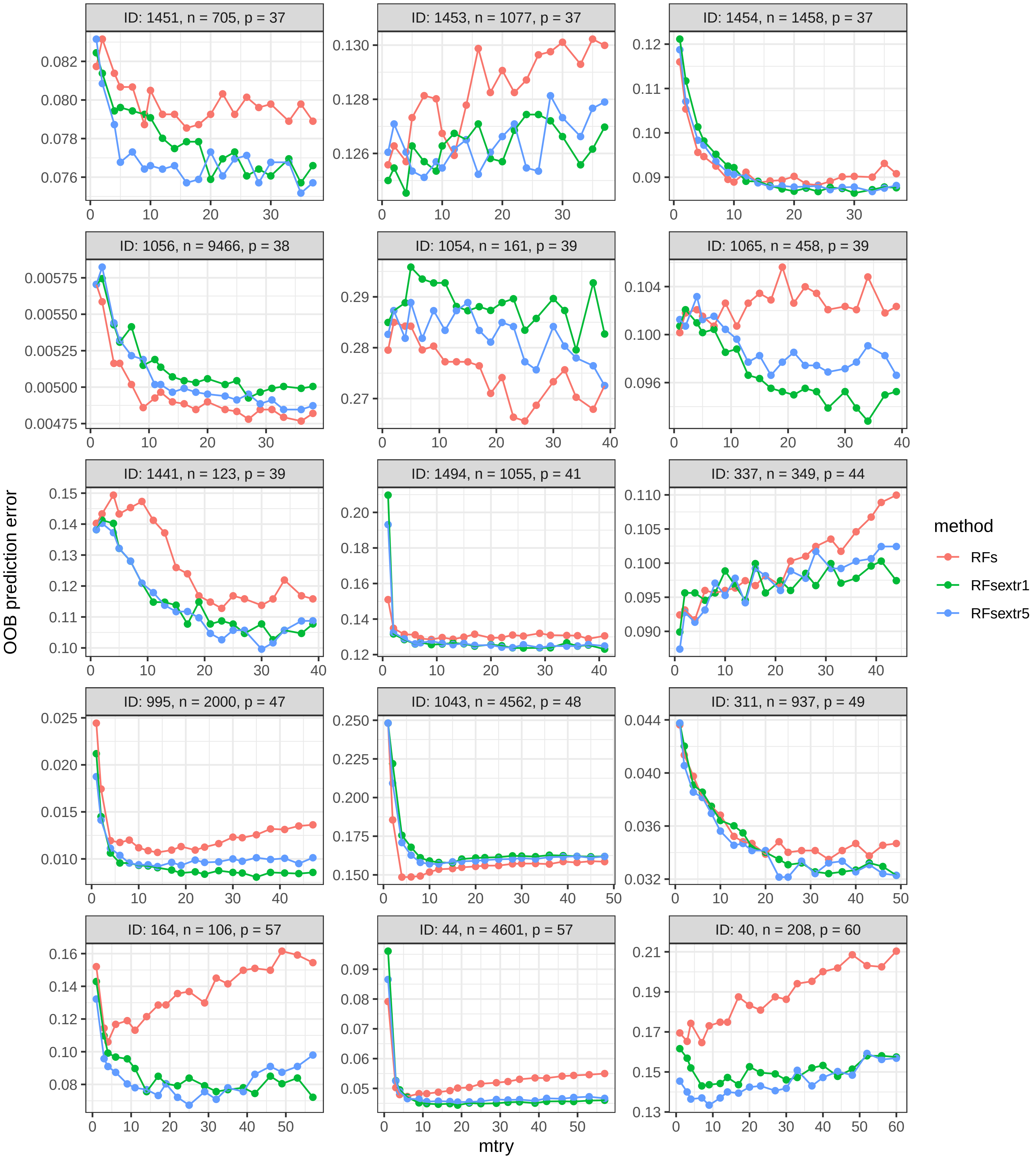
ID: 1451, n = 705, p = 37
ID: 1453, n = 1077, p = 37
ID: 1454, n = 1458, p = 37
ID: 1056, n = 9466, p = 38
ID: 1054, n = 161, p = 39
ID: 1065, n = 458, p = 39
ID: 1441, n = 123, p = 39
ID: 1494, n = 1055, p = 41
ID: 337, n = 349, p = 44
ID: 995, n = 2000, p = 47
ID: 1043, n = 4562, p = 48
ID: 311, n = 937, p = 49
ID: 164, n = 106, p = 57
ID: 44, n = 4601, p = 57
ID: 40, n = 208, p = 60
OOB prediction error
mtry
method
RFs
RFsextr1
RFsextr5

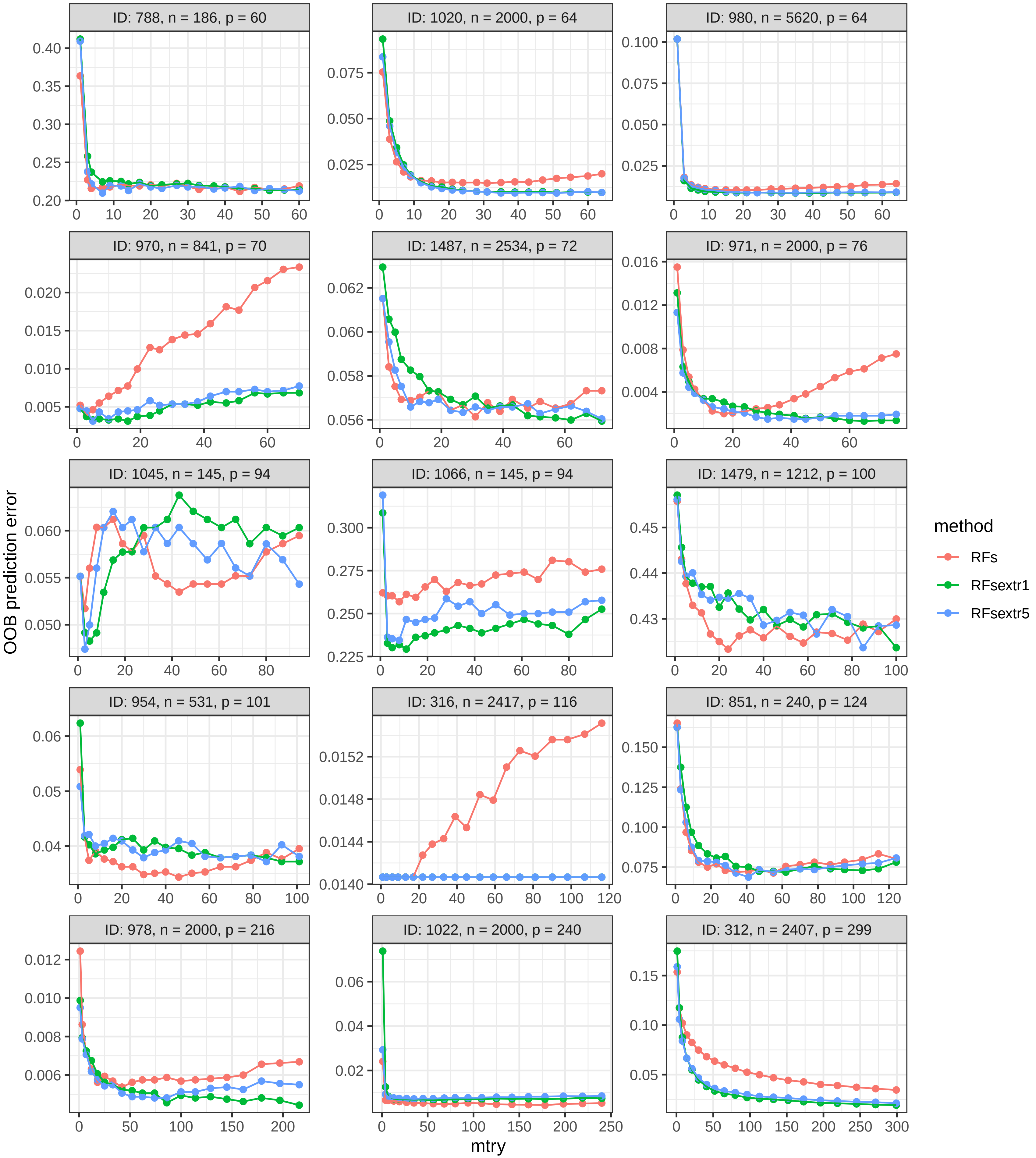
ID: 788, n = 186, p = 60
ID: 1020, n = 2000, p = 64
ID: 980, n = 5620, p = 64
ID: 970, n = 841, p = 70
ID: 1487, n = 2534, p = 72
ID: 971, n = 2000, p = 76
ID: 1045, n = 145, p = 94
ID: 1066, n = 145, p = 94
ID: 1479, n = 1212, p = 100
ID: 954, n = 531, p = 101
ID: 316, n = 2417, p = 116
ID: 851, n = 240, p = 124
ID: 978, n = 2000, p = 216
ID: 1022, n = 2000, p = 240
ID: 312, n = 2407, p = 299
OOB prediction error
mtry
method
RFs
RFsextr1
RFsextr5
