# Supplementary figures and images for: Diversity Forests: Using Split Sampling to Enable Innovative Complex Split Procedures in Random Forests
Source: SN Comput Sci. 2021 Oct 21;3(1):1. doi: 10.1007/s42979-021-00920-1 (PMC8533673; doi:10.1007/s42979-021-00920-1)

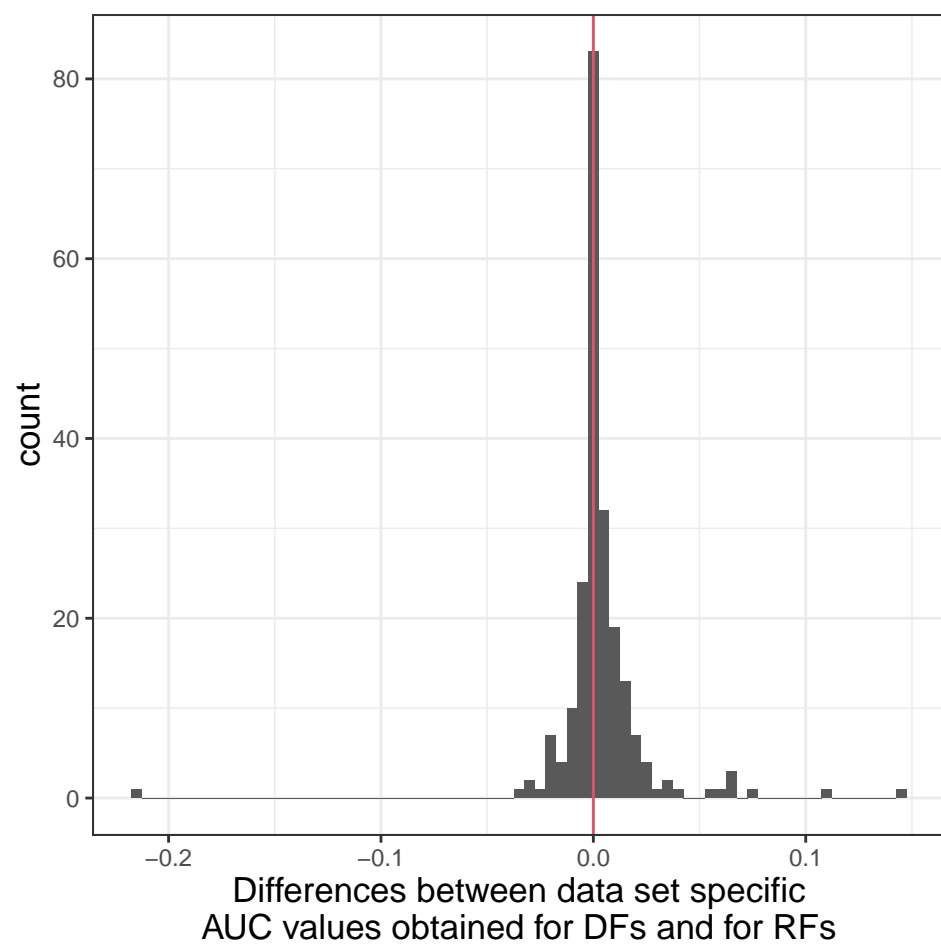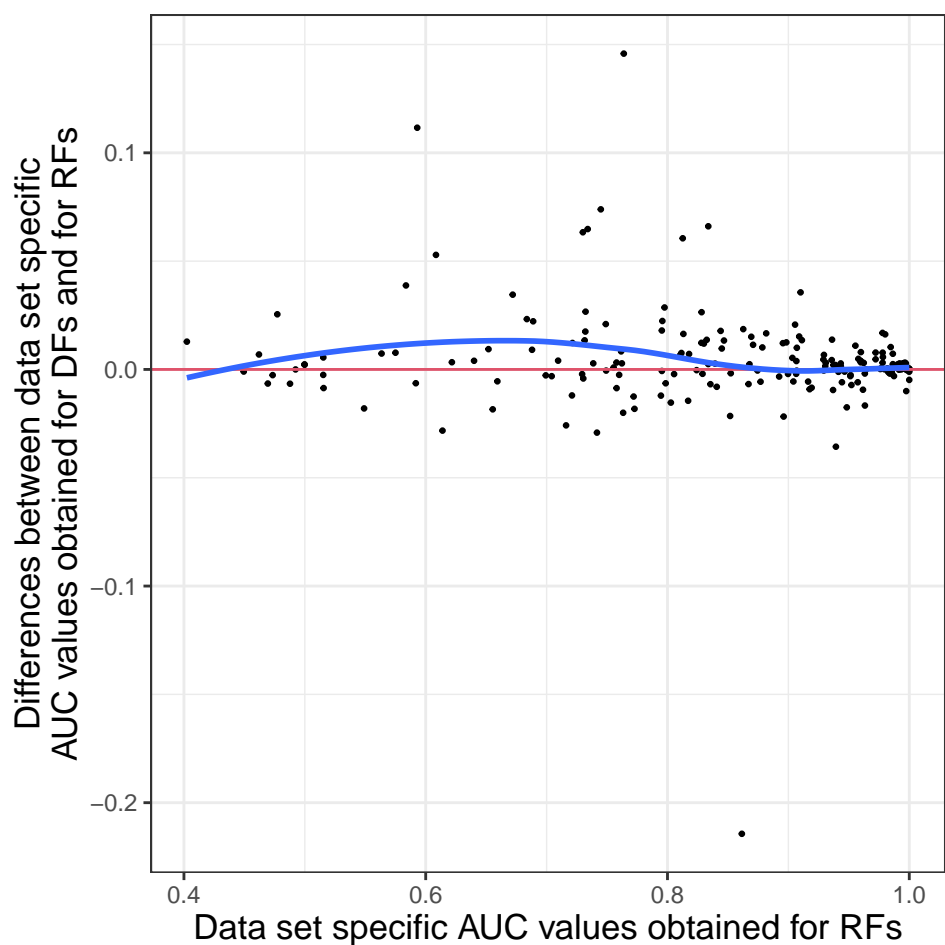

Supplement: Supplementary file 2 — Supplementary file1 (ZIP 108032 KB) [file 42979_2021_920_MOESM2_ESM.zip › Online_Resource_2/Results/Figures/CompRF_auc.pdf]

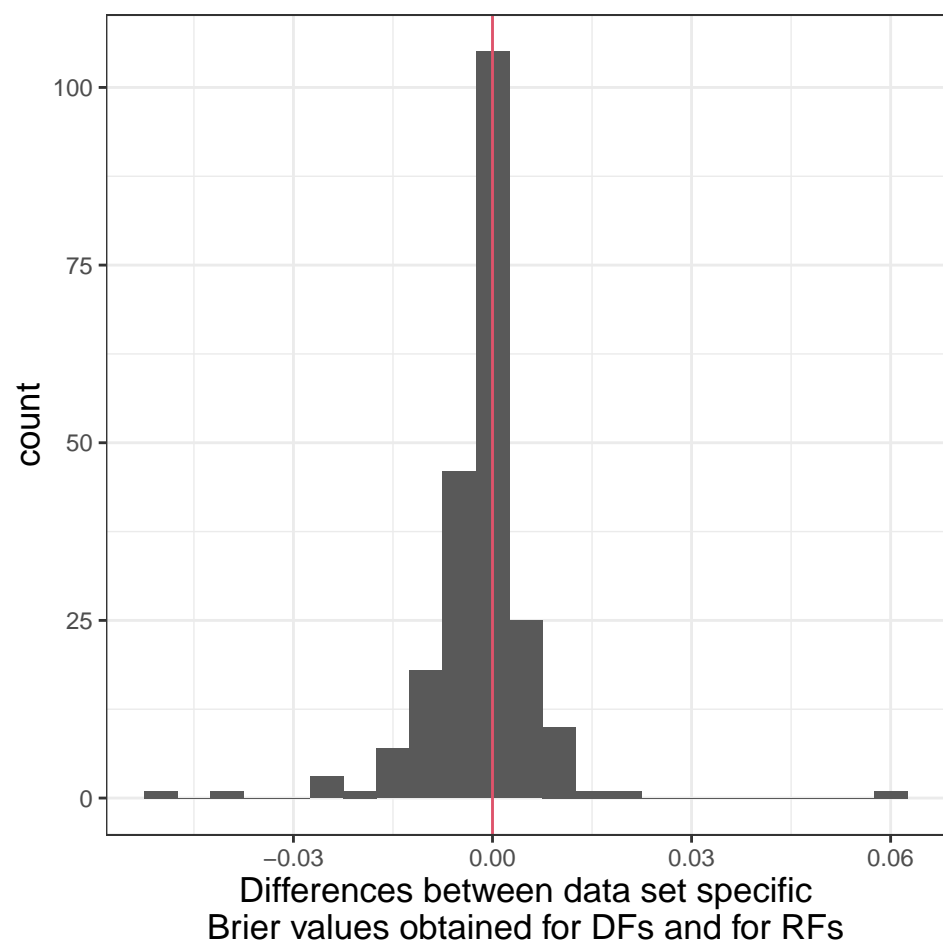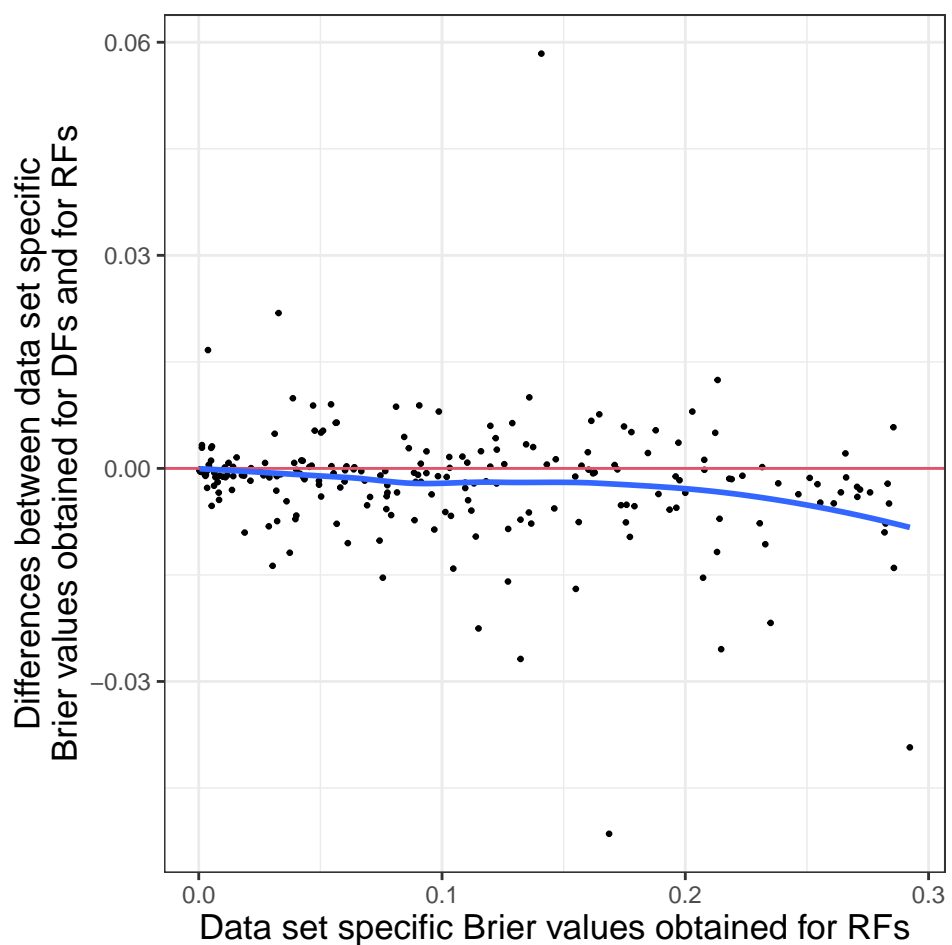

Supplement: Supplementary file 2 — Supplementary file1 (ZIP 108032 KB) [file 42979_2021_920_MOESM2_ESM.zip › Online_Resource_2/Results/Figures/CompRF_brier.pdf]

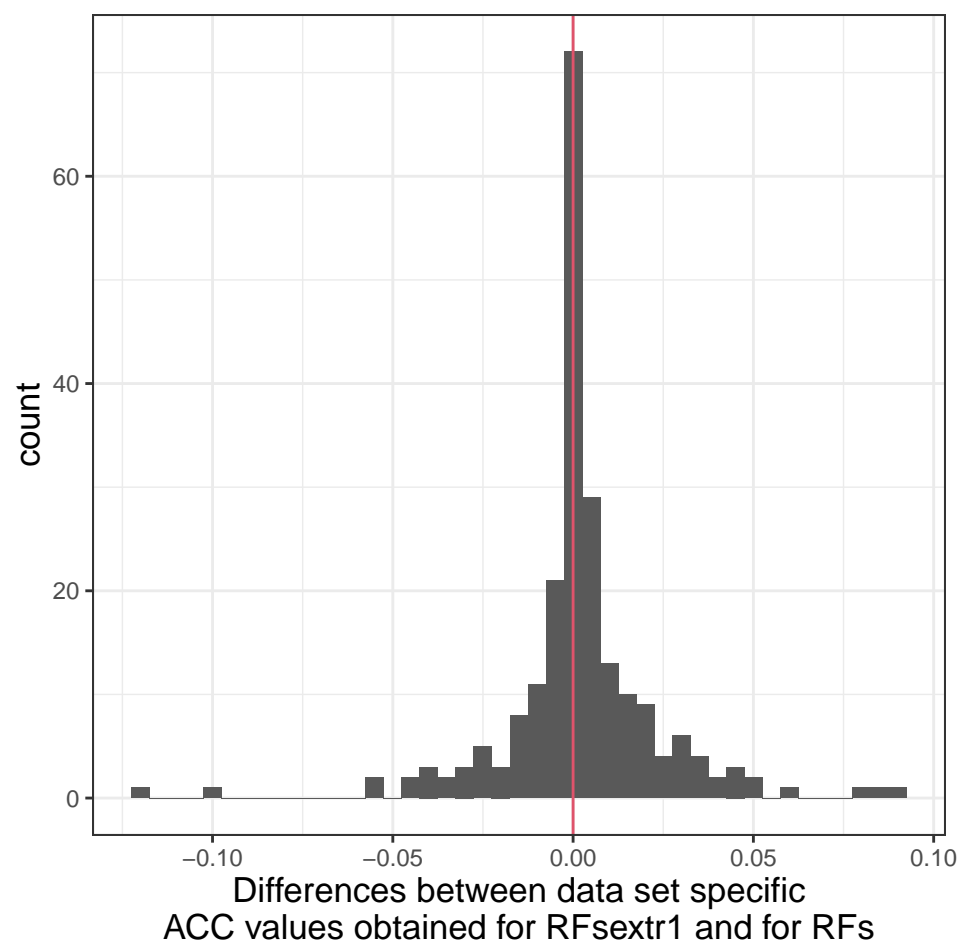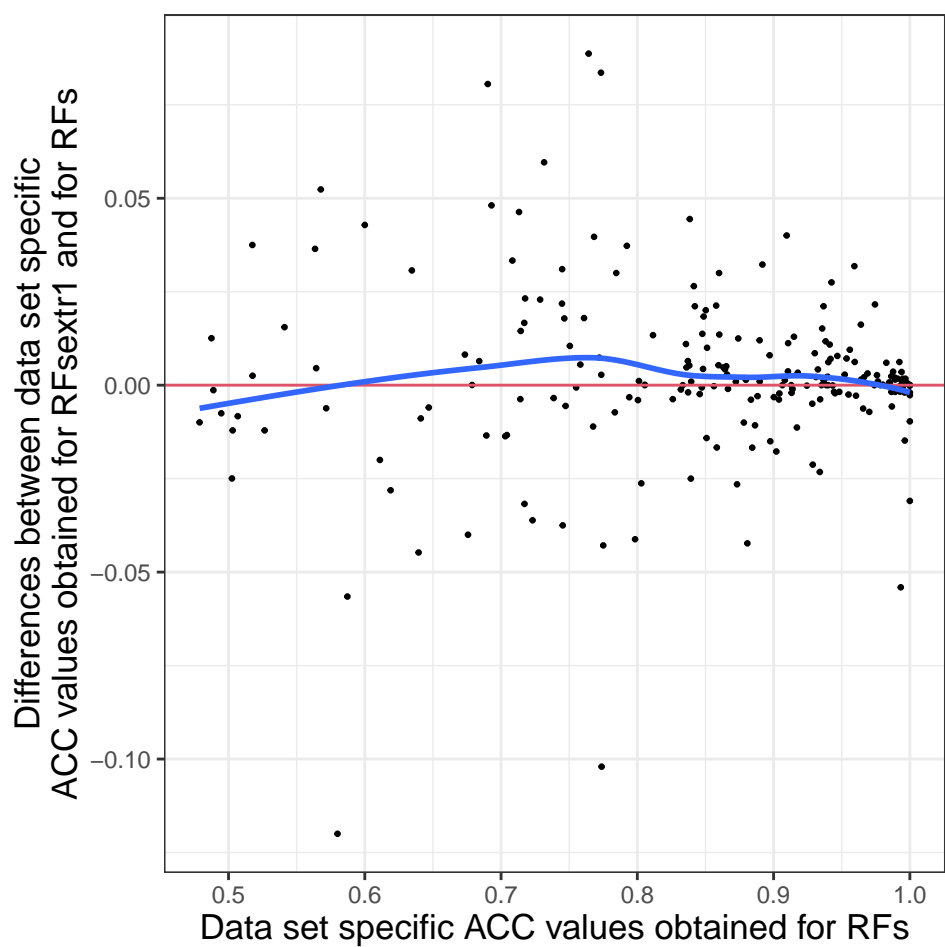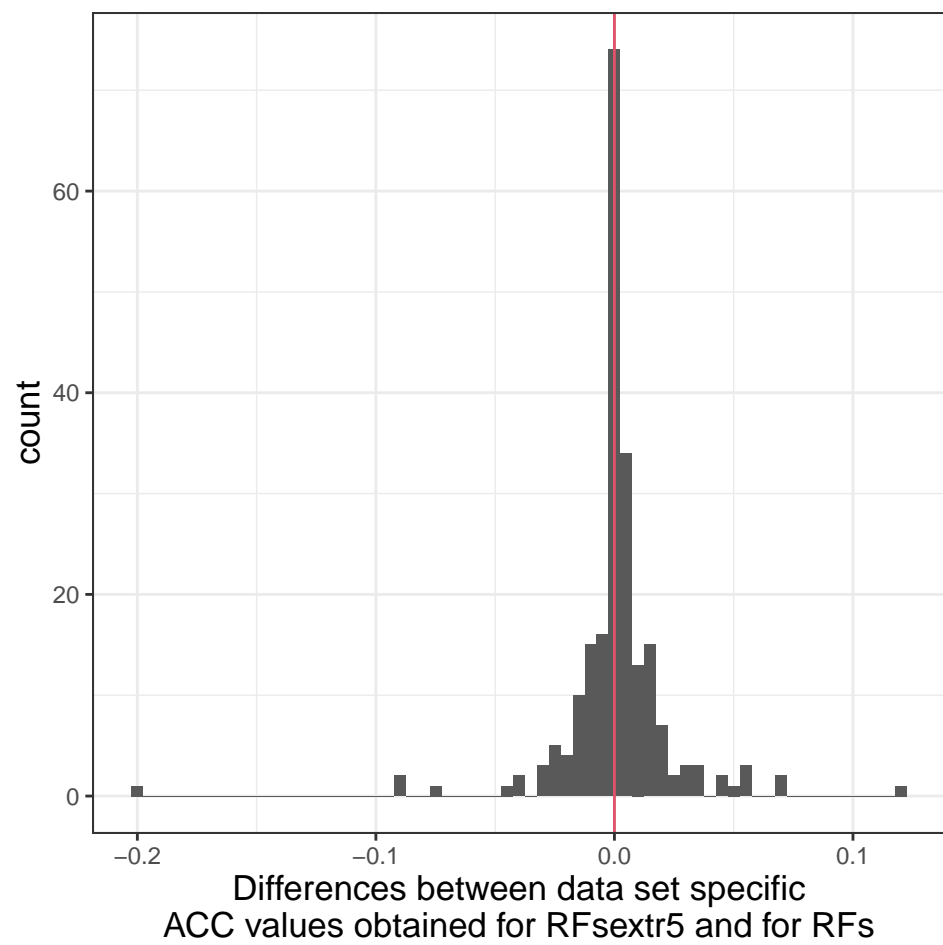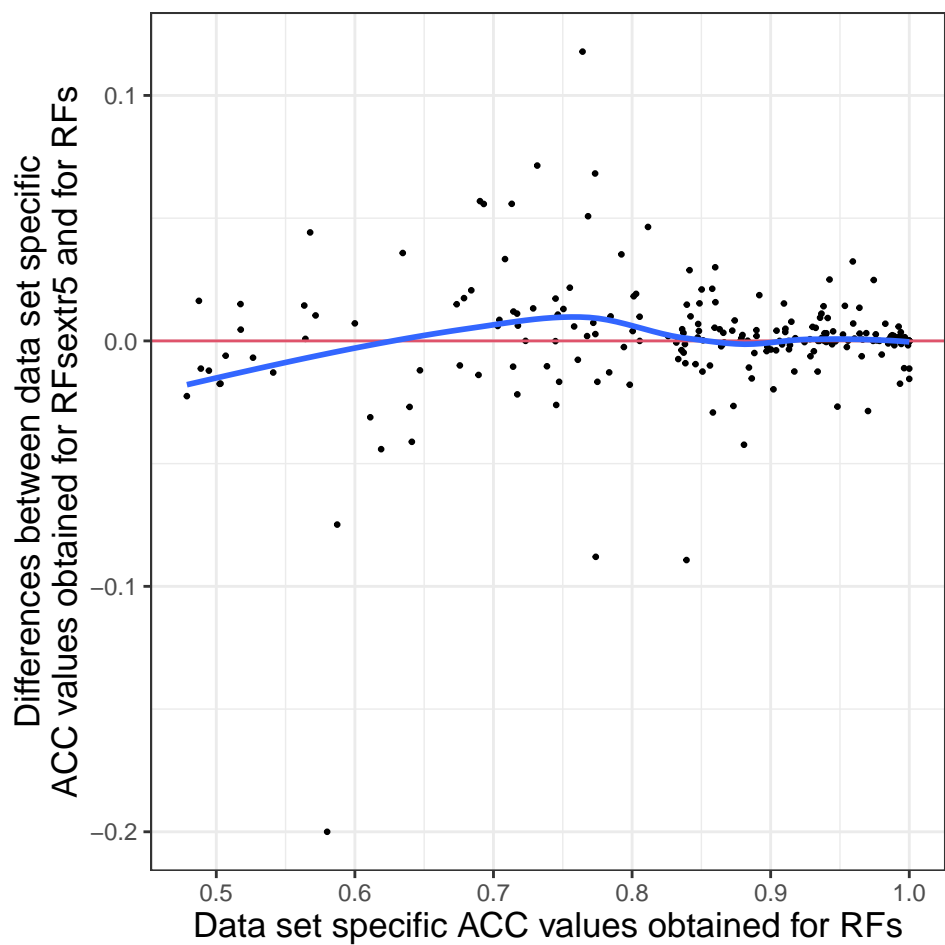

Supplement: Supplementary file 2 — Supplementary file1 (ZIP 108032 KB) [file 42979_2021_920_MOESM2_ESM.zip › Online_Resource_2/Results/Figures/CompRF_Supplement.pdf]

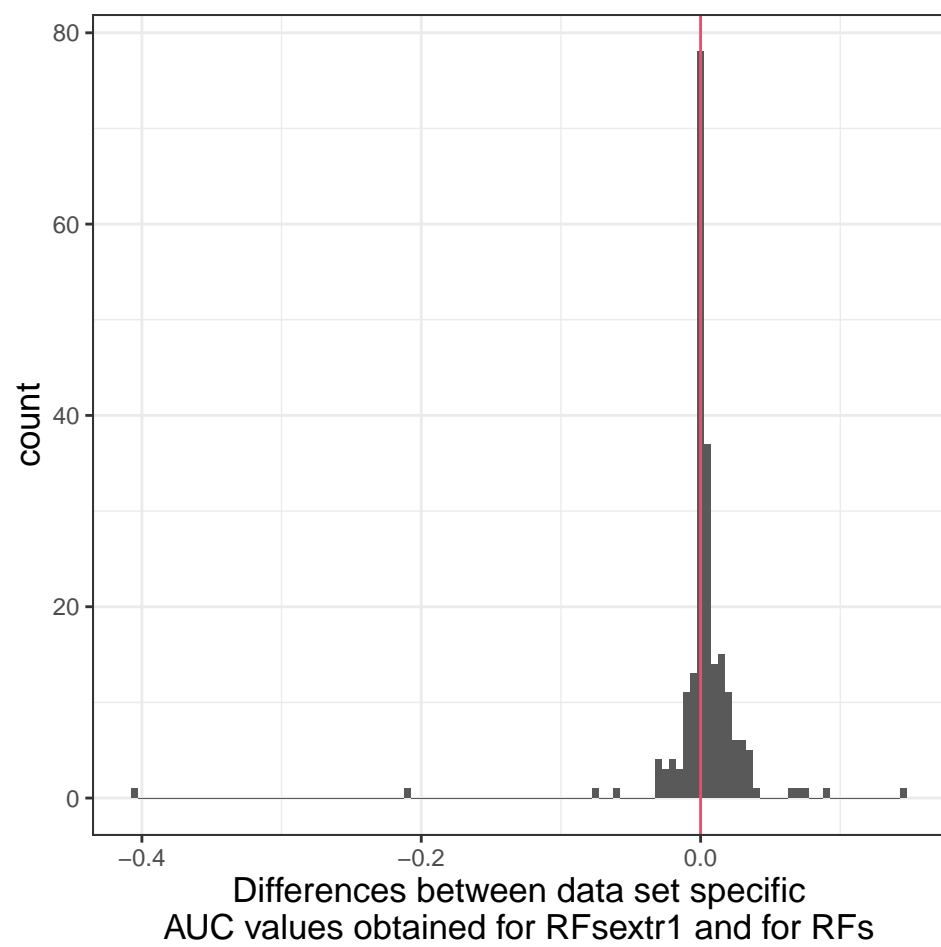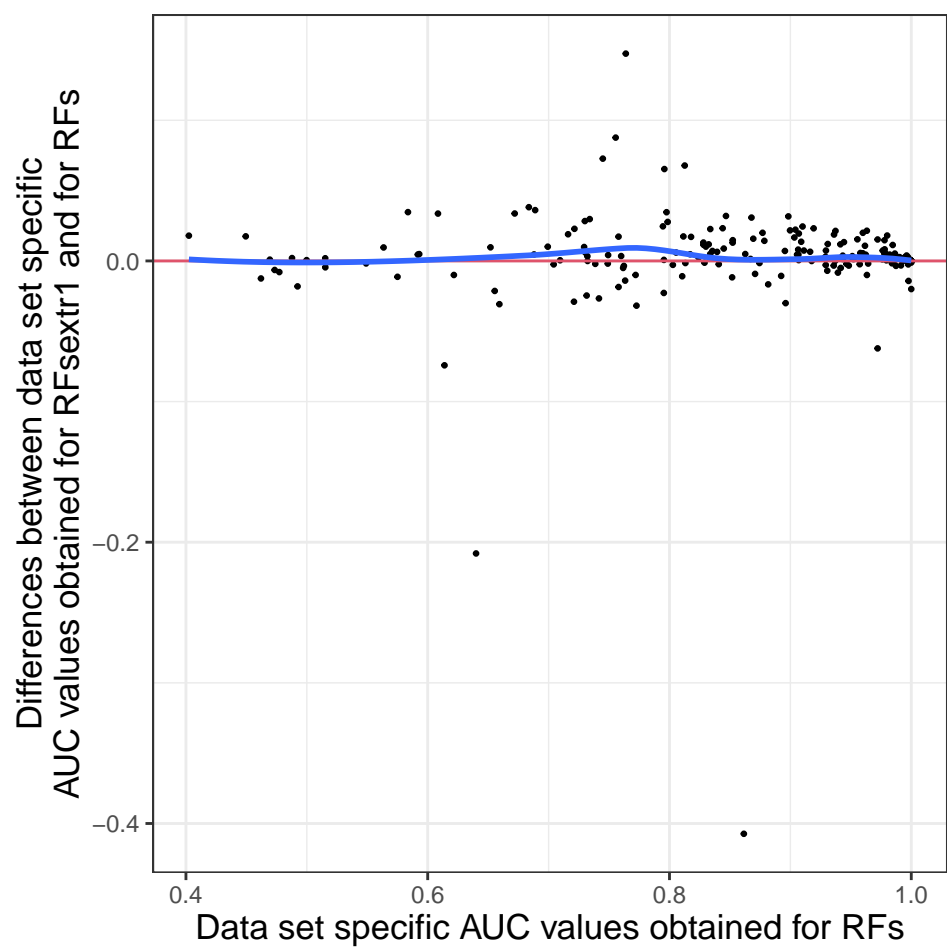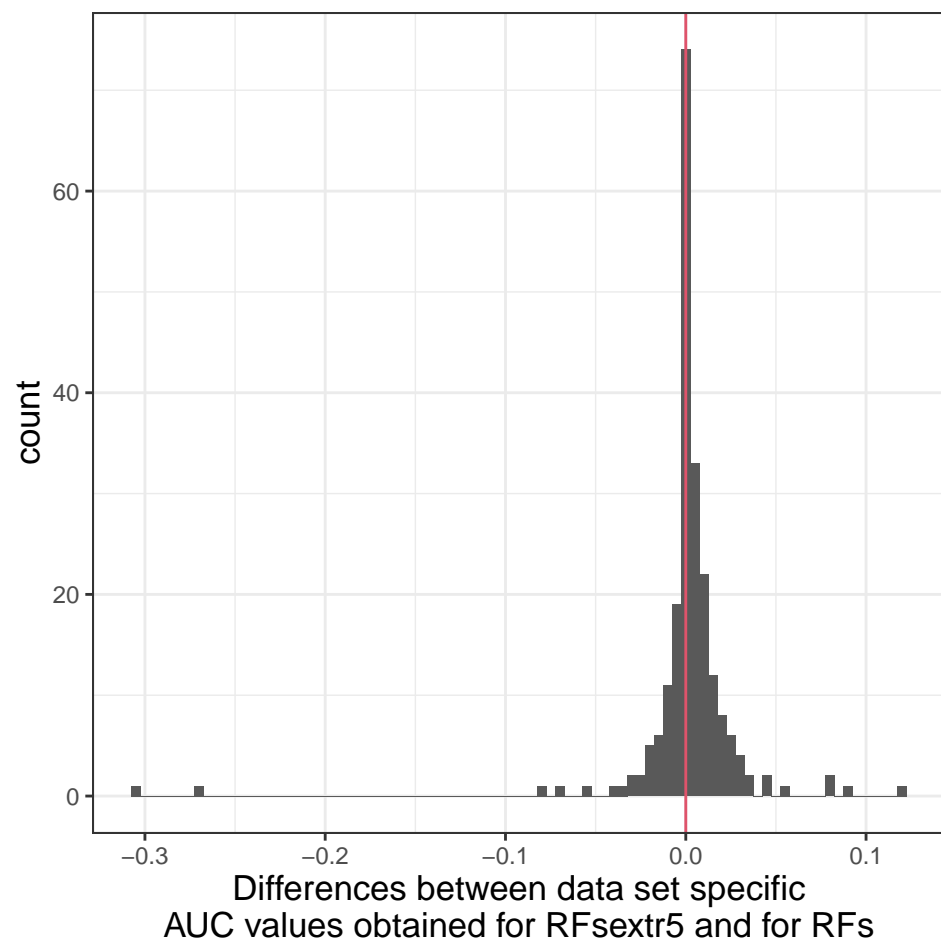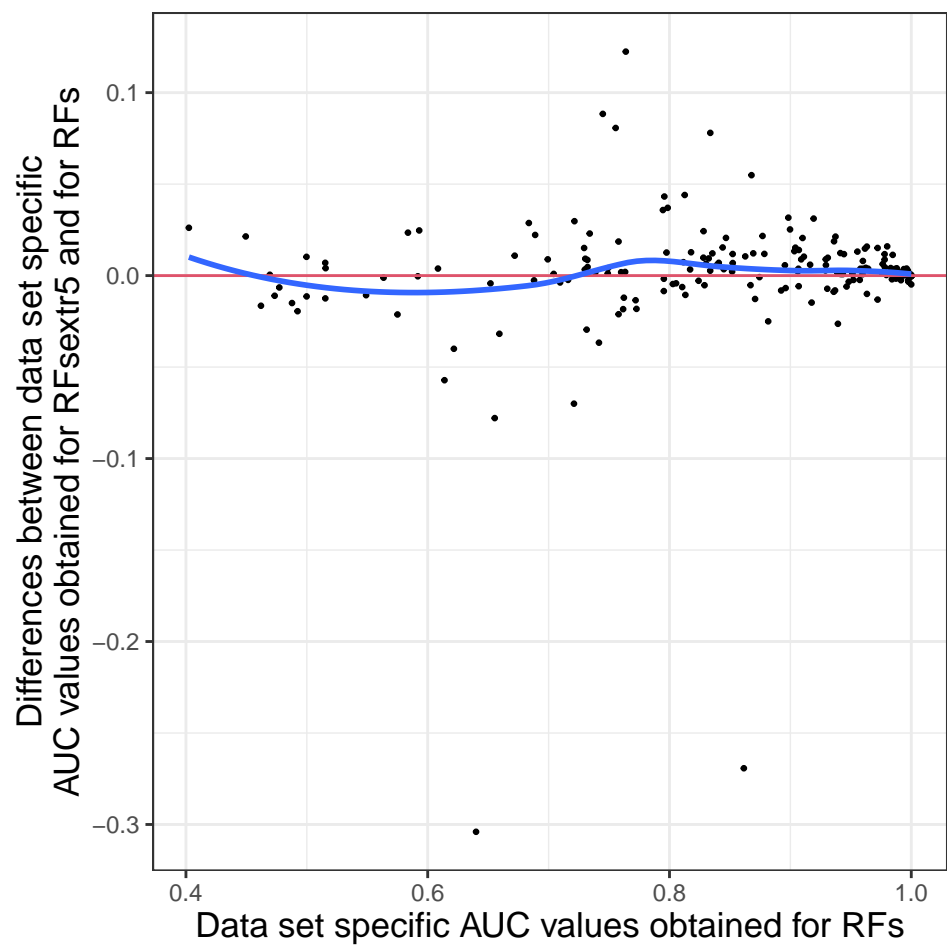

Supplement: Supplementary file 2 — Supplementary file1 (ZIP 108032 KB) [file 42979_2021_920_MOESM2_ESM.zip › Online_Resource_2/Results/Figures/CompRF_Supplement_auc.pdf]

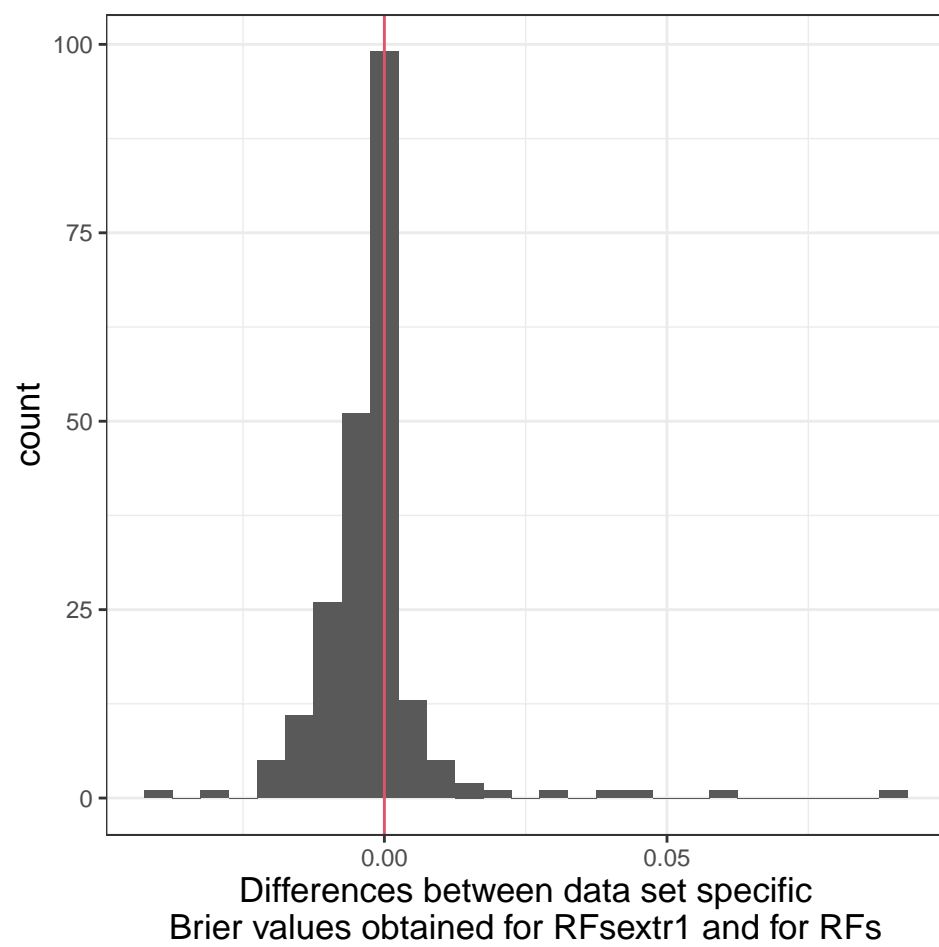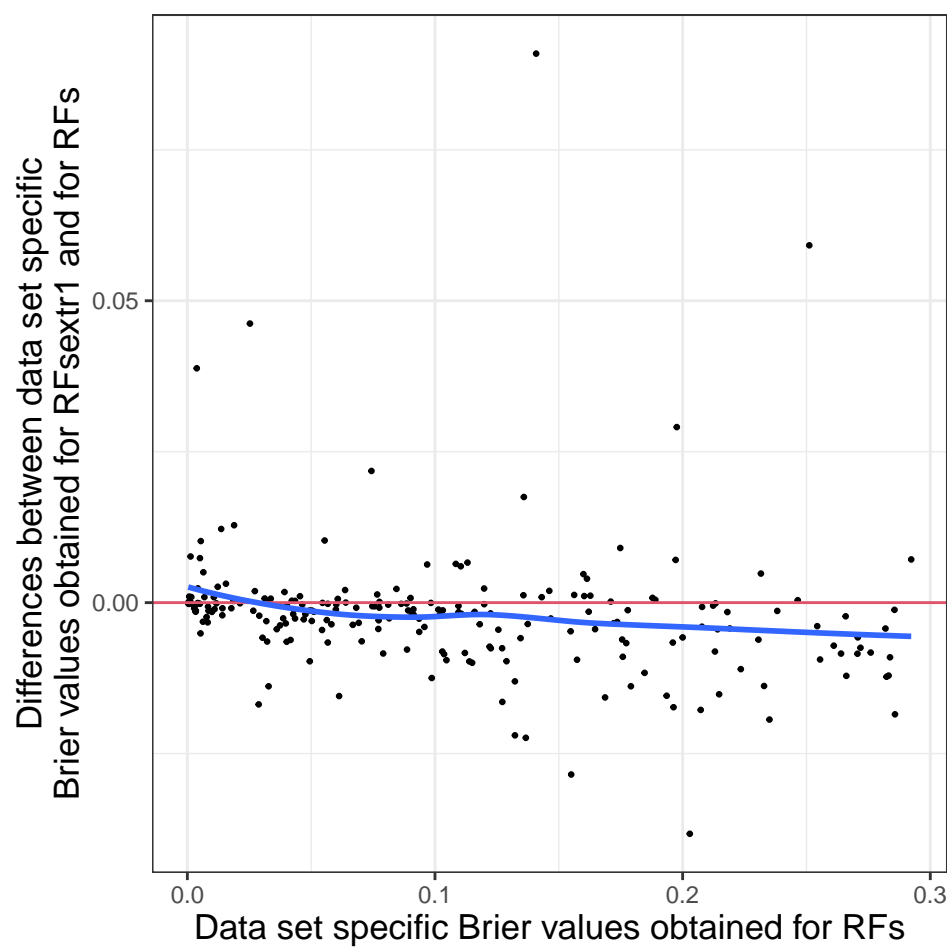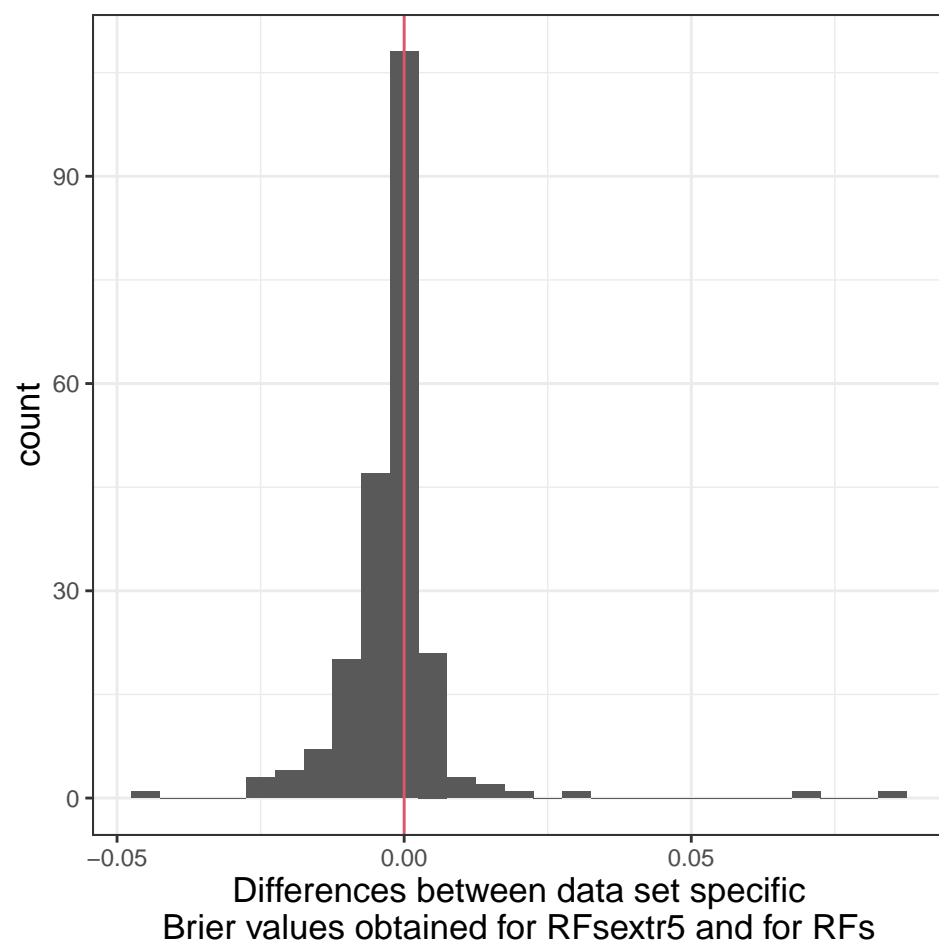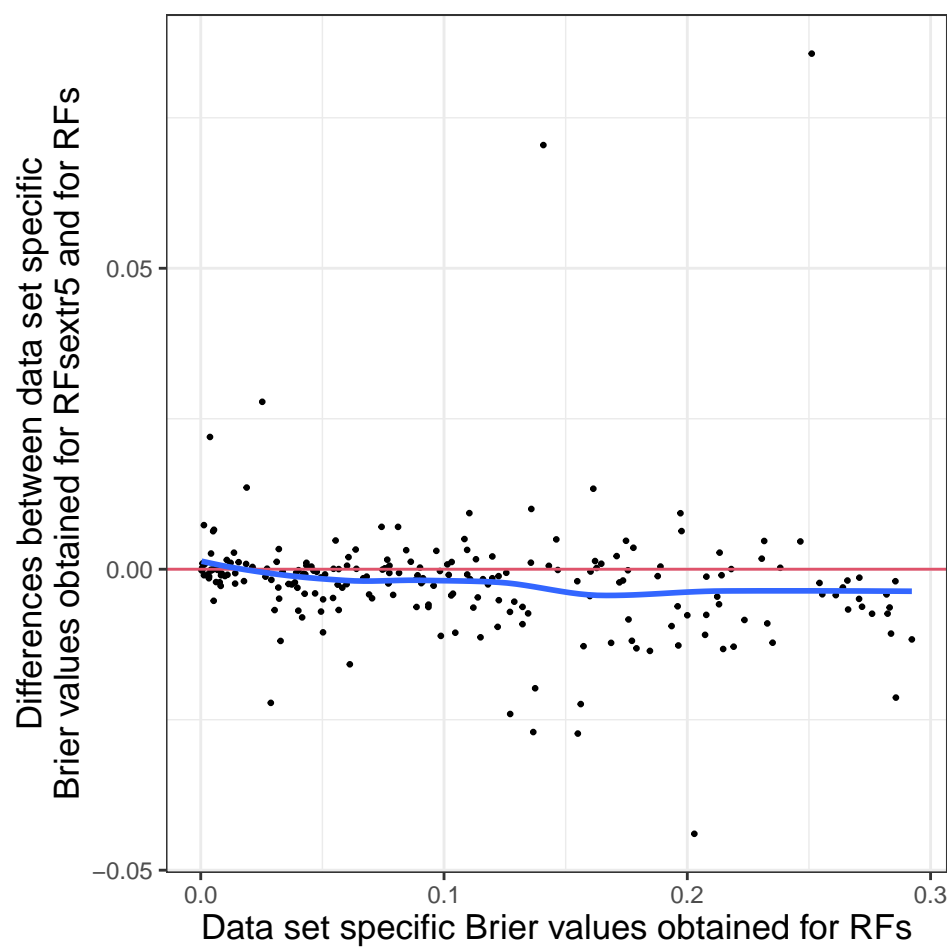

Supplement: Supplementary file 2 — Supplementary file1 (ZIP 108032 KB) [file 42979_2021_920_MOESM2_ESM.zip › Online_Resource_2/Results/Figures/CompRF_Supplement_brier.pdf]

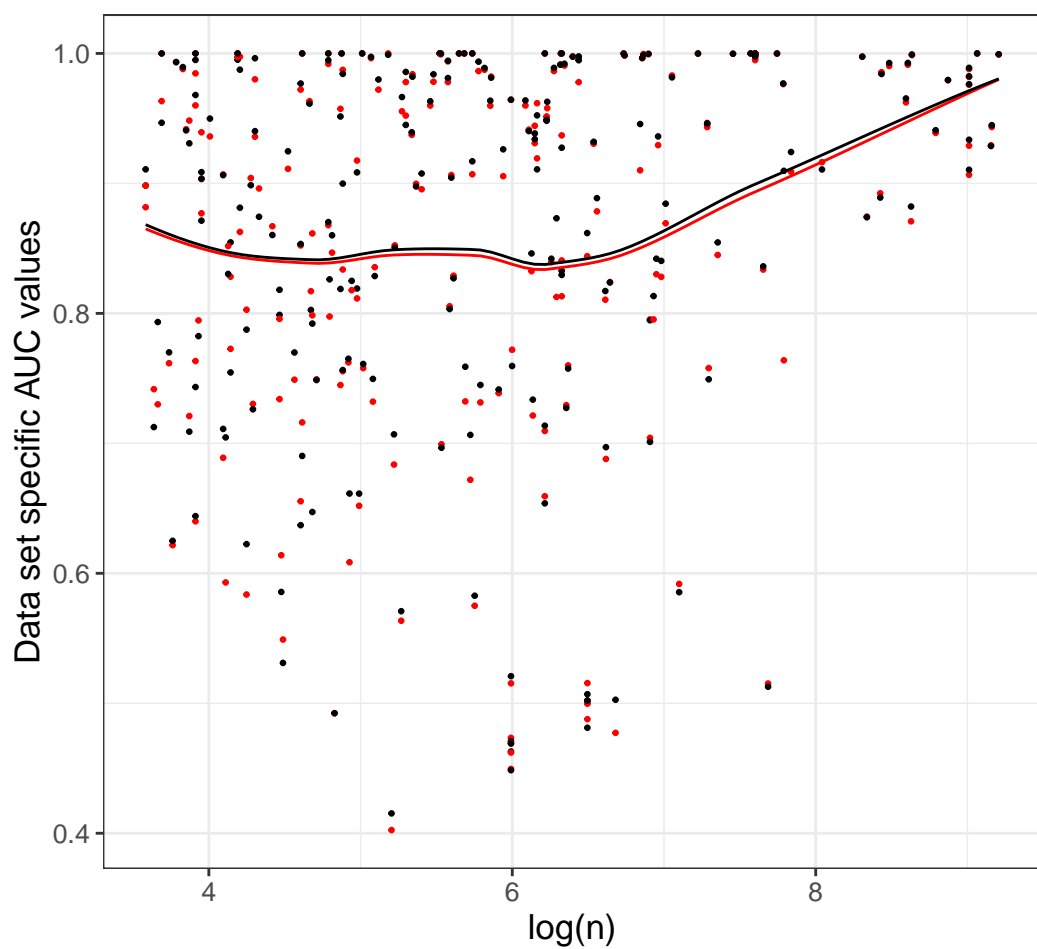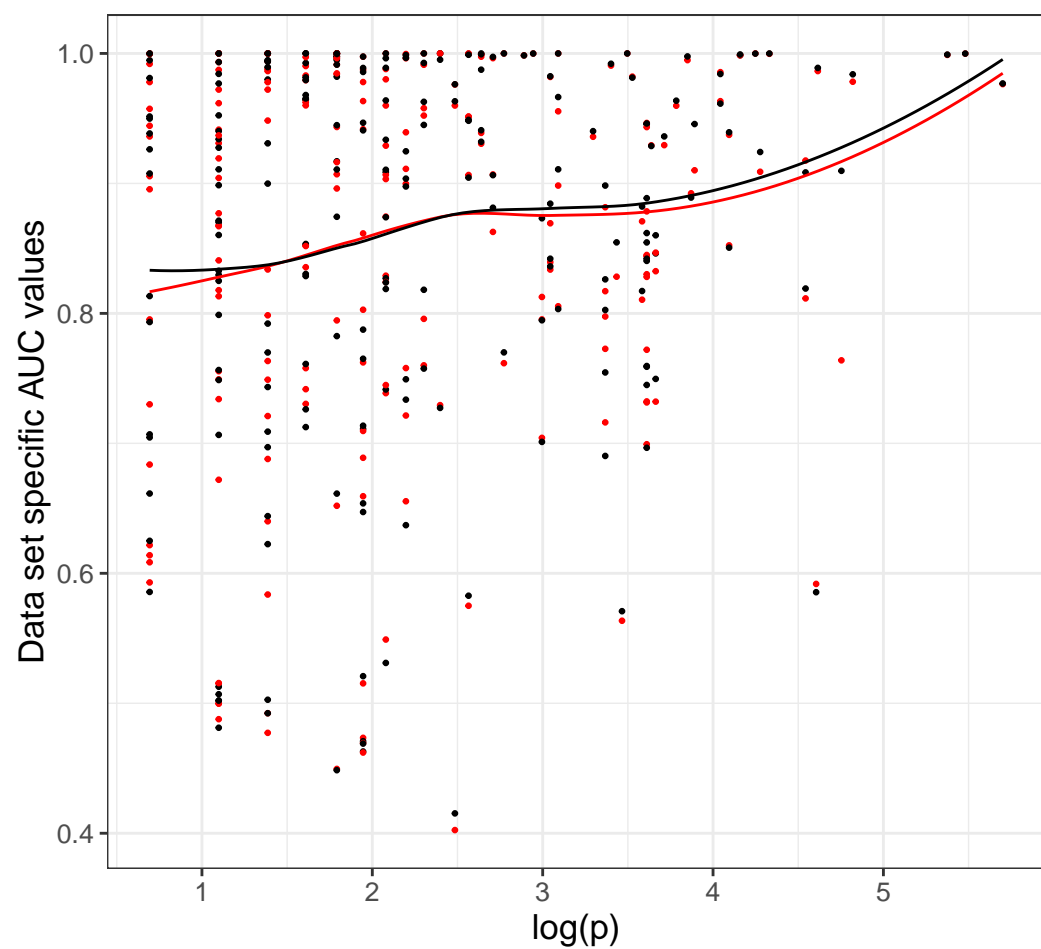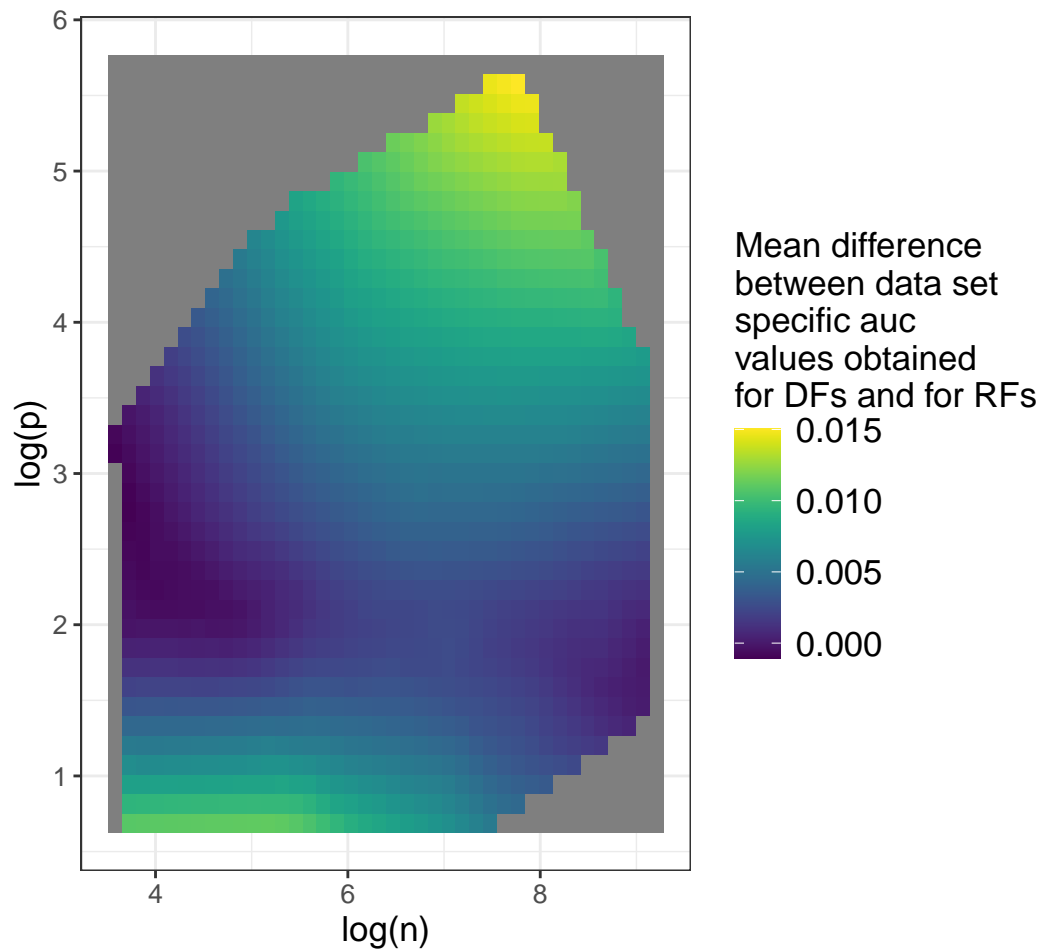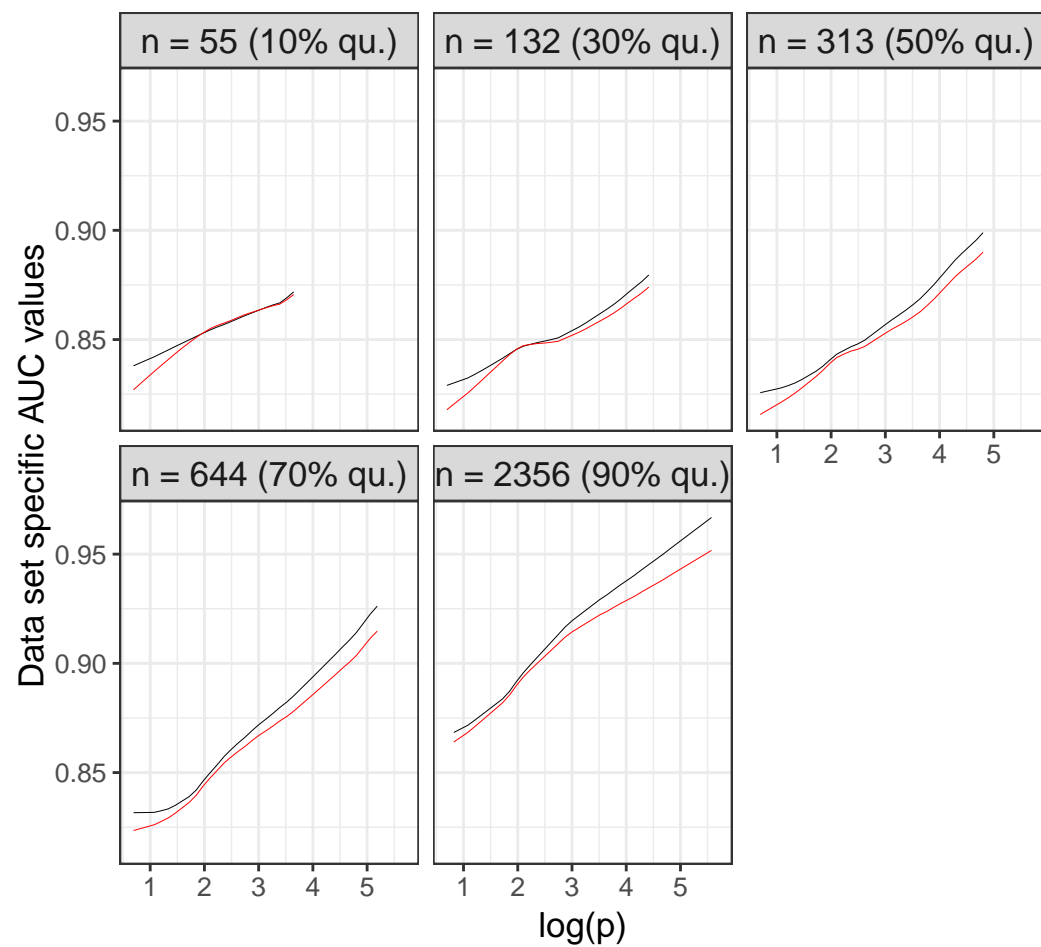

Supplement: Supplementary file 2 — Supplementary file1 (ZIP 108032 KB) [file 42979_2021_920_MOESM2_ESM.zip › Online_Resource_2/Results/Figures/DataCharacteristics_auc.pdf]

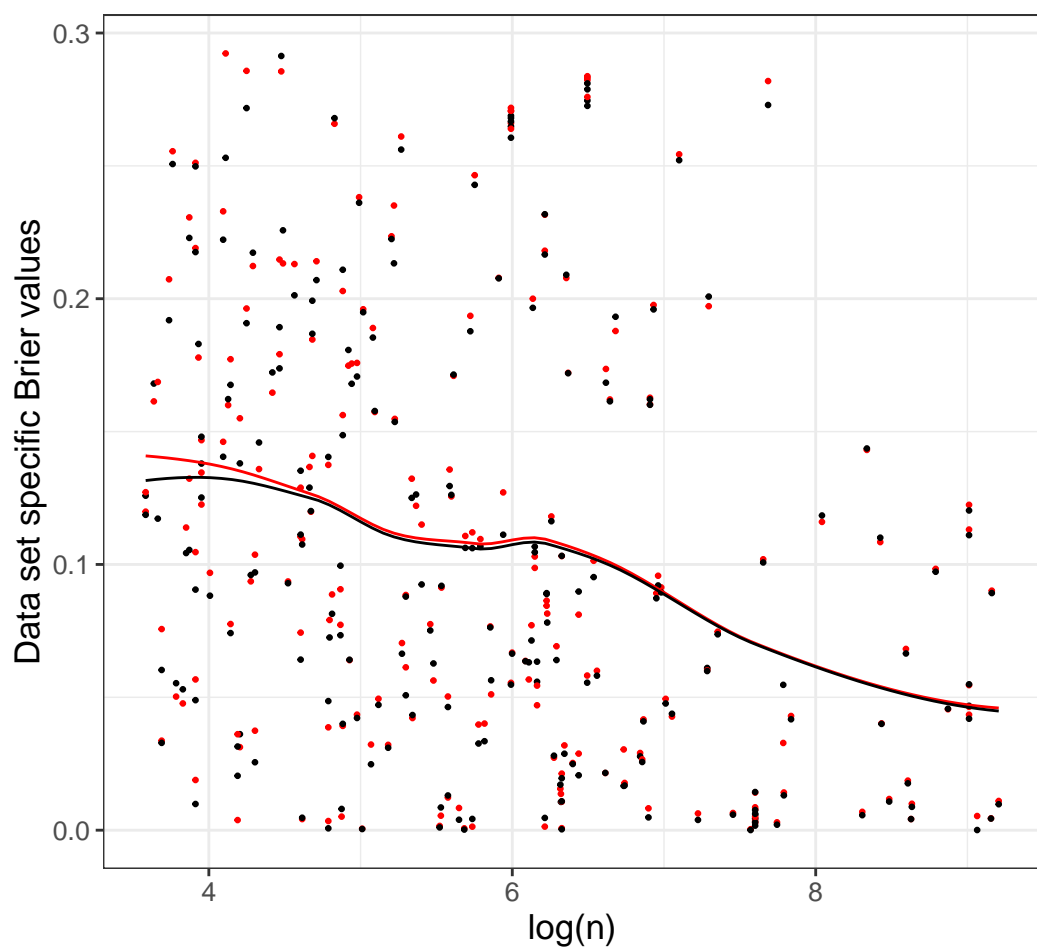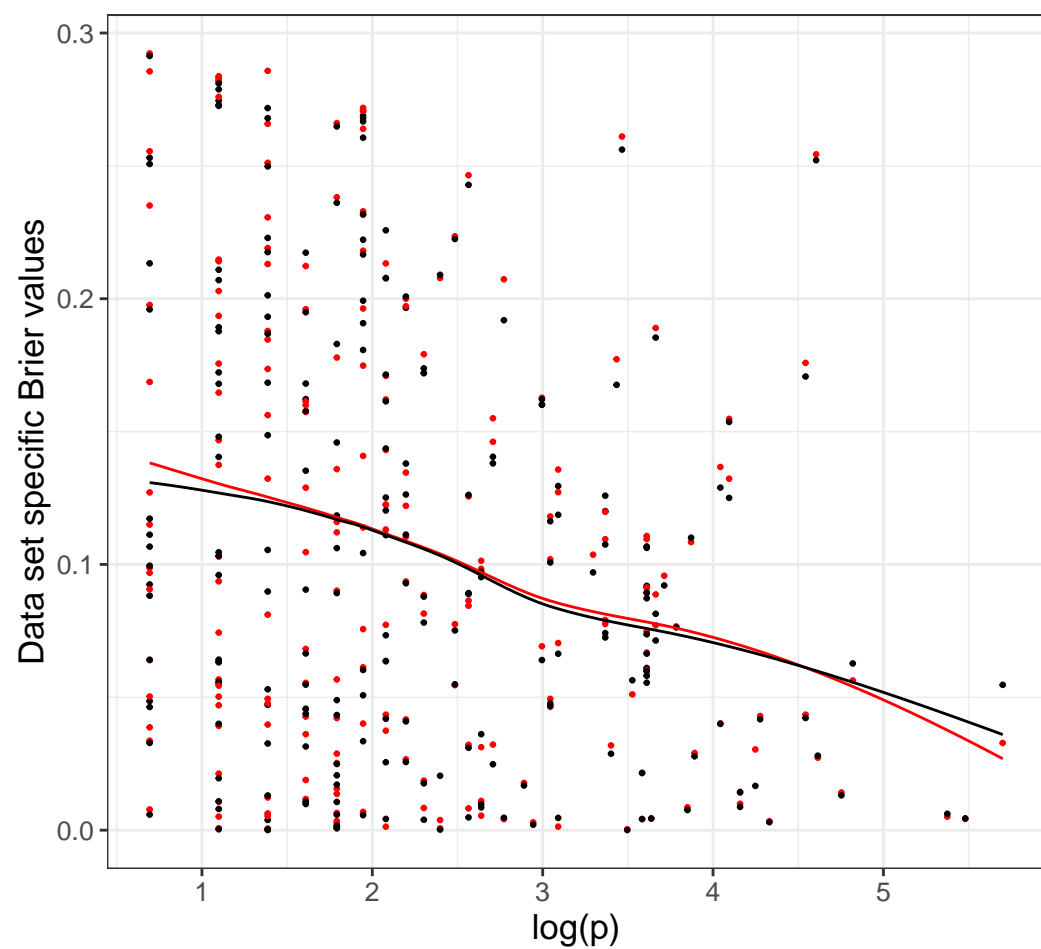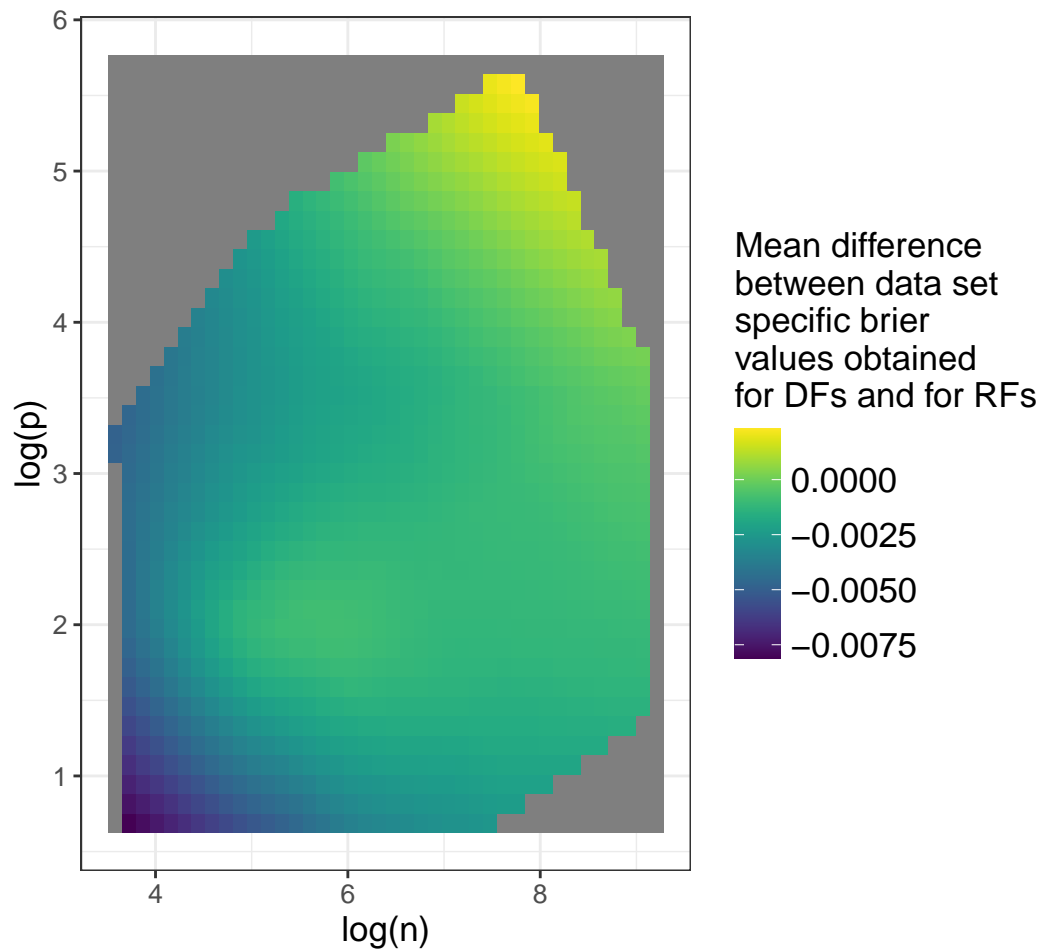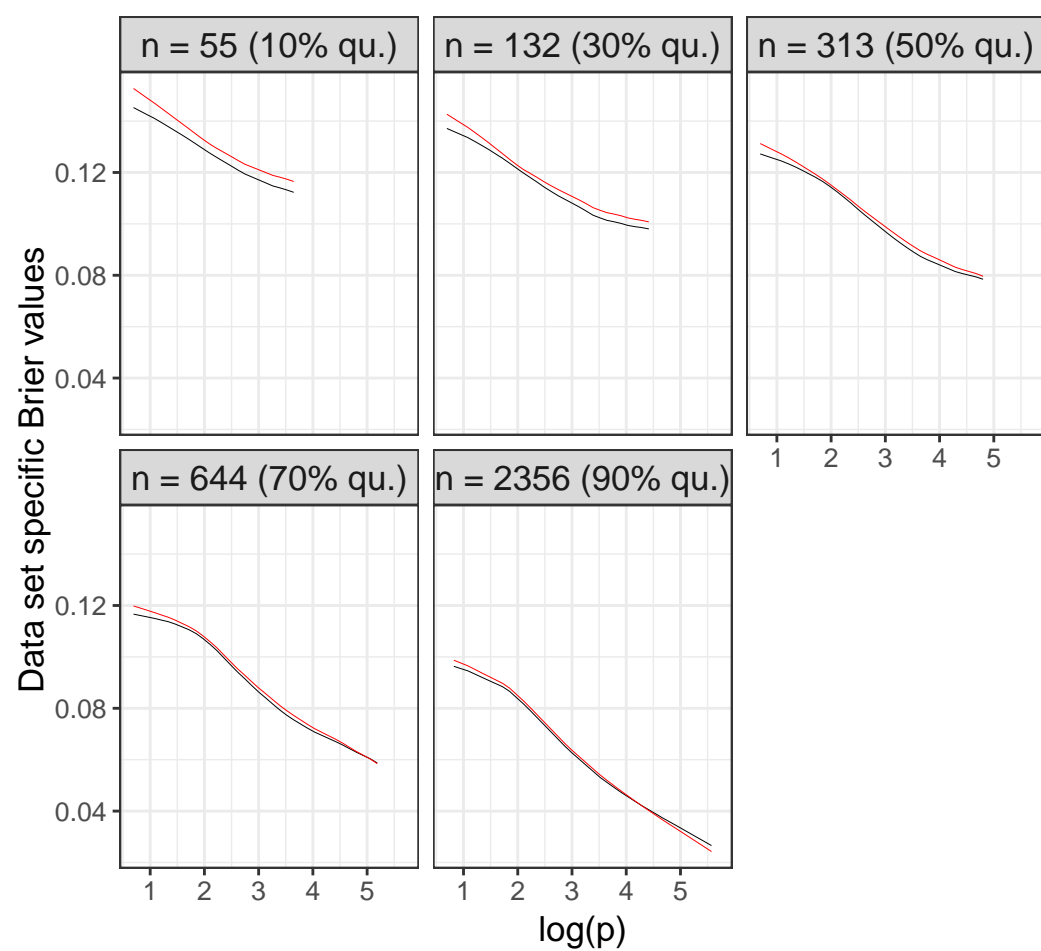

Supplement: Supplementary file 2 — Supplementary file1 (ZIP 108032 KB) [file 42979_2021_920_MOESM2_ESM.zip › Online_Resource_2/Results/Figures/DataCharacteristics_brier.pdf]

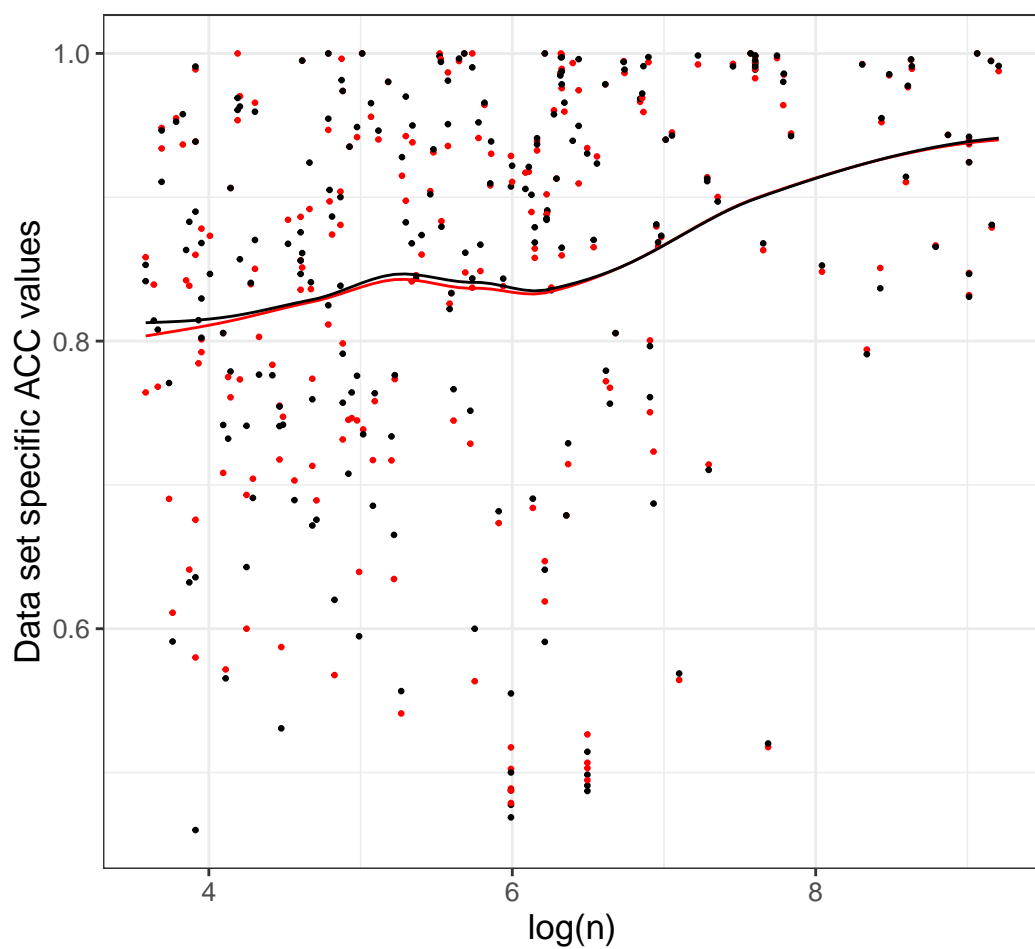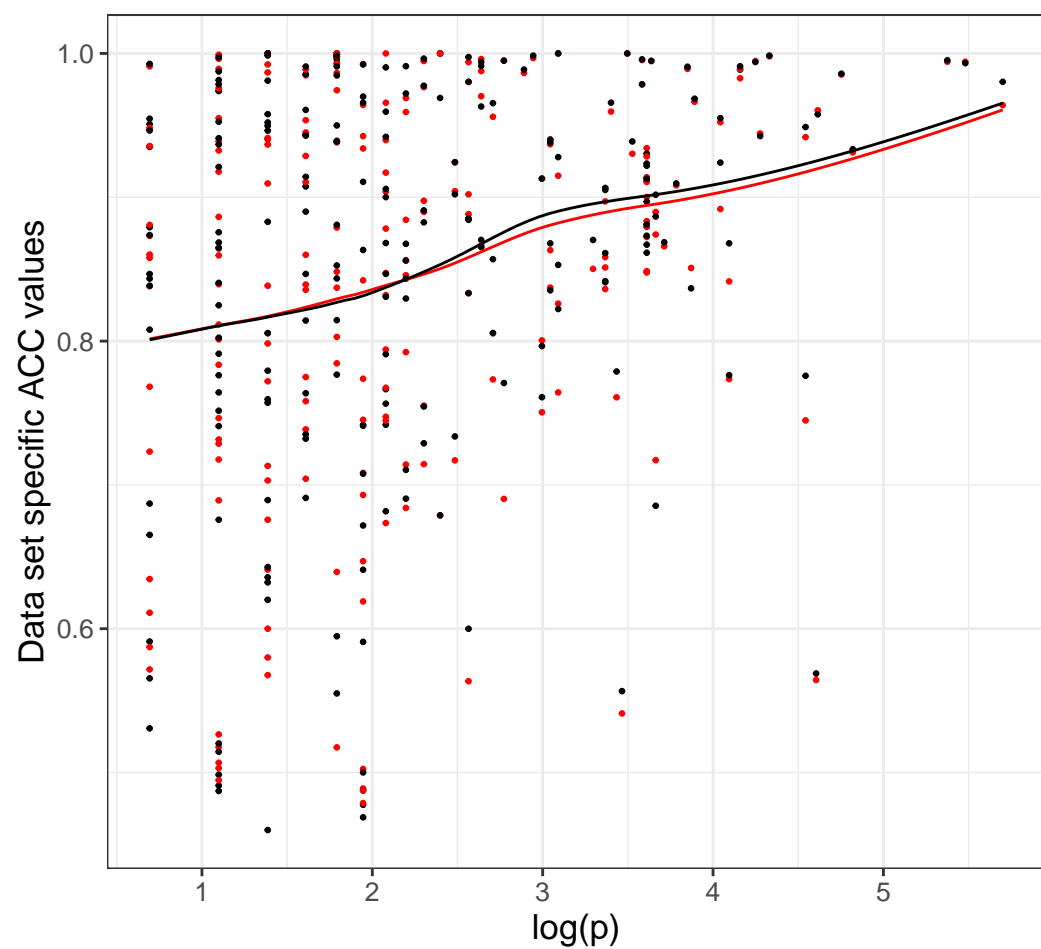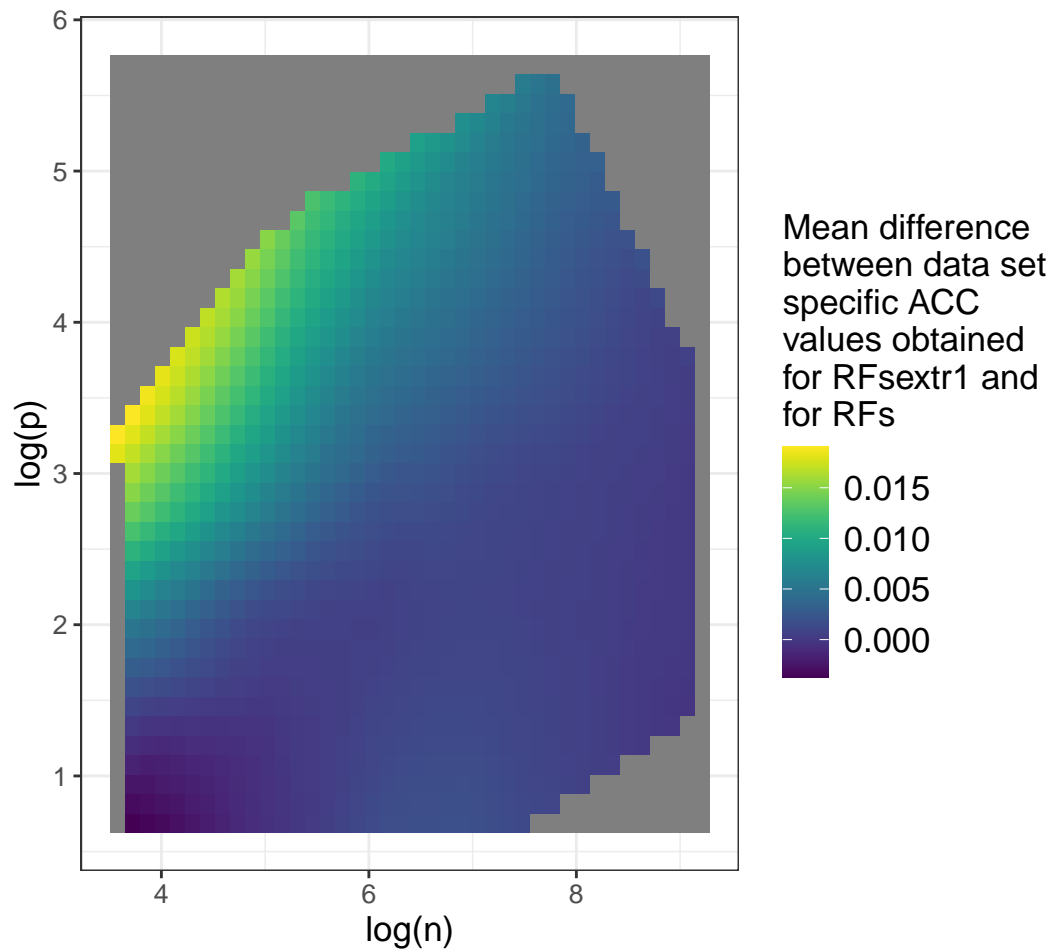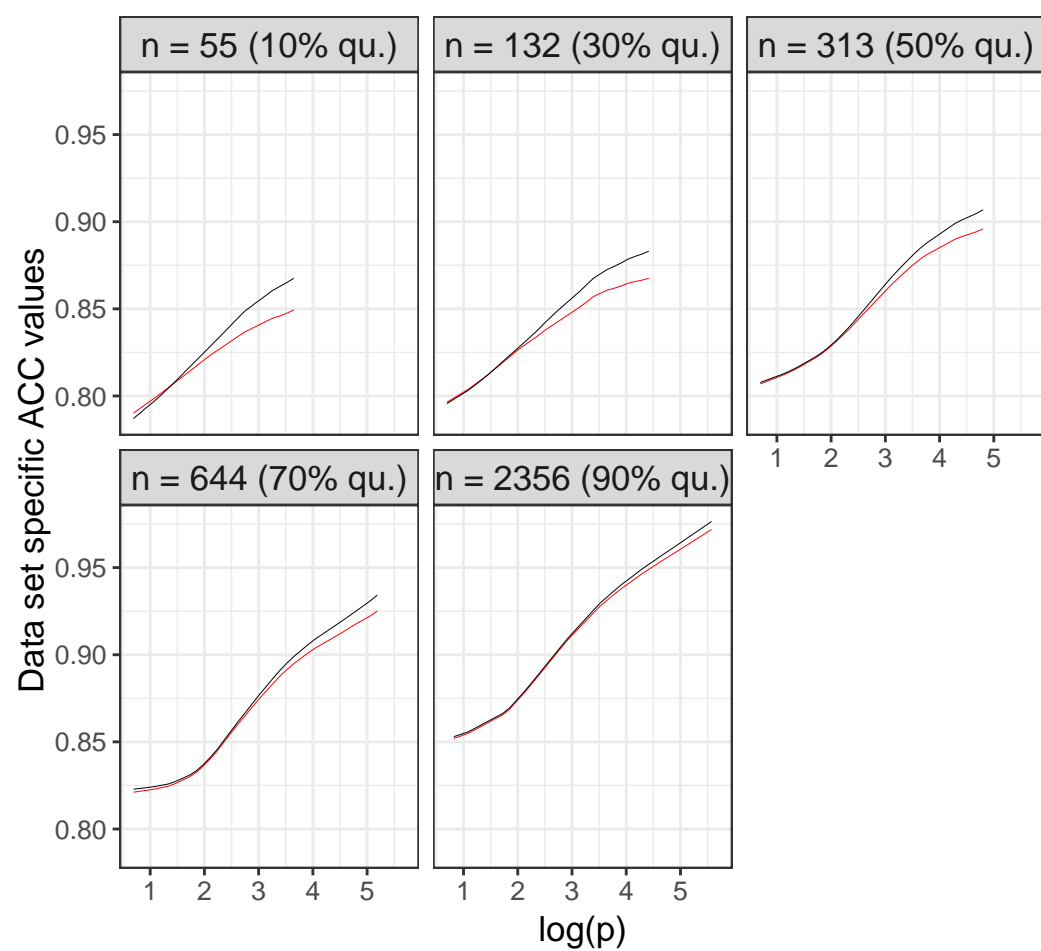

Supplement: Supplementary file 2 — Supplementary file1 (ZIP 108032 KB) [file 42979_2021_920_MOESM2_ESM.zip › Online_Resource_2/Results/Figures/DataCharacteristics_RFextr1.pdf]

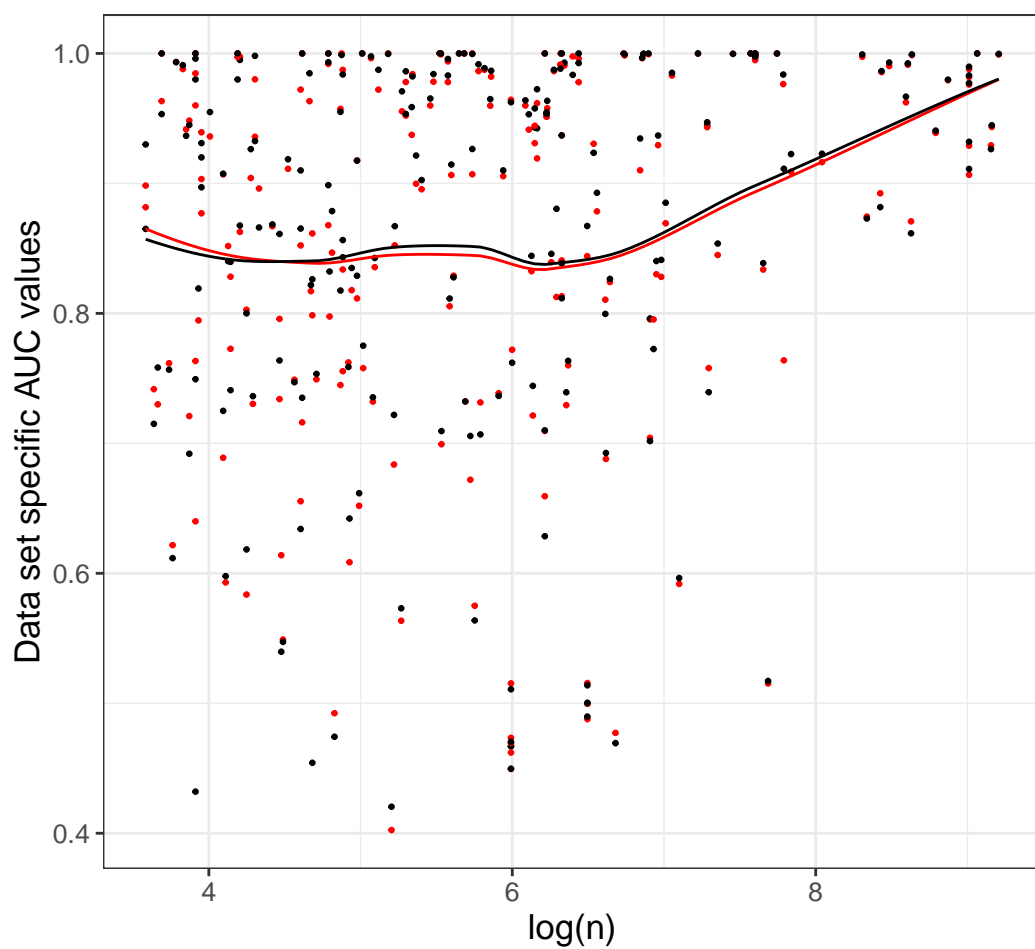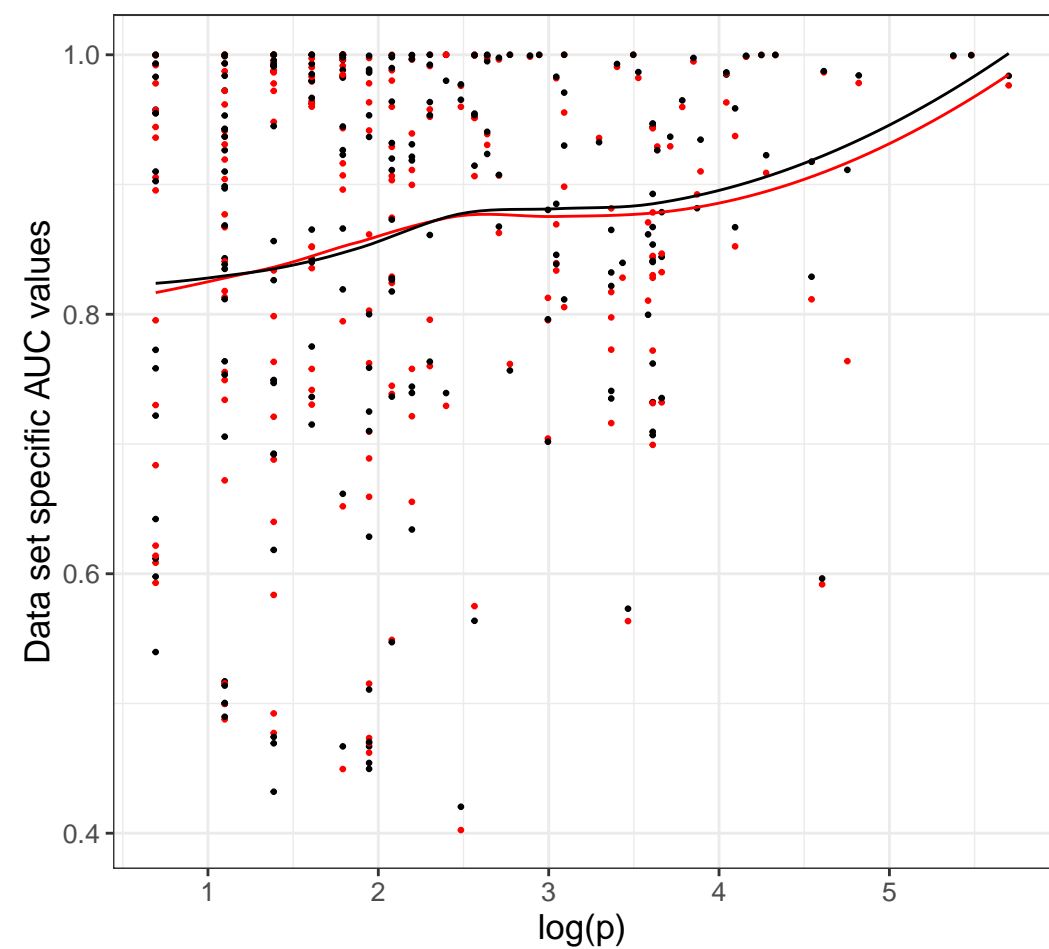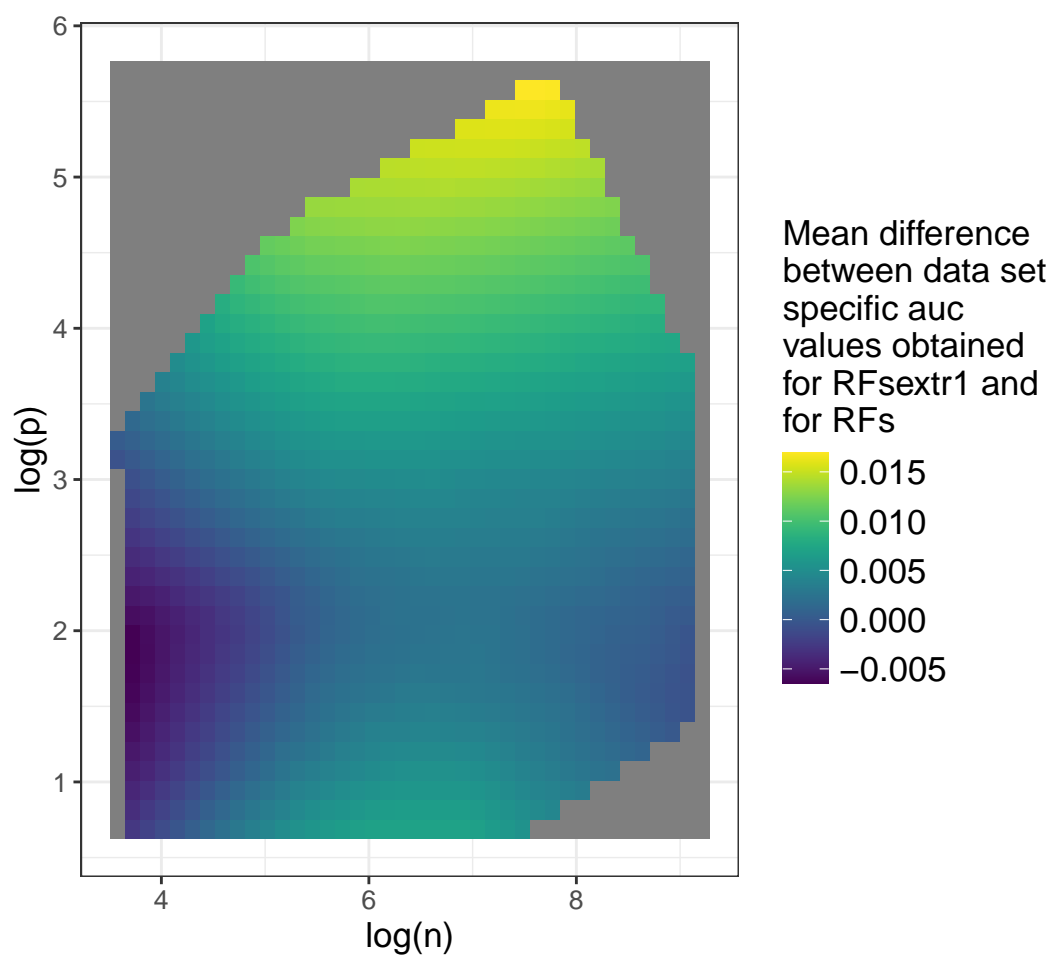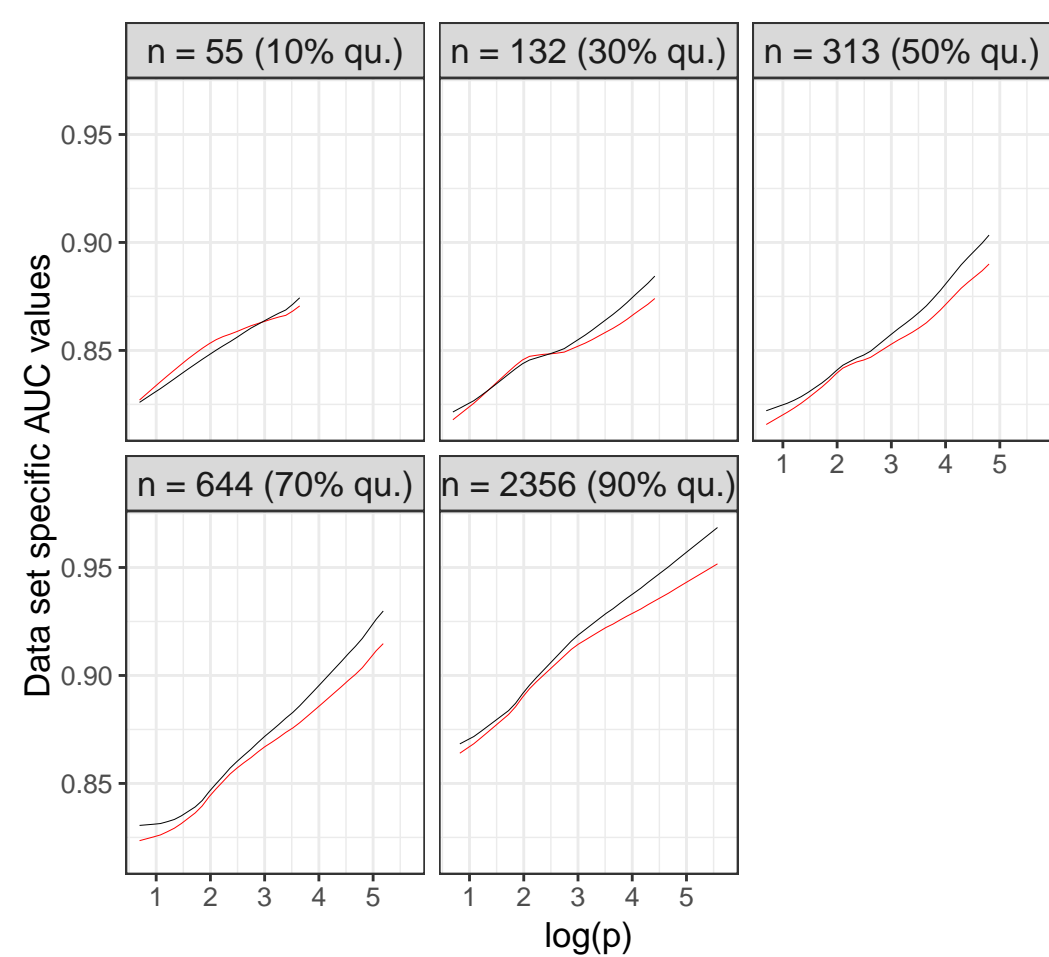

Supplement: Supplementary file 2 — Supplementary file1 (ZIP 108032 KB) [file 42979_2021_920_MOESM2_ESM.zip › Online_Resource_2/Results/Figures/DataCharacteristics_RFextr1_auc.pdf]

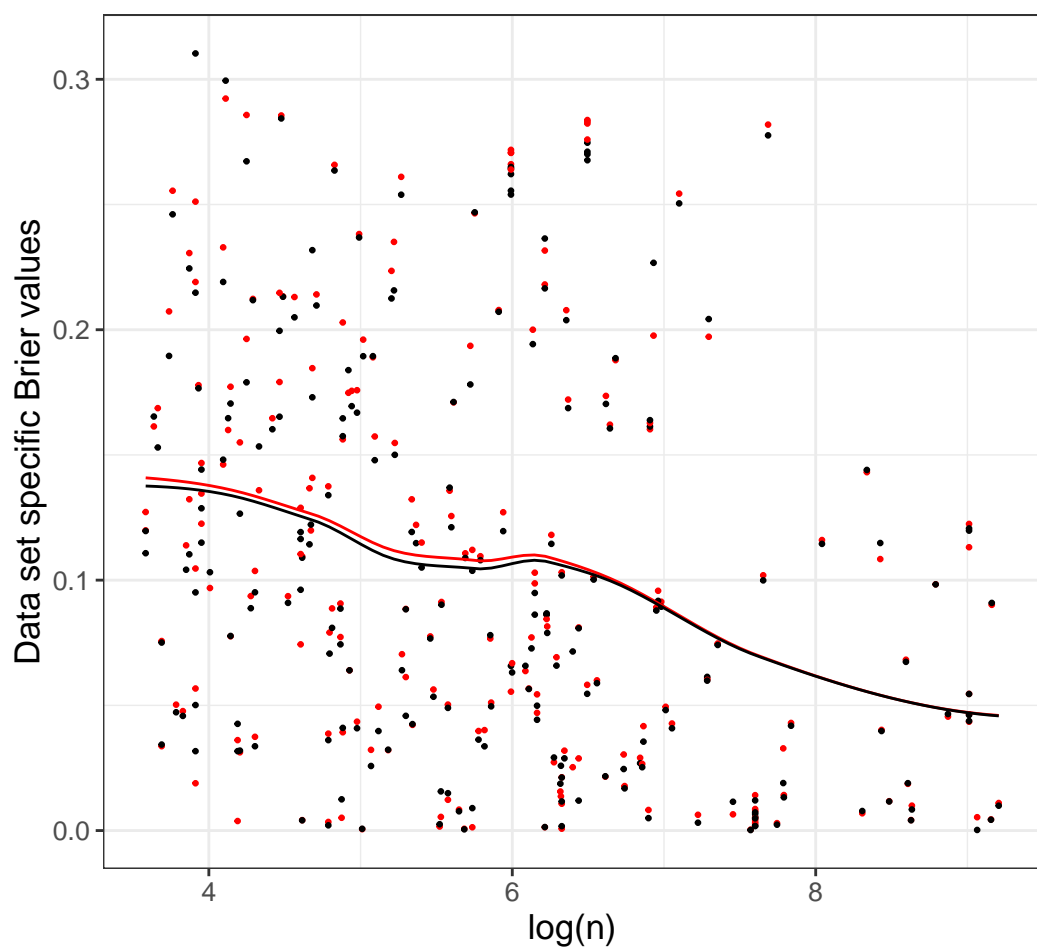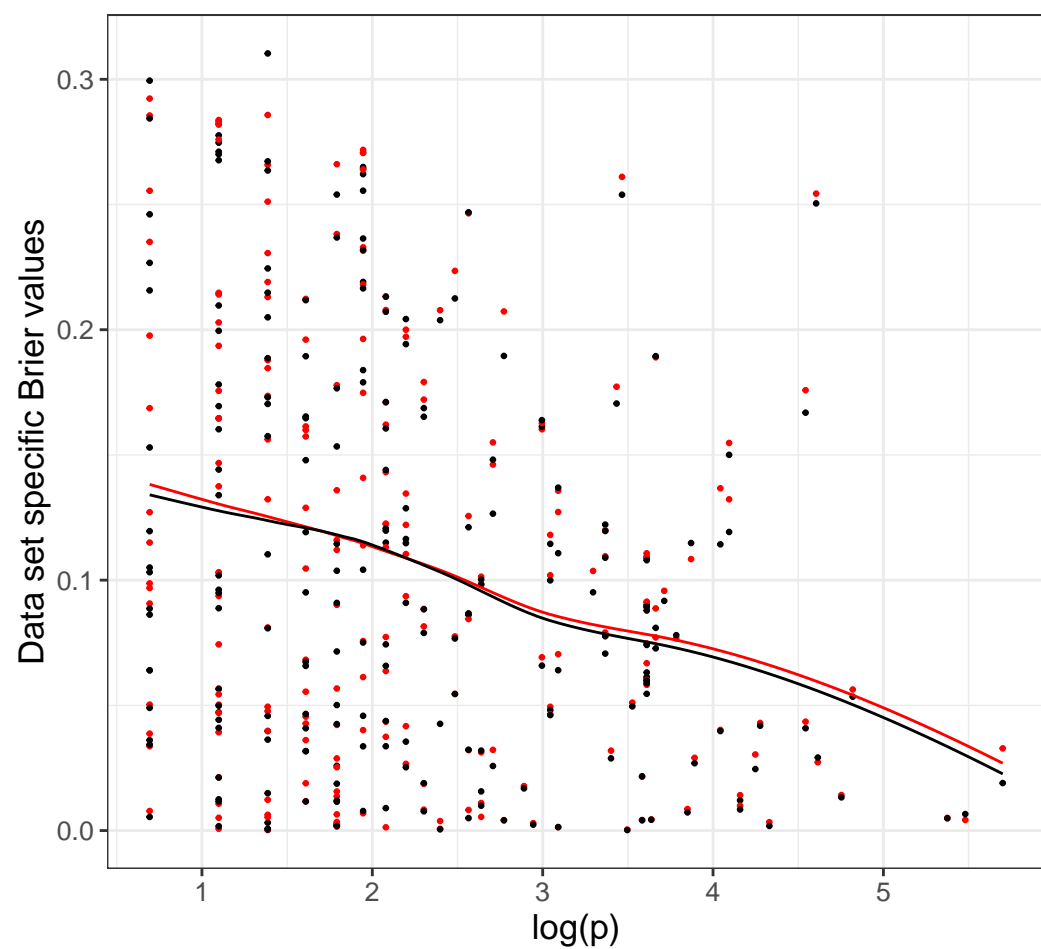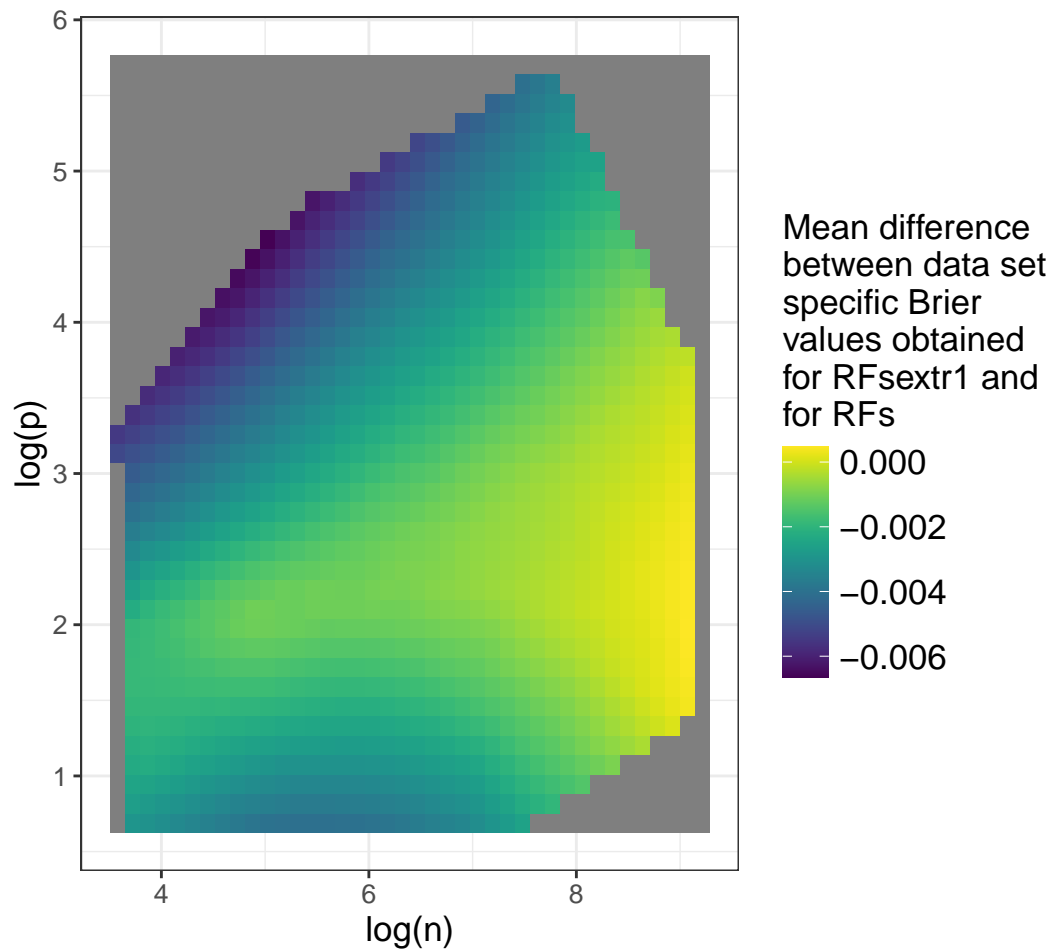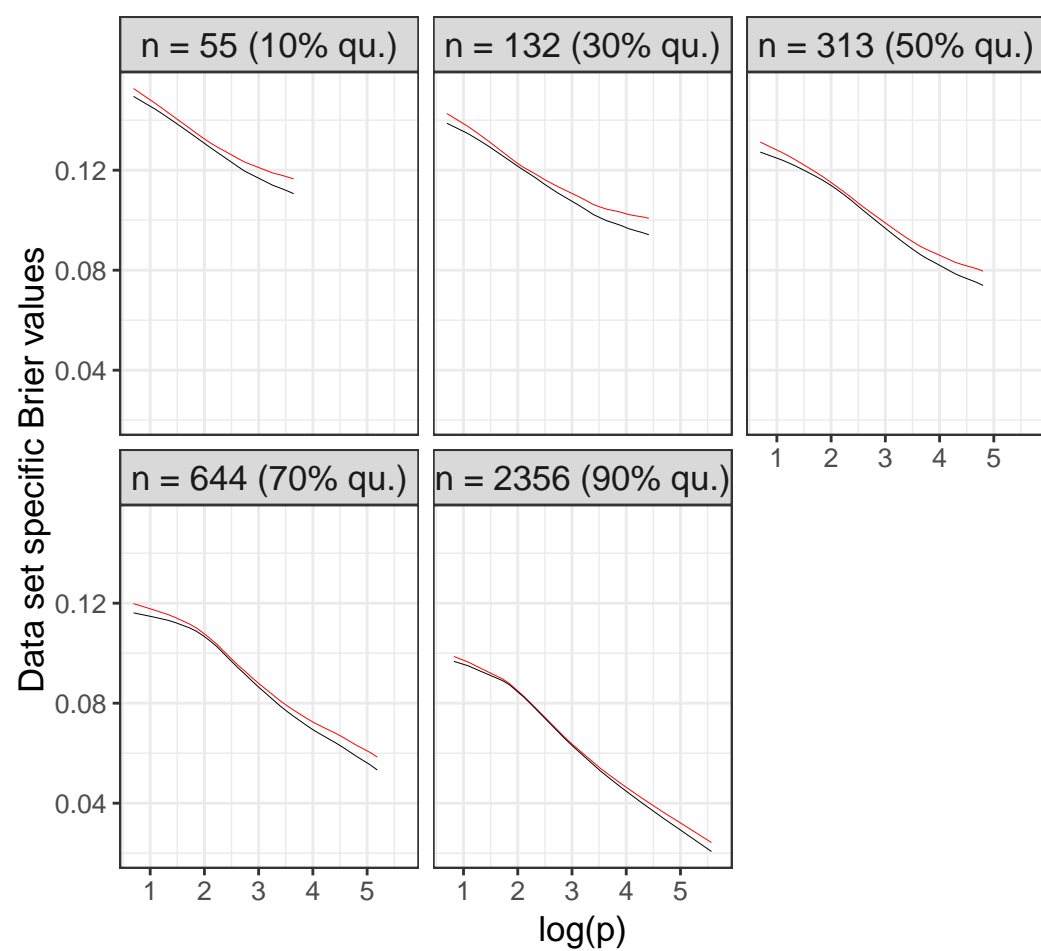

Supplement: Supplementary file 2 — Supplementary file1 (ZIP 108032 KB) [file 42979_2021_920_MOESM2_ESM.zip › Online_Resource_2/Results/Figures/DataCharacteristics_RFextr1_brier.pdf]

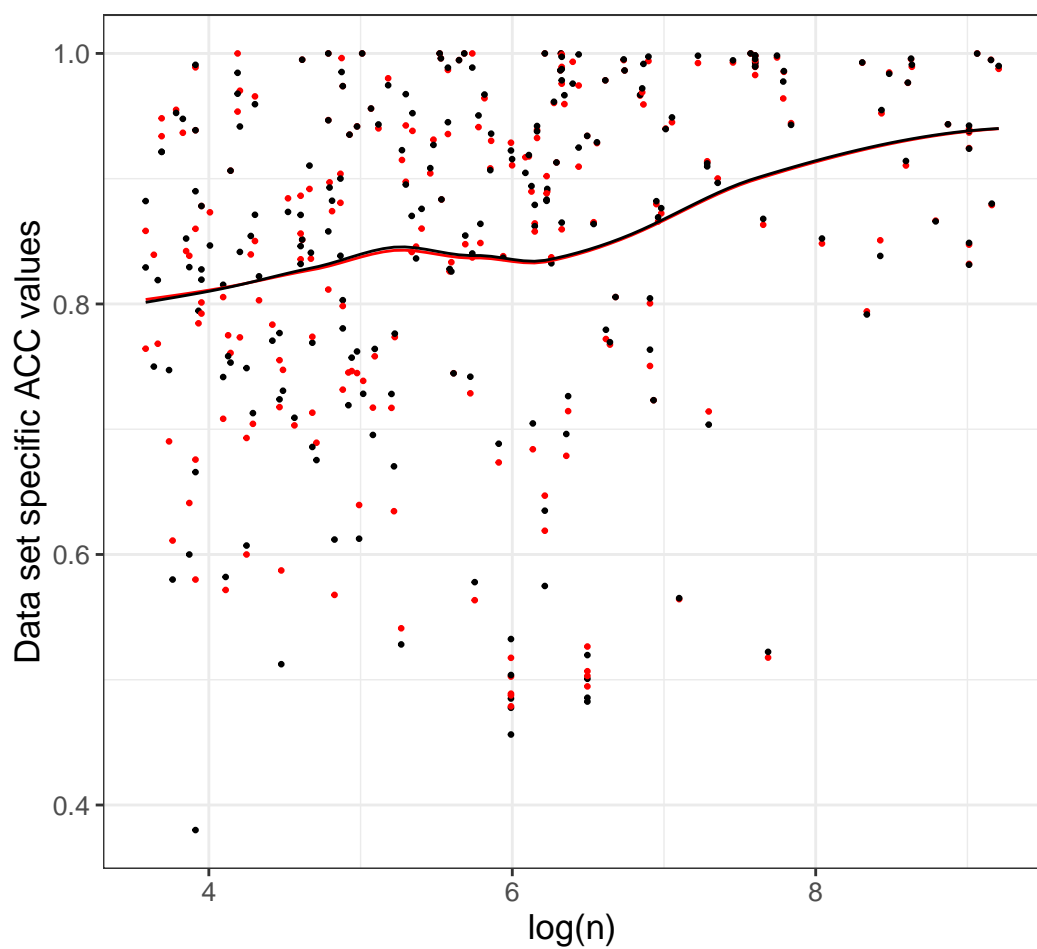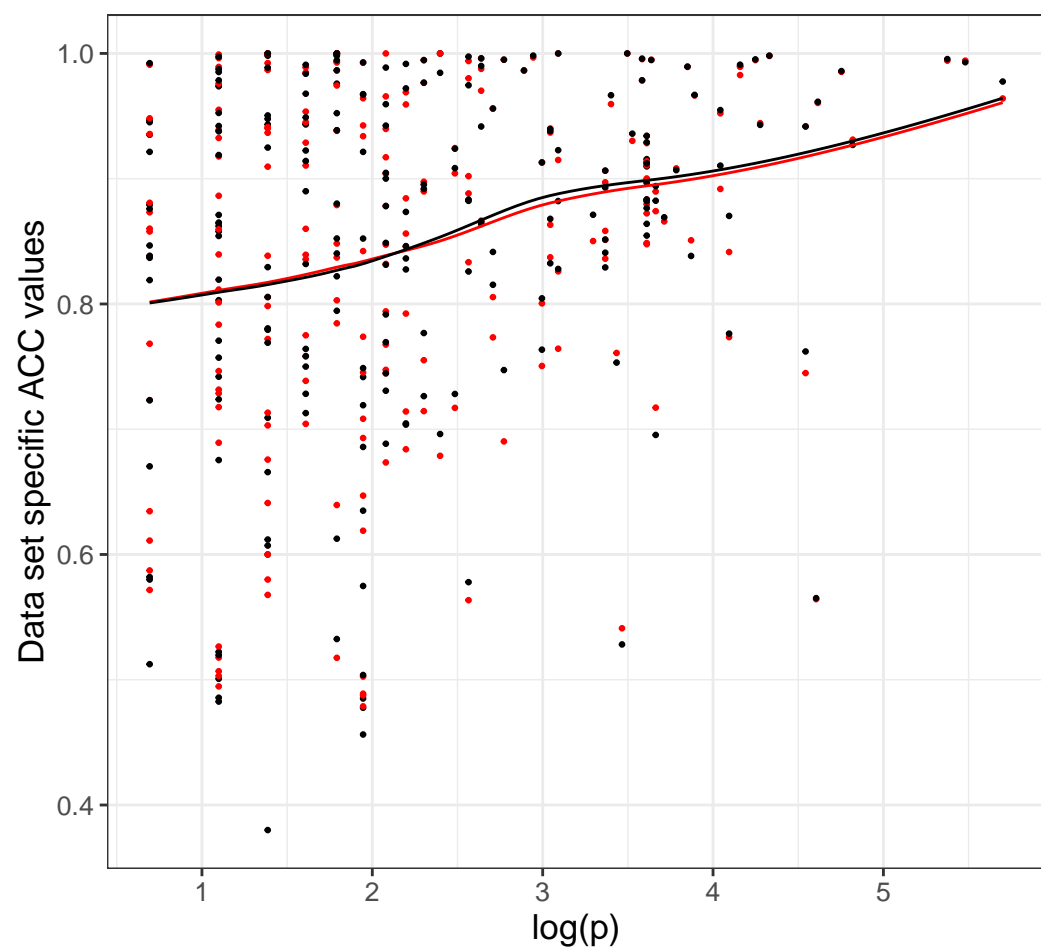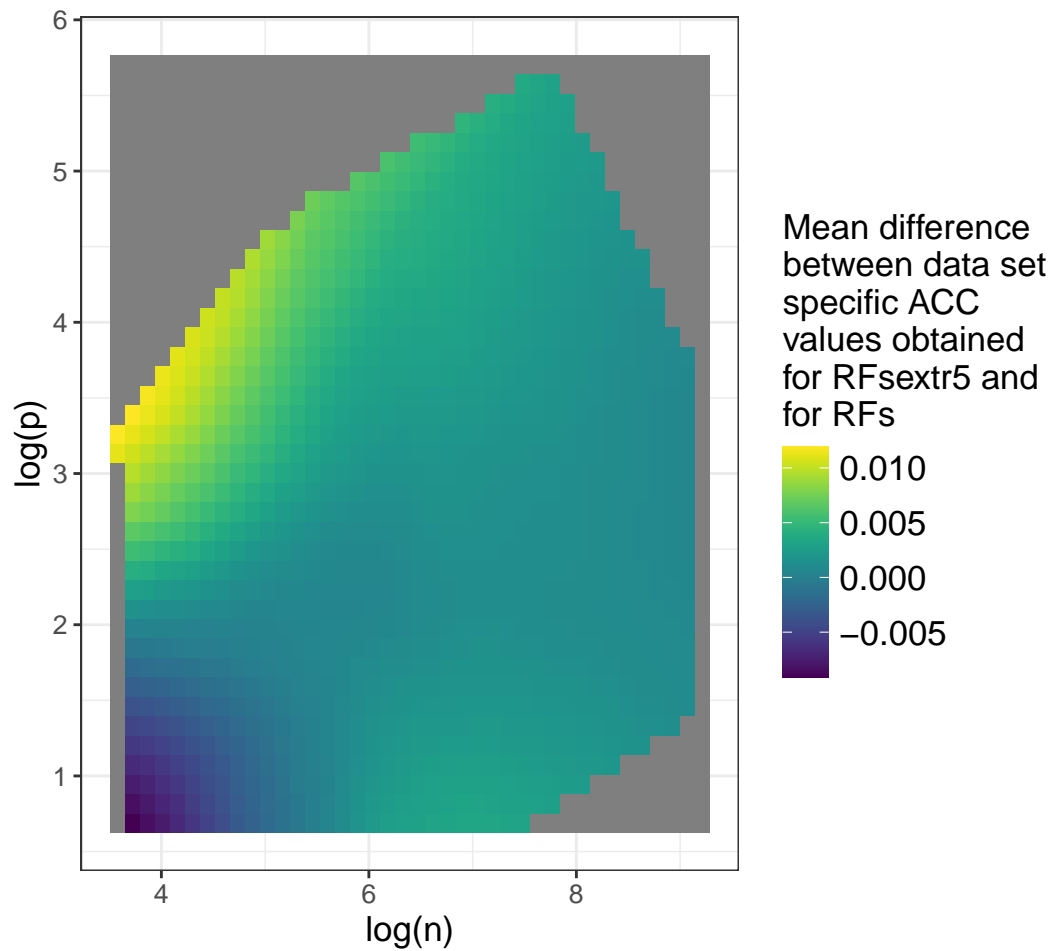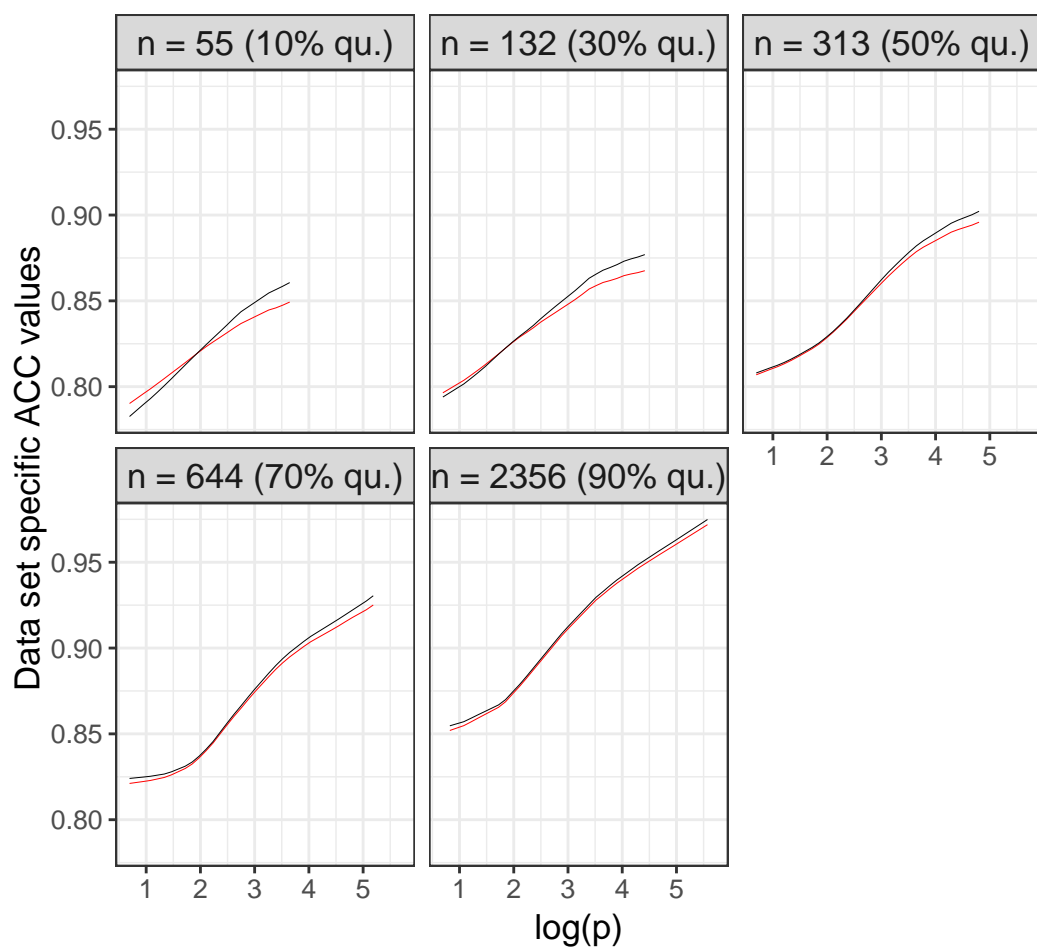

Supplement: Supplementary file 2 — Supplementary file1 (ZIP 108032 KB) [file 42979_2021_920_MOESM2_ESM.zip › Online_Resource_2/Results/Figures/DataCharacteristics_RFextr5.pdf]

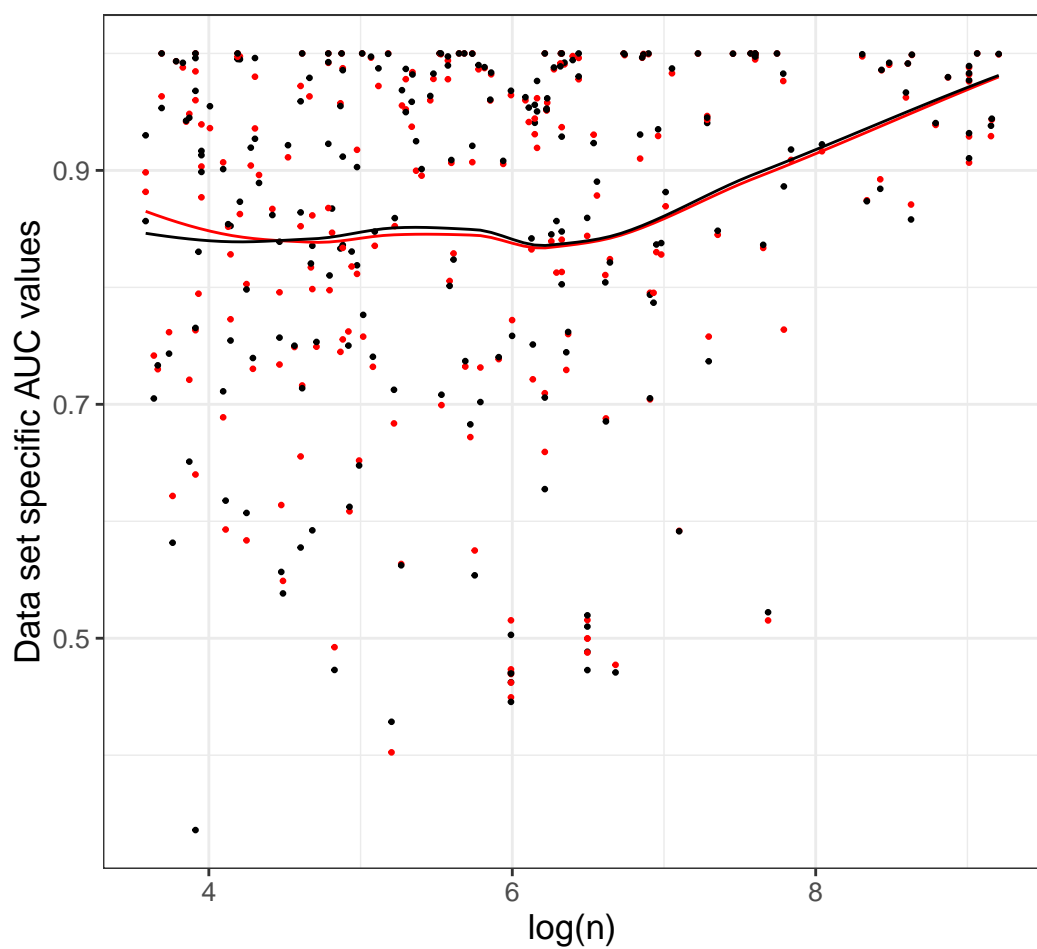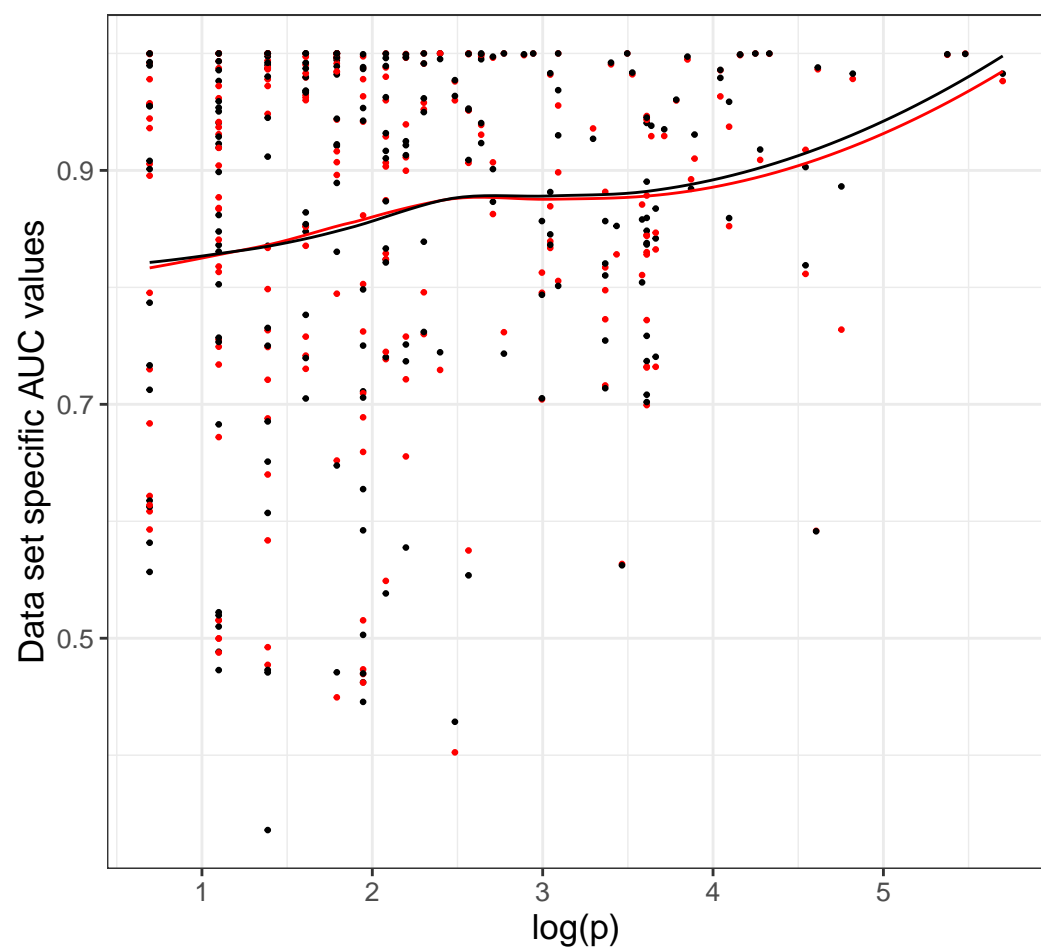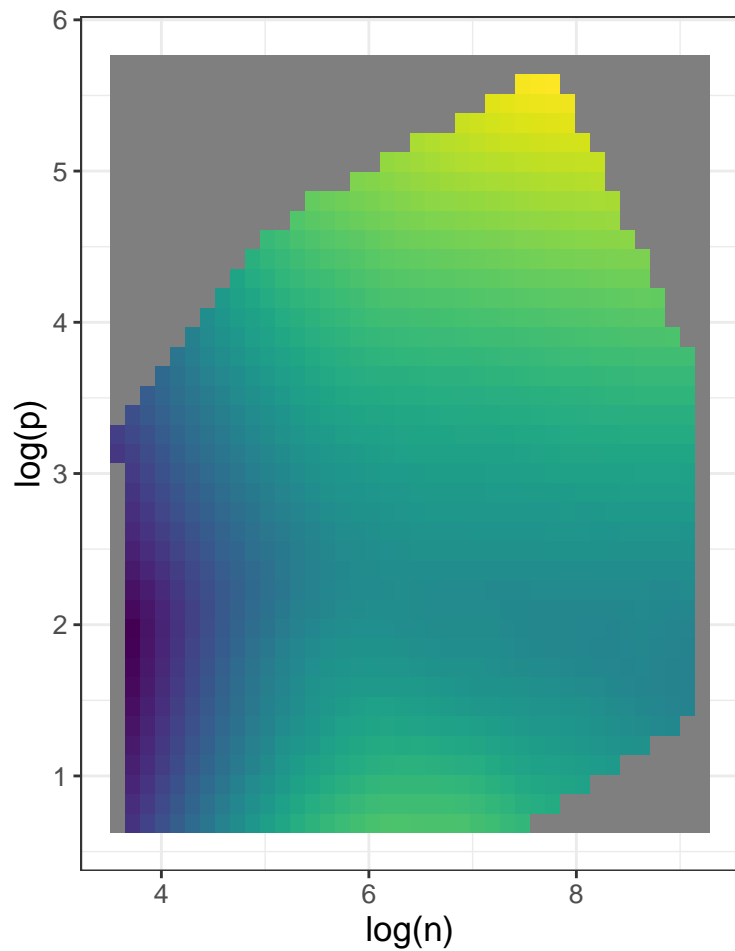

Mean difference  
between data set  
specific auc  
values obtained  
for RFsextr5 and  
for RFs

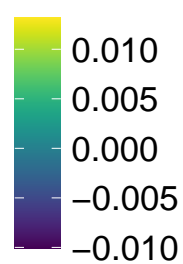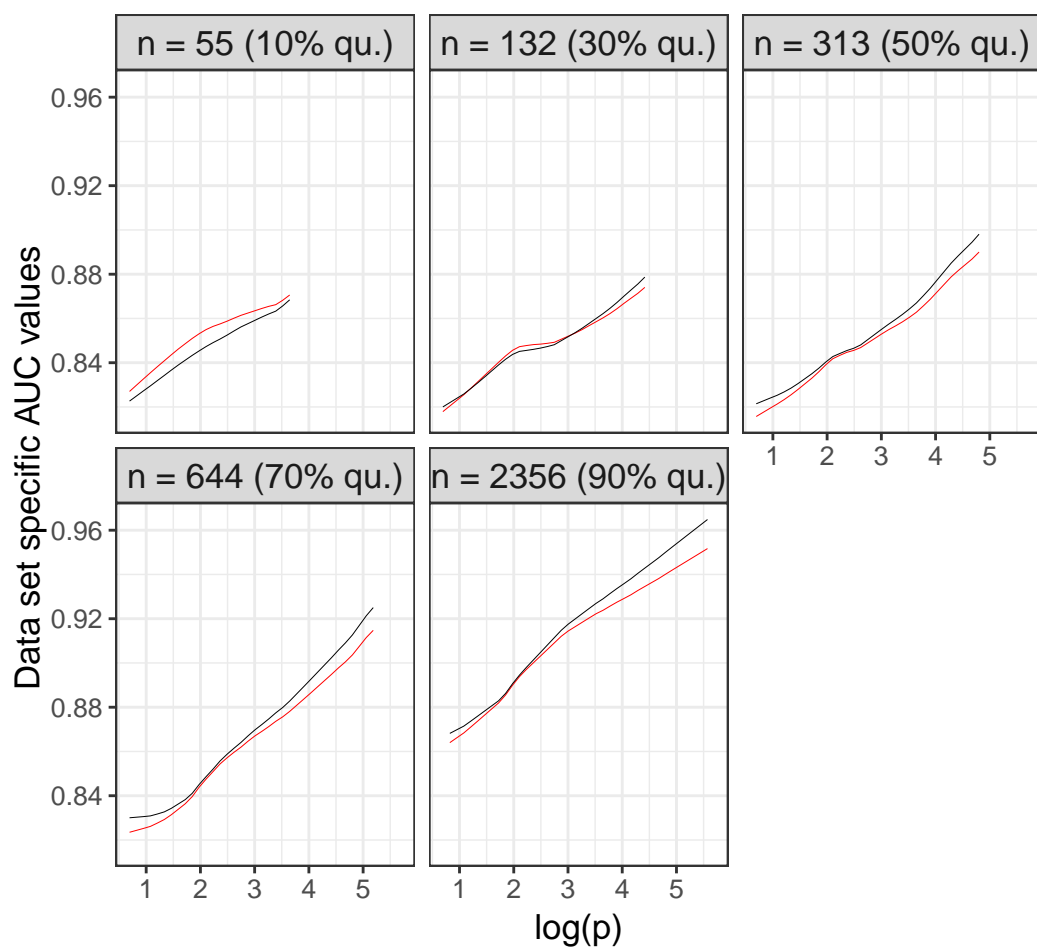

Supplement: Supplementary file 2 — Supplementary file1 (ZIP 108032 KB) [file 42979_2021_920_MOESM2_ESM.zip › Online_Resource_2/Results/Figures/DataCharacteristics_RFextr5_auc.pdf]

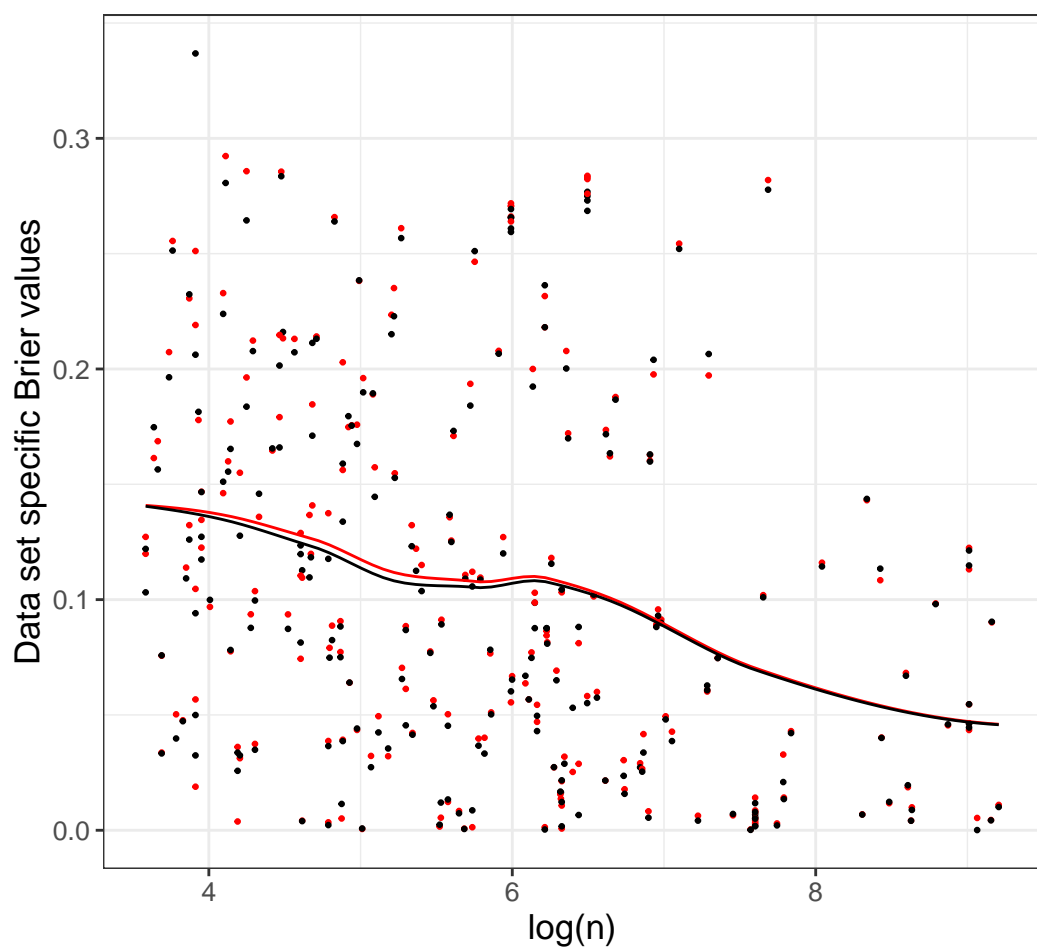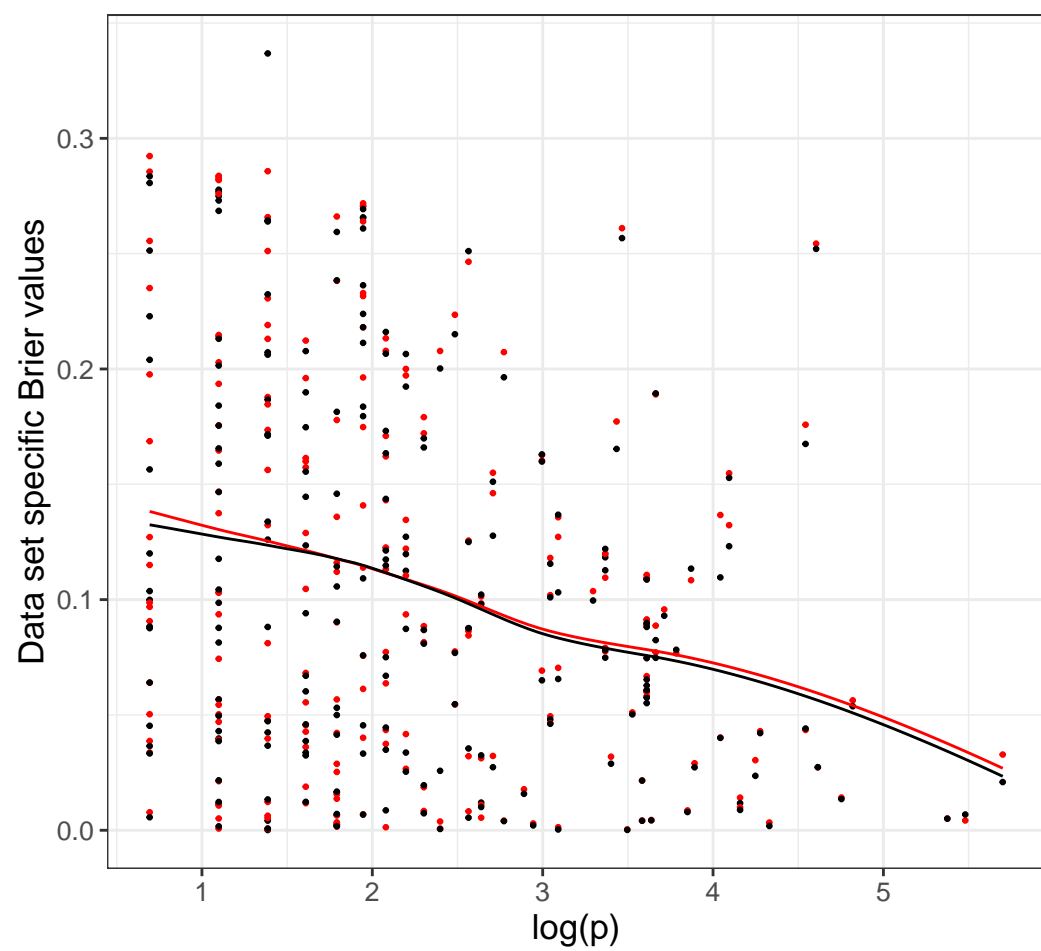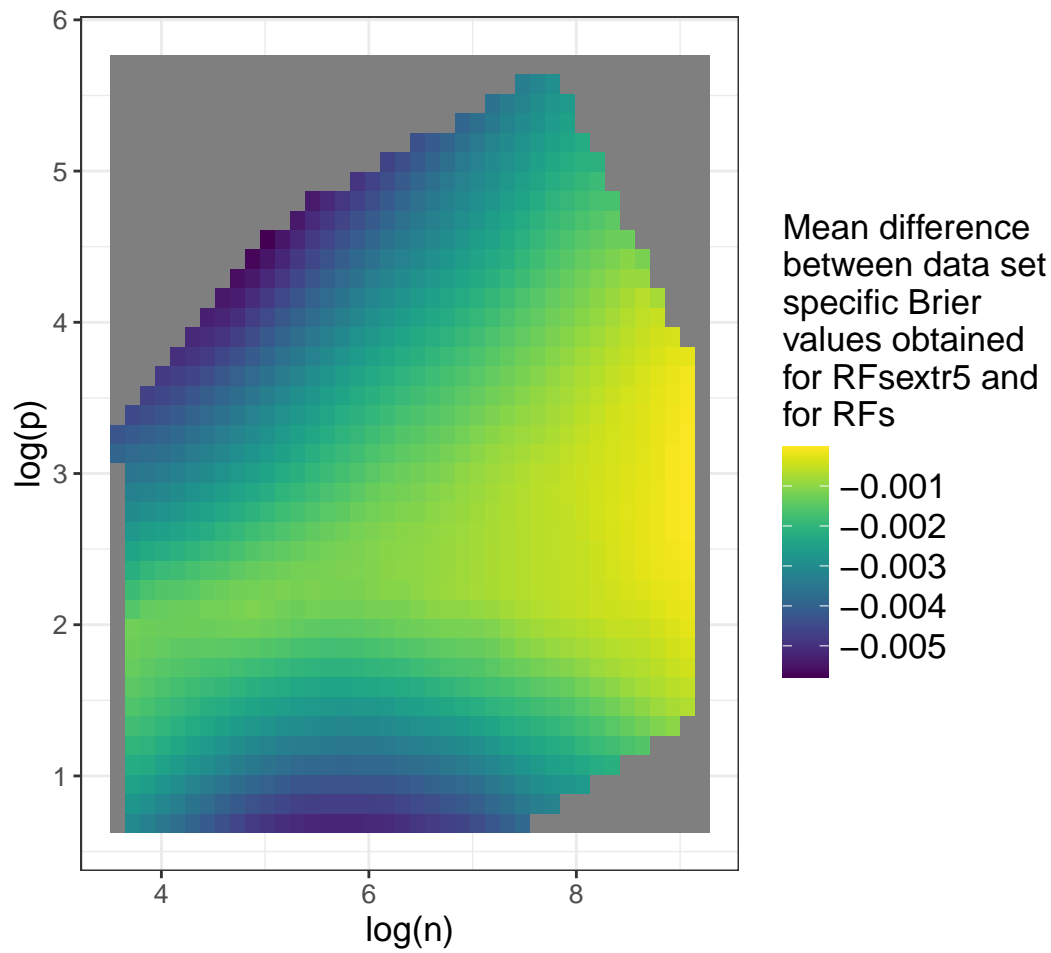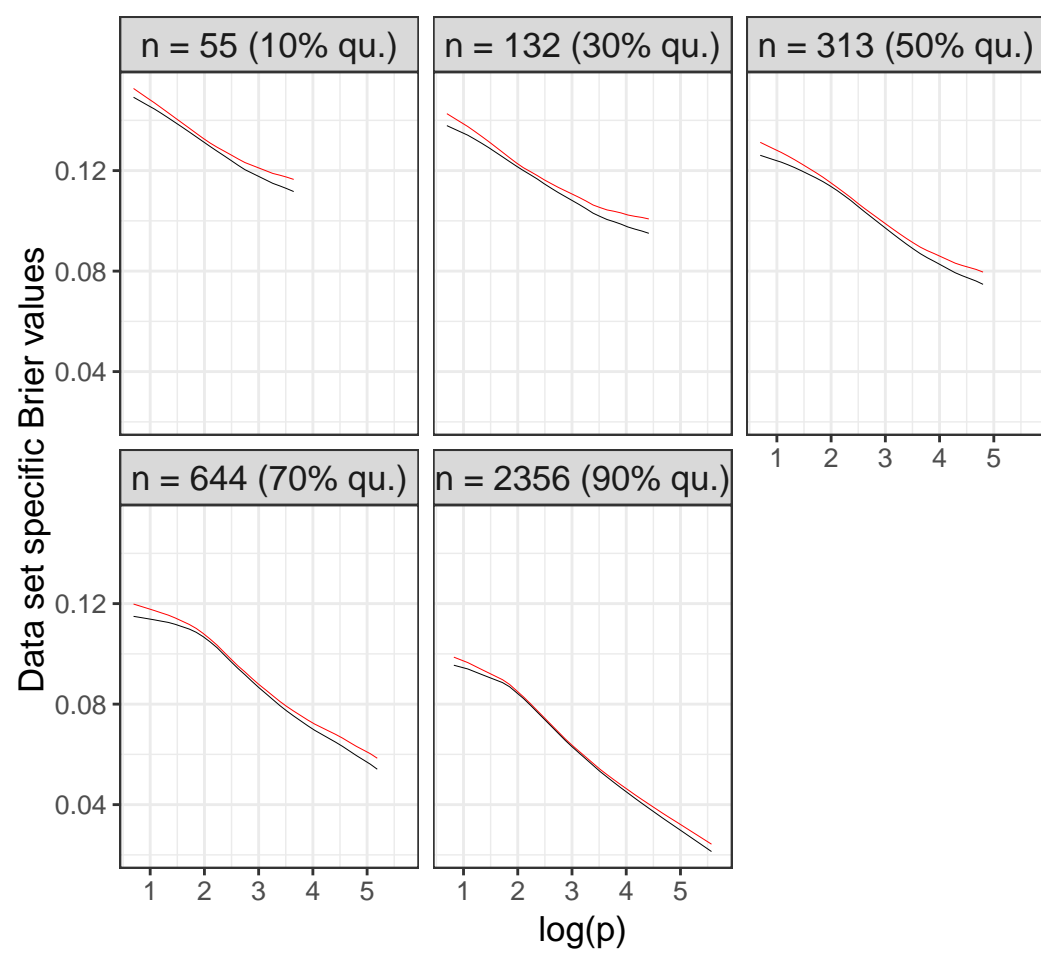

Supplement: Supplementary file 2 — Supplementary file1 (ZIP 108032 KB) [file 42979_2021_920_MOESM2_ESM.zip › Online_Resource_2/Results/Figures/DataCharacteristics_RFextr5_brier.pdf]

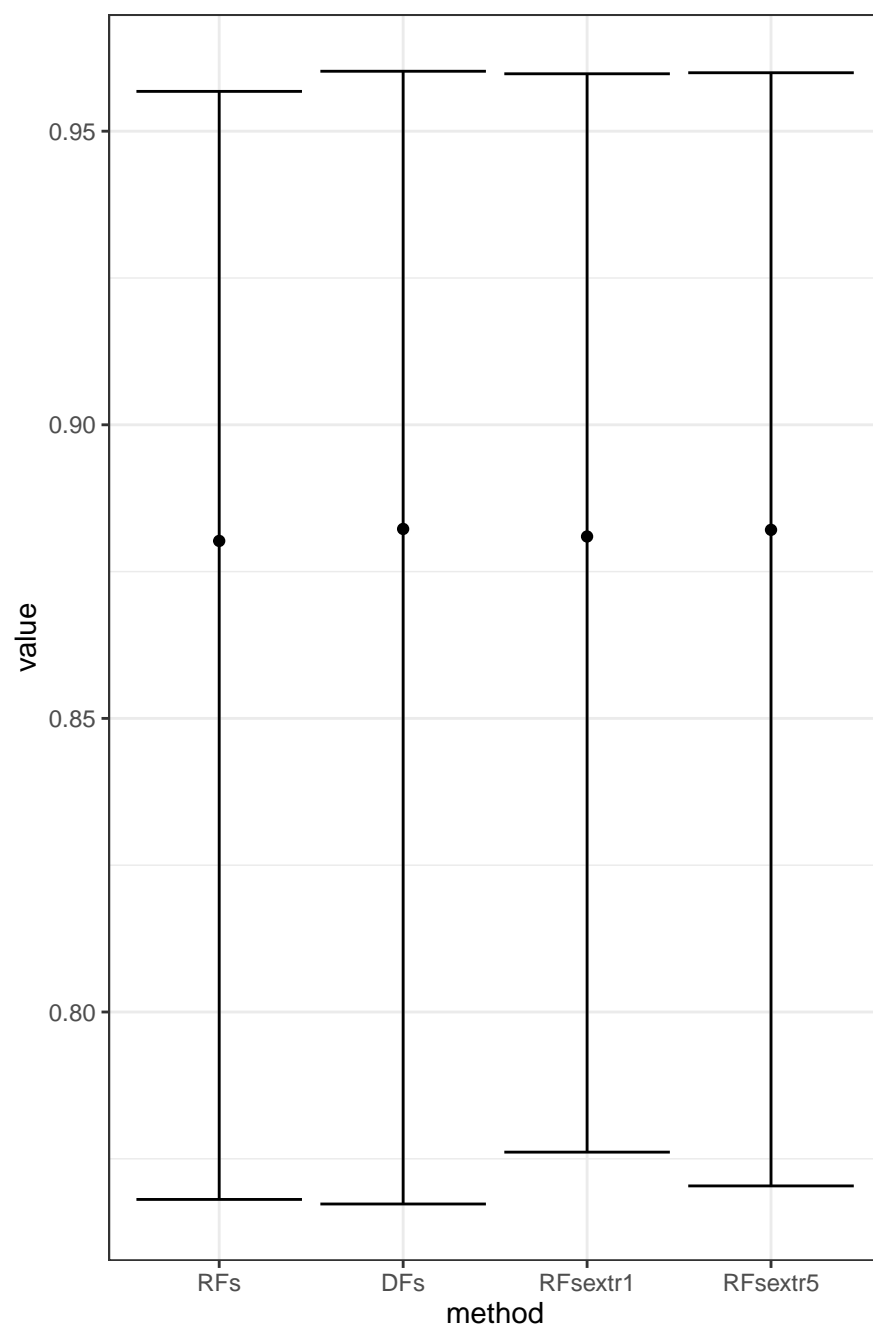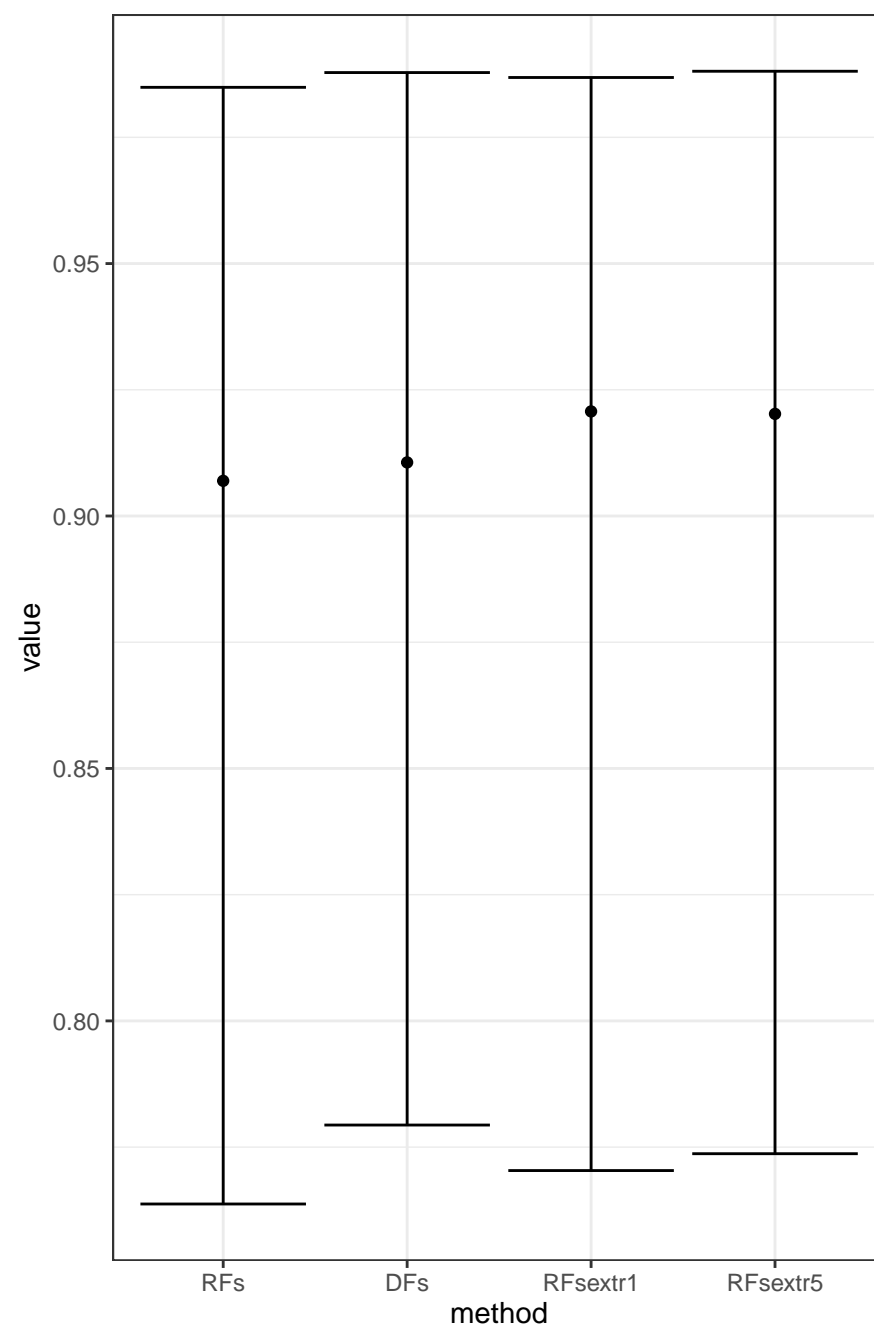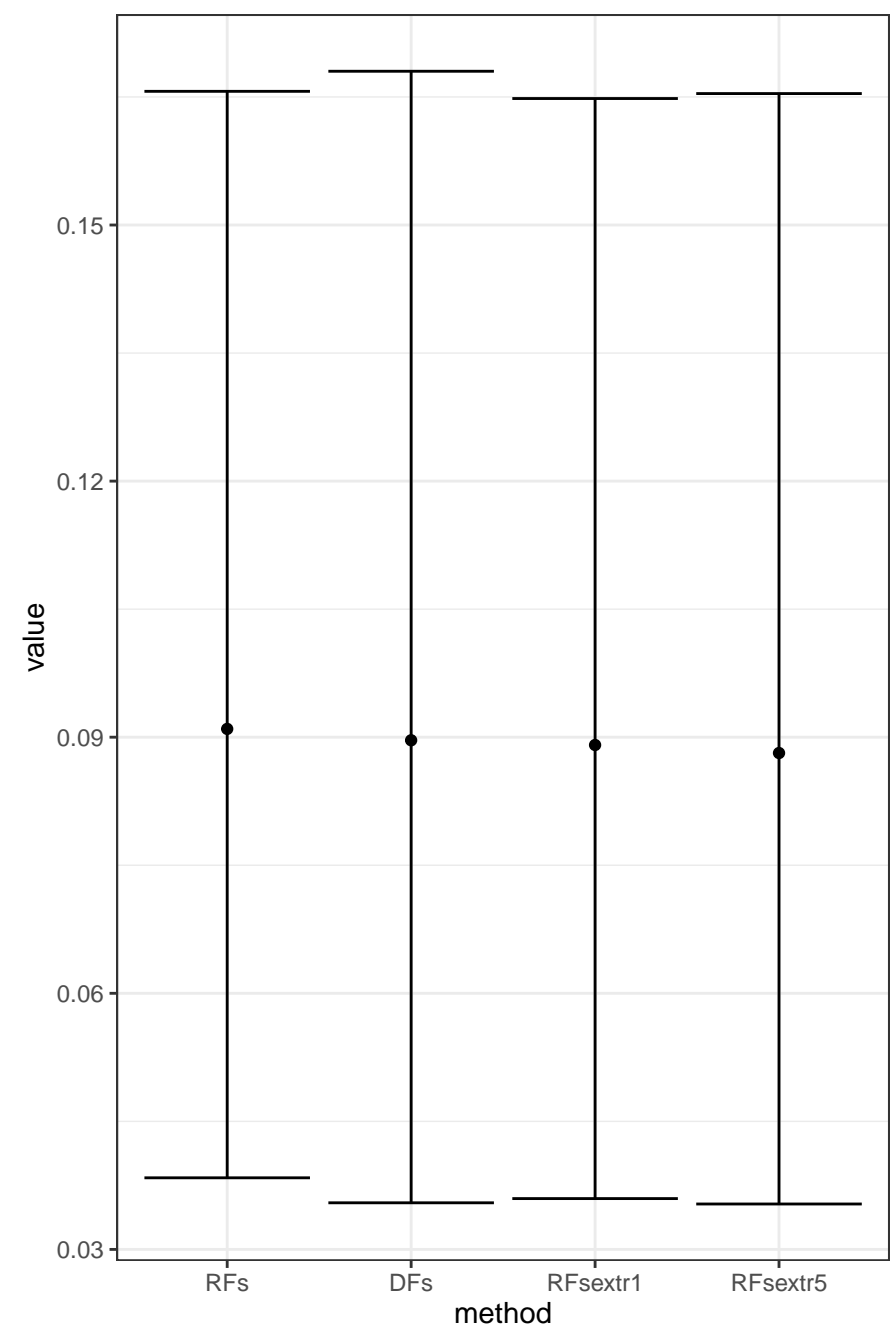

Supplement: Supplementary file 2 — Supplementary file1 (ZIP 108032 KB) [file 42979_2021_920_MOESM2_ESM.zip › Online_Resource_2/Results/Figures/FigureErrorBars.pdf]

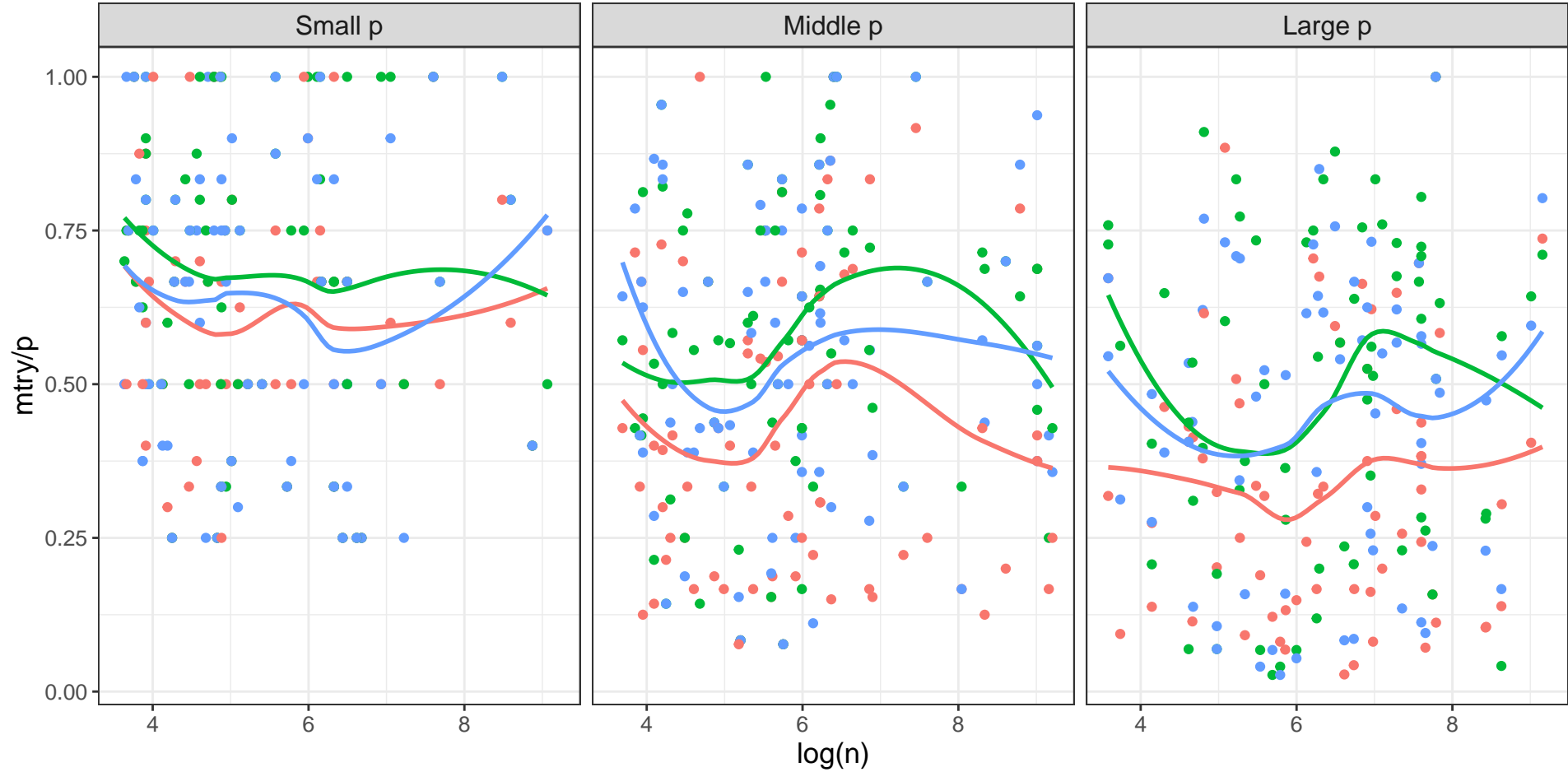

Supplement: Supplementary file 2 — Supplementary file1 (ZIP 108032 KB) [file 42979_2021_920_MOESM2_ESM.zip › Online_Resource_2/Results/Figures/InfluenceNstratified.pdf]

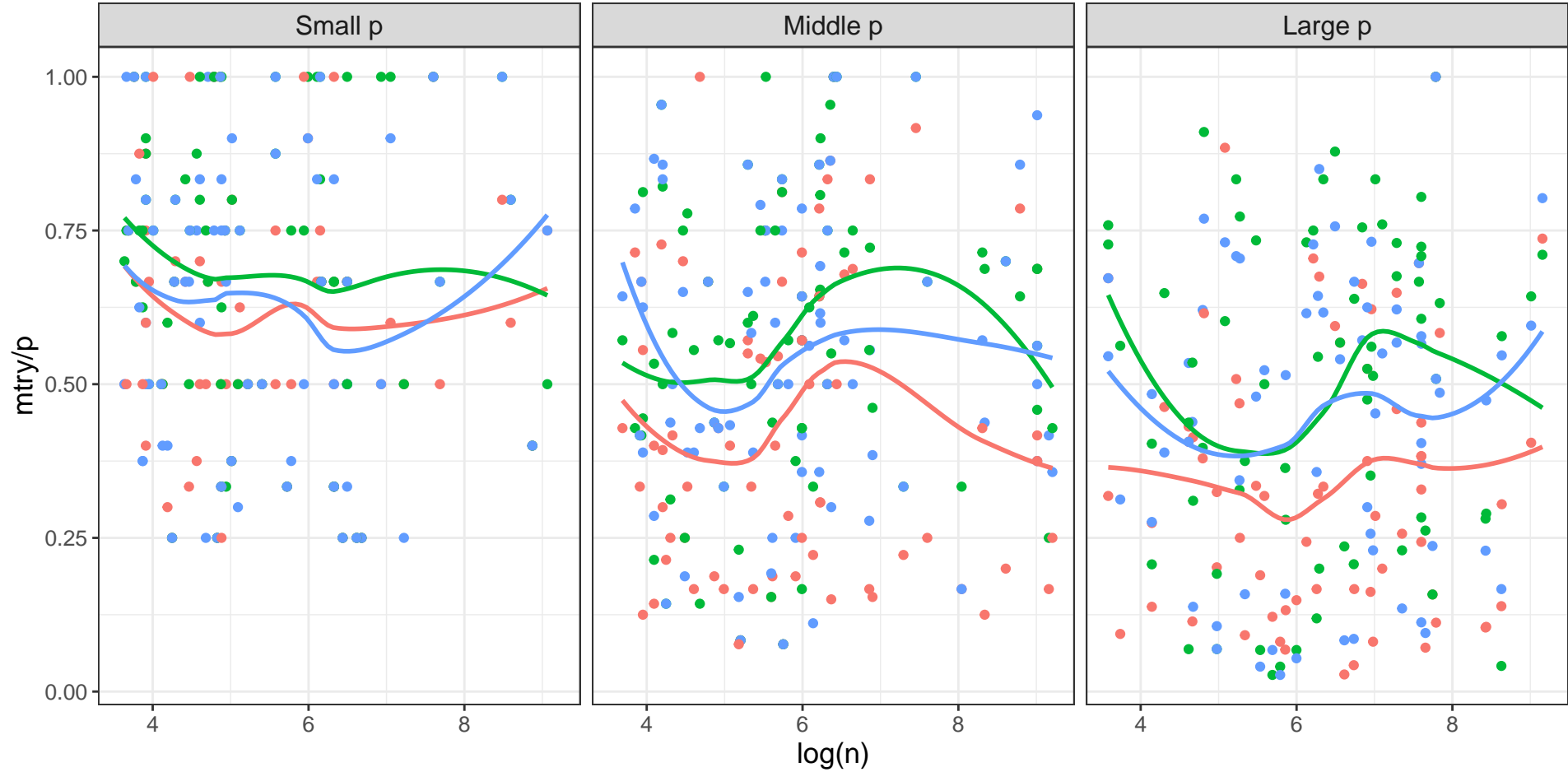

Supplement: Supplementary file 2 — Supplementary file1 (ZIP 108032 KB) [file 42979_2021_920_MOESM2_ESM.zip › Online_Resource_2/Results/Figures/InfluenceNstratified_auc.pdf]

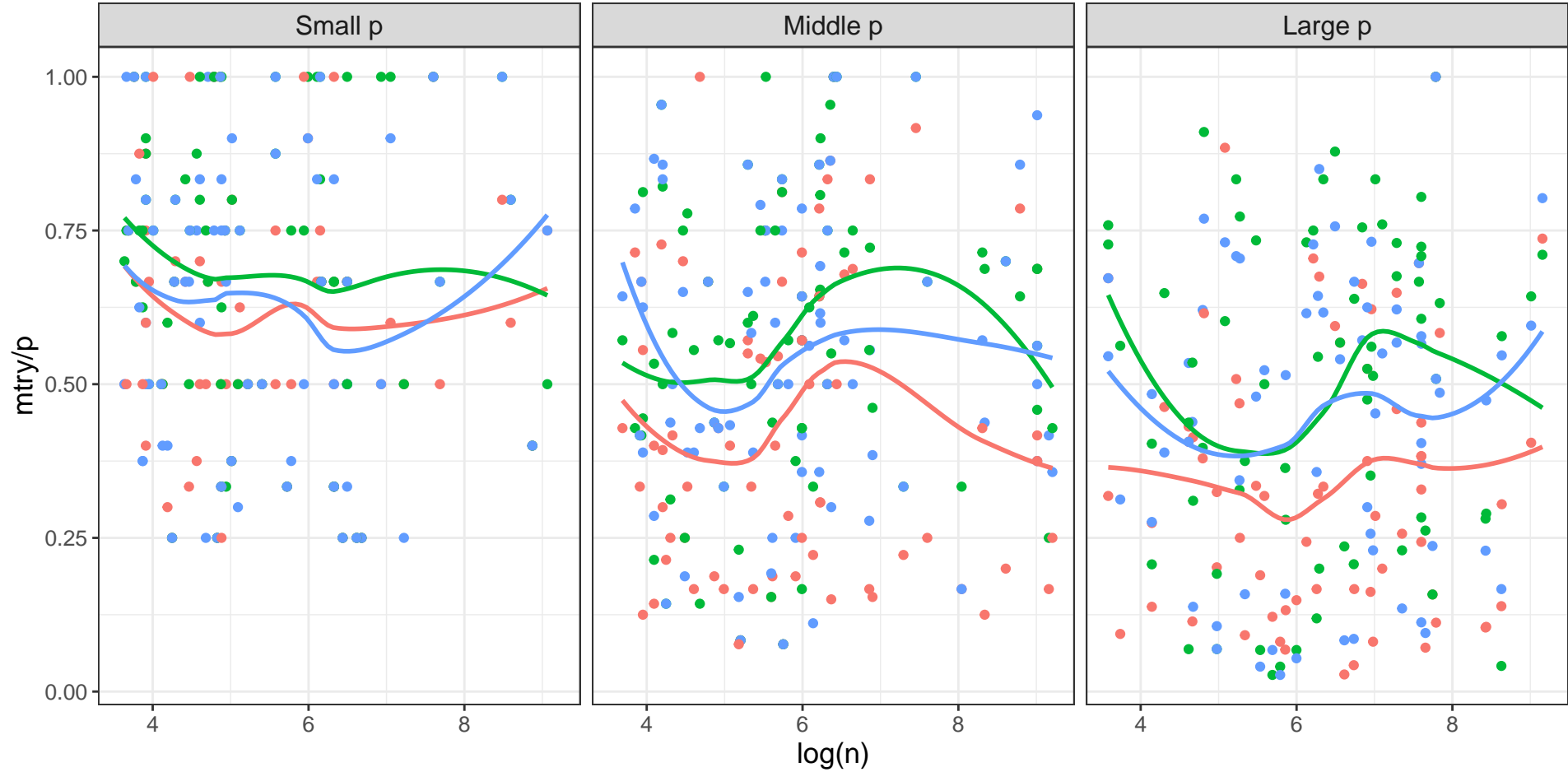

Supplement: Supplementary file 2 — Supplementary file1 (ZIP 108032 KB) [file 42979_2021_920_MOESM2_ESM.zip › Online_Resource_2/Results/Figures/InfluenceNstratified_brier.pdf]

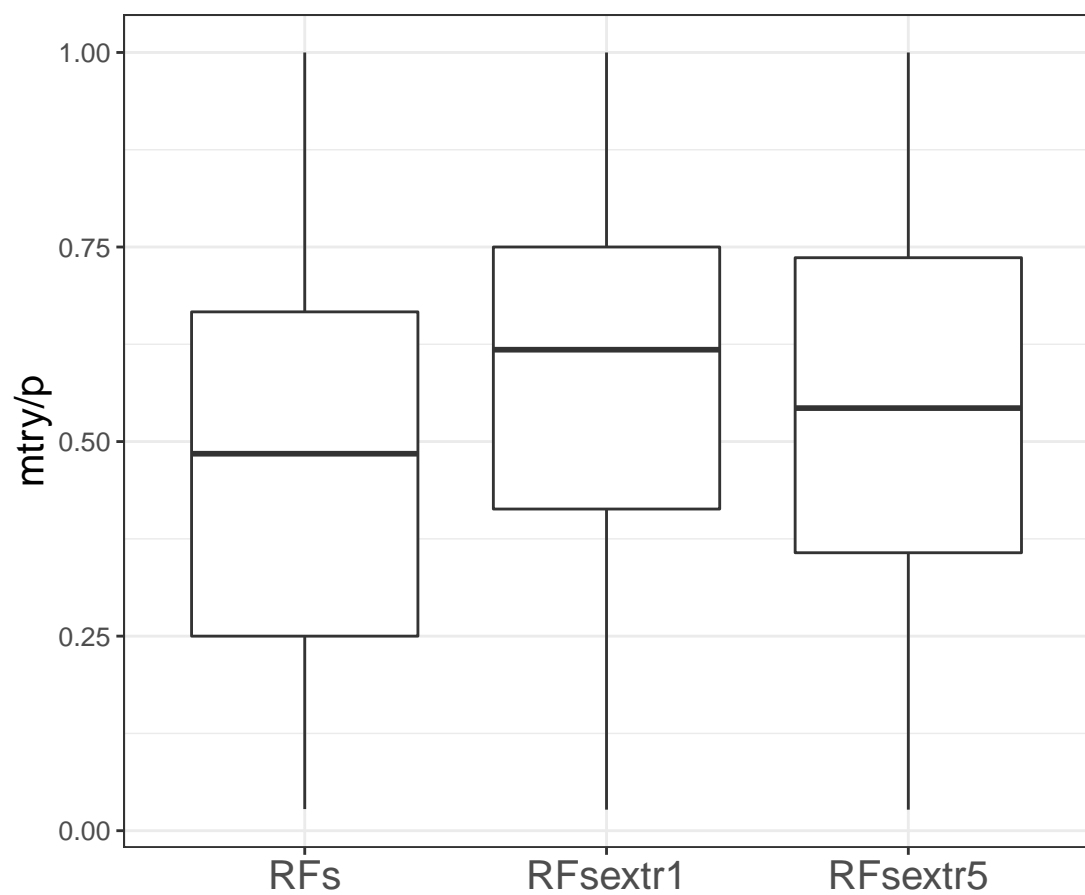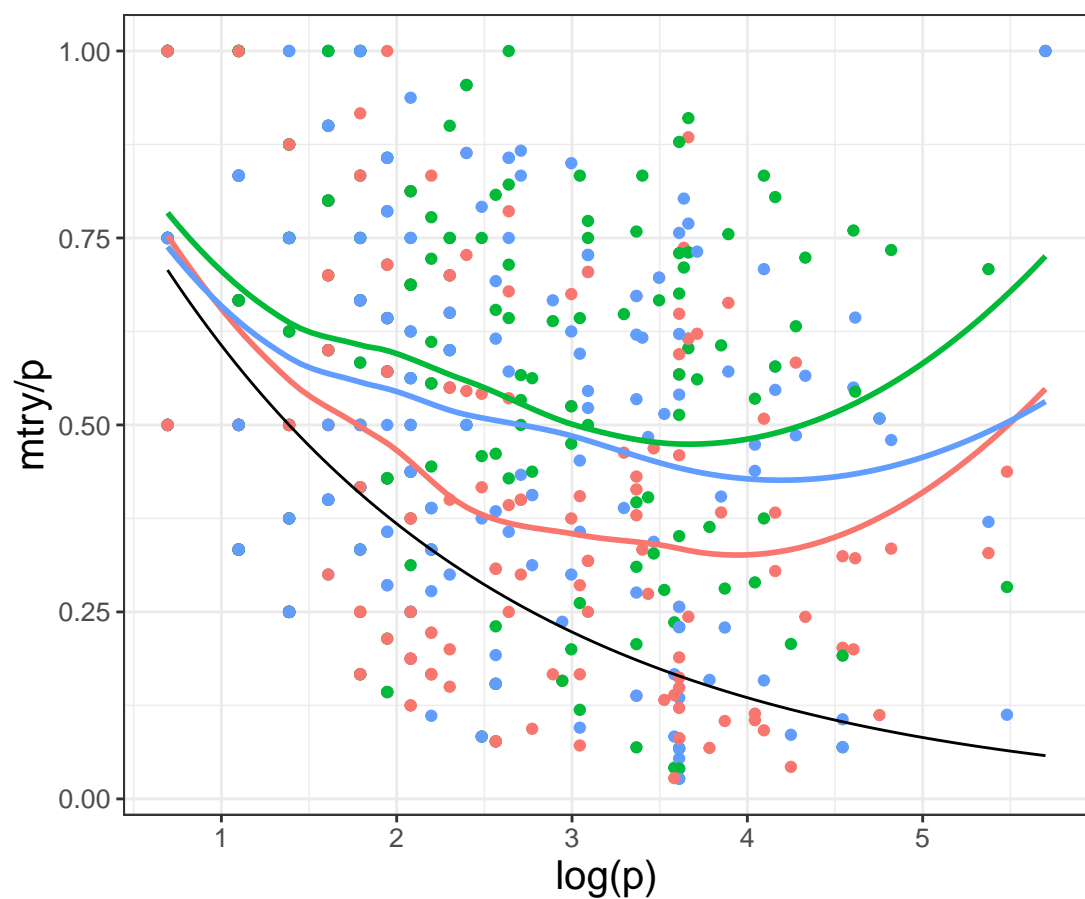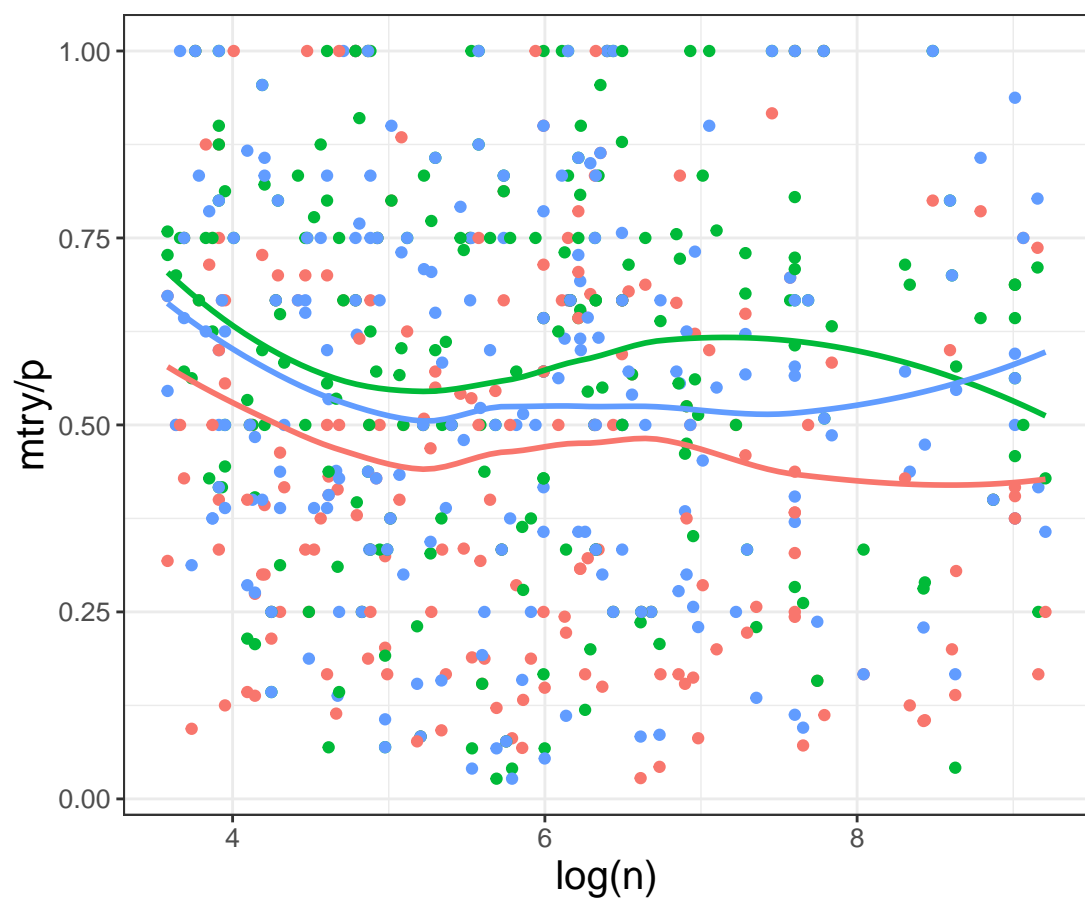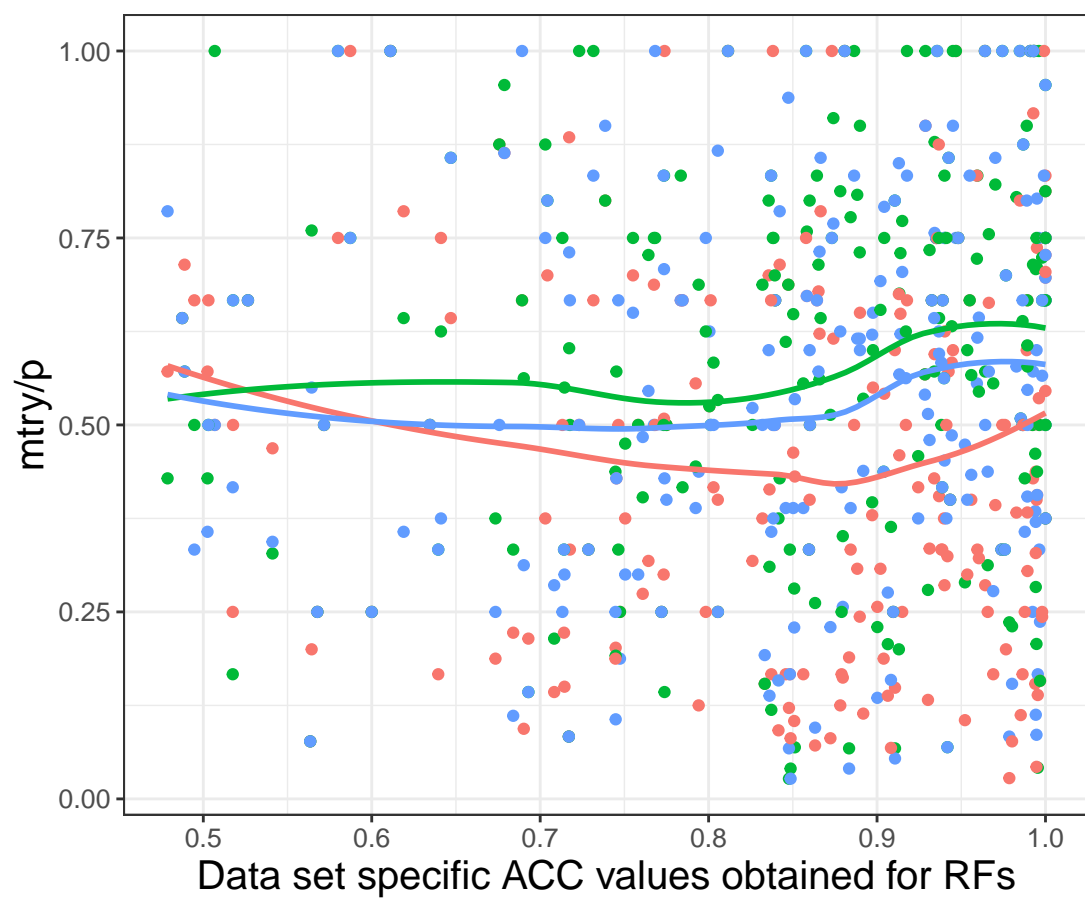

Supplement: Supplementary file 2 — Supplementary file1 (ZIP 108032 KB) [file 42979_2021_920_MOESM2_ESM.zip › Online_Resource_2/Results/Figures/Mtry.pdf]

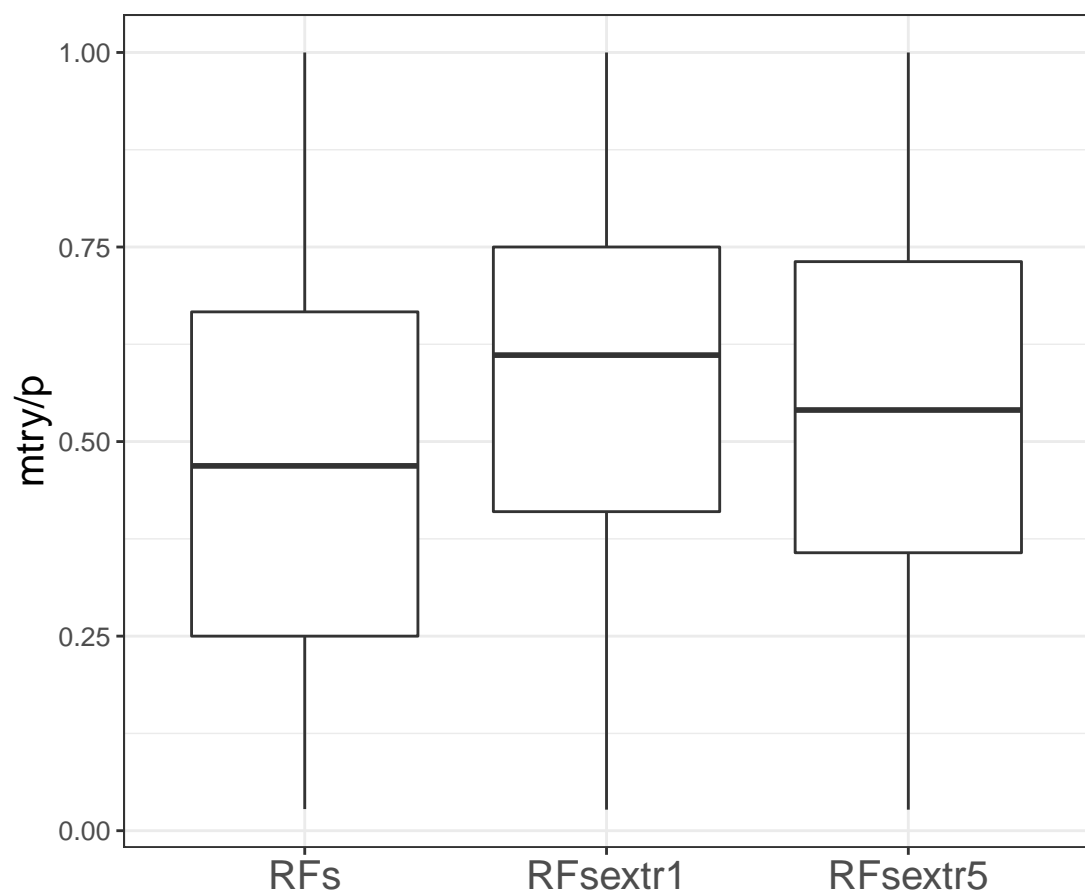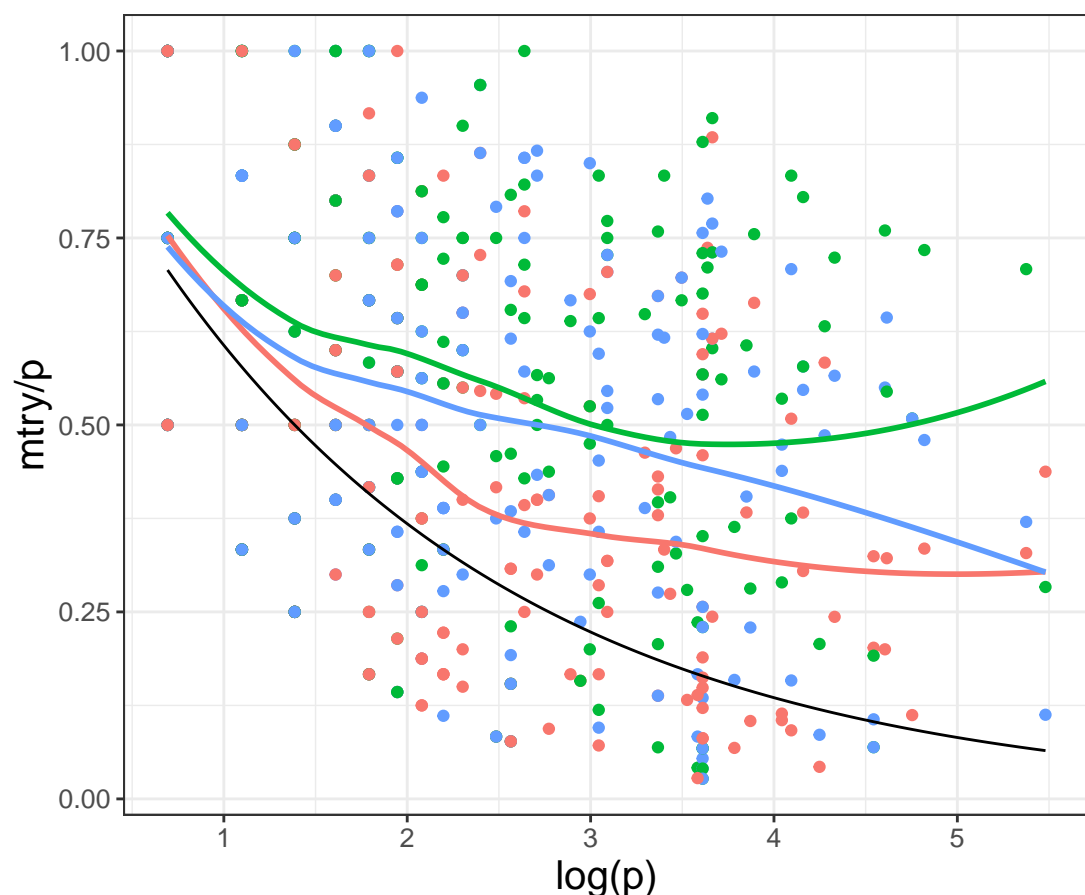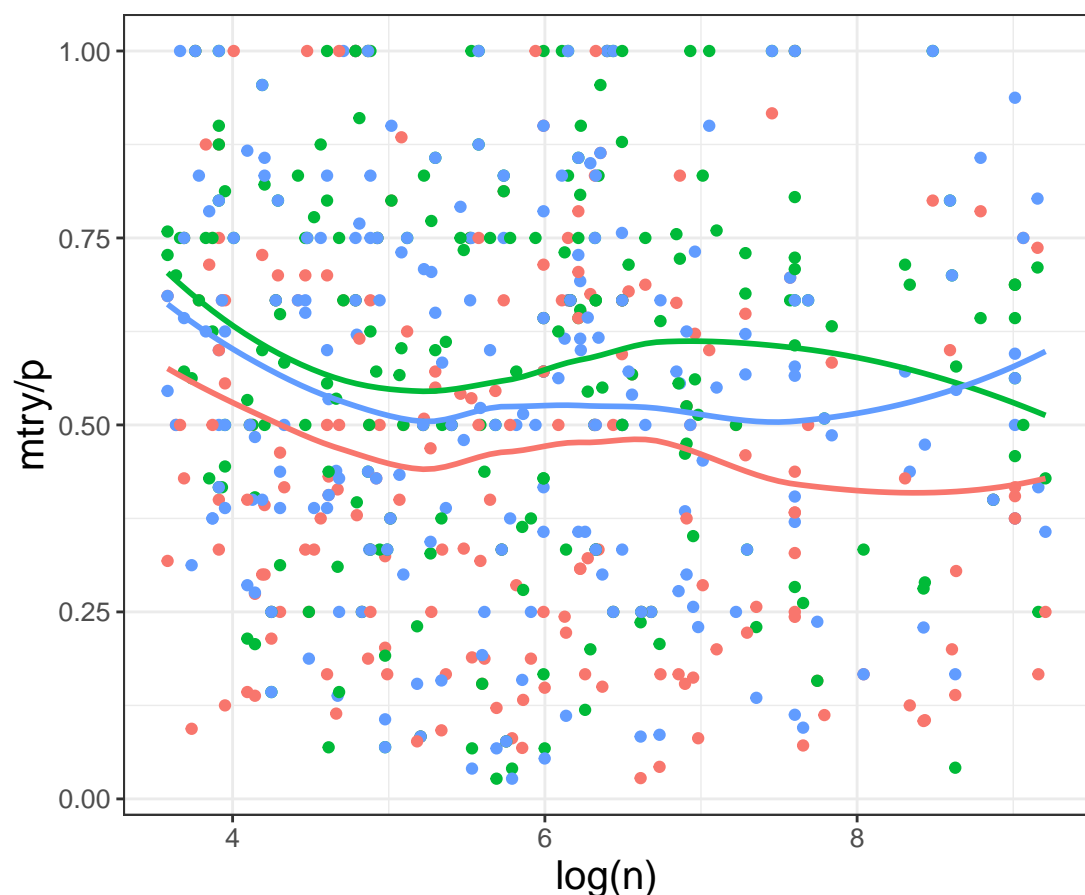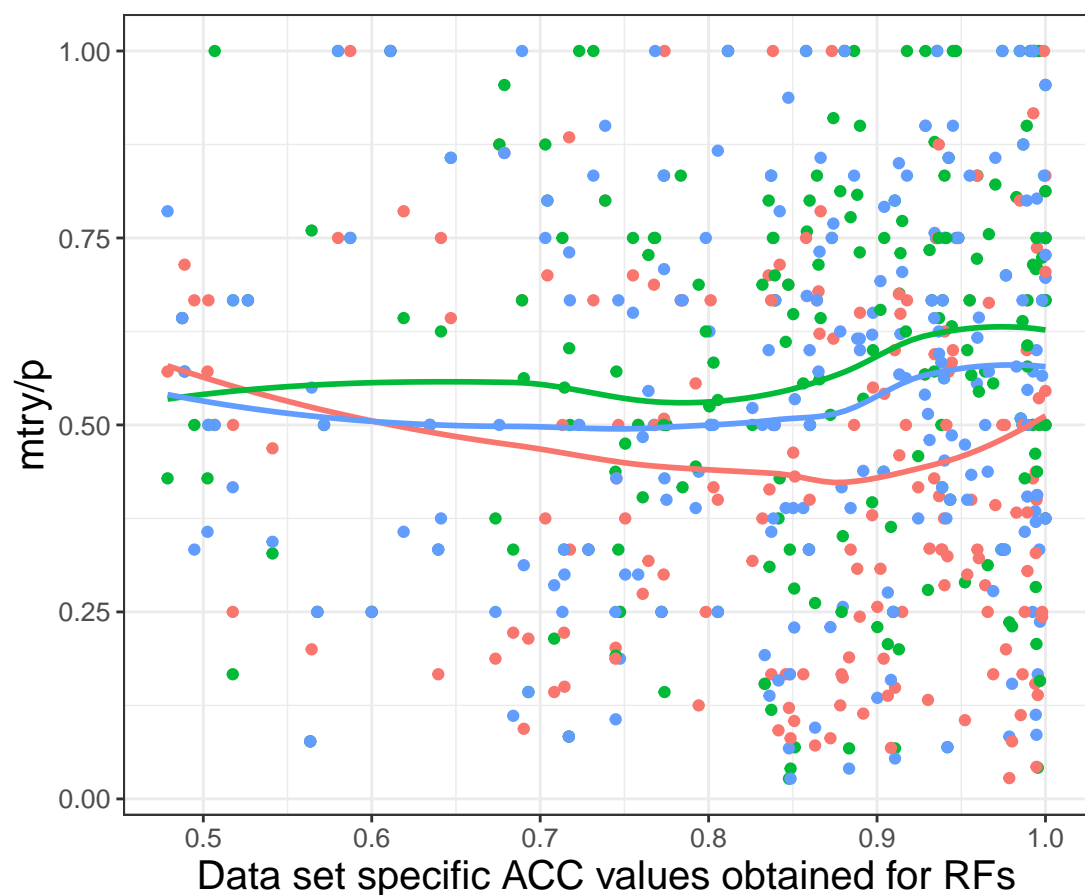

Supplement: Supplementary file 2 — Supplementary file1 (ZIP 108032 KB) [file 42979_2021_920_MOESM2_ESM.zip › Online_Resource_2/Results/Figures/Mtry_2.pdf]

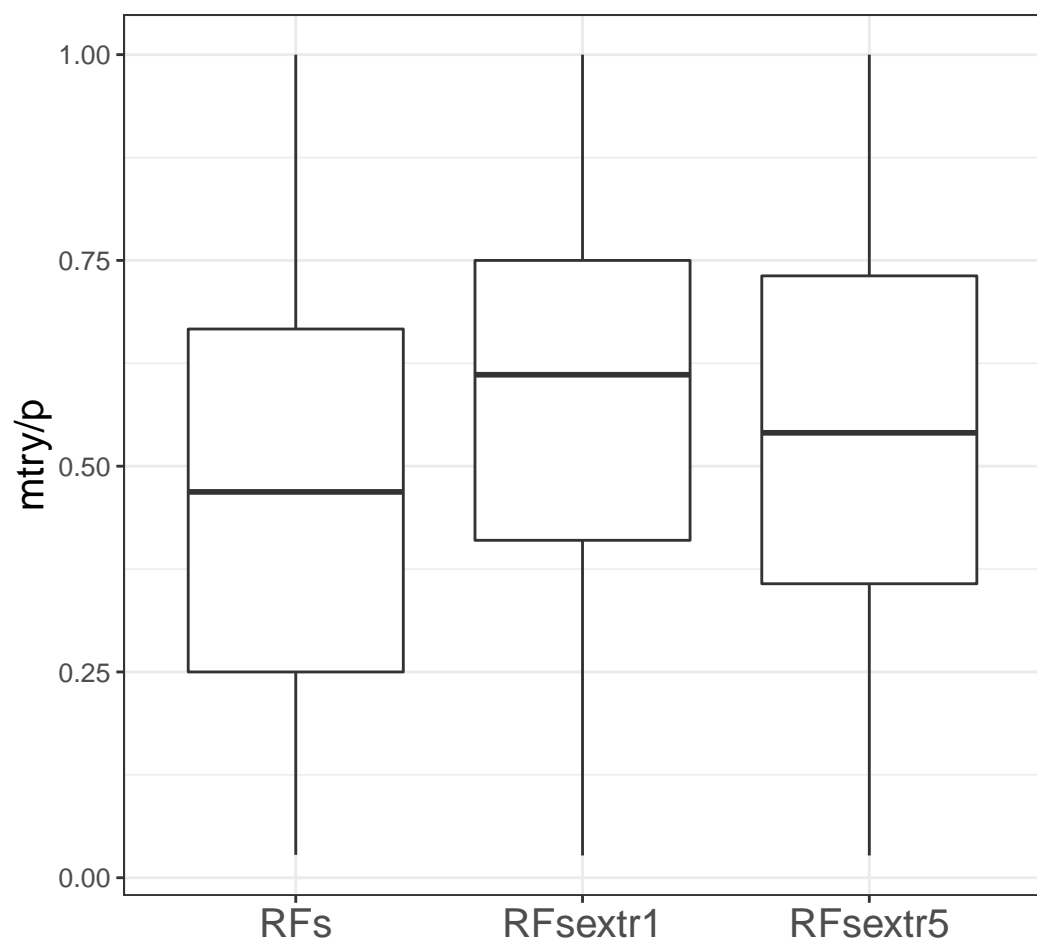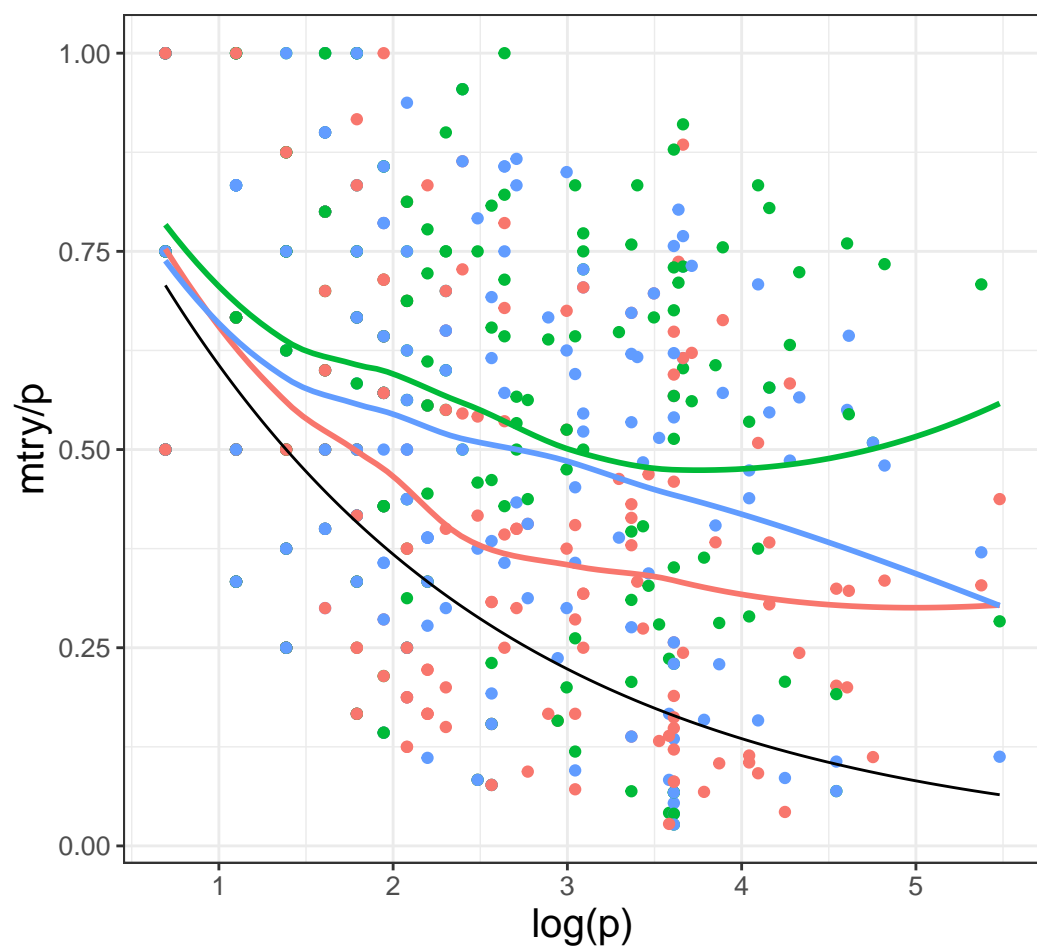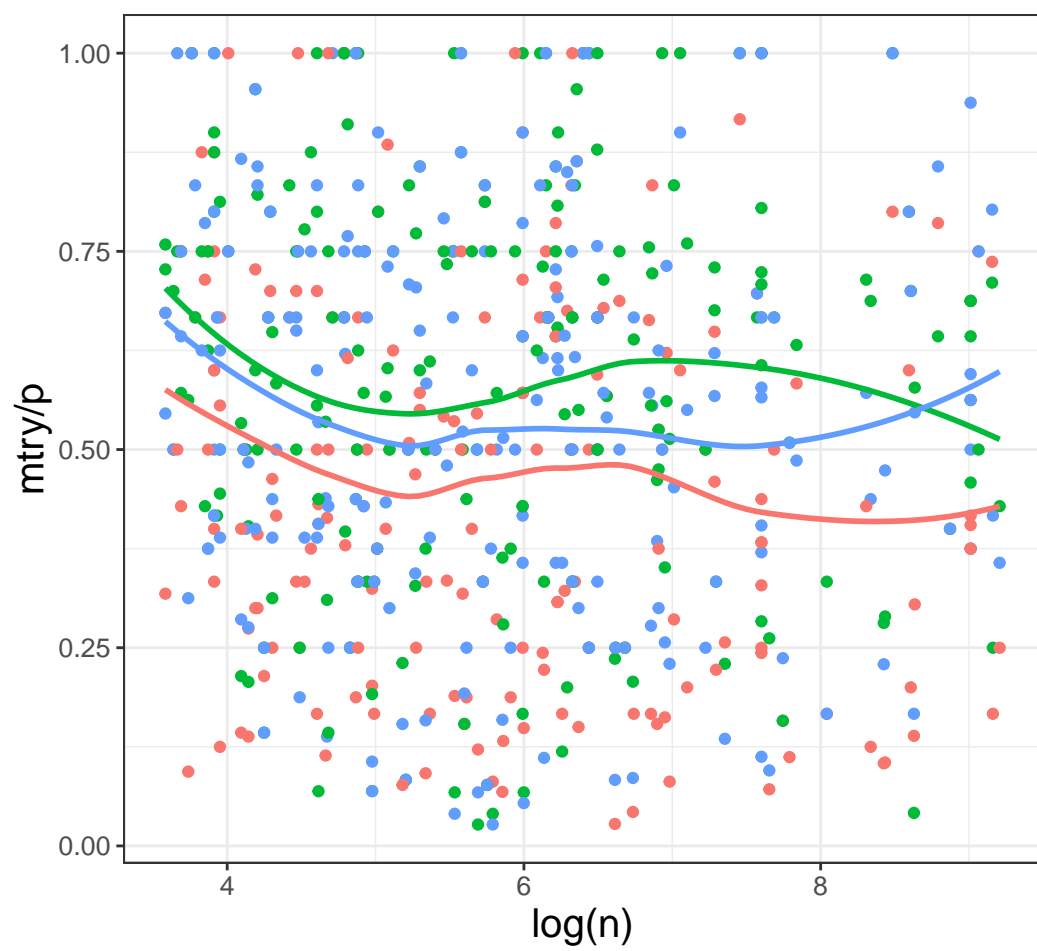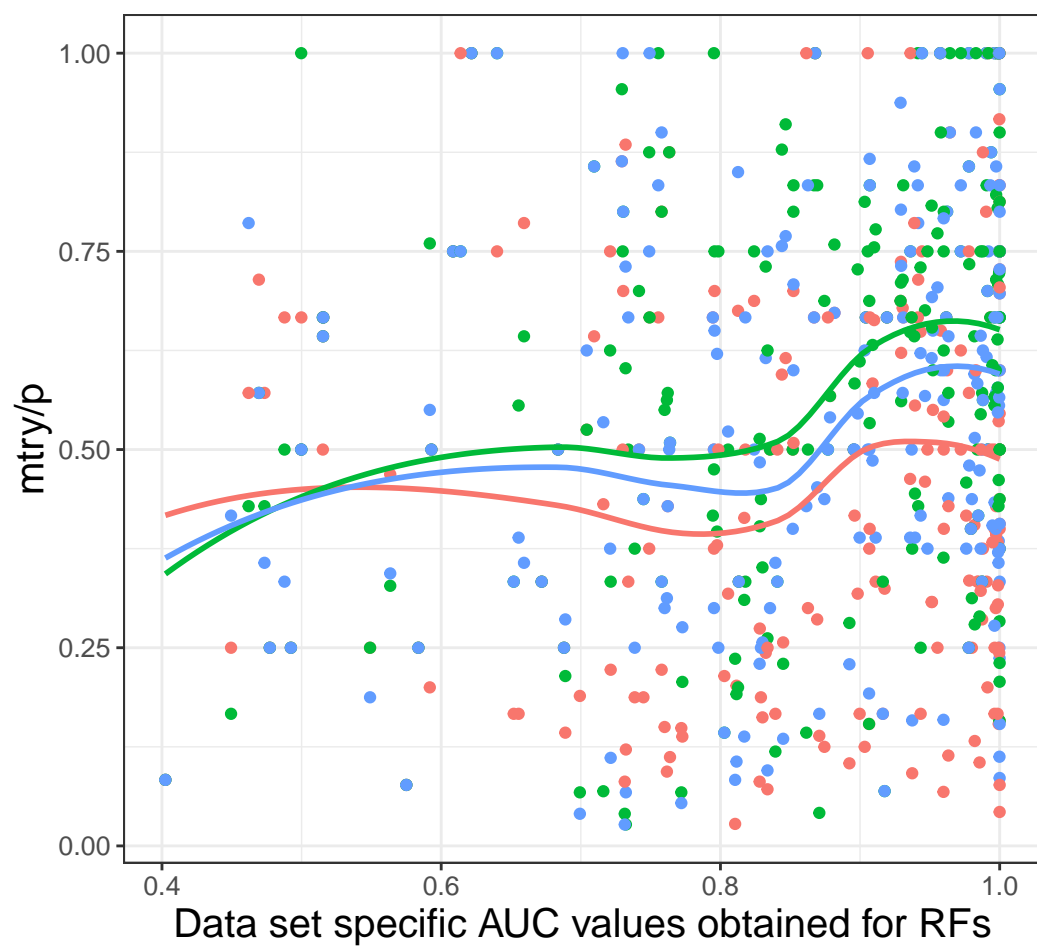

Supplement: Supplementary file 2 — Supplementary file1 (ZIP 108032 KB) [file 42979_2021_920_MOESM2_ESM.zip › Online_Resource_2/Results/Figures/Mtry_2_auc.pdf]

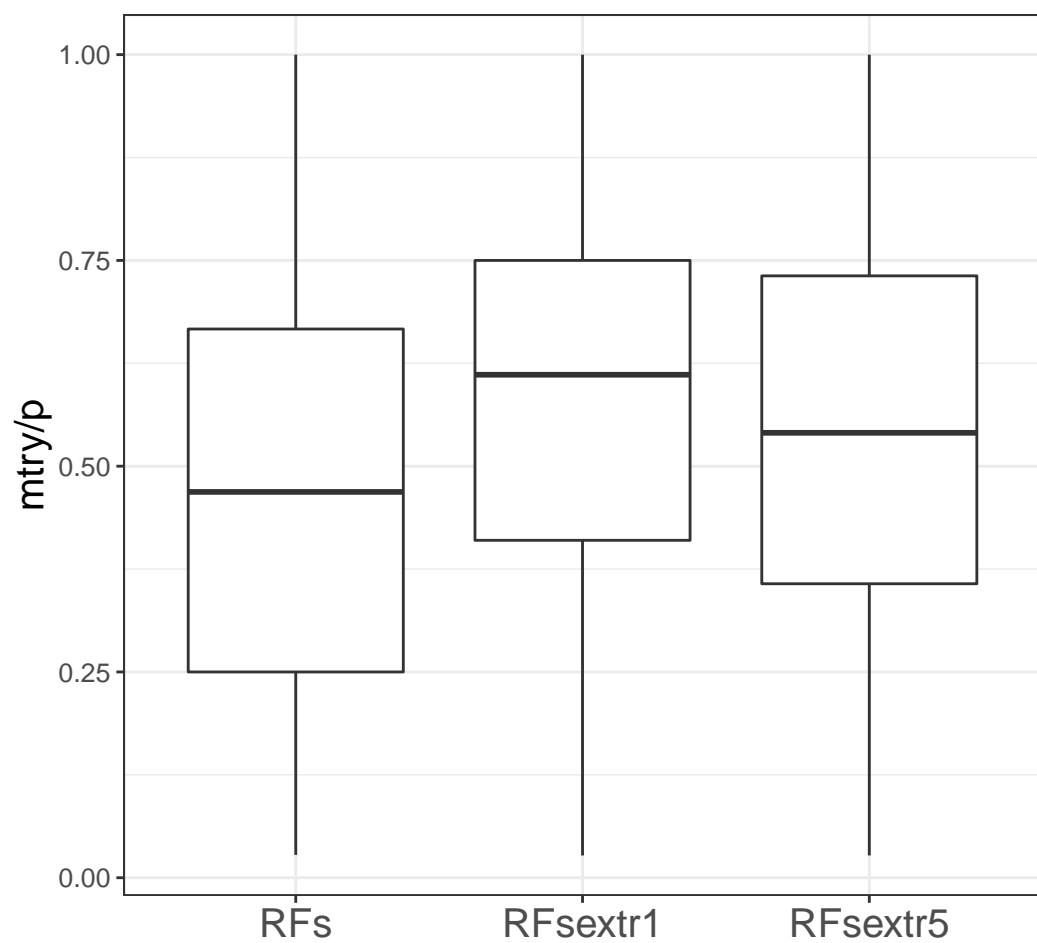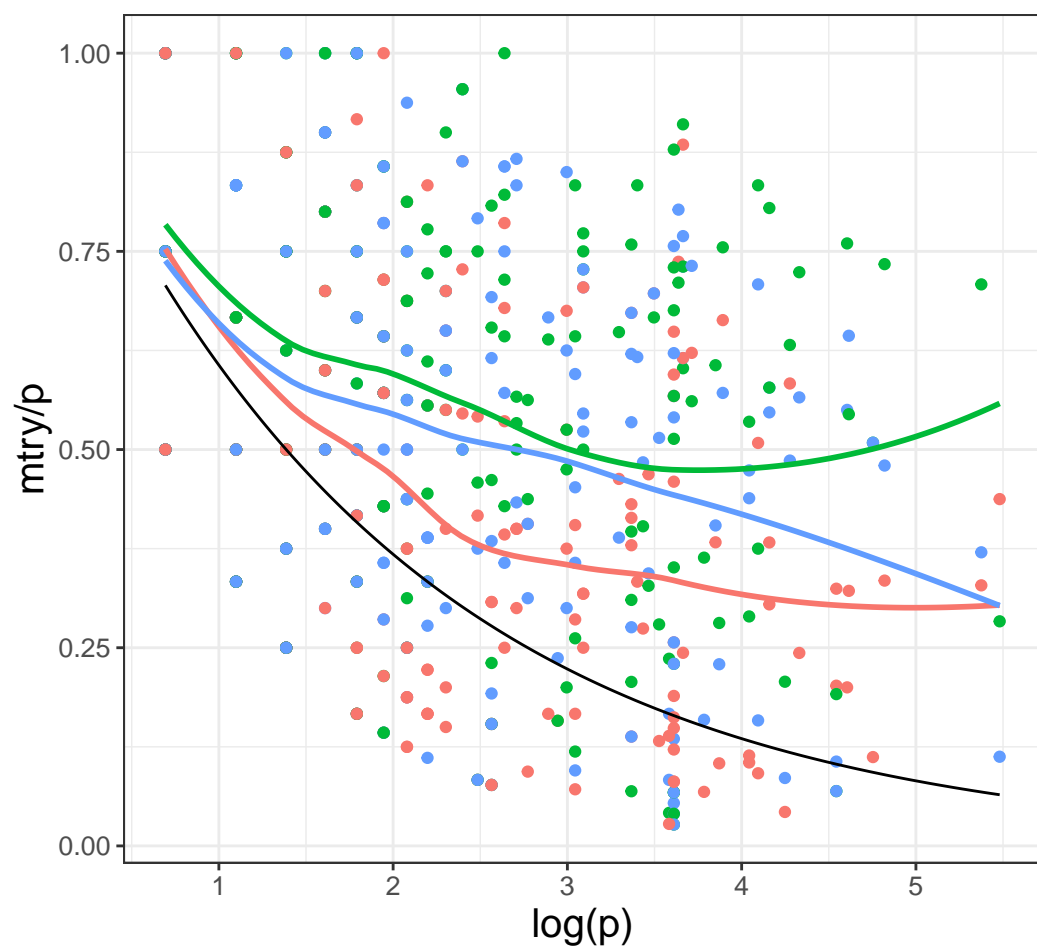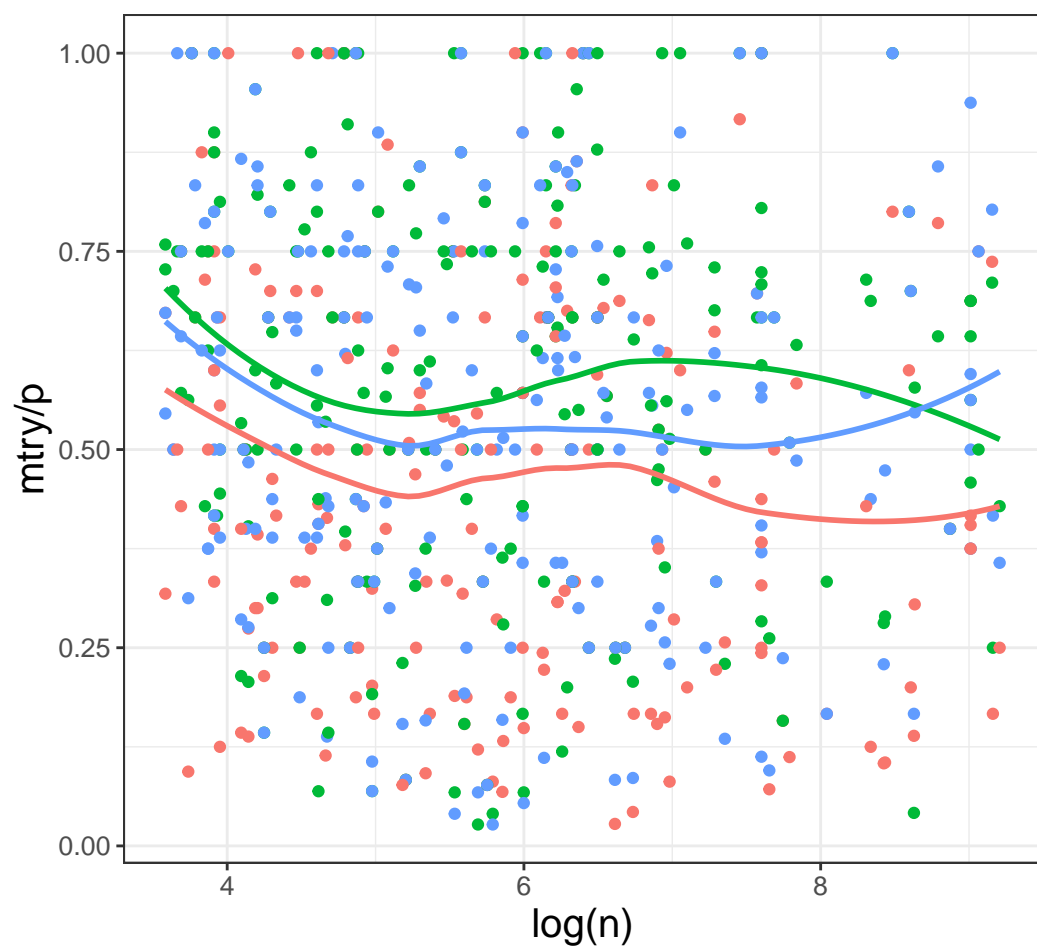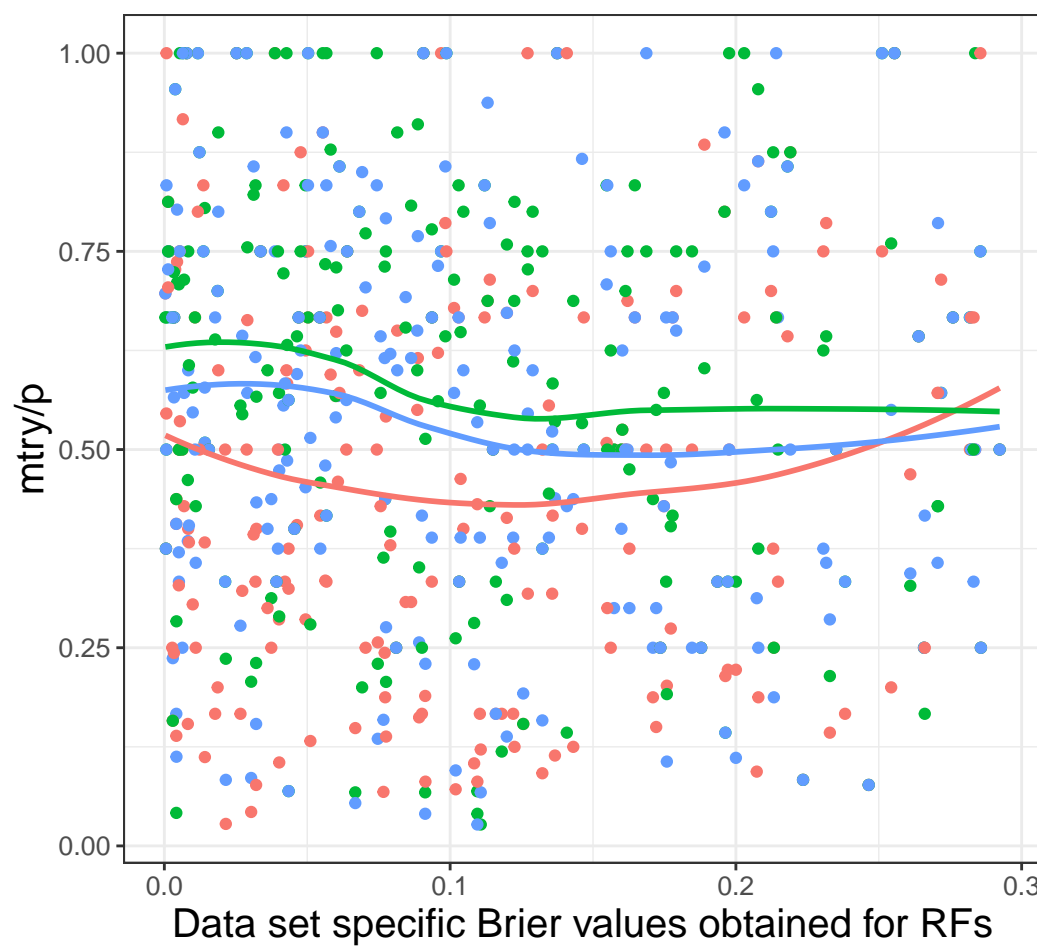

Supplement: Supplementary file 2 — Supplementary file1 (ZIP 108032 KB) [file 42979_2021_920_MOESM2_ESM.zip › Online_Resource_2/Results/Figures/Mtry_2_brier.pdf]

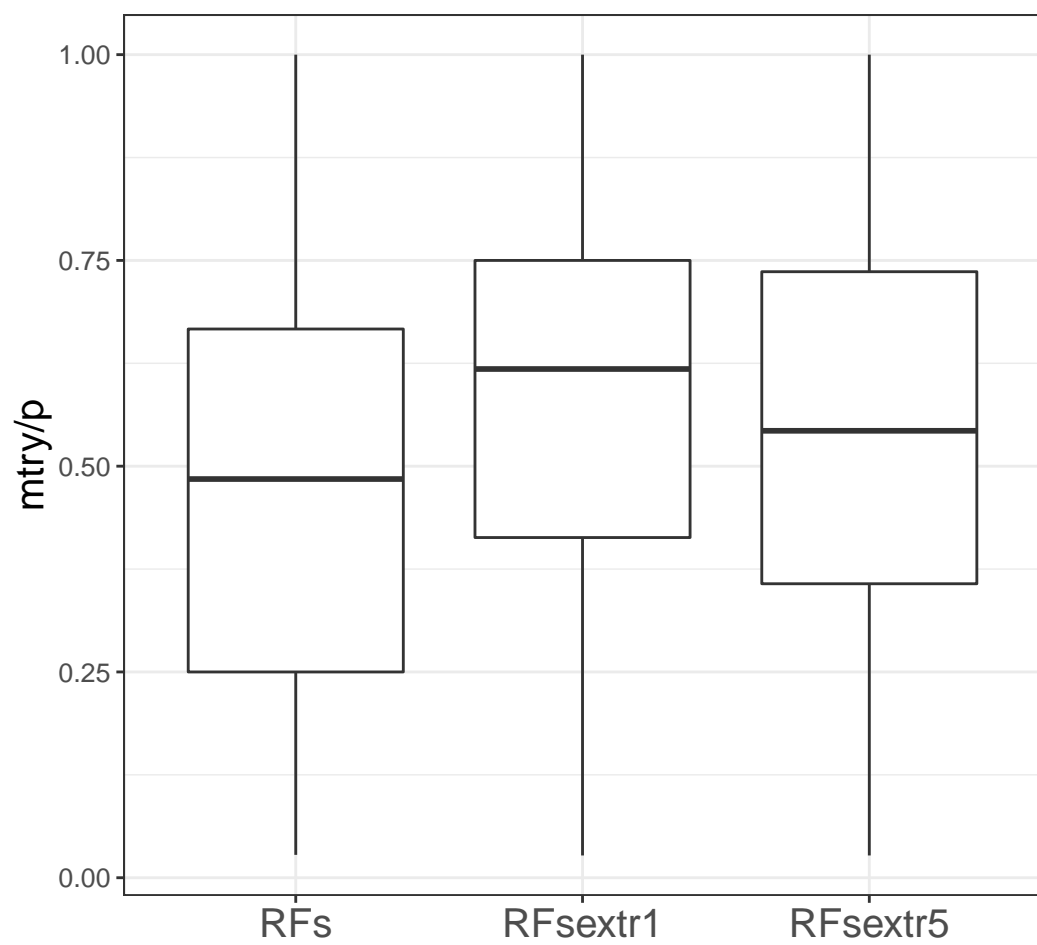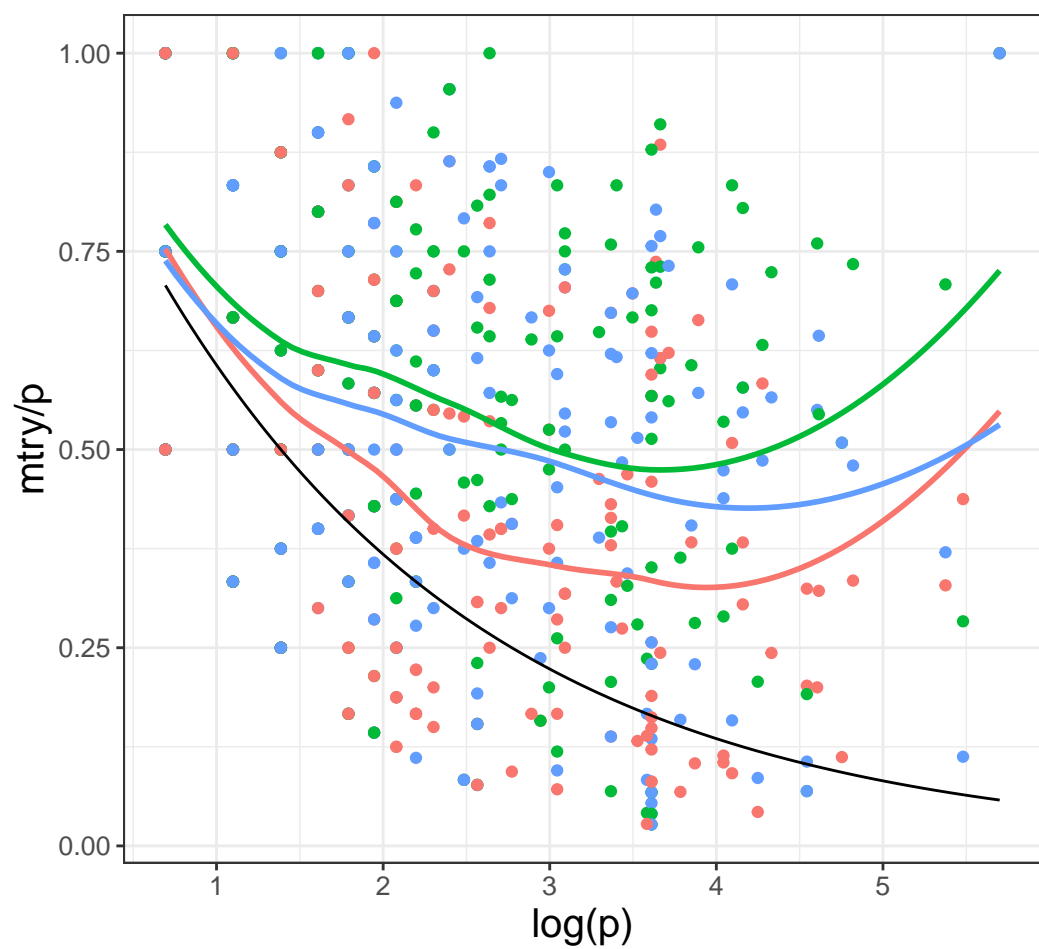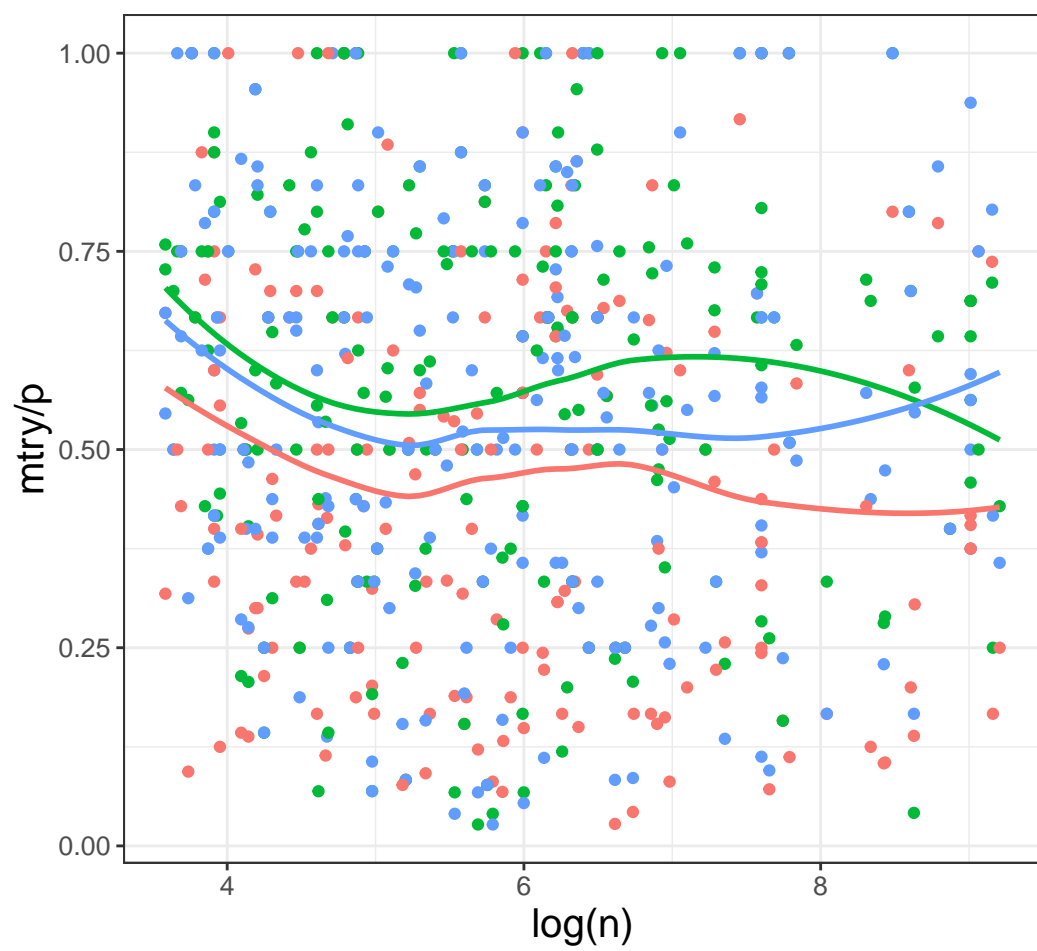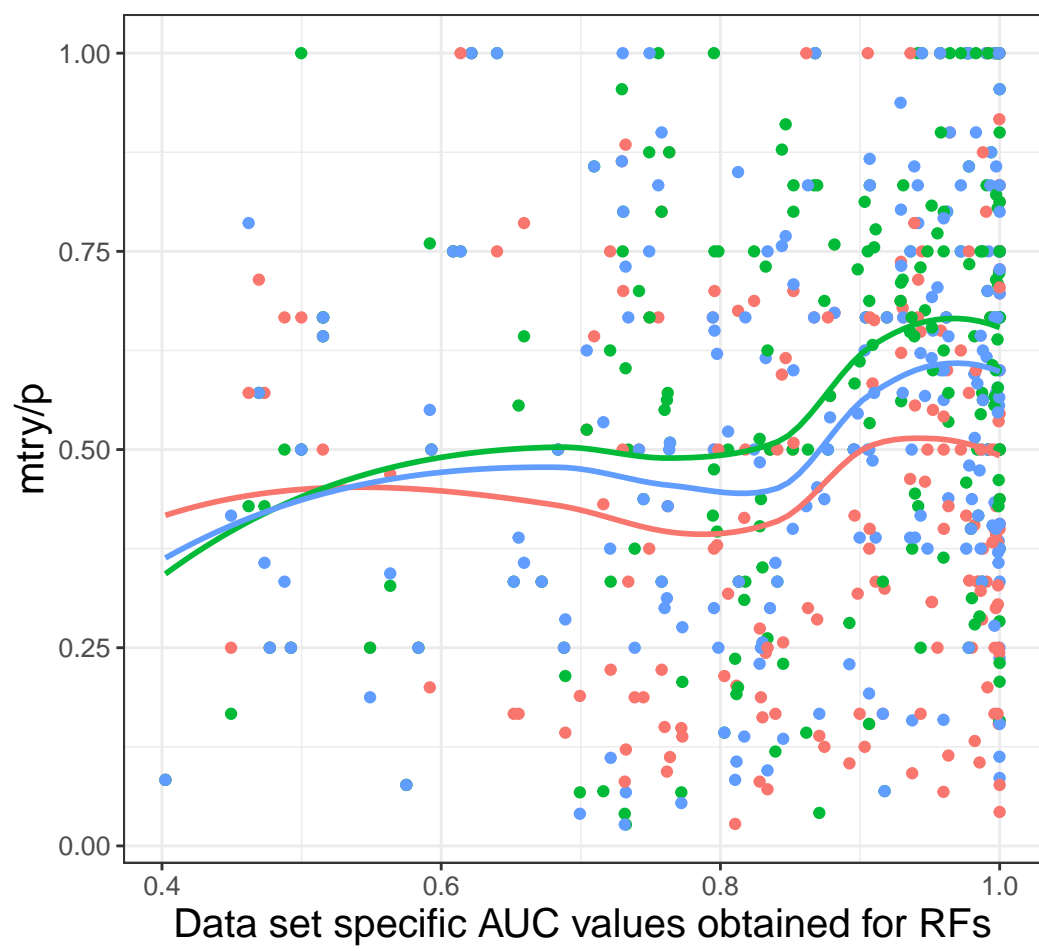

Supplement: Supplementary file 2 — Supplementary file1 (ZIP 108032 KB) [file 42979_2021_920_MOESM2_ESM.zip › Online_Resource_2/Results/Figures/Mtry_auc.pdf]

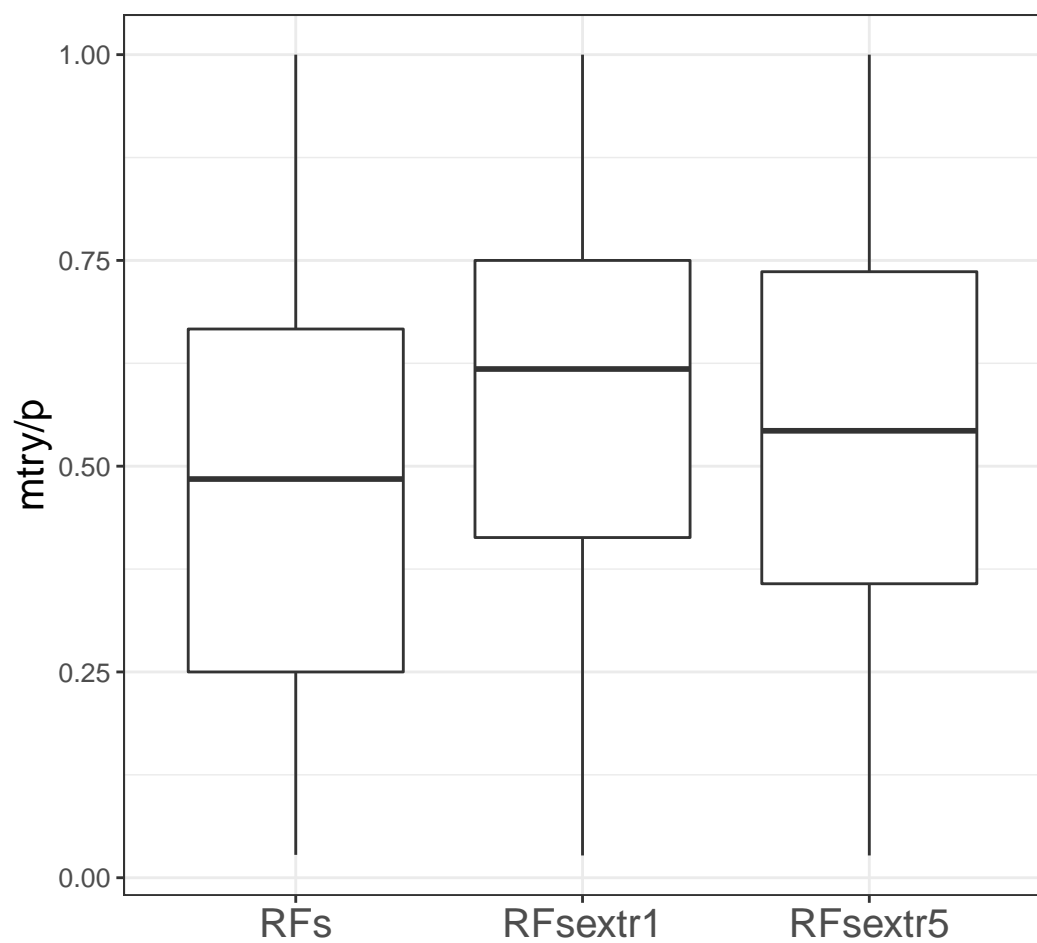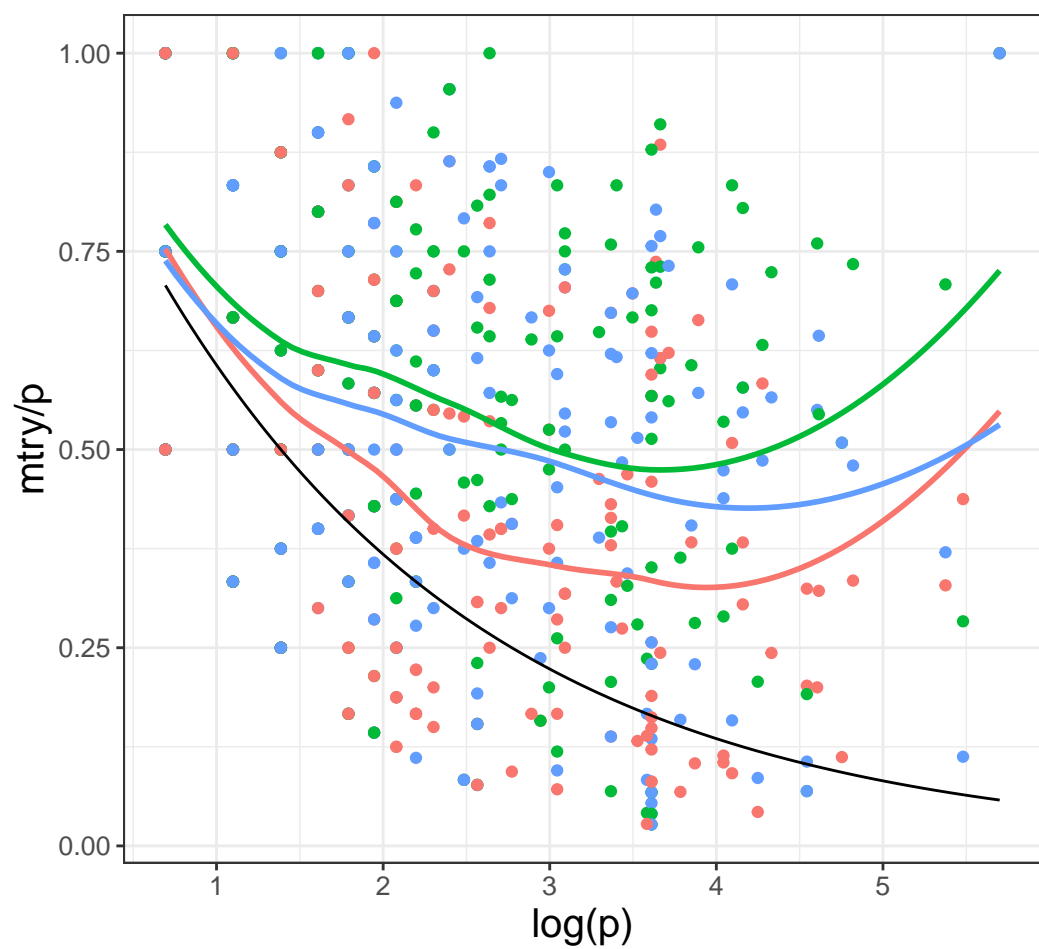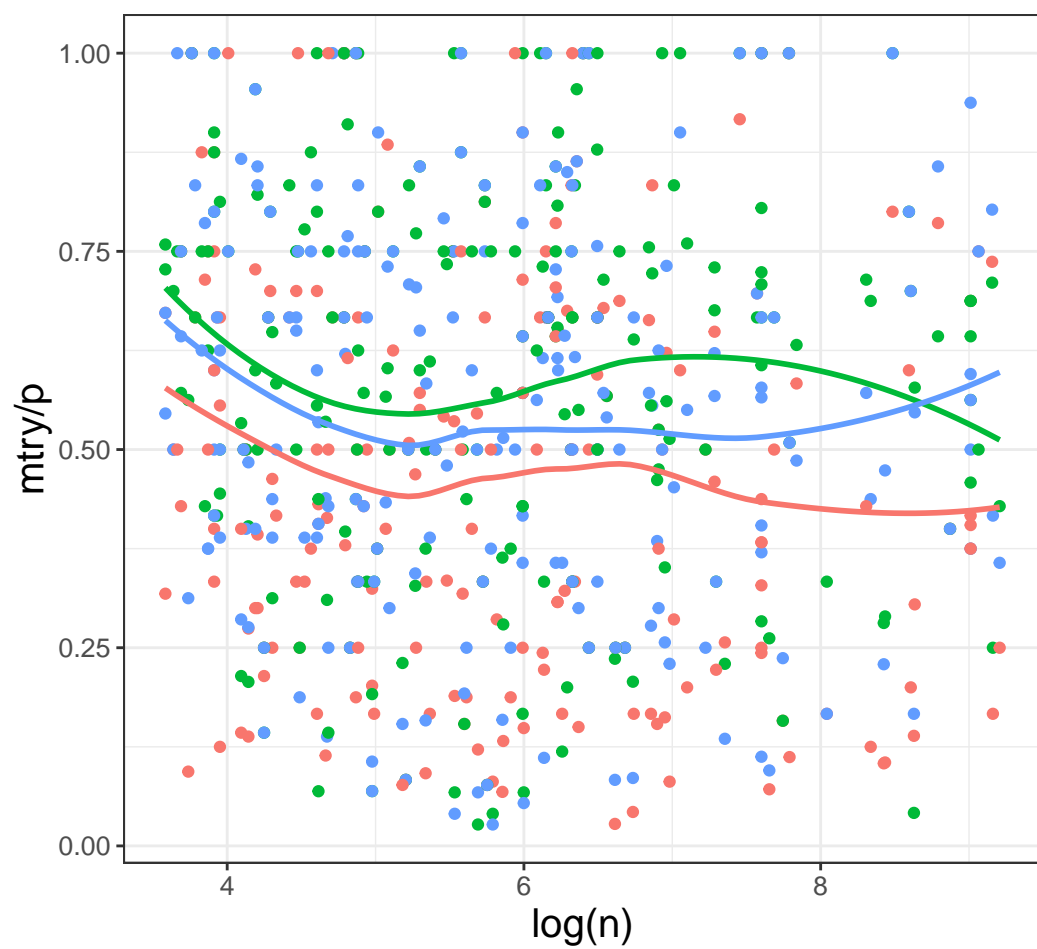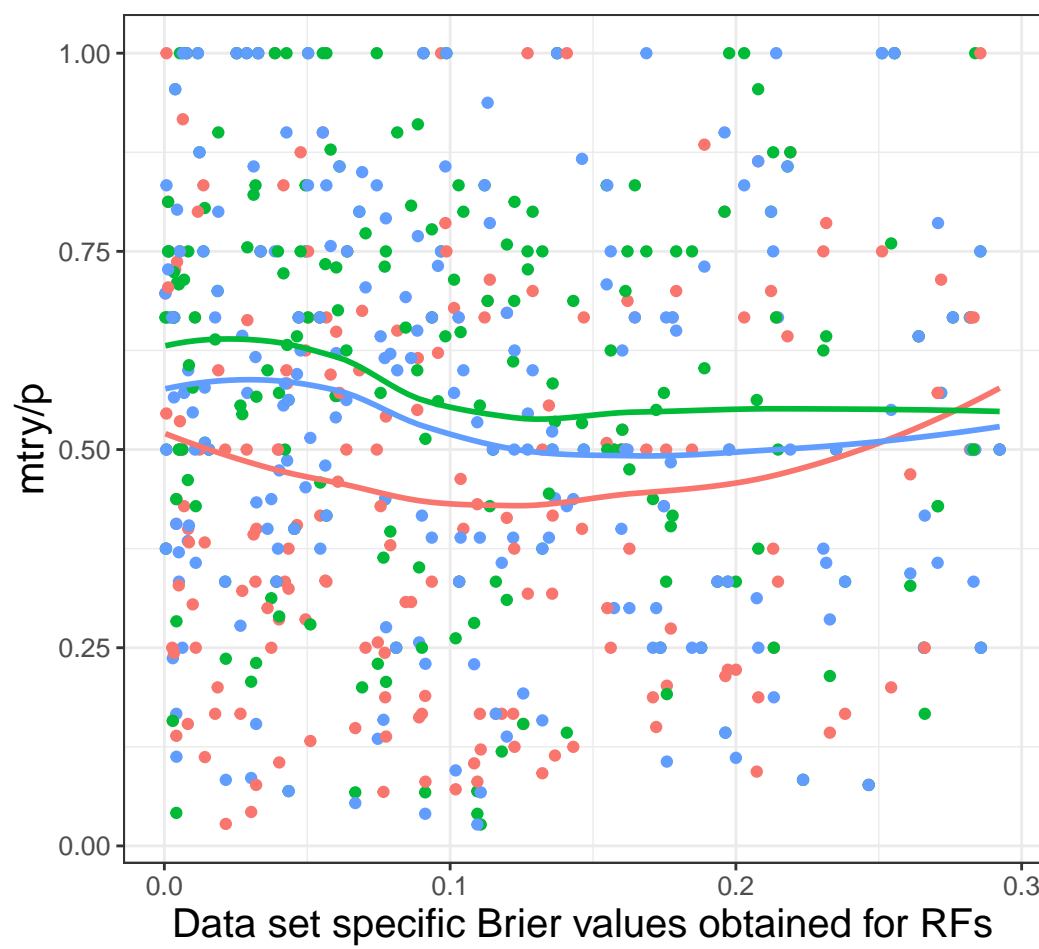

Supplement: Supplementary file 2 — Supplementary file1 (ZIP 108032 KB) [file 42979_2021_920_MOESM2_ESM.zip › Online_Resource_2/Results/Figures/Mtry_brier.pdf]

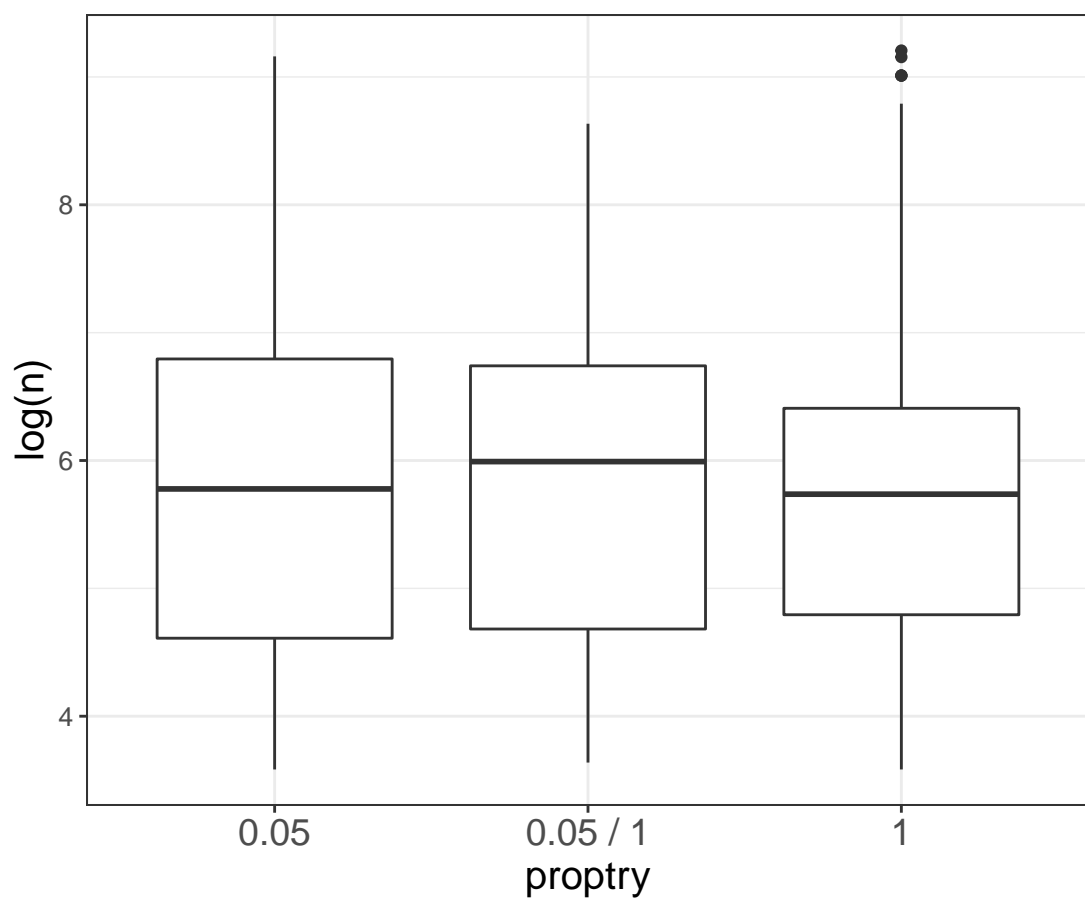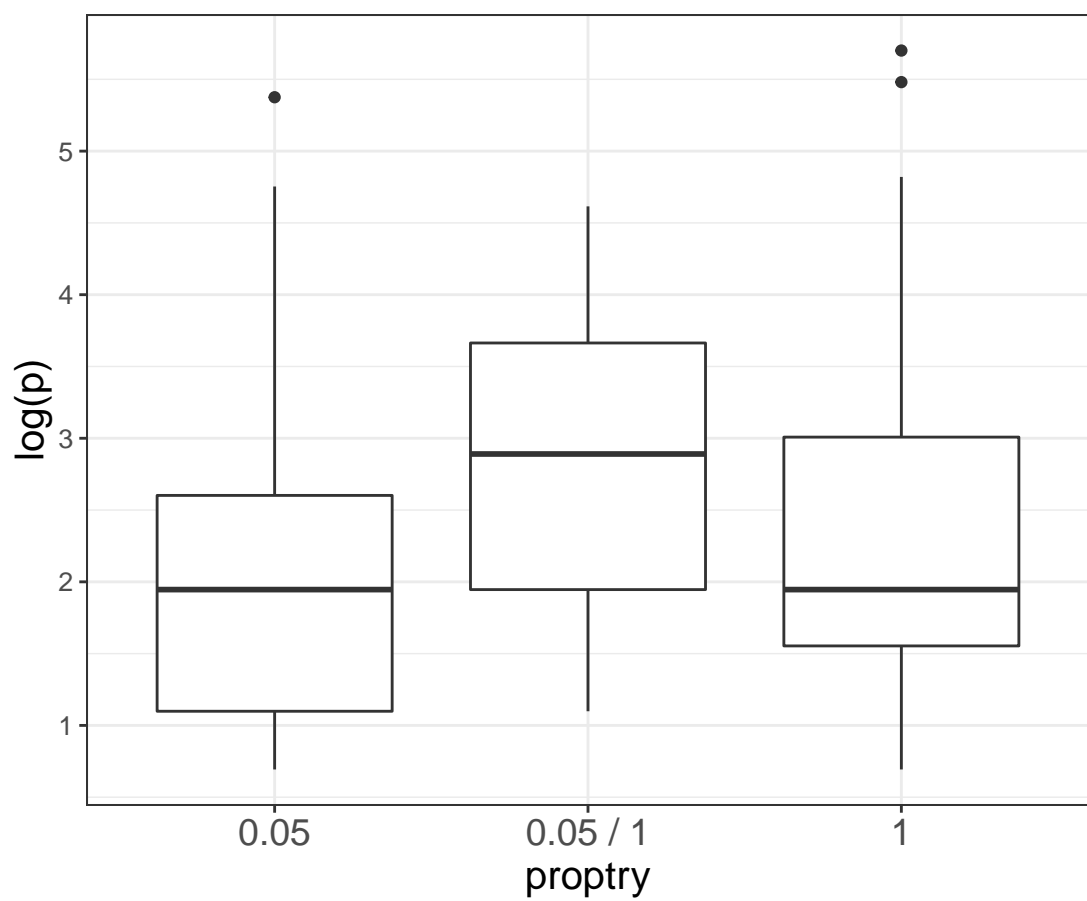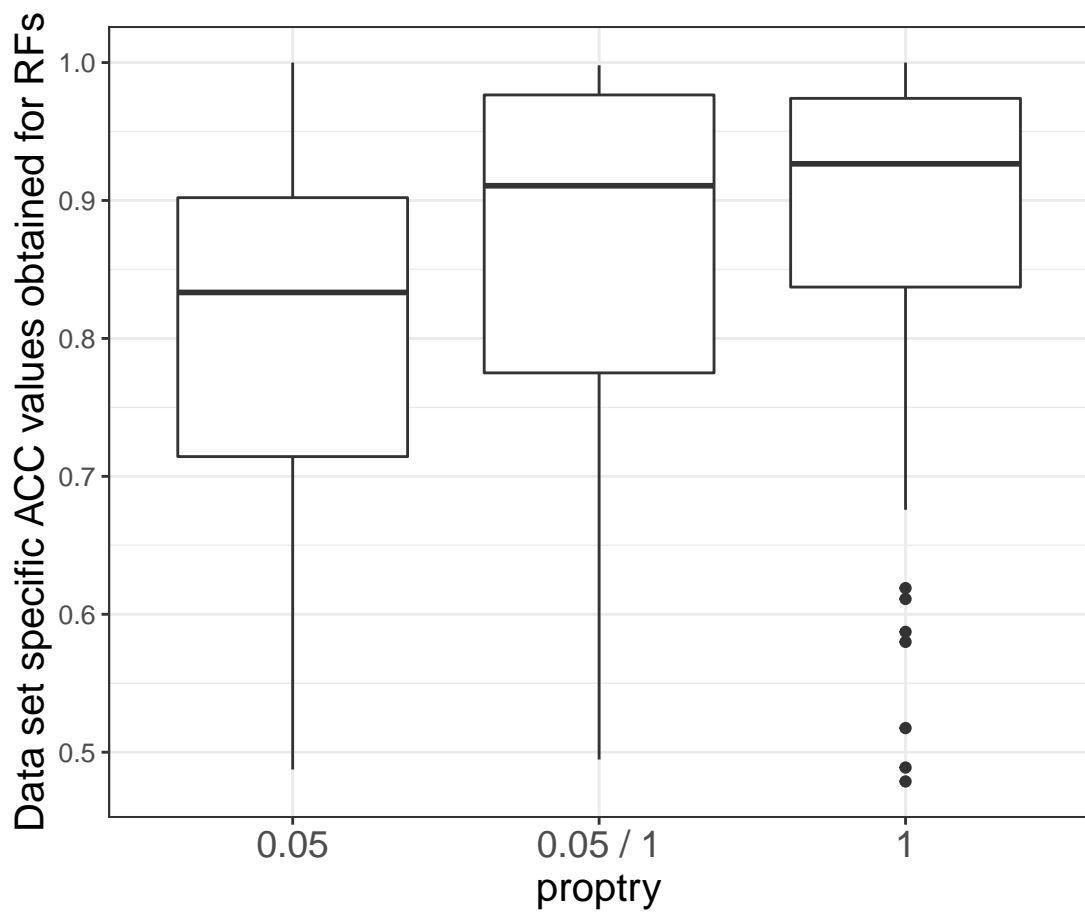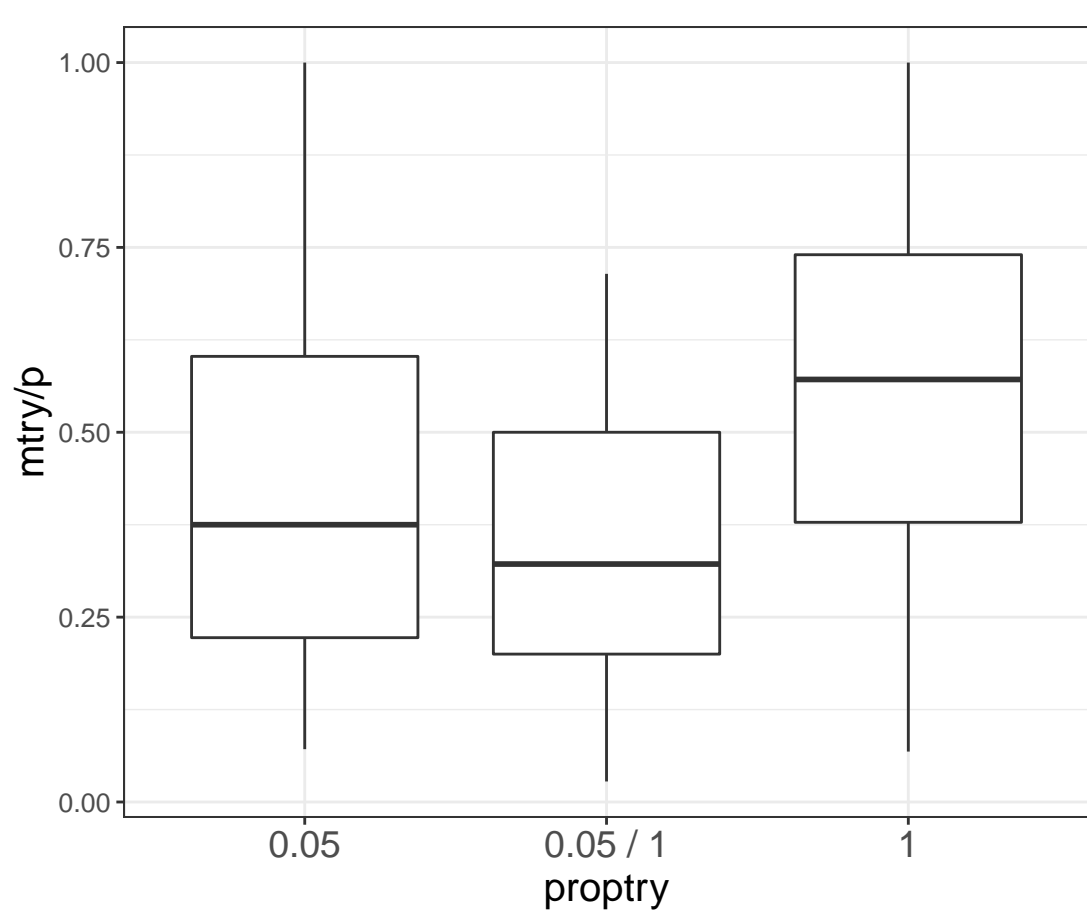

Supplement: Supplementary file 2 — Supplementary file1 (ZIP 108032 KB) [file 42979_2021_920_MOESM2_ESM.zip › Online_Resource_2/Results/Figures/Proptry.pdf]

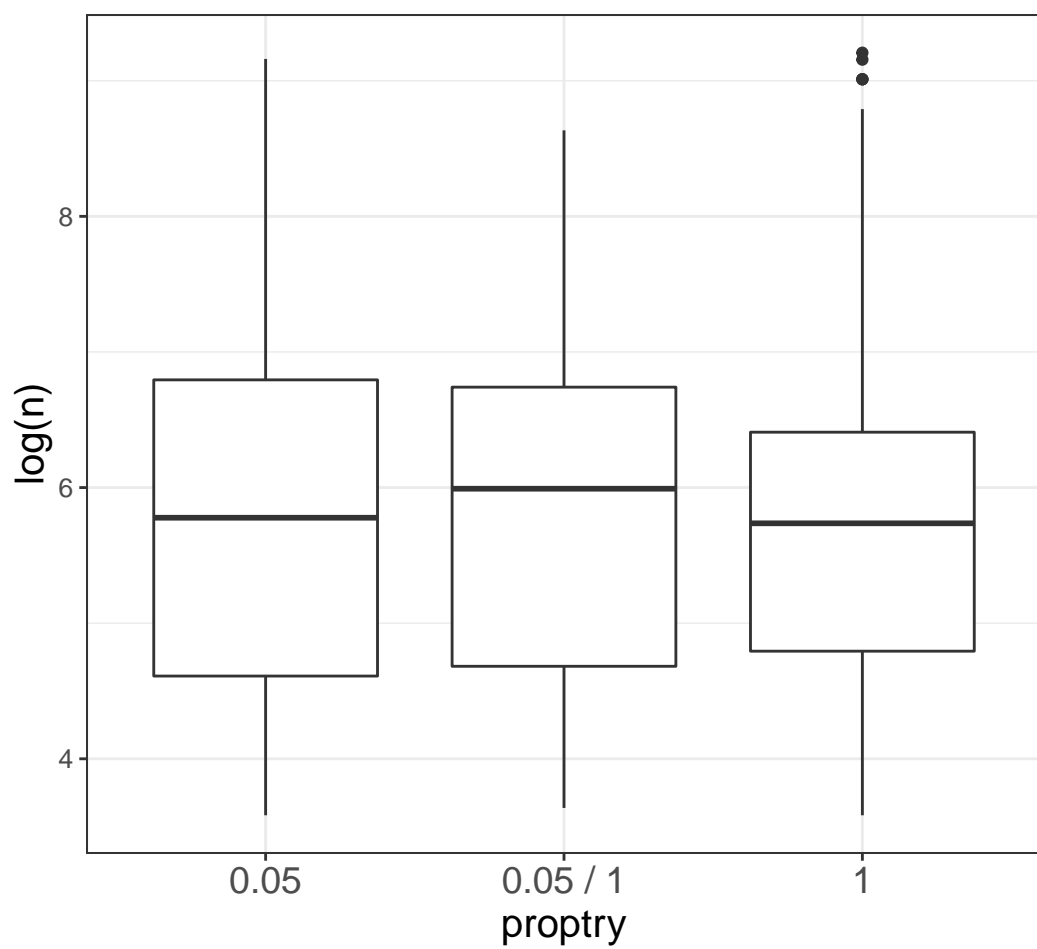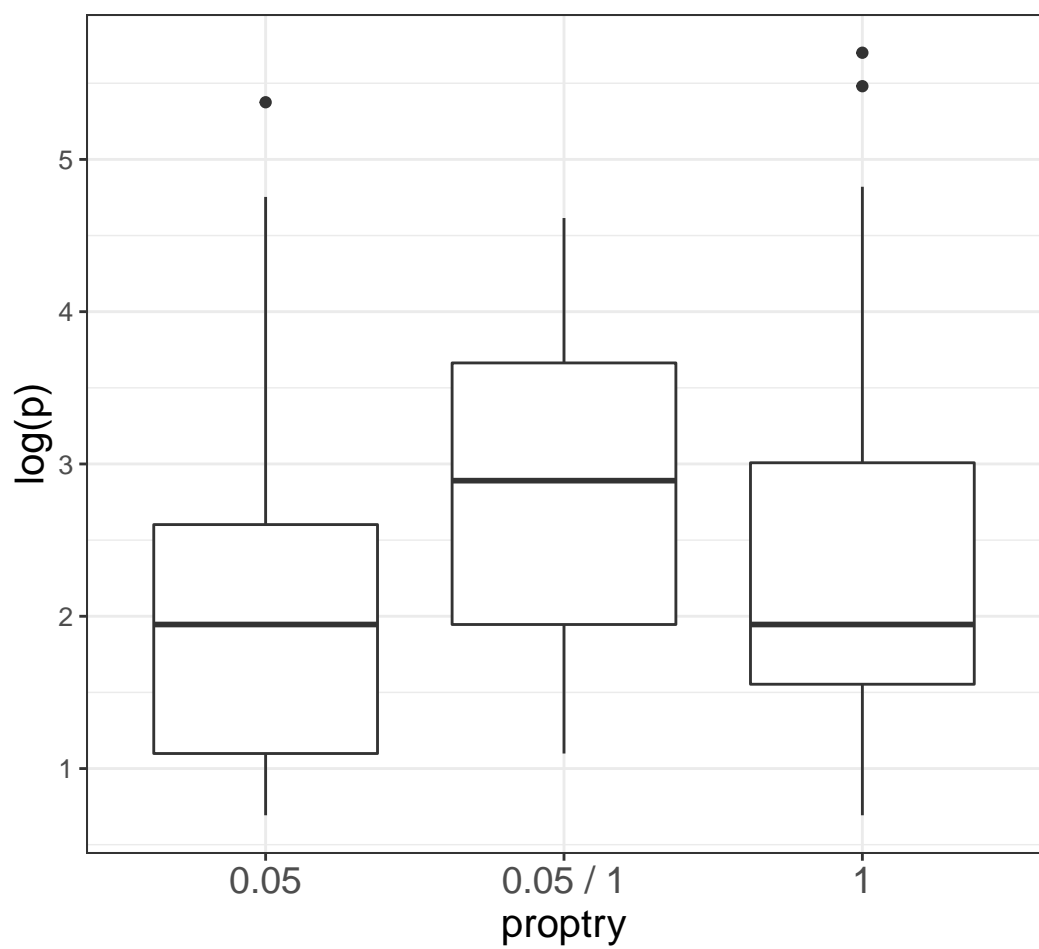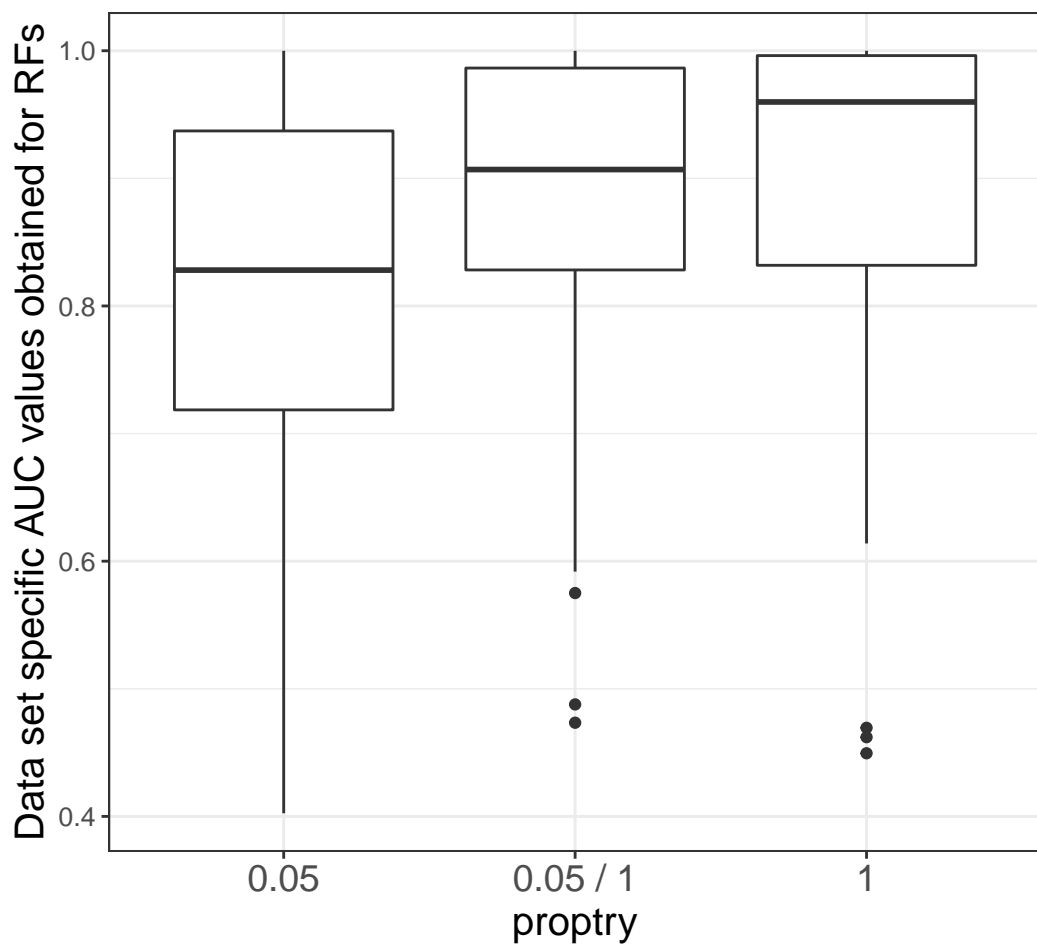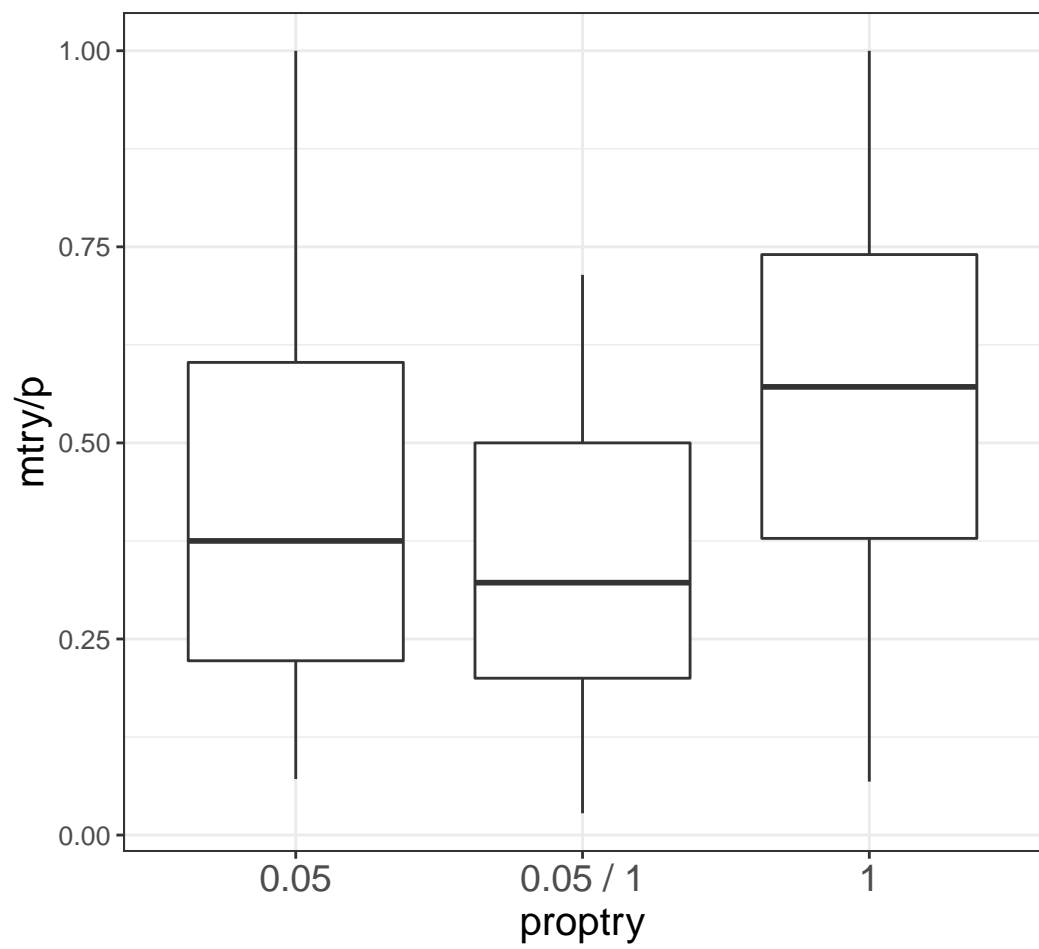

Supplement: Supplementary file 2 — Supplementary file1 (ZIP 108032 KB) [file 42979_2021_920_MOESM2_ESM.zip › Online_Resource_2/Results/Figures/Proptry_auc.pdf]

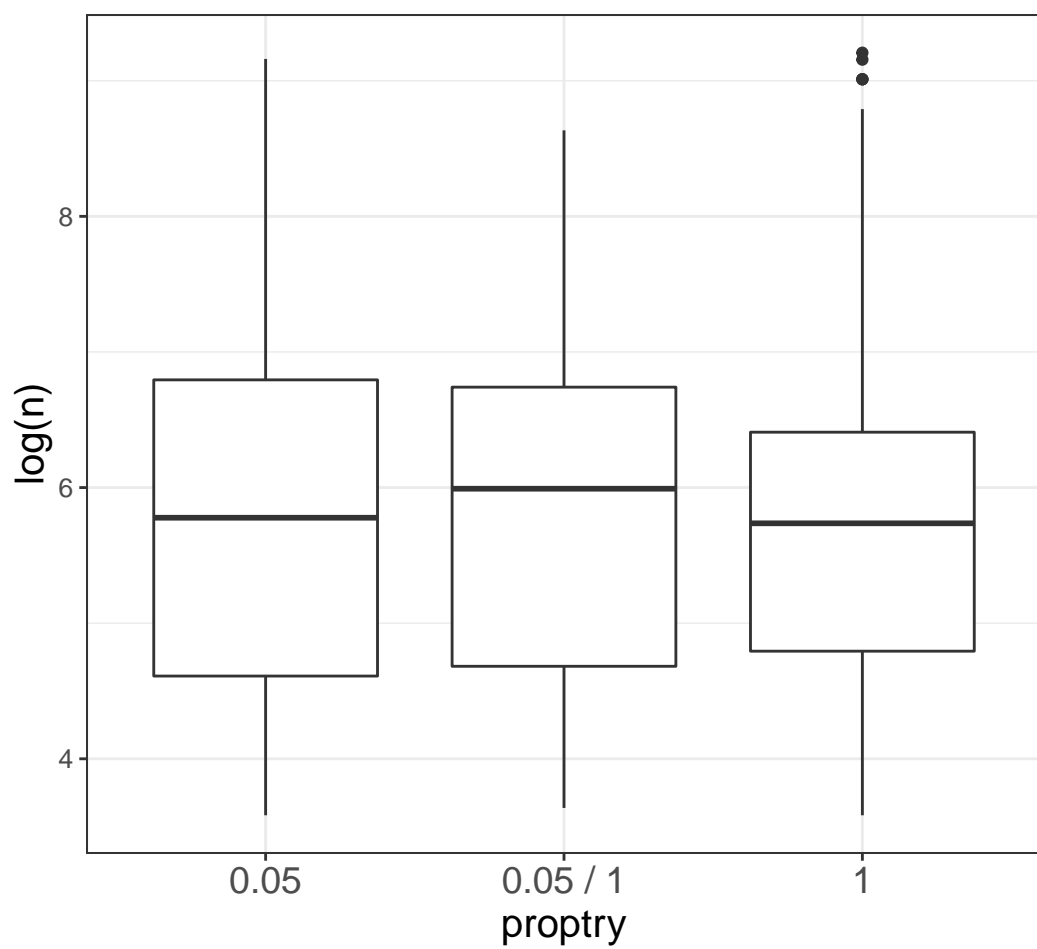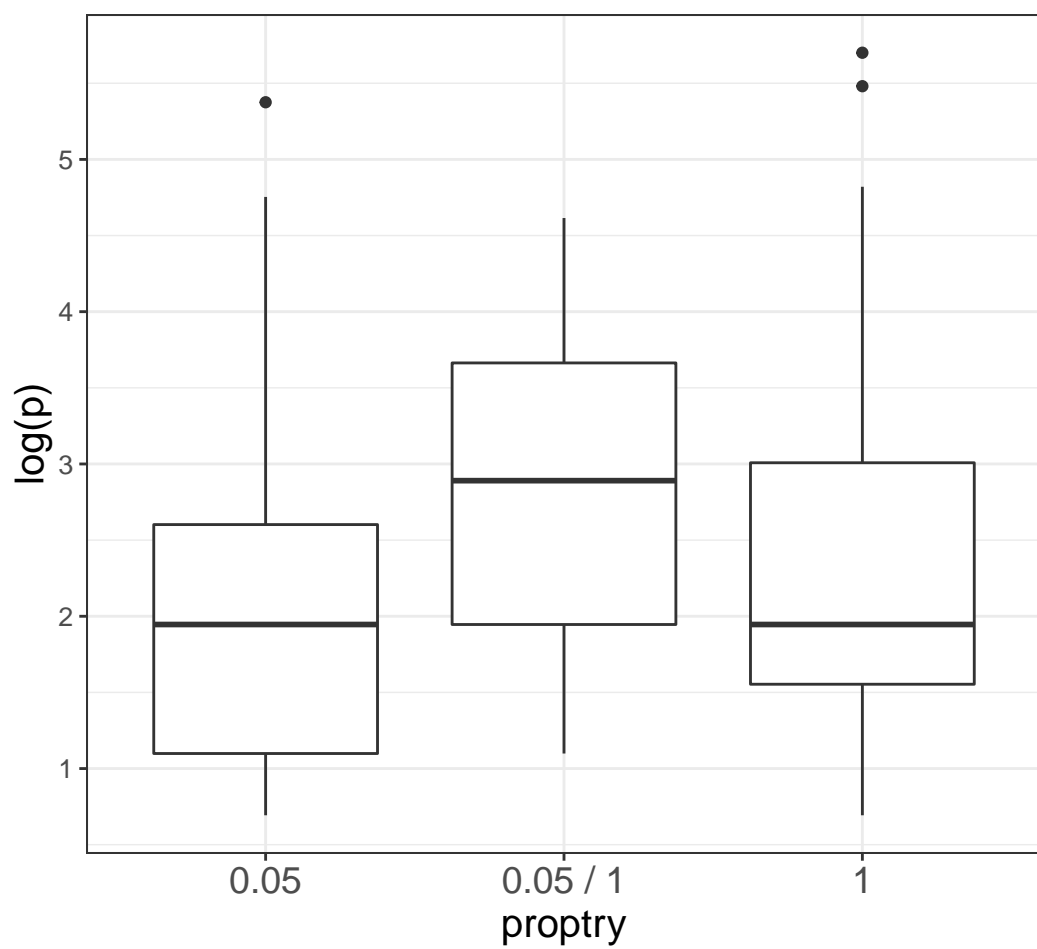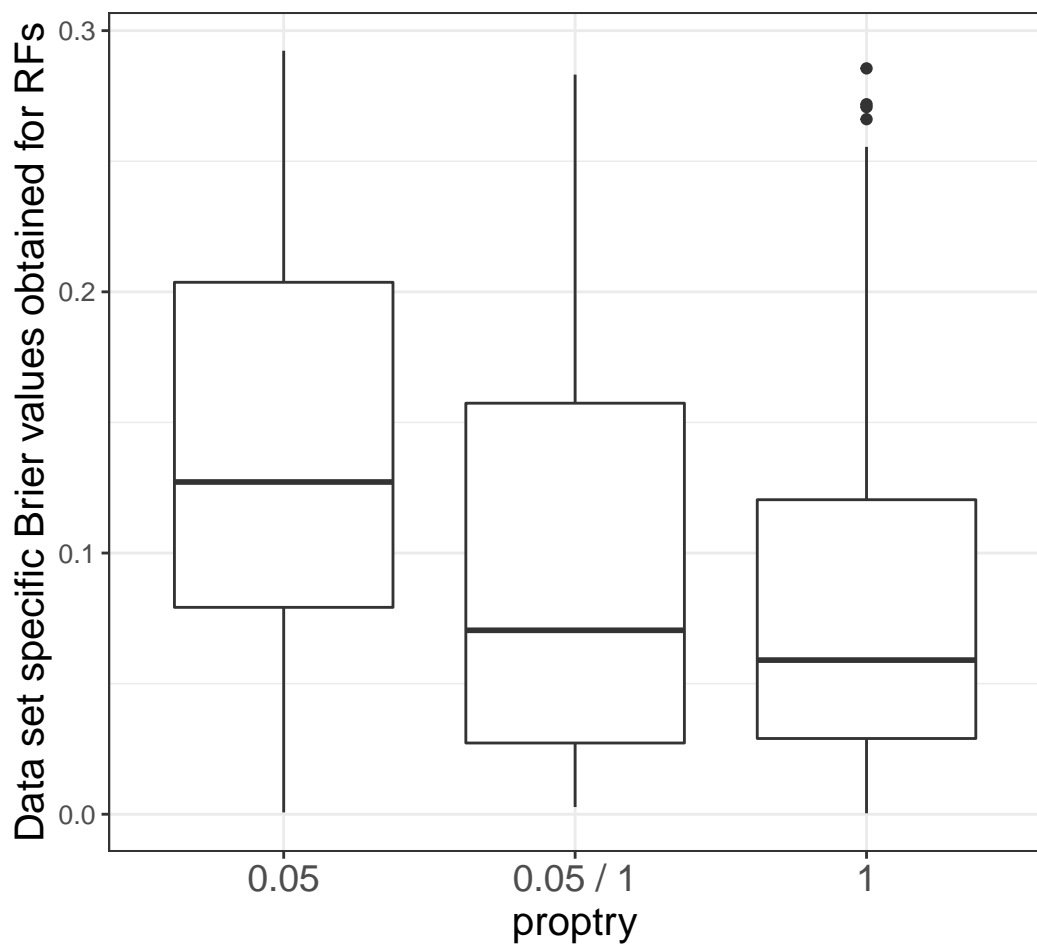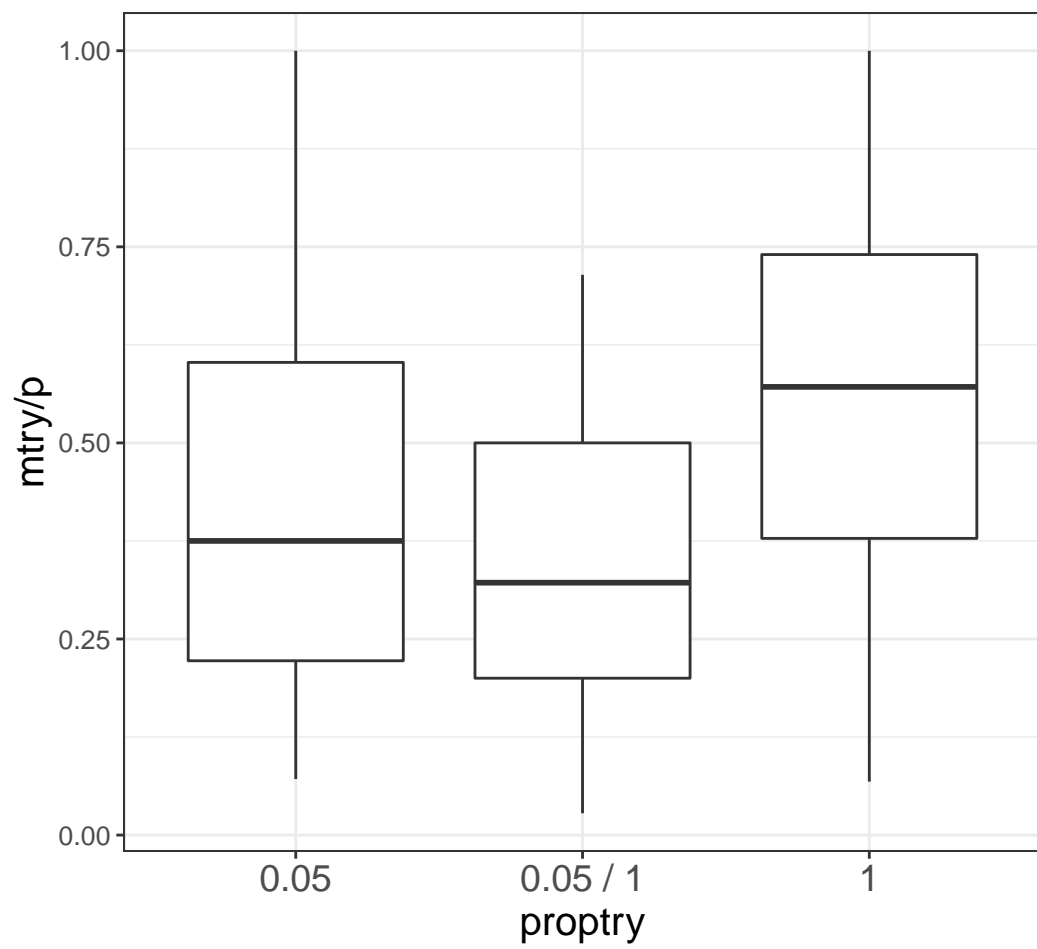

Supplement: Supplementary file 2 — Supplementary file1 (ZIP 108032 KB) [file 42979_2021_920_MOESM2_ESM.zip › Online_Resource_2/Results/Figures/Proptry_brier.pdf]

cross-validated AUC

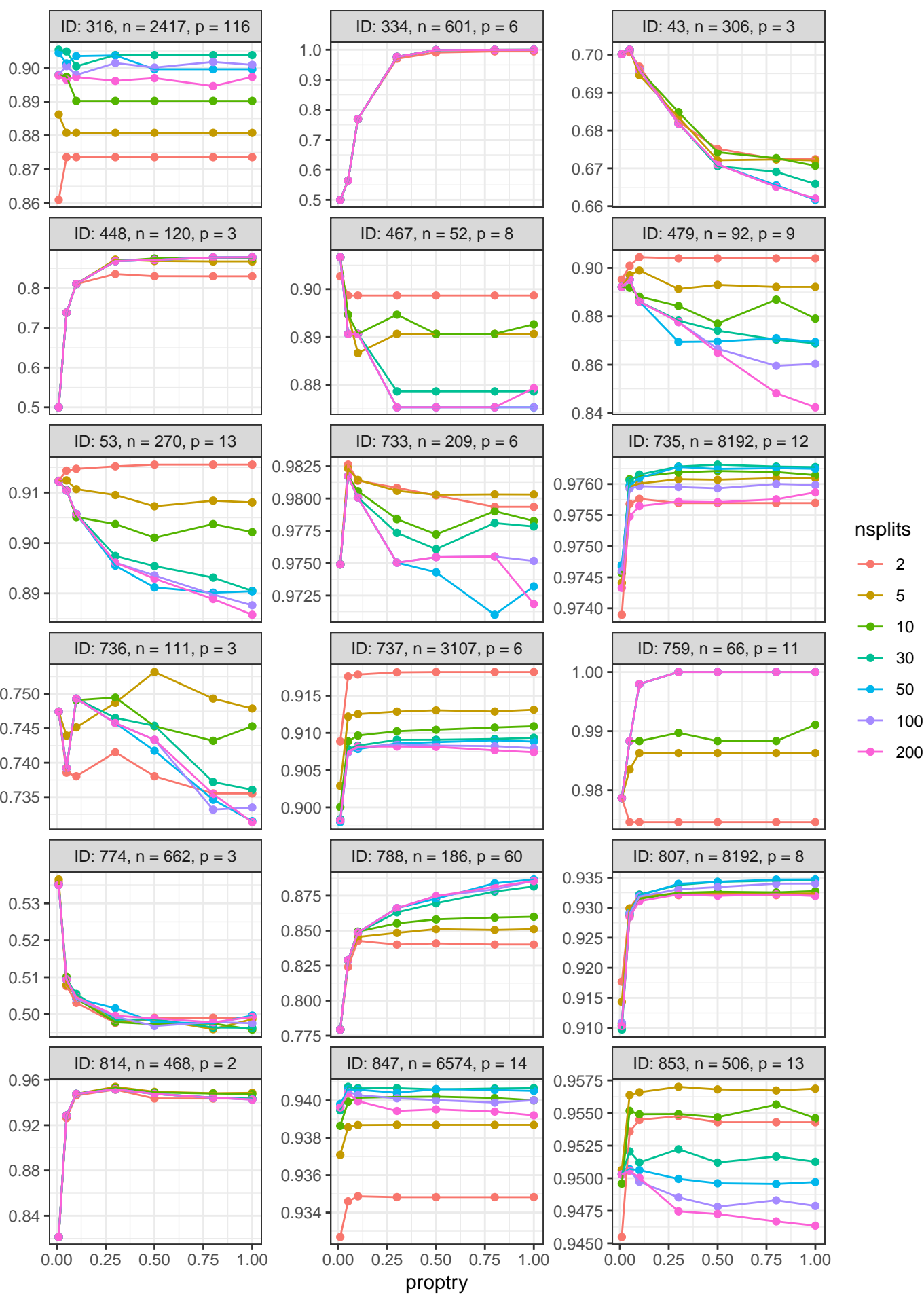

Supplement: Supplementary file 2 — Supplementary file1 (ZIP 108032 KB) [file 42979_2021_920_MOESM2_ESM.zip › Online_Resource_2/Results/Figures/TuningStudy_AllResults_1.pdf]

cross-validated AUC

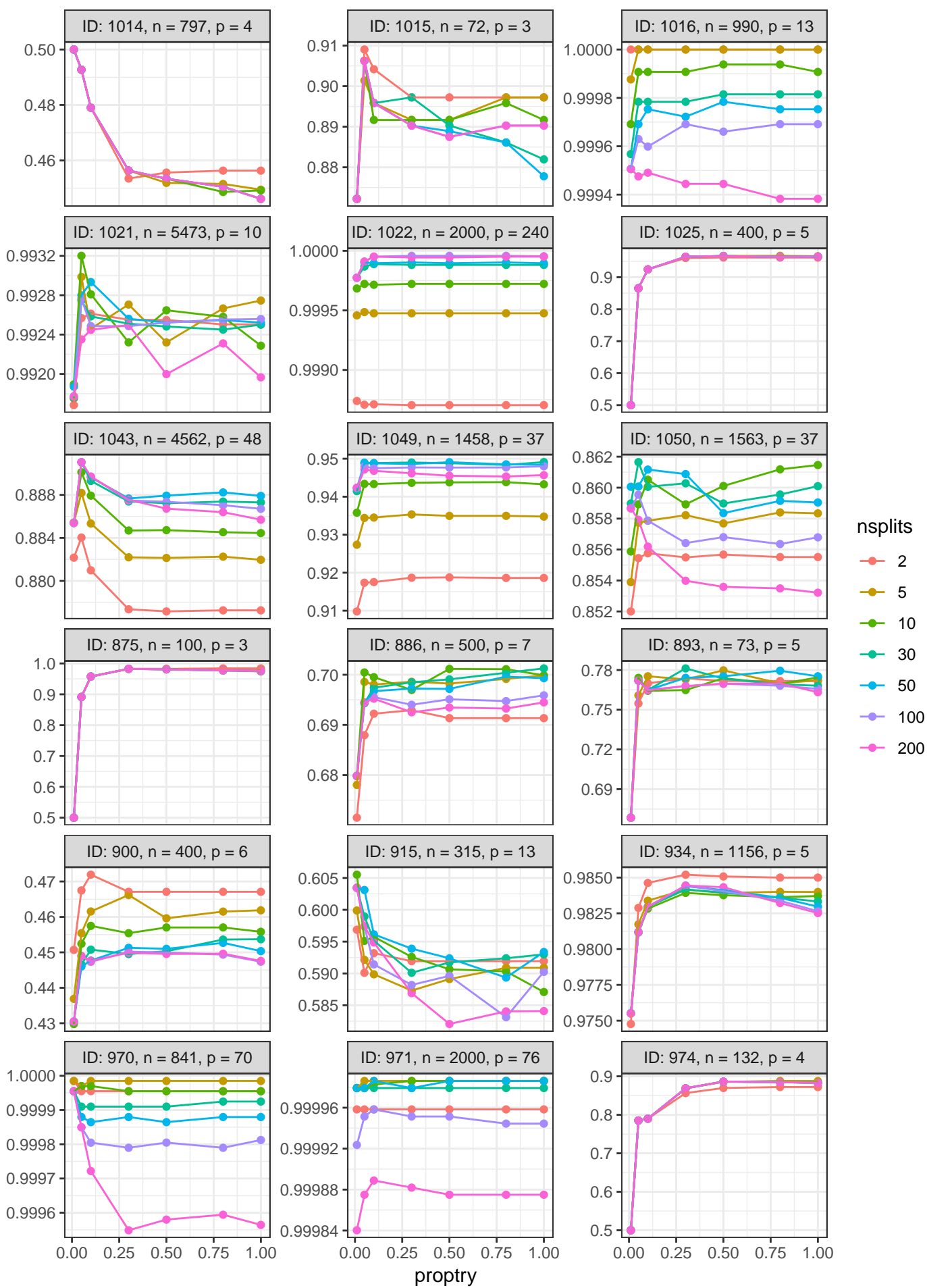

Supplement: Supplementary file 2 — Supplementary file1 (ZIP 108032 KB) [file 42979_2021_920_MOESM2_ESM.zip › Online_Resource_2/Results/Figures/TuningStudy_AllResults_2.pdf]

cross-validated AUC

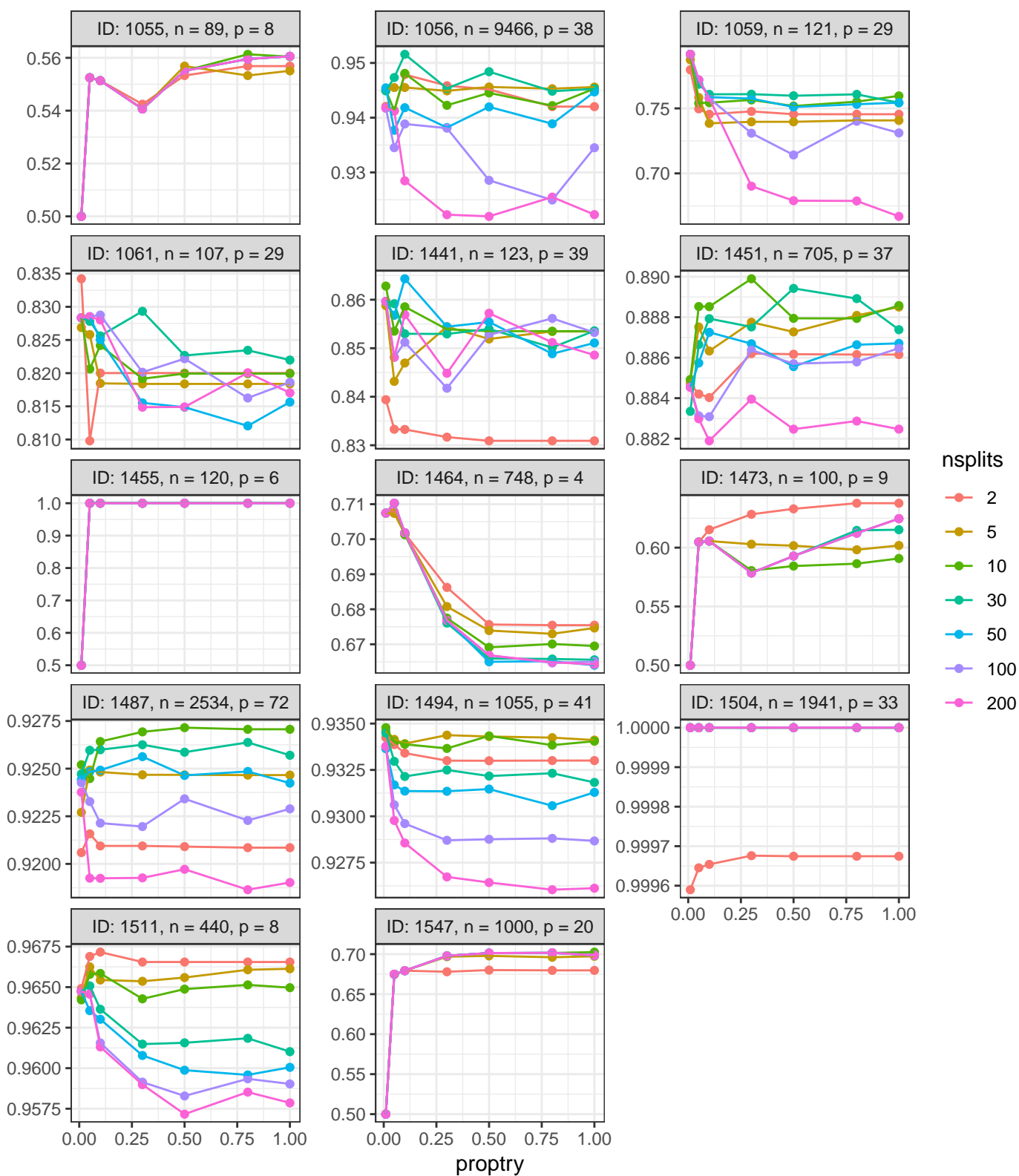

Supplement: Supplementary file 2 — Supplementary file1 (ZIP 108032 KB) [file 42979_2021_920_MOESM2_ESM.zip › Online_Resource_2/Results/Figures/TuningStudy_AllResults_3.pdf]

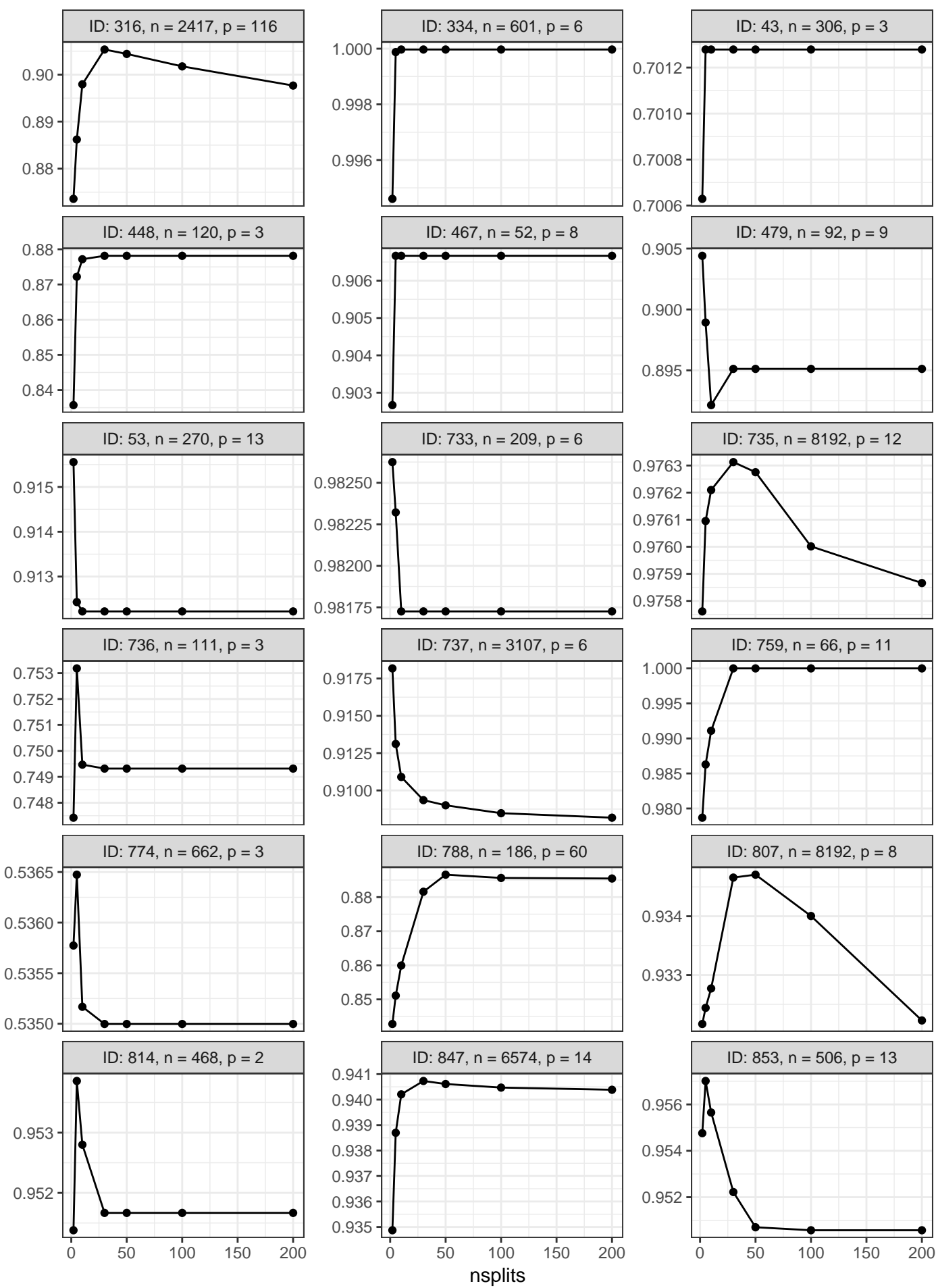

Supplement: Supplementary file 2 — Supplementary file1 (ZIP 108032 KB) [file 42979_2021_920_MOESM2_ESM.zip › Online_Resource_2/Results/Figures/TuningStudy_nsplits_1.pdf]

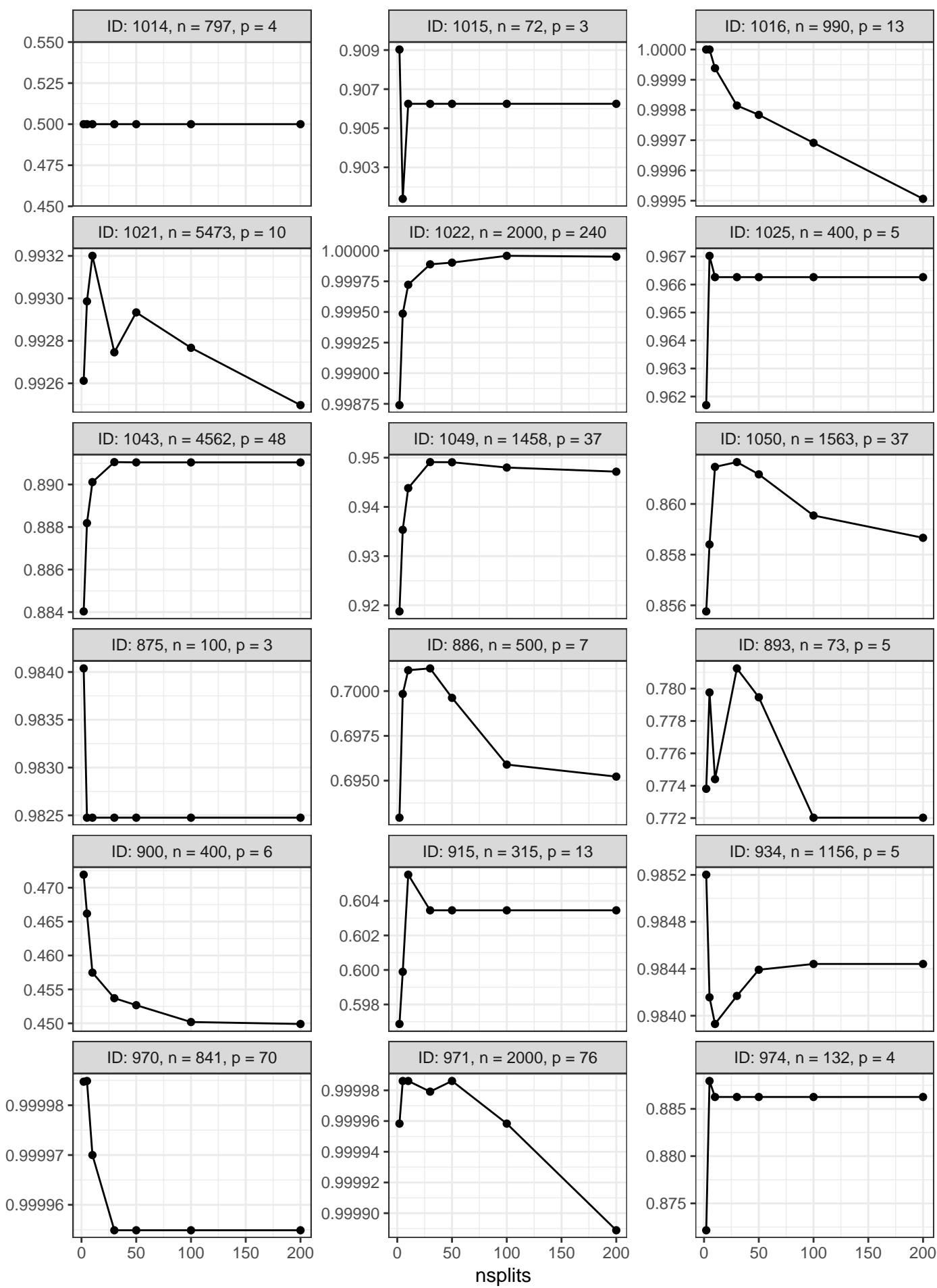

Supplement: Supplementary file 2 — Supplementary file1 (ZIP 108032 KB) [file 42979_2021_920_MOESM2_ESM.zip › Online_Resource_2/Results/Figures/TuningStudy_nsplits_2.pdf]

cross-validated AUC

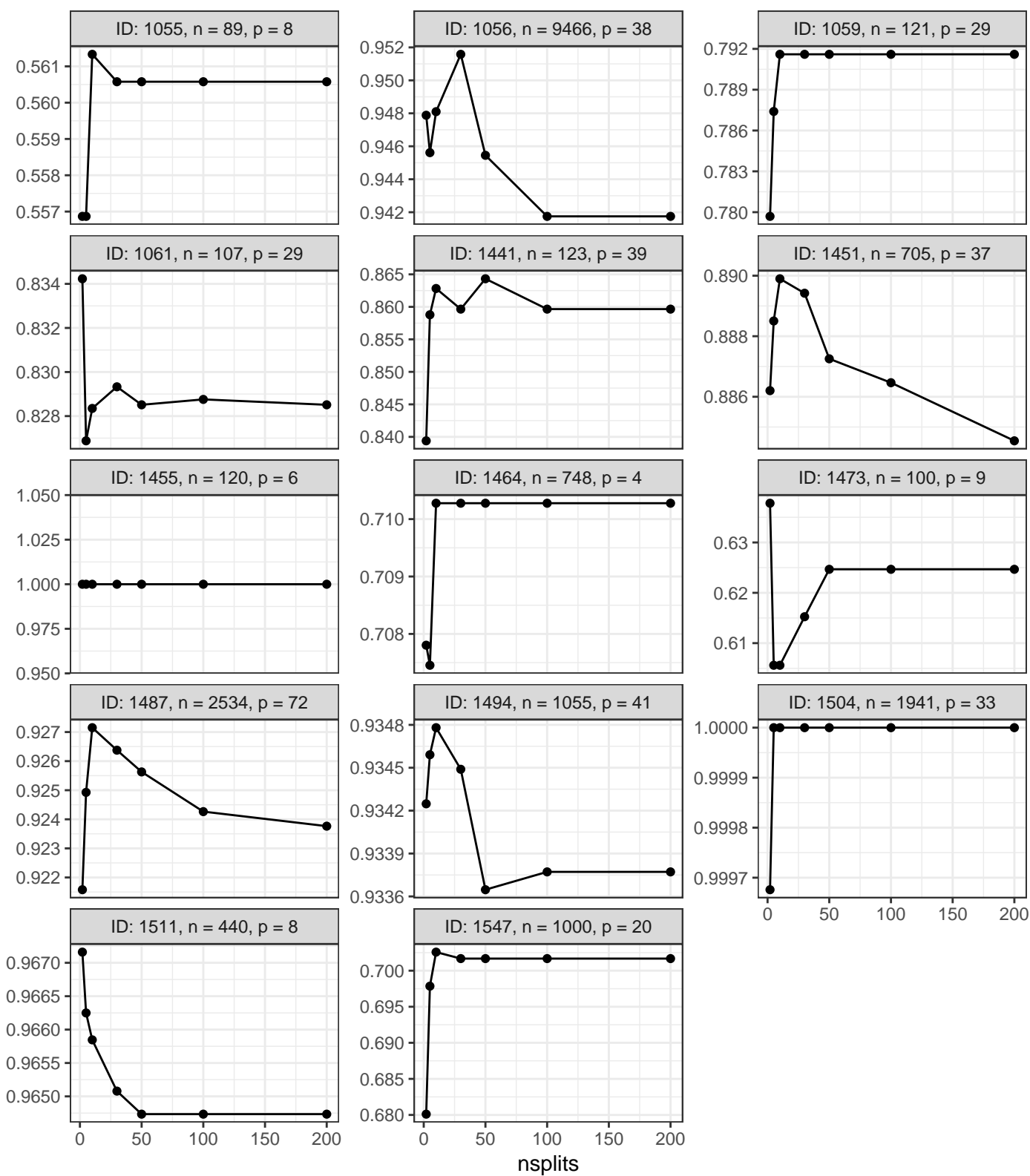

Supplement: Supplementary file 2 — Supplementary file1 (ZIP 108032 KB) [file 42979_2021_920_MOESM2_ESM.zip › Online_Resource_2/Results/Figures/TuningStudy_nsplits_3.pdf]

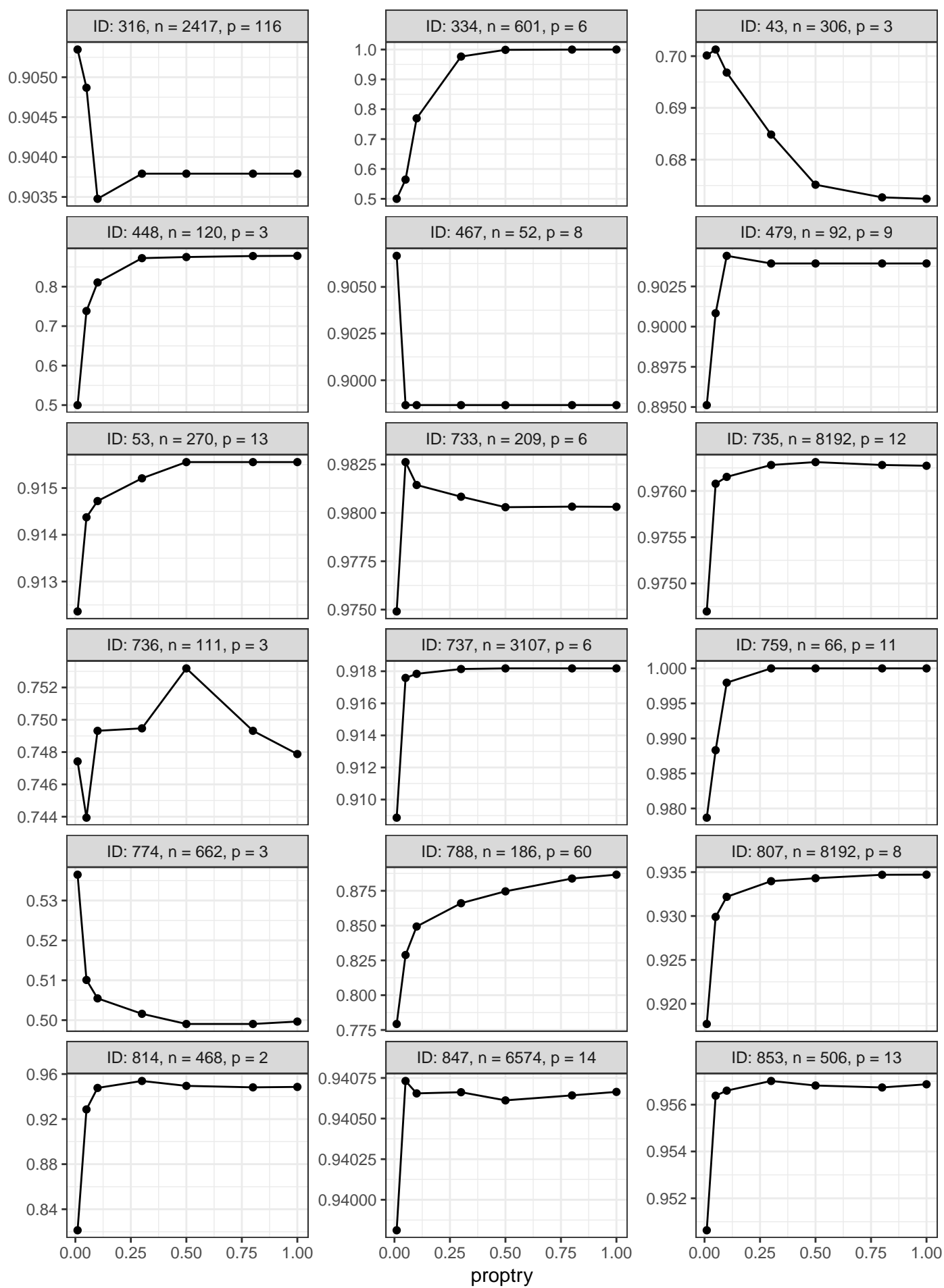

Supplement: Supplementary file 2 — Supplementary file1 (ZIP 108032 KB) [file 42979_2021_920_MOESM2_ESM.zip › Online_Resource_2/Results/Figures/TuningStudy_proptry_1.pdf]

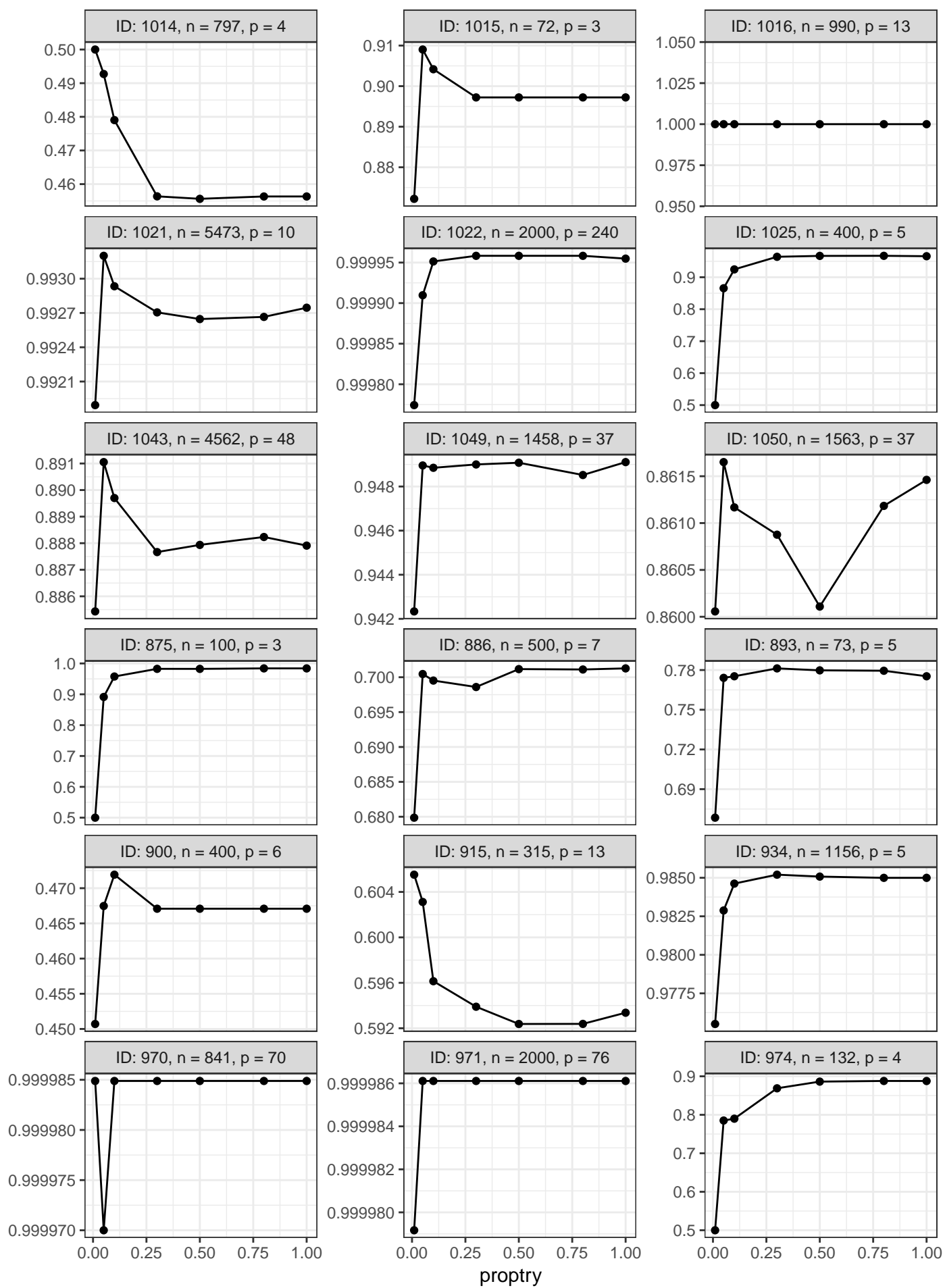

Supplement: Supplementary file 2 — Supplementary file1 (ZIP 108032 KB) [file 42979_2021_920_MOESM2_ESM.zip › Online_Resource_2/Results/Figures/TuningStudy_proptry_2.pdf]

cross-validated AUC

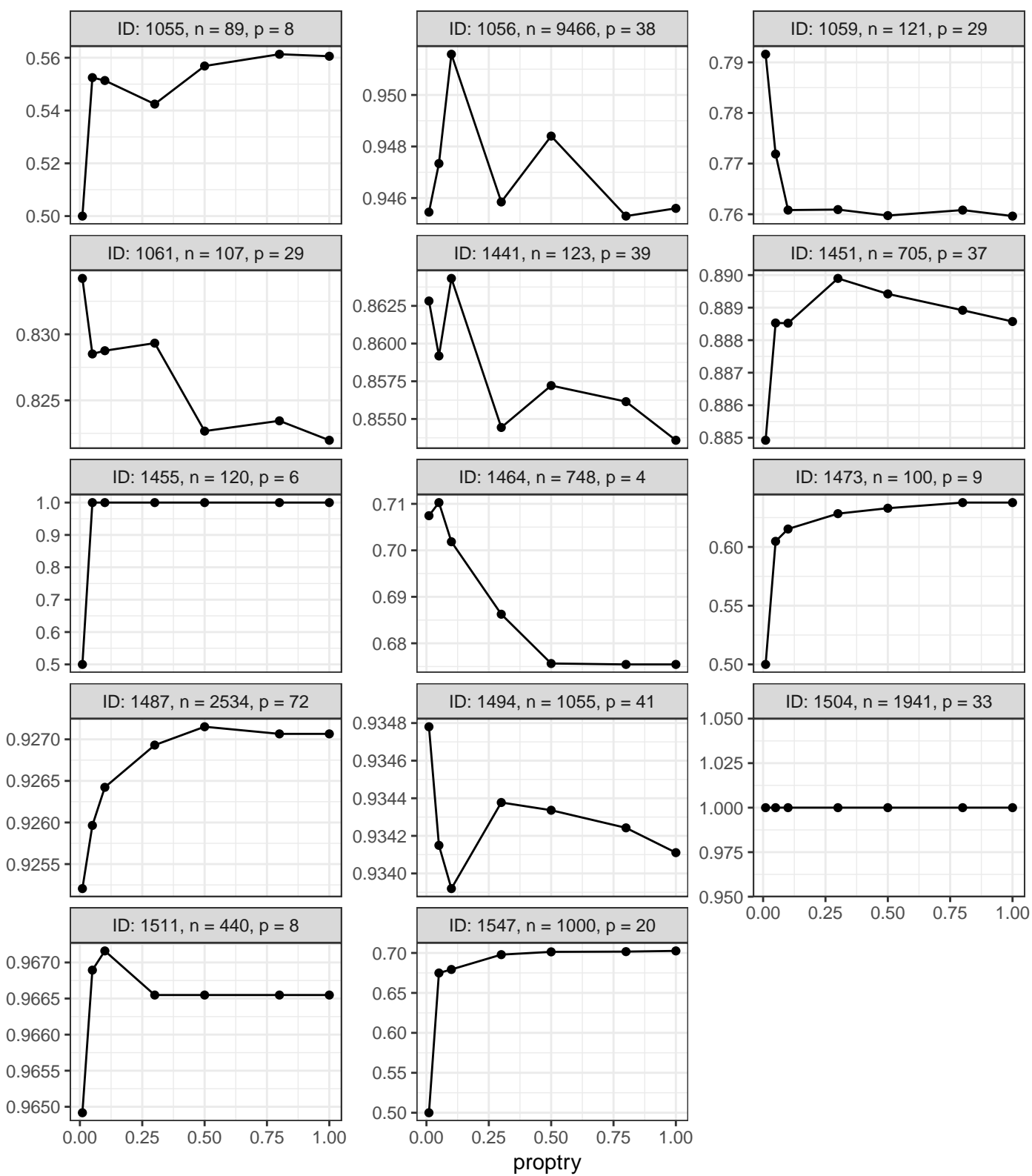

Supplement: Supplementary file 2 — Supplementary file1 (ZIP 108032 KB) [file 42979_2021_920_MOESM2_ESM.zip › Online_Resource_2/Results/Figures/TuningStudy_proptry_3.pdf]
